# Exploring chemical space for iridium(iii) complexes: a direct-to-biology (D2B) approach to identifying anticancer and antibacterial agents

**DOI:** 10.1039/d6sc04917f

**Published:** 2026-08-21

**Authors:** Timothy Kench, Yiran Wang, Jana Seefeldt, Aatikah Majid, Alex Man-Hei Yip, Justin Shum, Gianmarco G. Terrones, Yassin Antar, Benedikt V. Holbling, Kenneth Kam-Wing Lo, Nils Metzler-Nolte, Heather J. Kulik, Ramon Vilar

**Affiliations:** a Department of Chemistry, Imperial College London, White City Campus W12 0BZ London UK t.kench17@imperial.ac.uk r.vilar@imperial.ac.uk; b Department of Chemical Engineering, Massachusetts Institute of Technology 02139 Cambridge MA USA hjkulik@mit.edu; c Faculty of Chemistry and Biochemistry, Inorganic Chemistry I - Bioinorganic Chemistry, Ruhr University Bochum, Universitätsstrasse 150 Bochum 44801 Germany; d Department of Chemistry, City University of Hong Kong Tat Chee Avenue Hong Kong P. R. China; e Drug Discovery Hub, Translation & Innovation Hub Building, Imperial College London, White City Campus W12 0BZ London UK; f Department of Chemistry, Massachusetts Institute of Technology Cambridge 02139 MA USA

## Abstract

Metal complexes possess tuneable chemical and biological properties that make them promising candidates for anticancer and antibacterial therapies. They are increasingly explored as alternatives to conventional drugs, particularly in photodynamic therapy (PDT), where light activation induces reactive oxygen species for localised cytotoxicity. In this study, we present a direct-to-biology (D2B) approach involving the synthesis and screening of 336 iridium(iii) complexes. Using an automated, data-driven approach, we evaluate ROS generation, lipophilicity, cytotoxicity in the dark and light, cellular uptake, and localisation across normal, cancer and bacterial cell lines. Information gained from subcellular localization studies was translated and checked against immunogenic cell death (ICD)-inducing properties of selected complexes. This large, internally consistent dataset was used to train machine learning models that predict both physicochemical properties and biological responses using inexpensive xTB-level descriptors. Virtual high-throughput screening of a >200 000-member library of Ir complexes reveals a clear divergence in chemical space between antibacterial and anticancer activity, providing actionable design rules for next-generation complexes. This work demonstrates the synergy between experimental and computational workflows, identifying lead compounds for anticancer and antibacterial applications and generating systematic, high-quality datasets for data-driven discovery.

## Abstract 2

A direct-to-biology approach was used to screen 336 Ir^3+^ complexes against bacteria and cancer cells. High throughput experiments and ML models revealed divergent antibacterial and anticancer chemical space.

## Graphical abstract

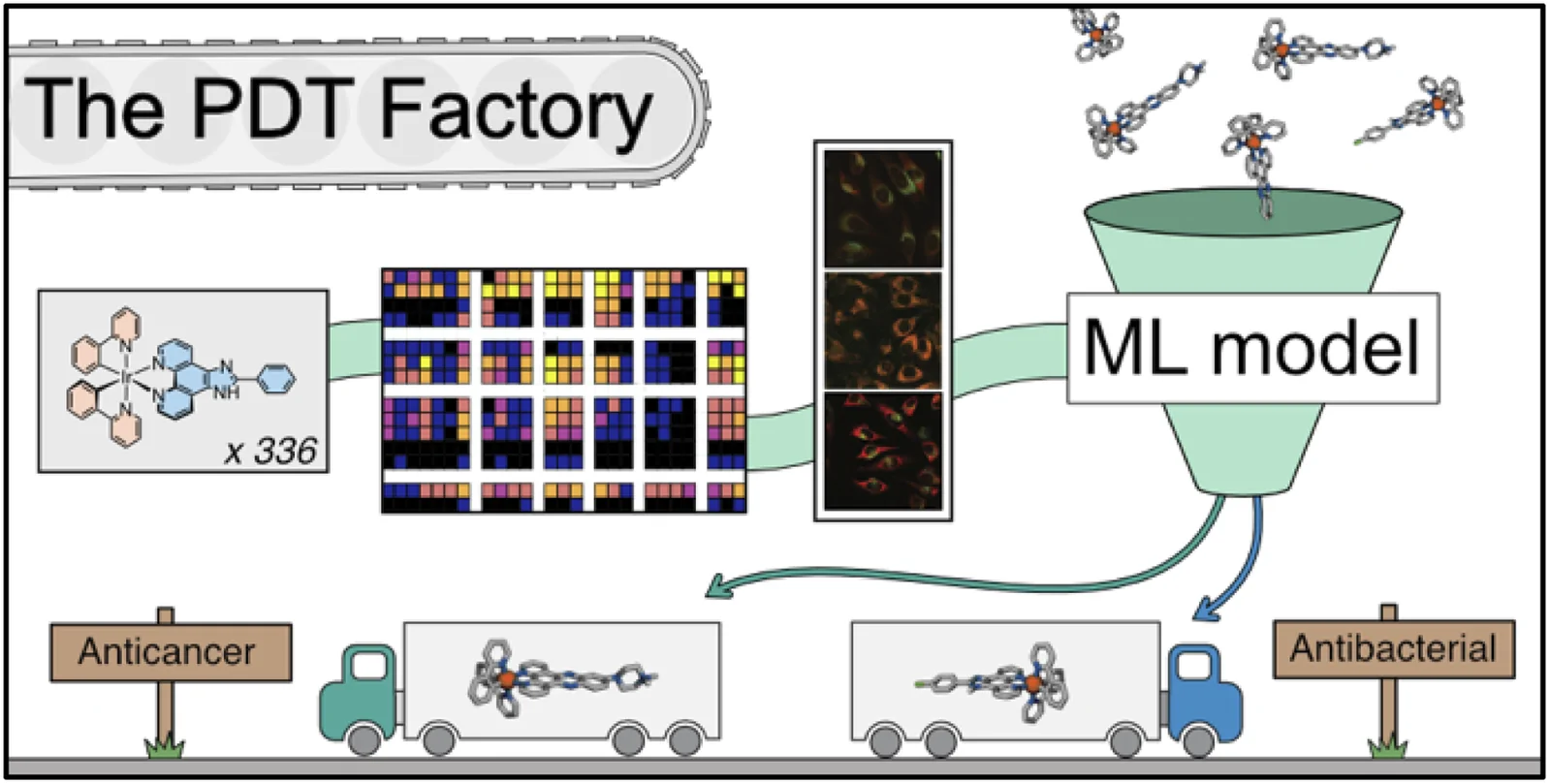

## Introduction

Metal complexes possess unique chemical and biological properties, which can be exploited to develop anticancer^1–4^ and antibacterial^5–9^ therapies with distinct modes of action.^10–14^ Unlike purely organic compounds, metal complexes can adopt diverse coordination geometries, have variable oxidation states, and their properties can be readily tuned by the ligands coordinated to the metal centre.^15–19^ This allows precise control over their reactivity, solubility, physicochemical properties, cellular permeability and bio-targeting capabilities. In cancer treatment, metal complexes – such as platinum-based drugs^20,21^ – have already demonstrated clinical success, inspiring the development of a broad range of different metal complexes with improved selectivity and reduced side effects as anticancer agents.^22–30^ More recently, there has been increasing attention in the use of metal complexes as antibacterial agents with distinct modes of action, making them attractive alternatives against antibiotic-resistant strains.^31–34^ Efforts such as the Community for Open Antimicrobial Drug Discovery (CO-ADD)^35^ and combinatorial libraries have shown that metal complexes can reach a significantly higher hit rate than purely organic compounds.^36–38^

One particularly attractive property of metal complexes is the possibility of activating them by light irradiation, triggering targeted cytotoxic effects. Several complexes – mainly with transition metals such as ruthenium(ii), iridium(iii), and platinum(ii) – can act as photosensitisers in photodynamic therapy (PDT), where light irradiation induces the formation of reactive oxygen species (ROS) that selectively damage biomolecules in illuminated regions where the metal complex accumulates, minimizing harm to healthy tissue.^18,39–43^ Complexes with these metals are especially attractive because of their long-lived triplet excited state, efficient intersystem crossing and stability, with numerous examples reported.^44–52^ Furthermore, their photophysical properties can be finely tuned through ligand design, enabling control over light absorption wavelengths, excited-state lifetimes, and energy transfer efficiency.^15,19^ In oncology, this allows for precise eradication of tumour cells while avoiding systemic toxicity; in antibacterial applications, it provides an effective means to combat drug-resistant pathogens *via* localised photoactivation. The dual advantages of spatial selectivity and reduced resistance development make photoactive metal complexes an exciting frontier in both anticancer and antimicrobial therapy.

Realising this potential requires efficient exploration of the ligand space available around a given metal centre. Metal fragment-based drug design offers a modular strategy in which a defined metal fragment is combined with diverse ligand sets to rapidly generate candidate libraries through combinatorial chemistry, and when coupled with automated screening and machine learning (ML), creates a data-driven pipeline for hit discovery.^53–61^

**Fig. 1:**
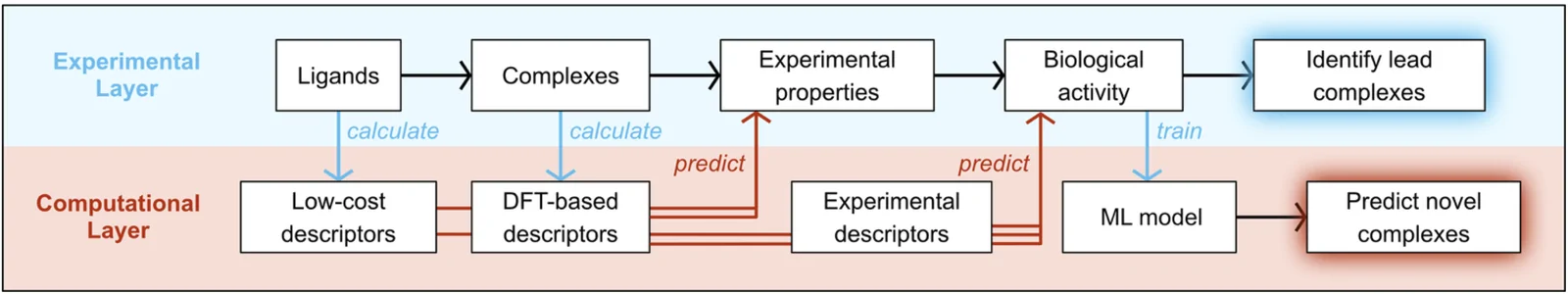
Schematic overview of the experimental and computational workflows, and their synergies.

In this paper we present a direct-to-biology (D2B) approach for a library of 336 iridium(iii) complexes, in which the complexes progress directly from synthesis to screening (Fig. 1). Physicochemical properties have been measured alongside biological endpoints spanning both dark and light toxicity across two cancer cell lines, a normal mammalian cell line and two bacterial strains. From these data, lead complexes were selected for detailed studies in cancer cells, with a particular focus on whether these complexes are photo-inducers of immunogenic cell death (ICD). Selected complexes, triggered the release of damage-associated molecular patterns (DAMPs), consistent with ICD and therefore represent leads for further development as anticancer agents. In parallel, we have developed a computational workflow, culminating in the coupling of inexpensive xTB-level descriptors with machine learning to rationalise and predict complex behaviour (Fig. 1). Virtual high-throughput screening of a synthetically achievable >200 000-member library reveals a fundamental functional difference between the two ligand classes in heteroleptic [Ir(CN)_2_(NN)]^+^ complexes: the CN ligand acts as a gatekeeper that determines whether a complex enters the biologically active chemical space at all, while the NN ligand dictates target selectivity between antibacterial and anti-cancer activity. These findings move beyond retrospective SAR, providing actionable rules for the rational development of next-generation photoactive metal complexes.

## Results and discussion

### Library design and synthesis

A primary aim of high-throughput chemistry is the facile generation of large libraries of compounds, which can be used to identify high-performing leads for further development and generate large datasets of consistently collected measurements, analyses of which can accelerate future discovery. This can be through the formation of general rules or guidelines based on structure–activity relationships (SARs), such as Lipinski's Rule of Five, or *via* prediction of as-yet-unmade compounds using machine learning (ML) models. In both cases, to make good predictions, it is important to not only cover a wide region of chemical space, but to do so consistently without large gaps.

**Fig. 2:**
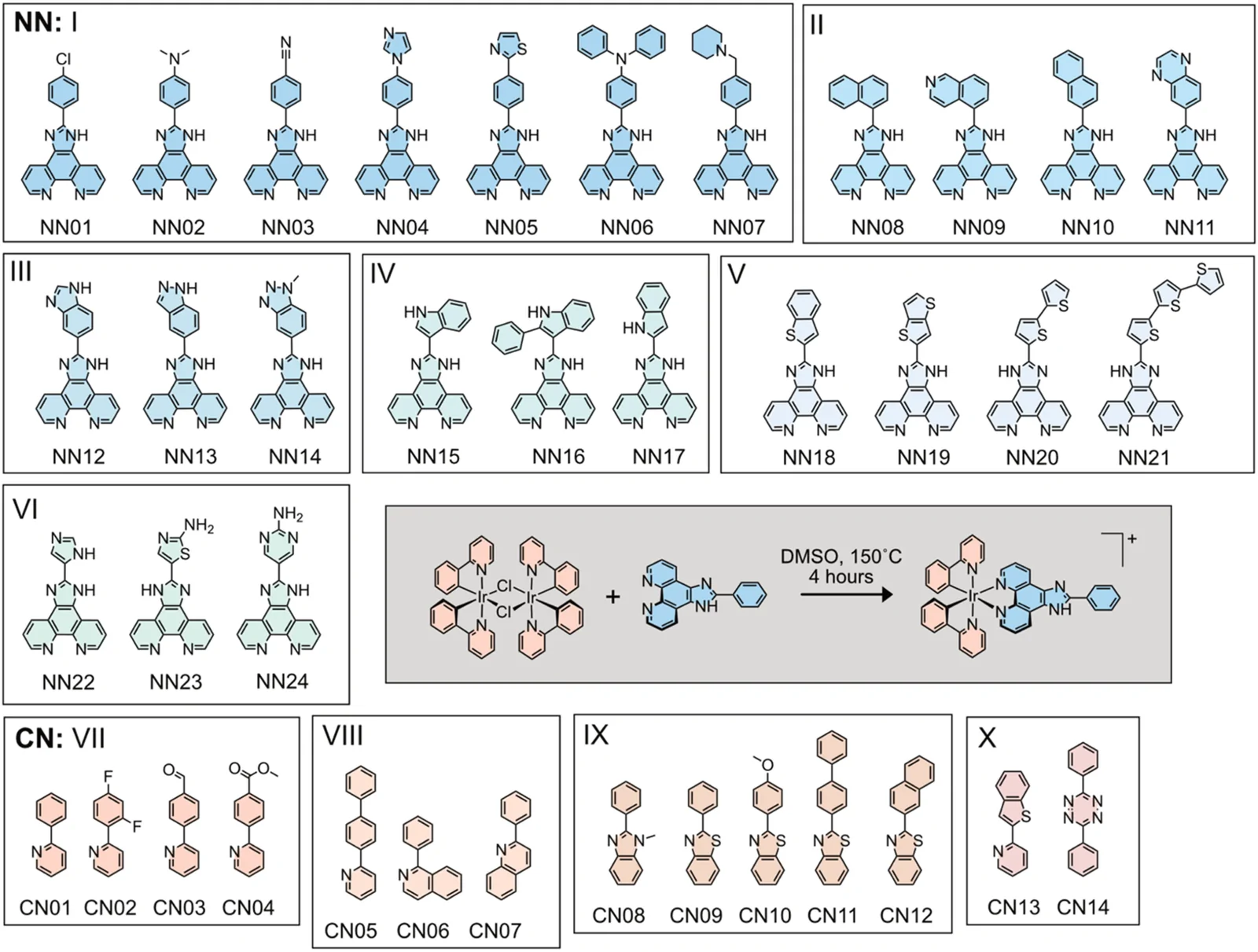
NN and CN ligands used as ‘fragments’ to generate the HTS336 library, and reaction scheme used for the high-throughput synthesis of the iridium(iii) complexes.

Metal complexes are particularly advantageous in this case, due to the potential for high-yielding coordination of ligands around a metal centre scaffold.^36–38,54,62–69^ We previously reported a library of 90 [Ir(CN)_2_(NN)]^+^ complexes generated *via* a combinatorial approach of CN and NN ligands (where CN and NN correspond to bidentate cyclometallating and bidentate *N*-heterocyclic based ligands, respectively).^62^ Here, we have expanded that library nearly four-fold, to 336 iridium(iii) complexes (which we refer to as HTS336 – see Fig. 2), through the combination of 24 unique NN ligands and 14 unique CN ligands. We focus solely on phenanthroline-imidazole (PI) type NN ligands, as these ligands can be easily synthesised in a high-yielding condensation reaction between phenanthrolinedione and an aldehyde, allowing access to a very diverse range of ligands (and hence complexes) from this very common building block.

Synthesis was carried out in an analogous manner to that previously reported, and purity analysed *via* LCMS. Generally, excellent conversion was observed, with 92% of the library reaching >75% conversion, and only 8 complexes showing <60% conversion (Fig. S1, see SI for Experimental and Characterisation Details).

This approach can be compared to more traditional procedures, wherein smaller libraries of a handful of compounds are synthesised, purified and tested, and literature knowledge is built up over time; recently, Bezzubov and co-workers published a dataset of experimental cytotoxicity values from 803 iridium(iii) complexes (covering the literature from 2008 to 2022), nearly half of which are [Ir(CN)_2_(NN)]^+^-type complexes (termed IrCytoToxDB).^70^ Analysis of the chemical space contained within this library subset (Fig. S2, 244 unique ligands forming 355 unique complexes) reveals that: (i) a significant portion of complexes in IrCytoToxDB contain 2-phenylpyridine as the CN ligand (148/355, 42%), demonstrating how when a more complex NN ligand is incorporated, researchers generally only use a very simple CN ligand. (ii) When researchers do vary CN ligand complexity, they often only test a single NN ligand, leading to 2-phenylpyridine becoming the *de facto* choice of CN ligand in the field, even when there are other commercially available building blocks that might impart superior activity. (iii) Filtering IrCytoToxDB for a given set of biological conditions for systematic comparisons, dramatically reduces the number of available compounds within that dataset (Fig. S3).

In contrast, our library (*i.e.*HTS336) uses only 38 ligands, all of which are either commercially available or easily prepared, to synthesize a library of comparable size to IrCytoToxDB. Furthermore, as will be shown in this work, each of these complexes can be tested under identical conditions, improving the quality of the resulting dataset. Finally, the chemical space of the resulting library is very different; there is no over-representation of 2-phenylpyridine or clustering along a single axis. Whilst the overall area of chemical space of HTS336 is reduced compared with IrCytoToxDB (which is to be expected due to comparatively simpler building blocks), that space is covered systematically and at a higher density.

### Lipophilicity and photophysical properties

For a given complex to be photo-cytotoxic, it must be bioavailable and be able to produce reactive oxygen species under irradiating conditions. To gain insight into these processes, three core experimental properties were determined in a high-throughput manner for the iridium(iii) complexes in HTS336.

**Fig. 3:**
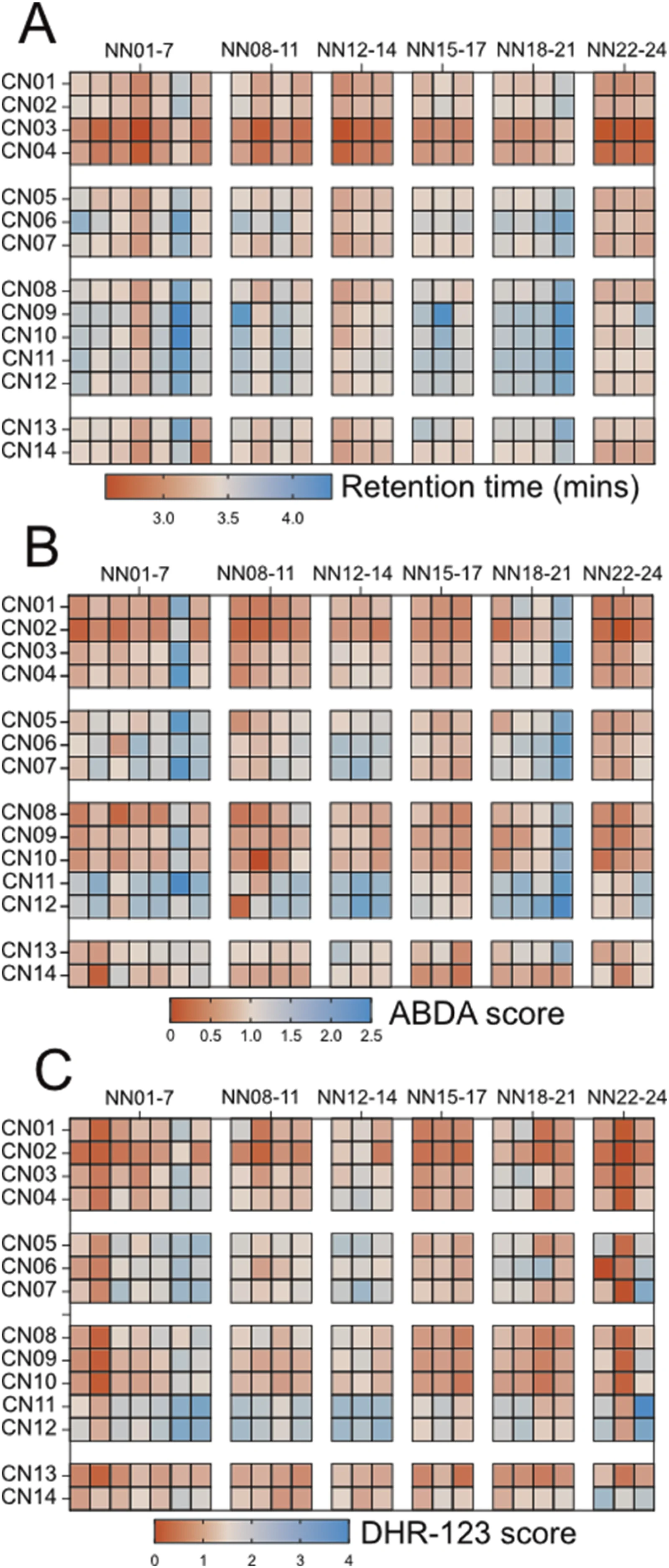
(A) Heat-map of the complexes in the HTS336 library showing RP-LC retention times as a measure of lipophilicity (the higher the retention time – *i.e.* the blue end of the heat map – the more lipophilic the compound is); (B and C) ROS generation measured using ABDA and DHR-123 dyes after irradiating the HTS336 library with blue light (470 nm, 15 mA (∼6 mW flux), 12 min). The higher the score – *i.e.* blue end of the heat map – the higher the ROS production.

Bioavailability correlates strongly with lipophilicity, typically measured *via* log *P* (octanol: water partition coefficient), with extreme values leading to poor cellular uptake. Since experimental log *P* determination is impractical at scale, we used reversed-phase liquid chromatography (RP-LC) retention time as a proxy (the more lipophilic the complex, the better it is retained on a reversed-phase column), which is highly amenable to high-throughput workflows and widely validated (Fig. 3A, core property one).^71–74^ To measure ROS upon light irradiation, experiments were conducted with HTS336 using ABDA (selective for singlet oxygen, ^1^O_2_) and DHR-123 (selective for a wider range of ROS including superoxide radical) assays. The results were normalised to [Ru(bipy)_3_]^2+^ to obtain the ROS assay scores (see Fig. 3B and C for overview, and SI Fig. S4–6 for values of all three properties). Importantly, both assays can be carried out in aqueous conditions and performed in high throughput.

As expected, trends in the complex lipophilicity were generally a combination of how hydrophilic or lipophilic each ligand was. For example, those containing multiple ionisable aliphatic or aromatic nitrogen atoms were consistently the most hydrophilic; when combined with CN ligands containing hydrogen-bond acceptor groups (such as CN03 and CN04), comparatively very low retention times were obtained. Conversely, combining ligands such as NN06 (with a diphenyl aniline functional group) or NN21 (terthiophene) with benzothiazole-containing CN ligands (CN09–12) led to complexes with long retention times, *i.e.* highly hydrophobic complexes. This data also shows how retention time alone does not capture nuances in structure. For example, [Ir(CN03)_2_(NN06)]^+^ contains both a very hydrophilic and hydrophobic component and [Ir(CN01)_2_(NN01)]^+^ has ligands of intermediate hydrophilicity; they have similar retention times but would have a very different polar surface area.

The ROS data (Fig. 3B and C) shows a broad range of activities depending on the CN and NN ligands with some library-wide trends emerging, and some key differences between assays. For example, most complexes containing CN11 or CN12, score highly on both assays, while those with CN02 and CN08–10, generally score poorly. For the NN ligands, the clearest trends are observed for NN06, which scores highly on both assays; in contrast, NN19–21, score very highly on the ABDA (Fig. 3B) but not on the DHR-123 assay (Fig. 3C). For some of the above, the data is reasonably straightforward to rationalise based on Δ*E*(T_1_–S_0_) values, which need to be in the correct range to match that of the ^3^O_2_ → ^1^O_2_ transition. As we have previously shown, fragments such as CN02 have a very high Δ*E* and low energy HOMO, which presumably leads to worse overlap with the oxygen ground state. Increasing the conjugation (such as in CN06, 7, 11 and 12) decreases Δ*E*, leading to better overlap.^62^ Whilst the NN ligand has little effect on HOMO, LUMO and Δ*E*, we reinforce here that in an aqueous environment, the NN ligand plays a very important role in determining ROS quantum yields. Further rationalisation based solely on inspection of functional groups is difficult and full DFT/TDDFT calculations are computationally costly on a large library of complexes. As will be discussed later, to further rationalise this data, ML models were developed (see below) using inexpensive descriptors computed with semi-empirical methods.

### Biological activity

Having successfully measured some key physicochemical properties relating to the potential biological activity of a given complex, we established a high-throughput cellular assay workflow capable of rapidly obtaining multi-point screening data against a range of cell lines. We screened HTS336 in the light (12 minute, 470 nm irradiation – see Fig. S7 for control experiments with irradiation of cells in the absence of any iridium complex) and in the dark against two cancer cell lines (HeLa and AT3), a non-cancerous cell line (HEK293), a Gram-positive bacterium (*Bacillus subtilis*) and a Gram-negative bacterium (*Escherichia coli*). To achieve this, we used a screening cascade in which the library was tested at a few initial concentrations and active complexes were re-screened to obtain half maximal inhibitory concentration (IC_50_) values for mammalian cells, or minimum inhibitory concentration (MIC) data for bacteria.

**Fig. 4:**
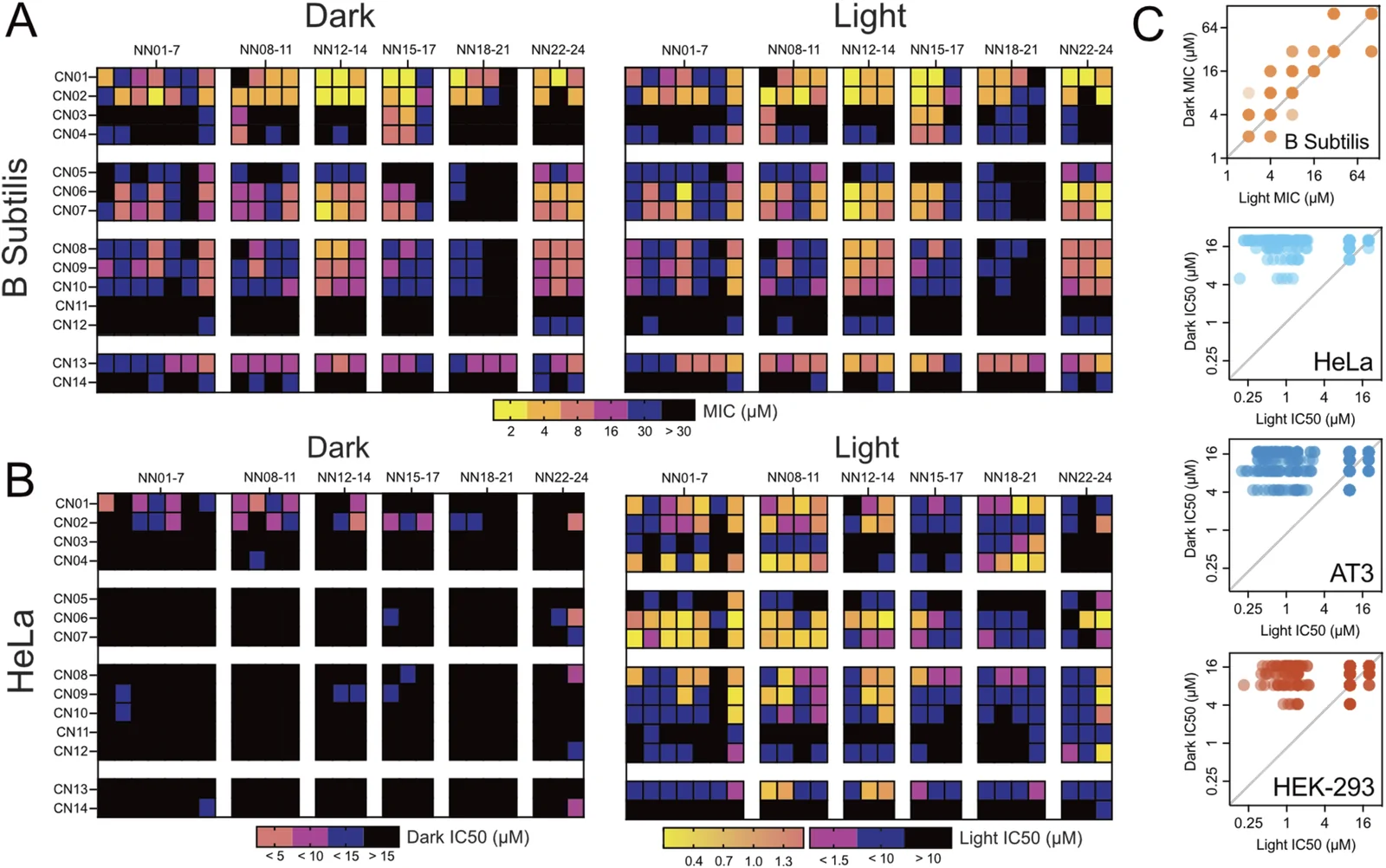
Representative screening results (shown as heat maps) for HTS336 against *B. subtilis* (A) and HeLa (B) in the dark and after light irradiation (470 nm, 15 mA, 12 min) and (C) library-wide light *vs.* dark behaviour, showing two distinct modes of action for bacteria and mammalian cells. Note that for MIC values the categorisation into discrete numbers leads to much fewer visible dots (darker dots = more overlapping data points).

A broad range of activity across all cell lines was observed, with representative heatmaps for HeLa cells and *B. subtilis* shown in Fig. 4 (see Fig. S8 for AT3 and HEK cell lines). Unfortunately, little-to-no activity was seen for the entire library against *E. coli*, in line with previous reports for other families of metal complexes,^36,37^ and presumably caused by a lack of permeability through the outer membrane.^75,76^

Fascinatingly, the activities of the library against mammalian cell lines and *B. subtilis*, are very different (Fig. 4A and B). For *B. subtilis*, many complexes have similar light and dark activity, whereas in HeLa cells, almost no complexes are active in the dark, with a marked increase in activity upon light irradiation. To quantify this, the dark IC_50_ and dark MIC values were plotted against their light counterparts (Fig. 4C). Complexes that are purely cytotoxic (light-independent) fall on or close to the diagonal, while ideal phototoxic complexes (*i.e.* non-toxic in the dark, very toxic in the light), would fall in the top left quadrant. We observe that antibacterial activity is primarily intrinsic to the iridium complex, indicating predominantly a cytotoxic rather than photo-cytotoxic mechanism of action; no complex displays above a 4-fold increase in MIC after irradiation (two dots away from diagonal, Fig. 4C). In contrast, in mammalian cells only the non-active complexes fall on the diagonal, likely due to poor cell permeability. The most phototoxic complexes (top left quadrant, Fig. 4C) display up to 200-fold enhancements upon light irradiation *vs.* dark conditions.

Against *B. subtilis*, a clear trend emerged favouring complexes with small CN ligands lacking hydrogen bond acceptors. Less active CN ligands tended to extend through the cyclometallating portion of the bidentate CN ligand, suggesting steric or electronic factors may hinder antimicrobial efficacy. A comparison between NN08 and NN10 revealed that a positional shift of the coordinating naphthalene ring significantly reduced activity, highlighting the sensitivity of microbial efficacy to subtle structural changes. The NN12–NN16 series stood out, consistently delivering high activity; these ligands incorporate heterocycles such as indole, imidazole, and triazole, which may facilitate favourable interactions with biomolecules. Interestingly, NN17, despite structural similarity to some of the other active NN ligands, showed a sharp decline in activity. This may be attributed to its unique bidentate binding motif *via* 2-indol-benzimidazole, potentially altering interaction profiles. Substituting the 2-indole with a 2-benzothiophene in the presence of potent CN ligands (*e.g.*CN01 or CN02) restored and even enhanced activity. Complexes with NN22–NN24 also showed strong antimicrobial activity, reinforcing the notion that small nitrogen-containing ligands are particularly effective. Pleasingly, NN12 and NN18 were inactive across all mammalian cell lines, suggesting a degree of microbial selectivity. Conversely, complexes containing CN02 and CN13 demonstrated generalised toxicity in mammalian cells, even in the absence of light activation. Across the library, light-induced toxicity remained mild in bacterial assays, even for compounds that exhibited pronounced photocytotoxicity in mammalian cells.

In mammalian cell lines, the most potent ligands were those with extended π-systems. Both NN08–NN11 and CN06–CN07 fall into this category and were associated with high ROS generation, correlating with increased photocytotoxicity. Similar to the bacterial context, CN ligands with high molecular weight or low lipophilicity (*e.g.*CN11–CN12) were generally inactive, even when paired with NN ligands associated with the generation of high ROS (*e.g.*, NN18, NN19 and NN21). This is consistent with well-established drug design principles where lipophilicity and size play a critical role in cellular uptake and efficacy. Interestingly, CN03 and CN04, despite having comparable lipophilicity, showed divergent activity profiles: complexes with the ester-containing CN03 ligands were active, while those with the aldehyde CN04 were not. This contrasts with the results against bacteria, where activity between complexes containing these two ligands was more consistent. Complexes containing NN23 and NN24 presented another interesting case; both showed low ROS scores in biophysical assays, yet complexes with NN24 became highly toxic upon irradiation, while those with NN23 did not.

We next explored whether the activity of the complexes could be systematically correlated to the experimentally determined physicochemical properties (*i.e.* ROS and LC retention time). Given the mixture of categorical and continuous data (single point screening *vs.* IC_50_/MIC data), we first defined a compound as a hit if it had an MIC ≤ 16 µM or an IC_50_ ≤ 1.5 µM. We next examined whether activity could be systematically enriched by “hit rate optimisation” by moving a narrow window along each property (*e.g.* retention time), counting how many compounds inside that window were hits, and comparing this local hit rate to the overall hit rate in the whole library (see Fig. S9 for a representative example). In *B. subtilis*, dark toxicity showed a strong dependence on retention time, with moderately hydrophilic complexes giving almost a two-fold enrichment in hit rate (36% to 70%), consistent with uptake-driven activity. In mammalian cells, photocytotoxicity also displayed an optimal retention time window but with weaker, cell-line-specific trends and a narrow region of apparent selectivity. In contrast, scanning over ROS metrics did not yield any hit-rate improvements, indicating that ROS generation alone does not account for photocytotoxicity (Fig. S10). This suggests that other mechanisms may be at play, such as sub-cellular localisation or specific binding to certain biomolecules.

### Selection of lead complexes for cellular studies

Sub-cellular localisation plays a key role in determining both the mechanism and potency of a bioactive metal complex;^77^ mitochondrial accumulation, for example, is commonly associated with apoptosis, or necrosis.^78^ A particularly promising downstream outcome is immunogenic cell death (ICD), which can be initiated from multiple organelles: compounds that localise in the ER directly induce oxidative ER stress (Type II ICD inducers), whereas those that accumulate elsewhere can trigger ER stress indirectly as a collateral effect (Type I).^79–81^ In both cases, the resulting ER stress leads to the emission of damage-associated molecular patterns (DAMPs), principally calreticulin (CRT) exposure, ATP release, and HMGB1 secretion, which collectively stimulate anti-tumour immunity, leading to the potential for long-term protection against tumour relapse and metastasis.^82,83^ More specifically, photoactive metal complexes are promising ICD inducers because they can trigger ER stress through ROS generation.^84^ We therefore examined library-wide cellular localisation in HTS336 and combined it with potency data to select diverse complexes and explore their potential for inducing ICD upon photoexcitation.

**Fig. 5:**
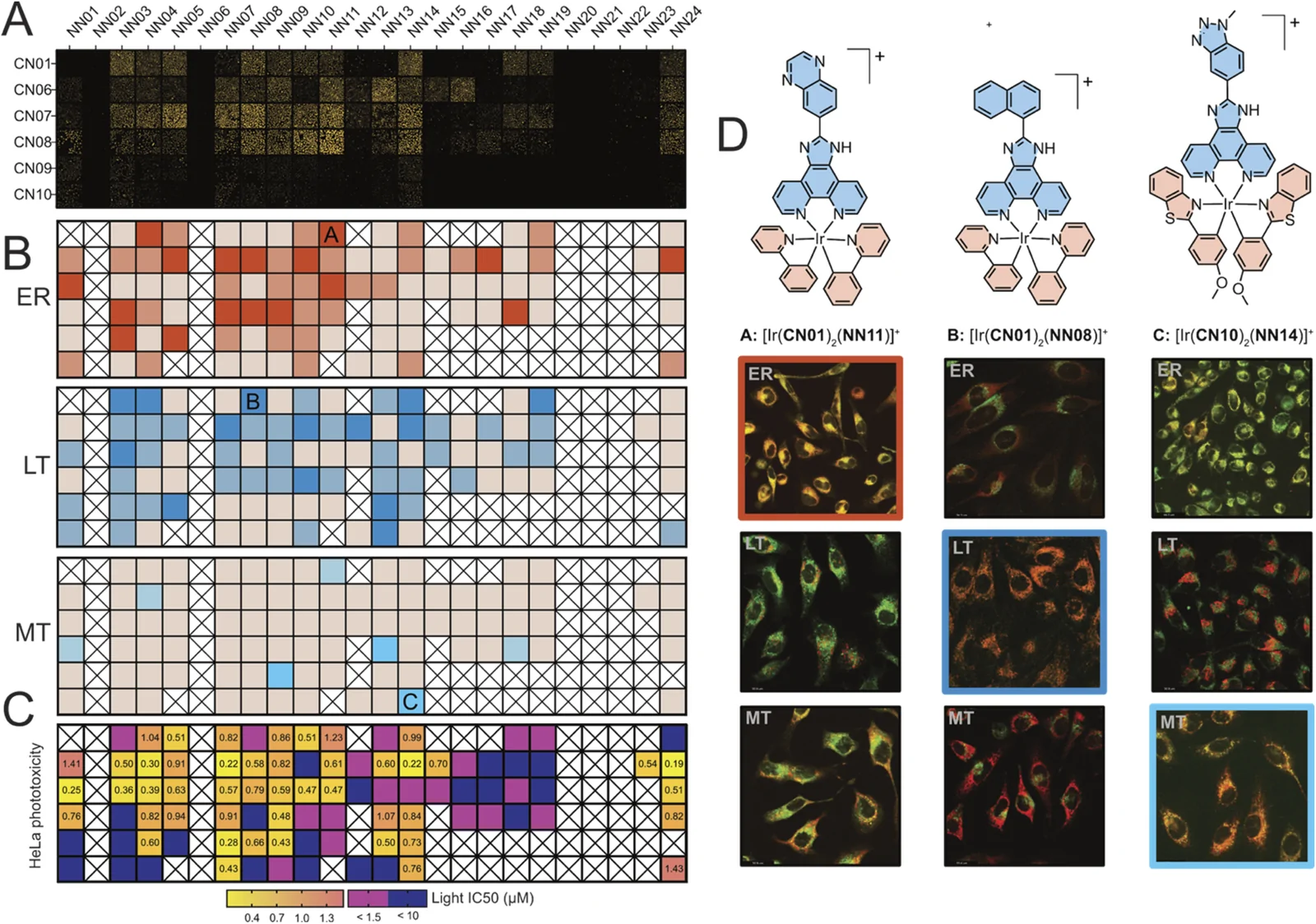
(A) Screening a subset of HTS336 for brightness in HeLa cells *via* microscopy (*λ*_ex_ = 470 nm/*λ*_em_ = 500–600 nm), (B) co-localisation scores with ER tracker (ER), LysoTracker (LT) and MitoTracker (MT), in which each compound was ranked for either none, partial, or good localisation (in which the light colour shade represents partial and dark represents good) and (C) photocytotoxicity scores. If a complex was not taken forward for co-localisation analysis due to low brightness, an X was placed through the cell; (D) structure and images of three representative examples with strong localisation in ER, lysosome and mitochondria respectively (Ir complex *λ*_ex_ = 488 nm/*λ*_em_ = 500–620 nm, and tracker dye *λ*_ex_ = 633 nm/*λ*_em_ = 650–750 nm).

A subset of the library was selected, based on CN ligands which generally showed very good photocytotoxicity and were structurally similar. This set of 144 complexes (with CN01 and CN06–10) were screened for a minimum brightness in HeLa cells which would allow for co-localisation studies (Fig. 5A). Those taken forward were treated separately with dyes that stain mitochondria (MitoTracker), lysosomes (LysoTracker) and endoplasmic reticulum (ER tracker), and images recorded by confocal microscopy. Compounds were manually scored by 0 (no channel overlap), 1 (reasonable) or 2 (excellent), with the results shown in Fig. 5 (and Fig. S11 for complete set of images). Three examples have been highlighted in Fig. 5D: A, [Ir(CN01)_2_(NN11)]^+^, which showed strong ER overlap and moderate MitoTracker overlap; B, [Ir(CN01)_2_(NN08)]^+^ which had excellent LysoTracker co-localisation and C, [Ir(CN10)_2_(NN14)]^+^ which primarily localised in the mitochondria. A truncated version of the photocytotoxicity scores against HeLa cells is also shown for reference. In all cases, a white square with an “X” through denotes a complex that was not taken forward for co-localisation experiments as the threshold brightness was not passed.

Library-wide localisation was heavily skewed towards the ER and lysosomes, with surprisingly few complexes showing mitochondrial localisation. Within the library, only three complexes showed strong co-localisation with MitoTracker: [Ir(CN08)_2_(NN13)]^+^, [Ir(CN09)_2_(NN09)]^+^ and [Ir(CN10)_2_(NN14)]^+^. In all cases, these were complexes with a benzimidazole/benzothiazole cyclometallating ligand. In several cases minor structural changes led to a large difference in cytotoxicity and co-localisation. For example, shifting the phenyl ring one position on the CN ligand ([Ir(CN06)_2_(NN01)]^+^ to [Ir(CN07)_2_(NN01)]^+^) led to higher ER localisation, and a 7-fold increase in photocytotoxicity. Similarly, [Ir(CN06)_2_(NN24)]^+^ also localised in ER, and displayed over two-fold higher phototoxic activity than complexes containing similar ligands.

However, higher ER localisation and increased toxicity are not universally observed; for example, within complexes containing the NN03 family of ligands, those with CN01/06/07 are highly toxic, and have better LysoTracker overlap, whereas those with CN08 and CN09 have strong ER overlap but lower toxicity.

Guided by the microscopy studies highlighted above, six iridium(iii) complexes with varying photocytotoxicity and subcellular localisation were selected (see Table 1). These complexes were re-synthesised and isolated on a preparative scale (see SI) and evaluated for their ability to induce ICD upon photoactivation in MCA205 fibrosarcoma cells. Firstly, IC_50_ values after light irradiation were determined for the six complexes and found to correlate well with those previously obtained in HeLa cells (see SI). We then proceeded to determine the three DAMPs for the six complexes following light irradiation. ATP release was monitored *via* a kinetic luminescence assay over 24 hours showing that all compounds were photoactive for these markers. Complexes [Ir(CN06)_2_(NN07)]^+^ and [Ir(CN07)_2_(NN11)]^+^ with ER localisation, triggered strong ATP release immediately after treatment (*i.e.* 4 h incubation in the dark followed by light irradiation) at concentrations >2.5 µM. The lysosome-localising [Ir(CN01)_2_(NN08)]^+^ displayed a similar profile, reaching delayed ATP maxima at 2.5 µM and 5 µM. In contrast, [Ir(CN01)_2_(NN03)]^+^ (also localised in lysosomes) showed a pronounced delay, and only reached a clear maximum at 10 µM. The ER/mitochondria-localizing [Ir(CN10)_2_(NN14)]^+^ was the only complex to trigger detectable ATP release already at 0.2 µM.

**Table 1:** Summary of lead complex biological data, including organelle localisation, IC_50_ data in MCA205 fibrosarcoma cells after light irradiation, and the three markers for ICD: ATP release, HMGB1 release, and CRT exposure

Complex	Primary organelle	IC50 (µM)	ATP rel. 3 h (µM)	ATP rel. 24 h (µM)	HMGB1 rel. (µM)	CRT exp. (%) at 1 µM	CRT exp. (%) tested conc
[Ir(CN01)_2_(NN03)]^+^	Lyso	0.35	10	0.5	1	1.6	3 (1.5 µM)
[Ir(CN01)_2_(NN08)]^+^	Lyso	0.37	2.5	0.5	1	7.3	7.3 (1 µM)
[Ir(CN06)_2_(NN07)]^+^	ER/Lyso	0.33	2.5	0.5	1	5.4	6.2 (1.5 µM)
[Ir(CN07)_2_(NN11)]^+^	ER	0.14	2.5	0.5	0.5	2.6	3 (0.75 µM)
[Ir(CN08)_2_(NN18)]^+^	ER/Mito	2.24	5	1	1	None	None (2 µM)
[Ir(CN10)_2_(NN14)]^+^	Mito/ER	0.15	2.5	0.2	1	5.3	5.4 (1 µM)

All complexes induced HMGB1 release at ≥1 µM after light irradiation, while [Ir(CN07)_2_(NN11)]^+^ also showed detectable levels at 0.5 µM. Next, CRT translocation to the plasma membrane was quantified after 24 h and compared at a fixed concentration of 1 µM and at adjusted concentrations chosen (after previously-performed cell death assays, see ESI) to yield comparable levels of cell death. All complexes induced CRT exposure – which is considered the most predictive marker for ICD. The ER-localising [Ir(CN07)_2_(NN11)]^+^ and the ER/mitochondria-localising [Ir(CN10)_2_(NN14)]^+^ displayed CRT exposure of 3–5.4% in viable cells at 30–60% cell death. [Ir(CN08)_2_(NN18)]^+^ did not induce significant CRT exposure at the tested treatment concentrations and light irradiation conditions, which correlates with its low photocytotoxicity (≤3% at 2 µM). The lysosomal complex [Ir(CN01)_2_(NN08)]^+^ showed the highest CRT-positive cell fraction (7.3%), while a second lysosome locating complex, [Ir(CN01)_2_(NN03)]^+^, also induced detectable CRT exposure (3%) despite limited photocytotoxicity. Notably, [Ir(CN06)_2_(NN07)]^+^ (ER/lysosomal localisation) and [Ir(CN01)_2_(NN08)]^+^ (lysosomal localisation) emerge as the most promising candidates, inducing high levels of CRT-positive live cells in the absence of significant cell death. Structurally, [Ir(CN06)_2_(NN07)]^+^ pairs a more hydrophilic, π-extended ligand in CN06 with an NN ligand with a polarisable amine; other complexes with a similar profile would be [Ir(CN06)_2_(NN24)]^+^, or [Ir(CN06)_2_(NN04)]^+^. However, these two complexes also exemplify how changing one ligand can have a large effect in cellular localisation; [Ir(CN06)_2_(NN04)]^+^ is one of the only complexes to show some degree of mitochondrial co-localisation. On the other hand, [Ir(CN01)_2_(NN08)]^+^ pairs a small CN ligand with a π-extended NN ligand, but reaches a very similar ICD profile, pointing towards the other similar NN ligands (Group II, Fig. 2) with this CN ligand.

### Machine learning models and virtual high-throughput screening

The experimental workflows described above have provided us with a large, internally consistent dataset of physicochemical (*i.e.* lipophilicity, photo-generation of ROS) and biological (*i.e.* light/dark cytotoxicity, cell permeability and cellular localisation) data. However, the volume and multidimensional nature of these data make direct interpretation challenging across multiple fronts: (i) balancing out the contributions to biological activity from uptake, ROS generation and sub-cellular localisation, amongst other properties, can be difficult; (ii) within a 300 metal complex library there are thousands of potential pairwise comparisons to make, and (iii) as shown above, a modular design approach can offer multiple pathways to reach similar endpoints. However, this is a problem ideally suited to machine-learning approaches, which we use here to rationalise the observed behaviour and to guide the design of new complexes with improved properties.

To construct a machine learning model, each iridium complex must first be represented to the model. This is carried out *via* featurisation, a process in which molecular structures are converted into quantitative descriptors that capture relevant aspects of the compounds' properties.

Many types of featurisation can be implemented; here we have first calculated ligand-level descriptors for each CN and NN ligand, which are then concatenated to represent a complex. This enables efficient scaling to large metal-complex datasets with only a few low-cost calculations on individual ligands rather than computing individual properties for each complex that are both larger in number and in size and electronic structure complexity. For both CN and NN ligands GFN2-xTB^85^ was used to calculate electronic properties, such as HOMO, LUMO, ground state energy, ionisation potential, electron affinity, coordinating atom partial charge, and condensed Fukui function on the coordinating atoms.^86^ The condensed Fukui function provides an indication of how much a molecule will localise an additional positive, radical or negative charge on the coordinating atoms. In total, this corresponds to 28 features per complex (see Table S1 for a list of all computational descriptors). In addition, further features were generated *in silico* by applying the same semiempirical tight-binding method directly to the complete complexes comprising HTS336 for the purpose of obtaining an estimate of log *P* (see SI Methods). Steric descriptors such as buried volume^87^ and solvent accessible surface area (SASA)^88,89^ of the complexes are also computed (see SI Methods).

We then employed a random forest model to predict experimental physicochemical properties, namely retention time, ABDA and DHR-123 scores. As in prior work, random forest models can offer interpretability through feature importance analysis based on impurity scores.^90^ To assess whether GFN2-xTB calculations were sufficient, we also computed electronic properties using higher-level DFT methods and found that these descriptors did not improve model performance (see Table S2 for all performance metrics for all subsequent ABDA, DHR-123, and retention time models).

First, with a 70 : 30 random train-test split, we aimed to predict experimental retention time using both ligand-level and complex-level descriptors. The model demonstrated excellent correlation between predicted and experimental values, achieving an average test-set *R*^2^ value of 0.84. As expected, the computational log *P* was correctly ranked by the model as the top predictive feature, while other complex-level descriptors contributed only marginally (Fig. S12). Even though a single feature dominated, the *R*^2^ of the model was significantly improved over the univariate, linear correlation between retention time and the computational log *P* (*R*^2^ = 0.601, Fig. S13).

**Fig. 6:**
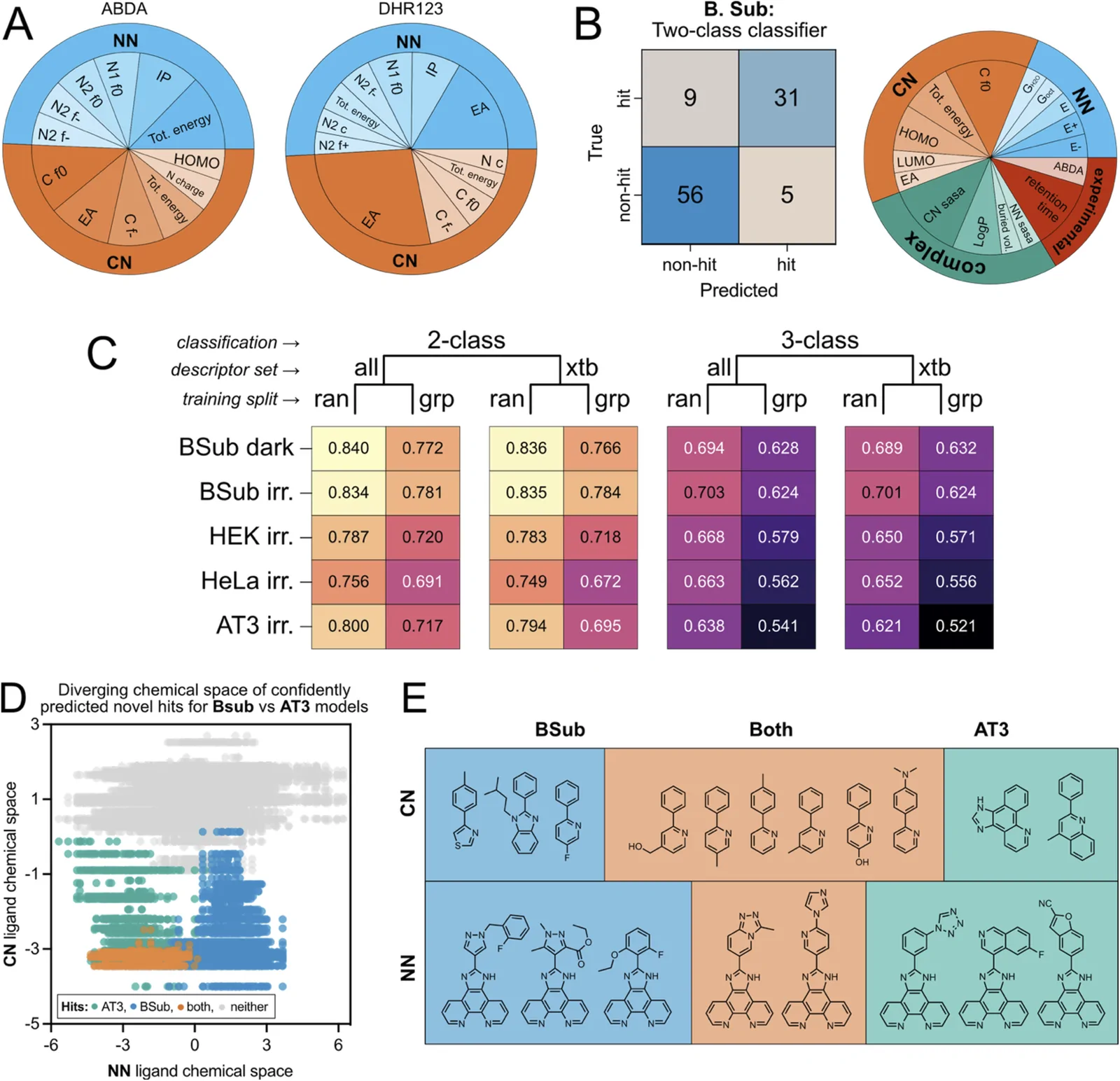
(A) Feature importance of ML models for ABDA and DHR-123; (B) ML model classifier showing example for the binary classification of activity in *B. subtilis* in the dark alongside the top sixteen ranked feature importance split by NN, CN, complex and experimental features; (C) testing model performance varying classification type, descriptor set and split type (all numbers as balanced accuracy); (D) confidently predicted novel hits in vHTS for *B. subtilis* and AT3, and (E) selection of enriched NN and CN fragments within *B. subtilis* and AT3 pools, or both.

We then trained models to predict ABDA scores and DHR scores with ligand-level descriptors only and achieved an average test-set *R*^2^ of 0.77 and 0.68 respectively, also with a 70 : 30 random train-test split. Feature importance analysis revealed an approximately 50 : 50 contribution from CN and NN ligands, indicating they play equally significant roles in determining ROS activity (Fig. 6A), even though previous work has shown that the CN ligand dominates many of the photophysical properties of the complex. To evaluate model transferability for ABDA, we employed a Leave-One-Group-Out (LOGO) train-test split, which provides a more rigorous assessment than a random split. In LOGO, one ligand is excluded from the training set and used only in the testing set. LOGO analysis of ABDA score regression revealed that excluding NN21 and NN06 from the training set resulted in substantially higher test-set mean absolute errors (Fig. S14). Both NN21 and NN06 belong to complexes exhibiting high ROS scores, and they are lipophilic. Accordingly, there might be some aggregation induced enhancement,^91^ which the ML model cannot directly consider. For complexes containing ligands in groups which were well represented in terms of shared molecular descriptors (such as NN12–17), model performance remained high. The same analysis was carried out for DHR-123, but no similar trend was observed (Fig. S15).

In comparison to the physicochemical parameters described above, the biological activity (*i.e.* light/dark cytotoxicity and cellular uptake/localisation) is potentially more difficult to rationalise/predict due to the complexity of the cellular environment. Therefore, in addition to the ligand-level and complex-level descriptors described earlier to predict the physicochemical properties, we included descriptors such as retention time, ABDA scores, and DHR-123 scores which were obtained experimentally. Because MIC and IC_50_ values were only determined for complexes exceeding the activity threshold, a classification task was more appropriate than regression. For example, for *B. subtilis*, a two-class classification can be used, in which complexes with MIC ≤ 16 µM are labelled as hits and those with an MIC > 16 µM non-hits. Training an RF classifier on this data using a 70 : 30 random train-test split produces a predicted *vs.* true matrix such as that in Fig. 6B (shown for *B. subtilis*, dark); in this case with an accuracy score of 0.85. Retention time, the condensed Fukui function on the carbon coordinating atom of CN ligand, and CN SASA are the most important features identified by the model.

This methodology was used to train models on *B. subtilis* (dark and light irradiated), and the three mammalian cell lines (all light irradiated). To explore the limits of the model, we also tested reformulating the models as a 3-class classification, using a random *versus* grouped split, and using xTB-features only (Fig. 6C, see Table S3 for model performance analysis). As expected, the average test accuracy decreased both when a 3-class classifier was used, and when grouped split was applied (representative confusion matrices shown in Fig. S16 and S17). The mammalian models perform worse than those applied to *B. subtilis*, presumably due to additional factors such as the effect of intracellular ROS generation and subcellular localisation not being captured within the current descriptor space. However, across all models, the use of an xTB-only descriptor set, made very little difference to accuracy, indicating that they alone successfully capture the structural diversity of the library. This has important consequences, as the low cost of generating ligand-only xTB features allows for virtual high-throughput screening of hundreds of thousands to millions of complexes. To this end, two independent RF binary classifiers were trained on the full HTS336 library to predict antibacterial activity against *B. subtilis* in the dark (MIC ≤ 16 µM classified as a hit) and cytotoxicity against AT3 cells under light irradiation (IC_50_ ≤ 1.5 µM classified as a hit), to make useful predictions about lead candidate ligands for future libraries and to conduct SAR analysis to investigate if there were areas of diverging chemical space between complexes most likely to be hits for bacterial applications compared with cancer cells.

To build the enlarged virtual library, a list of 187 CN ligands was obtained through scraping of MetalCytoToxDB, a recently published dataset of biologically active metal complexes.^92^ As the rationale behind picking the phenanthroline-imidazole scaffold was that it provides a synthetically accessible route to a large and electronically diverse NN-ligand space through a one-step condensation, we built our own set of 1000 NN ligands based on commercially available, drug-like aldehydes from the enamine catalogue. These ligands were then combined with those from HTS336 to give a total library of >200 000 virtual complexes (termed vHTS), 9% of which contained one novel ligand and one from HTS336, and the remaining 91% of which were completely novel. To ensure that accurate and in-scope predictions were made, an applicability domain (AD) filter was applied, followed by uncertainty cutoffs (UQ) determined using normalised Shannon entropy (see Fig. S18 for PCA analysis of the vHTS chemical space and effects of filtering). The effect of these cutoffs on prediction performance was assessed by cross-validation on HTS336, using a grouped split (*i.e.* withholding NN and CN ligands) to mimic the novelty of the virtual library. Restricting to predictions below the entropy cutoff raised balanced accuracy from 0.75 to 0.88 for *B. subtilis* and from 0.70 to 0.83 for AT3, with hit precision of 0.85 and 0.86 respectively (see Fig. S19 for confusion matrices before and after). This led to a total of 16 665 and 4010 novel vHTS complexes for *B subtilis* and AT3 respectively, of which 3790 (23%) and 2143 (53%) were predicted active, with 576 predicted active in both assays. When these hits are plotted, a clear divergence in hit chemical space for *B subtilis* and AT3 can be observed, with the NN and CN axes having markedly different effects both on whether a given complex is a hit at all and secondly which target it is more likely to favour (Fig. 6D).

Ligand-level enrichment was carried out by ranking and shortlisting ligands based on the Wilson score lower bound of their hit rate and filtering by cross-target predicted activity to enforce selectivity. This revealed a clear division between the two ligand types (representative examples shown in Fig. 6E). As the two models are filtered at different uncertainty thresholds to keep only high-quality predictions, the screen-able pools differ in size and composition (16 665 complexes from 926 NN and 90 CN ligands for *B subtilis*, *versus* 4010 from 463 NN and 56 CN for AT3). A fragment absent from one target's filtered pool is therefore treated as not predicted-active in that target, which reflects an absence of confident predictions rather than confident inactivity; selectivity assignments should be read as enrichment within each model's confident domain rather than as quantitative target discrimination. The CN ligands show near-identical enrichment profiles across both assays: 26 CN fragments achieve 100% hit rates across tens to hundreds of complexes in both the *B. subtilis* and AT3 pools (a selection is shown in Fig. 6E). Their activity appears target-agnostic; inclusion of one of these CN ligands is effectively sufficient for predicted activity regardless of the NN-ligand partner or biological target. These ligands share various xTB features; they are electron-rich with high-lying frontier orbitals and enhanced electron density at the coordinating carbon. The number of CN ligands specific to either *B. subtilis* or AT3 was much smaller but did diverge in structure. Overall, it is also clear that analogues of experimentally active π-extended CN ligands (such as CN06 and CN07) are not identified by the model. Presumably, this is because three-ring CN systems predominantly produce higher-uncertainty predictions and are removed by the UQ filter, as further π-extension into four-ring and larger systems leads to complexes that pass the UQ filter but are confidently predicted as inactive. Future libraries should therefore deprioritise four-ring CN scaffolds in favour of targeted three-ring variants, where expanded training data could resolve the current ambiguity.

In contrast, the NN ligand preferences diverge between the two model predictions, with negligible overlap between the top-ranked NN ligands for *B. subtilis* and AT3. The *B. subtilis*-enriched NN ligands show hit rates of 73–83% against the 22.7% background, while a distinct set of AT3-enriched NN ligands achieve hit rates of 100% against the 53.4% background. Inspection of the top-ranked xTB features distinguishing the two shortlists reveals that AT3-selective NN ligands are markedly more π-electron-deficient; their mean LUMO energy is 0.69 eV lower (−8.13 eV *versus* −7.44 eV for *B subtilis*-selective ligands, against an all-NN baseline of −7.75 eV), their electron affinity is 0.52 eV higher (2.09 *versus* 1.57 eV), and their HOMO–LUMO gap is substantially narrower (1.64 *versus* 2.06 eV, baseline 1.90 eV). This electronic profile is chemically consistent with the irradiated AT3 assay conditions. As the LUMO in cationic Ir(iii) complexes of this type is localised predominantly on the NN ligand,^19^ the NN ligand LUMO energy is a direct determinant of the complex HOMO–LUMO gap. A smaller HOMO–LUMO gap facilitates intersystem crossing to the long-lived excited state, and subsequent ROS generation (although as discussed earlier, this is only one part of an overall complex picture of effective PDT in cells).

By contrast, the *B. subtilis*-selective NN ligands are more electron-rich at the coordinating nitrogen atoms, exhibiting higher electrophilic Fukui coefficients at both donor nitrogen atoms (*f*^+^ = 0.050 *versus* 0.041), lower ionisation potentials (7.78 *versus* 8.08 eV), and higher-lying HOMOs (−9.49 *versus* −9.76 eV). The dark antibacterial mechanism does not rely on photochemical activation, and this different electronic character at the metal-binding site could translate to altered charge distribution at the iridium centre and modified interactions with bacterial targets such as membranes.

These electronic trends align directly with the top-ranked NN-ligand features in each model: in the AT3 model, the NN HOMO–LUMO gap and ionisation potential rank third and fourth among all features, reflecting the primacy of frontier orbital energetics in the photocatalytic mechanism; in the *B. subtilis* model, the activation energy (third) and neutral and electrophilic Fukui coefficients at the coordinating nitrogen atoms (ranked fourth and fifth) are the most discriminating NN descriptors. Notably, both models share identical top-ranked CN features (the carbon neutral Fukui coefficient and total energy), consistent with the target-agnostic dominance of the CN ligand observed at the ligand-enrichment level. Together, this provides a practical roadmap to design new complexes. CN-ligand choice governs whether a complex enters the active chemical space at all, while NN-ligand choice determines which target it is selective for. These findings provide concrete synthetic priorities for the next generation of complexes: deprioritise large π-extended CN scaffolds, focus CN exploration on targeted three-ring variants and small electron-rich scaffolds, and use the divergent NN selectivity profiles to direct synthesis toward either antibacterial or anticancer leads or, where dual activity is desired, toward the overlapping predicted-active space that exploits both.

## Conclusions

The automated and high-throughput platform presented here provides a systematic approach to synthesise and characterise large number of photoactive iridium(iii) complexes. Taking a direct-to-biology (D2B) approach, we screened 336 complexes against three mammalian cell lines (two cancer, one non-cancer) and two bacterial strains and selected a 144-member subset for detailed cellular imaging studies to determine their cellular uptake and localisation. From these studies, we identified six complexes with distinct subcellular localisation for in-depth studies of immunogenic cell death. While all six complexes caused characteristic DAMPs upon light activation, subtle structural modifications leading to altered subcellular localisation resulted in distinct DAMP profiles, suggesting that organelle targeting influences the kinetics and quality of ICD induction. Among the investigated compounds, [Ir(CN06)_2_(NN07)]^+^ appears to be the most promising ICD lead, as it combines low-dose cytotoxicity with early CRT exposure and robust ATP and HMGB1 release.

Working with this consistent, multi-dimensional dataset enabled machine learning analysis that goes beyond rationalising the observed behaviour. Using inexpensive xTB-level descriptors alone, random forest models predict both physicochemical and biological responses with good accuracy, while feature importance analysis reveals that ROS metrics play a surprisingly minor role. The models perform best for broadly cytotoxic behaviour and are less accurate for strictly phototoxic responses, underscoring the added complexity of light-activated mechanisms in mammalian cells. Virtual screening of a >200 000 complex library with an ML model using quantified uncertainty, built using the modular design principles of the NN ligand, identified thousands of novel predicted-active complexes. It revealed a clear division in ligand preference between antibacterial and anticancer activity; CN-ligand identity governs entry into the active chemical space largely irrespective of target, while NN-ligand electronic character determines target preference. These findings provide concrete and experimentally actionable design rules for the next generation of complexes.

## Author contributions

T. K. and R. V. designed the overall project, planned experiments and analysed the data. T. K. performed the automated synthesis, biophysical characterisation and high throughput cellular experimentation. Y. W., G. T., T. K. and H. J. K. designed and performed the computational and machine learning studies. A. M. recorded confocal microscopy images and analysed the data. A. M.-H. Y., J. S. and K. K.-W. L. designed and synthesised some of the iridium complexes. J. S., Y. A. and N. M.-N. performed cellular experiments to determine mechanism of cell death. B. V. H. supported the cellular high-throughput experiments. T. K. and R. V. wrote the manuscript. All authors contributed to editing the manuscript. K. K.-W. L., N. M.-N., H. J. K. and R. V. secured funding for the project.

## Conflicts of interest

There are no conflicts to declare.

## Supplementary Material

SC-OLF-D6SC04917F-s001

SC-OLF-D6SC04917F-s002

## Data Availability

All data supporting this research are provided as part of the supplementary information (SI) available online. Supplementary information is available. See DOI: https://doi.org/10.1039/d6sc04917f.
