# Supplementary material for: Exploring chemical space for iridium(iii) complexes: a direct-to-biology (D2B) approach to identifying anticancer and antibacterial agents
Source: Chem Sci. 2026 Aug 21;17(40):19469–82. doi: 10.1039/d6sc04917f (PMC13531521; doi:10.1039/d6sc04917f)
Supplement: Supplementary file 1 [file SC-017-D6SC04917F-s001.pdf]

Supplementary Information (SI) for Chemical Science.
This journal is © The Royal Society of Chemistry 2026

Supplementary Information

Exploring Chemical Space for Iridium(III) Complexes: a Direct-to-Biology (D2B) Approach to Identifying Anticancer and Antibacterial Agents

Timothy Kench,[1*] Yiran Wang,[2] Jana Seefeldt,[3] Aatikah Majid,[1] Alex Man-Hei Yip,[4] Justin Shum,[4] Gianmarco G. Terrones,[2] Yassin Antar,[3] Benedikt V. Holbling,[5] Kenneth Kam-Wing Lo,[4] Nils Metzler-Nolte,[3] Heather J. Kulik,[2,6*] Ramon Vilar[1*]

[1] Department of Chemistry, Imperial College London, White City Campus, W12 0BZ London, UK
[2] Department of Chemical Engineering, Massachusetts Institute of Technology, 02139 Cambridge, MA, USA
[3] Faculty of Chemistry and Biochemistry, Inorganic Chemistry I – Bioinorganic Chemistry, Ruhr University Bochum, Universitätsstrasse 150, 44801 Bochum, Germany
[4] Department of Chemistry, City University of Hong Kong, Tat Chee Avenue, Hong Kong, P. R. China
[5] Drug Discovery Hub, Translation & Innovation Hub Building, Imperial College London, White City Campus, W12 0BZ London, UK
[6] Department of Chemistry, Massachusetts Institute of Technology, 02139 Cambridge, MA, USA

Contents

Contents	Page
Main ESI figure	2
Experimental Methods	18
Computational Methods	25
Synthesis and Characterisation	30
Additional ESI figures	43

Main Supplementary Figures

	NN01	NN02	NN03	NN04	NN05	NN06	NN07	NN08	NN09	NN10	NN11	NN12	NN13	NN14	NN15	NN16	NN17	NN18	NN19	NN20	NN21	NN22	NN23	NN24
CN01	78	81	76	90	75	77	85	83	94	83	92	96	93	89	96	95	90	98	91	88	93	55	97	96
CN02	86	89	91	83	82	84	80	87	90	91	94	87	92	89	85	90	87	89	88	87	81	63	94	94
CN03	93	74	90	69	92	91	50	93	93	94	84	92	96	93	93	94	92	88	92	91	92	68	90	97
CN04	94	96	97	86	91	96	68	96	94	94	86	97	97	91	98	97	93	90	94	95	94	90	91	97
CN05	87	78	90	71	87	88	65	90	86	88	76	86	89	87	93	93	88	88	89	87	85	61	88	89
CN06	95	97	95	92	91	95	90	95	88	93	93	95	94	93	94	92	95	97	95	95	95	71	95	88
CN07	96	90	94	95	93	95	95	98	96	95	97	99	95	91	97	95	96	96	99	95	95	94	93	95
CN08	95	96	94	90	92	95	85	96	94	100	96	96	95	90	96	98	98	98	96	98	96	61	96	94
CN09	90	95	95	87	91	85	85	93	93	93	97	89	91	86	89	96	94	95	92	90	90	40	88	90
CN10	91	96	97	86	92	95	85	94	94	95	98	90	90	87	94	97	94	98	93	93	90	34	88	90
CN11	86	80	87	85	87	93	56	87	90	87	88	93	88	89	89	92	88	89	86	84	82	48	84	84
CN12	91	86	93	62	90	95	71	90	90	89	86	91	89	91	91	93	89	90	88	89	86	60	84	86
CN13	83	85	82	68	77	72	75	89	69	87	82	75	76	81	86	86	87	85	83	77	77	33	86	81
CN14	96	88	91	85	88	90	75	89	87	93	92	88	91	96	94	97	88	93	95	95	92	62	89	87

Figure S1: Reaction yields. Conversion percentage data values extracted from LCMS experiments (5-95% gradient of MeCN in H_2O with 0.1% formic acid). Please note, for those with **NN07** the integrations were manually assigned as two peaks in the LCMS trace with the same mass were observed.

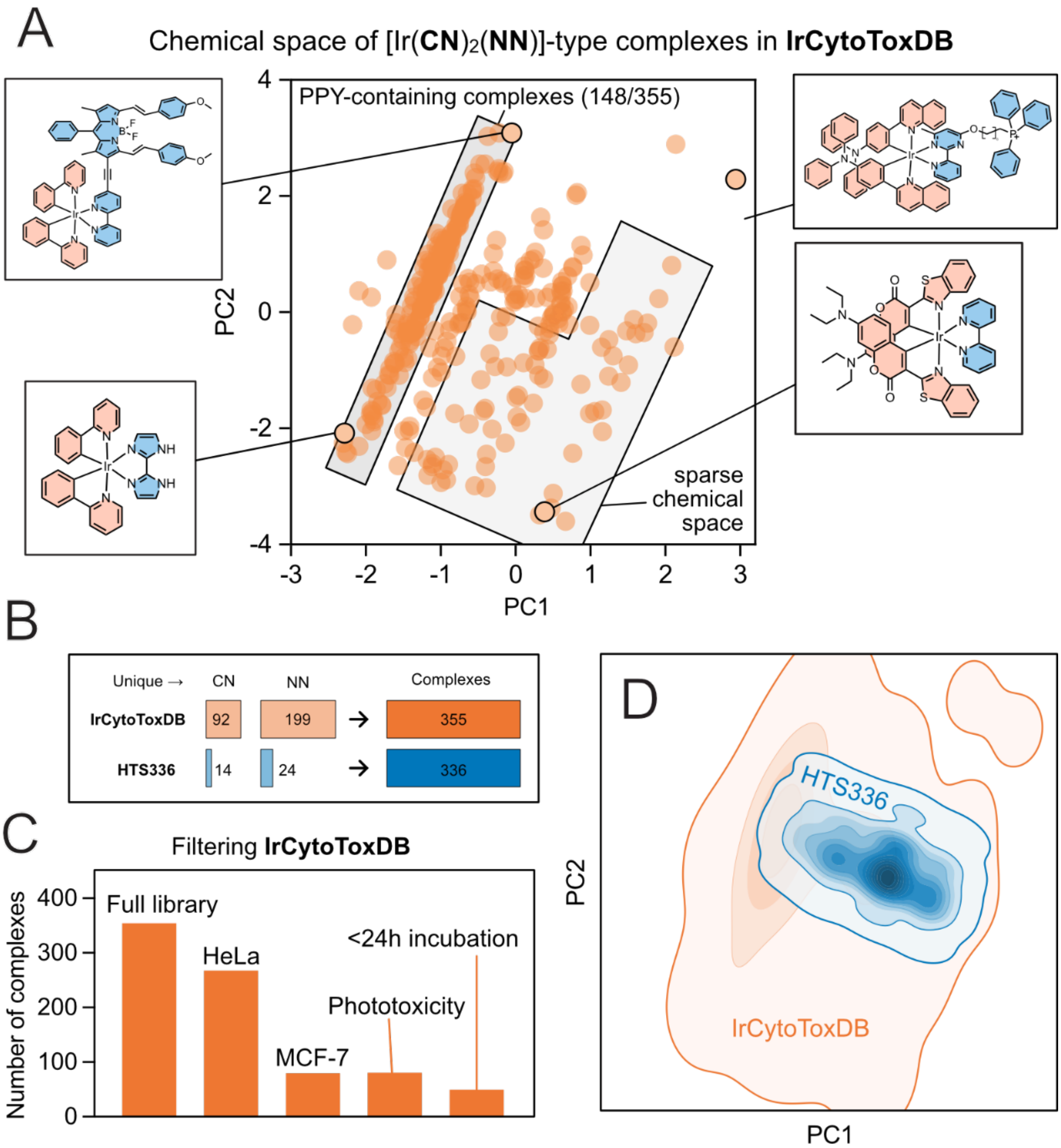

Figure S2: (A) Chemical space of 355 [Ir(**CN**)$_2$(**NN**)]$^+$-type complexes reported in **IrCytoToxDB**. PC1 captures **CN** ligand complexity, whilst PC2 captures **NN** ligand complexity. Four examples are highlighted, indicating the extremes of each combination of low and high complexity. (B) comparison of number of fragments needed to synthesis similarly sized libraries via tradition and HTP means, (C) filtering **IrCytoToxDB** to obtain more consistent biological testing conditions, showing the significant reduction in dataset size and (D) comparison of chemical space of **IrCytoToxDB** and **HTS336**.

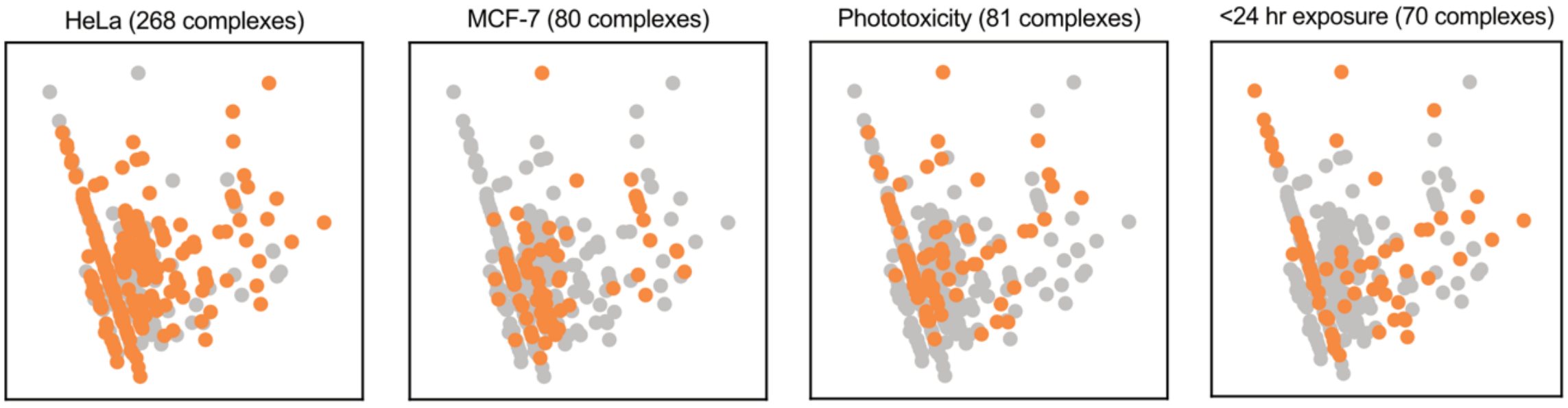

Figure S3: filtering **IrCytoToxDB** by various parameters, showing significant reduction in dataset size.

	NN01	NN02	NN03	NN04	NN05	NN06	NN07	NN08	NN09	NN10	NN11	NN12	NN13	NN14	NN15	NN16	NN17	NN18	NN19	NN20	NN21	NN22	NN23	NN24
CN01	1.01	0.27	0.81	1.18	1.13	2.13	1.31	1.79	0.46	1.05	1.00	1.32	1.66	0.99	0.50	0.65	0.66	1.26	1.89	0.40	0.79	0.81	0.15	0.90
CN02	0.37	0.24	0.42	0.59	0.80	1.46	0.61	0.60	0.25	0.74	0.59	1.32	1.60	0.53	0.40	0.54	0.38	1.11	0.78	0.38	0.59	0.39	0.01	0.51
CN03	0.93	0.49	1.06	0.68	1.41	2.28	1.28	1.47	0.81	1.34	1.10	1.27	1.76	1.41	0.74	1.02	0.96	1.74	2.11	1.42	0.90	0.62	0.22	0.98
CN04	1.09	0.45	1.63	0.91	1.31	2.18	1.95	1.52	1.24	1.25	1.20	1.81	2.01	1.62	0.76	1.11	0.86	1.78	1.59	0.50	0.88	1.07	0.20	1.35
CN05	1.32	0.69	1.97	1.35	2.05	2.31	2.57	1.76	1.32	1.68	1.53	2.19	2.17	1.78	1.00	1.27	0.91	1.69	1.69	0.70	0.90	1.97	0.34	1.85
CN06	0.84	0.53	1.58	1.80	1.74	2.41	1.88	1.62	0.89	1.20	1.54	1.59	1.72	1.46	1.17	1.27	0.95	1.88	2.15	2.45	1.08		0.53	2.19
CN07	1.26	0.63	2.34	1.58	1.73	2.58	2.55	1.66	1.33	1.50	1.70	1.93	2.54	1.86	1.37	1.30	1.16	1.63	1.70	0.95	1.11	1.17	0.06	2.95
CN08	0.87	0.24	1.54	1.26	1.82	1.41	2.03	1.41	1.78	1.09	1.64	1.68	1.50	1.20	0.83	0.92	0.57	1.21	0.99	0.55	0.65	1.07	0.35	1.37
CN09	0.71	0.19	1.06	1.04	1.31	2.07	1.76	1.21	0.94	1.00	1.05	1.46	1.75	1.13	0.79	0.86	0.70	1.11	0.91	0.56	0.92	1.41	0.27	1.95
CN10	0.82	0.17	0.99	0.89	1.15	1.82	1.59	1.15	1.15	0.85	1.03	1.20	1.44	1.10	0.83	0.83	0.47	0.85	0.81	0.45	0.84	0.98	0.24	1.49
CN11	1.49	0.96	1.91	1.86	2.05	2.81	3.09	2.25	2.07	1.64	2.22	2.54	2.55	2.48	1.63	2.00	1.26	1.96	2.03	1.08	1.12	1.90	0.81	4.00
CN12	1.80	1.04	1.96	1.65	2.15	2.99	2.82	2.45	2.13	1.67	2.17	2.54	2.22	2.69	1.43	1.82	1.33	1.82	2.19	1.37	1.41	2.18	0.85	3.08
CN13	0.59	0.25	0.69	1.18	0.85	1.06	1.00	0.94	1.07	0.87	0.66	1.26	1.05	0.95	0.62	1.09	0.39	0.73	0.72	0.51	0.76	1.20	0.45	0.85
CN14	0.86	1.26	1.10	1.49	1.17	1.96	1.71	1.32	1.23	0.78	0.86	1.49	0.94	1.28	1.01	1.62	1.37	1.04	1.39	1.41	1.60	2.51	1.73	2.11

Figure S4: Raw data values for retention time peak maxima data extracted from LCMS experiments (5-95% gradient of MeCN in H_2O with 0.1% formic acid). The higher the retention time – i.e. the blue end of the heat map – the more lipophilic the compound is.

	NN01	NN02	NN03	NN04	NN05	NN06	NN07	NN08	NN09	NN10	NN11	NN12	NN13	NN14	NN15	NN16	NN17	NN18	NN19	NN20	NN21	NN22	NN23	NN24
CN01	3.35	3.27	3.17	2.91	3.25	3.60	3.20	3.38	3.08	3.33	3.10	2.95	3.09	3.13	3.20	3.40	3.25	3.32	3.25	3.33	3.58	3.01	2.97	3.09
CN02	3.45	3.39	3.29	3.01	3.33	3.69	3.21	3.49	3.18	3.43	3.23	3.10	3.22	3.23	3.31	3.51	3.33	3.45	3.36	3.49	3.67	3.14	3.14	3.24
CN03	2.99	2.73	2.84	2.55	2.91	3.25	2.84	2.97	2.67	3.01	2.78	2.59	2.75	2.77	2.90	2.97	2.92	3.00	2.95	3.03	3.24	2.62	2.67	2.66
CN04	3.09	2.85	2.95	2.65	3.02	3.35	2.94	3.07	2.79	3.11	2.89	2.70	2.86	2.88	3.01	3.07	3.03	3.10	3.05	3.15	3.34	2.74	2.78	2.77
CN05	3.51	3.25	3.33	3.01	3.39	3.68	3.33	3.45	3.32	3.48	3.30	3.11	3.25	3.27	3.38	3.44	3.38	3.48	3.42	3.49	3.68	3.14	3.18	3.16
CN06	3.84	3.58	3.42	3.11	3.54	4.01	3.40	3.71	3.54	3.72	3.37	3.20	3.35	3.36	3.54	3.51	3.59	3.66	3.55	3.77	3.97	3.24	3.28	3.18
CN07	3.49	3.41	3.28	3.03	3.40	3.80	3.33	3.37	3.21	3.52	3.23	3.06	3.19	3.29	3.41	3.39	3.36	3.51	3.41	3.52	3.77	3.14	3.23	3.10
CN08	3.50	3.43	3.36	3.04	3.42	3.94	3.44	3.48	3.17	3.56	3.30	3.05	3.21	3.32	3.52	3.46	3.33	3.51	3.48	3.56	3.91	3.17	3.22	3.15
CN09	3.61	3.61	3.56	3.12	3.62	4.30	3.57	4.13	3.32	3.70	3.51	3.19	3.40	3.44	3.70	4.23	3.48	3.65	3.73	3.72	4.15	3.32	3.38	3.76
CN10	3.63	3.58	3.44	3.09	3.62	4.29	3.53	3.78	3.35	3.73	3.45	3.14	3.35	3.48	3.55	3.85	3.51	3.62	3.69	3.69	4.11	3.31	3.29	3.49
CN11	3.69	3.47	3.59	3.25	3.66	4.04	3.59	3.70	3.48	3.73	3.57	3.36	3.50	3.52	3.68	3.72	3.57	3.71	3.70	3.80	4.06	3.39	3.42	3.39
CN12	3.65	3.39	3.52	3.18	3.60	3.95	3.52	3.64	3.40	3.66	3.50	3.27	3.42	3.43	3.59	3.63	3.51	3.64	3.63	3.73	3.95	3.30	3.35	3.31
CN13	3.44	3.46	3.40	3.09	3.46	3.98	3.14	3.51	3.25	3.55	3.37	3.13	3.30	3.39	3.67	3.58	3.38	3.53	3.53	3.52	3.89	3.30	3.35	3.30
CN14	3.41	3.39	3.30	2.97	3.36	3.64	2.87	3.40	3.19	3.45	3.26	3.05	3.20	3.23	3.36	3.39	3.34	3.43	3.39	3.47	3.65	3.10	3.12	3.12

Figure S5: Averaged calculated ROS scores relative to $[Ru(bpy_3)]^{2+}$ for DHR-123. The higher the score – i.e. blue end of the heat map – the higher the ROS production.

	NN01	NN02	NN03	NN04	NN05	NN06	NN07	NN08	NN09	NN10	NN11	NN12	NN13	NN14	NN15	NN16	NN17	NN18	NN19	NN20	NN21	NN22	NN23	NN24
CN01	0.46	0.77	0.59	0.66	0.49	1.83	0.69	0.42	0.33	0.48	0.63	0.78	0.65	0.81	0.66	0.49	0.43	0.63	1.34	0.99	1.71	0.35	0.40	0.60
CN02	0.16	0.41	0.27	0.52	0.41	1.20	0.38	0.22	0.21	0.31	0.40	0.53	0.51	0.34	0.47	0.41	0.37	0.27	0.56	0.78	1.56	0.23	0.05	0.34
CN03	0.64	0.83	0.70	0.90	0.98	1.97	0.87	0.54	0.73	0.89	0.75	1.09	0.97	0.88	0.82	0.64	0.74	0.89	1.06	1.06	2.26	0.51	0.53	0.89
CN04	0.65	0.94	0.75	0.77	0.83	2.23	0.96	0.42	0.63	0.87	0.76	0.83	1.06	1.02	0.82	0.56	0.65	0.76	1.18	0.98	2.00	0.54	0.52	0.69
CN05	0.81	1.14	1.03	0.85	1.13	2.19	1.29	0.48	0.72	0.93	1.08	0.96	1.10	1.16	0.89	0.65	0.79	0.63	1.03	1.11	1.93	0.57	0.56	0.77
CN06	1.01	1.24	0.54	1.61	1.19	1.53	1.45	0.90	0.78	0.93	1.25	1.49	1.38	1.38	1.06	0.80	0.76	1.07	1.37	1.50	2.13	0.68	0.75	0.95
CN07	0.82	1.38	0.99	1.42	1.24	2.24	1.57	0.84	0.75	1.27	1.12	1.46	1.70	1.24	0.84	0.72	0.57	1.21	1.08	1.41	1.99	0.70	0.58	1.03
CN08	0.37	0.76	0.20	0.50	0.40	1.23	0.64	0.31	0.36	0.68	1.19	0.83	0.70	0.65	0.84	0.54	0.42	0.70	0.85	1.04	1.58	0.44	0.37	0.79
CN09	0.50	0.74	0.73	0.80	0.87	1.66	0.82	0.56	0.59	0.54	0.72	0.87	0.87	0.55	0.60	0.65	0.50	0.47	0.55	0.95	1.93	0.40	0.37	0.69
CN10	0.49	0.75	0.52	0.75	0.63	1.33	0.70	0.53	0.01	0.52	1.00	0.72	0.78	0.54	0.70	0.50	0.43	0.48	0.82	0.92	1.70	0.27	0.48	0.61
CN11	1.31	1.80	1.04	1.45	1.66	3.01	1.77	1.11	0.63	1.40	1.60	1.49	1.84	1.67	1.09	1.00	0.67	1.38	1.71	1.18	2.13	0.99	0.86	1.50
CN12	1.27	1.54	0.75	1.55	1.60	1.17	1.50	0.21	1.18	1.55	1.52	1.58	2.04	1.85	1.26	1.06	0.91	1.58	1.64	1.93	2.89	0.96	0.82	1.48
CN13	0.71	0.54	0.91	1.01	1.11	1.17	1.12	0.98	1.00	0.88	0.90	1.41	1.10	0.98	0.78	1.07	0.44	0.80	1.13	1.06	1.73	0.61	0.70	1.00
CN14	0.70	0.16	1.21	0.78	0.70	0.94	1.20	0.67	0.62	0.62	0.62	1.03	0.89	0.93	0.46	0.54	0.30	0.59	0.69	0.53	0.53	1.00	0.41	0.99

Figure S6: Averaged calculated ROS scores relative to $[Ru(bpy_3)]^{2+}$ for ABDA. The higher the score – i.e. blue end of the heat map – the higher the ROS production.

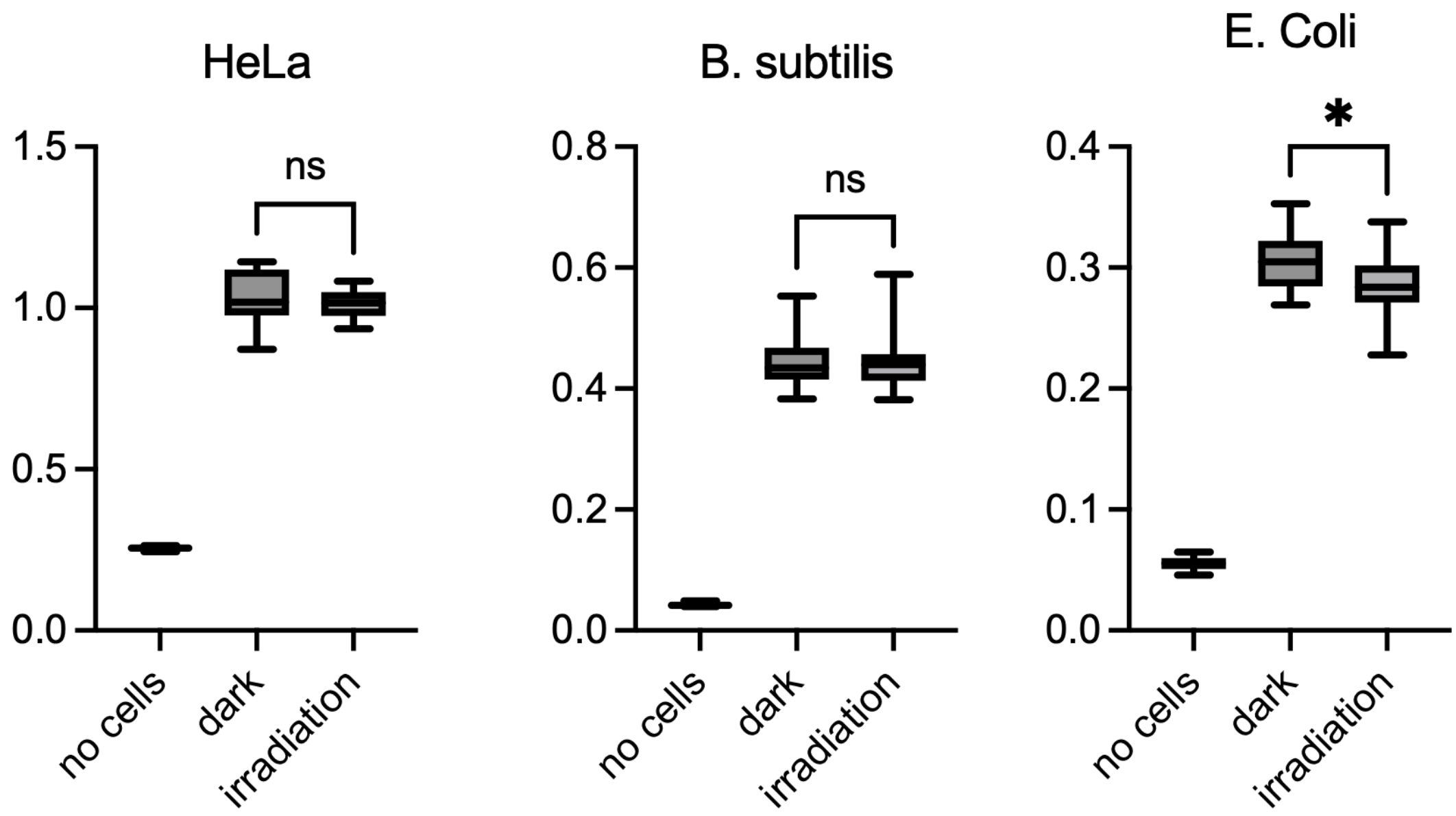

Figure S7: Control experiment to assess the effect of light irradiation on cells/bacteria in the absence of iridium complexes. Plots showing raw absorbance data in the y-axis for no cells, cells in the dark, and cells after irradiation; for HeLa the MTS assay readout was at 490 nm, and for *B. subtilis* or *E. coli*, OD readout at 600 nm.

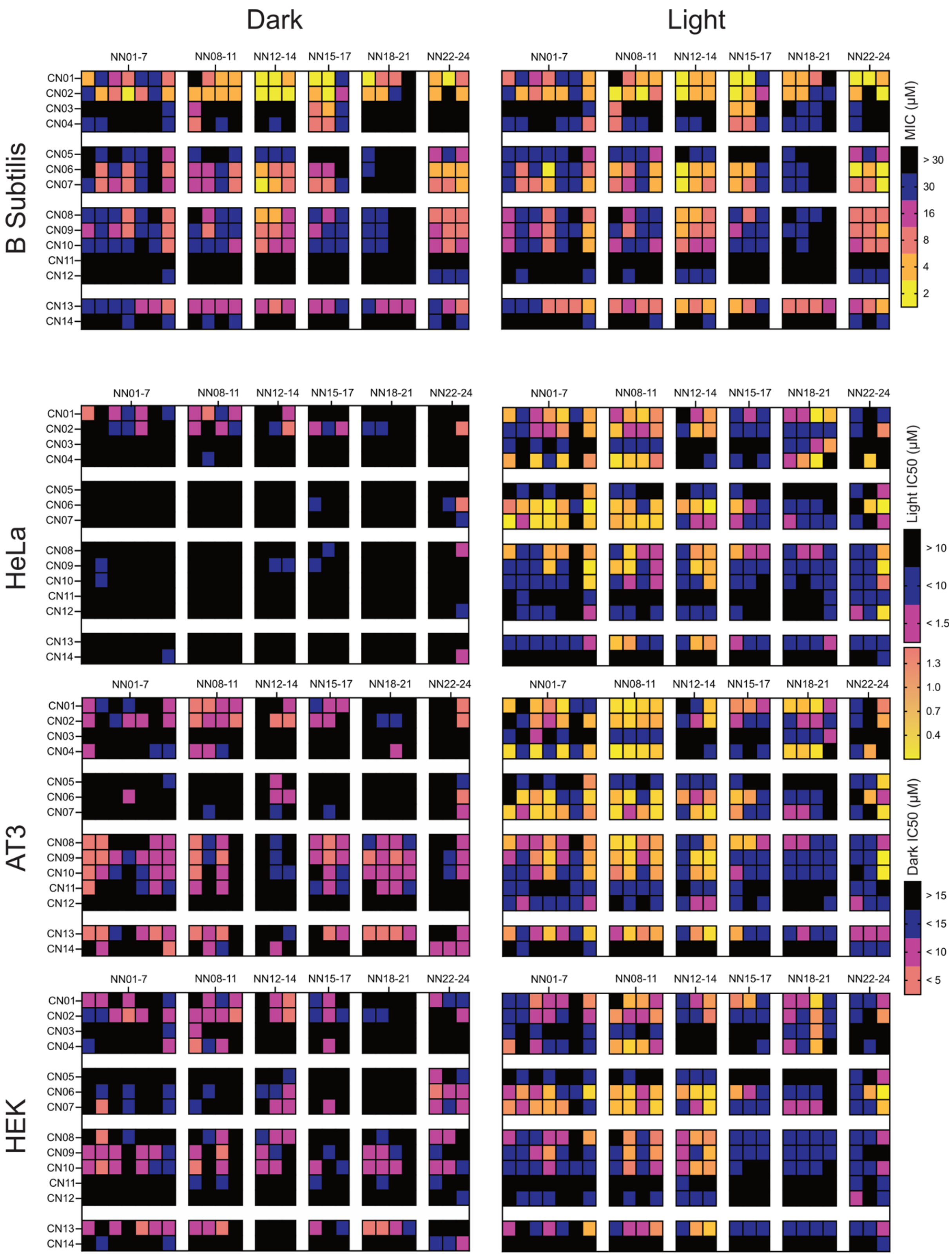

Figure S8: Screening results (shown as heat maps) **HTS336** against *B. subtilis*, HeLa, AT3 and HEK cell lines in the dark and after light irradiation (450 nm, power: 20%, 10 min, 1.2 J/cm^2).

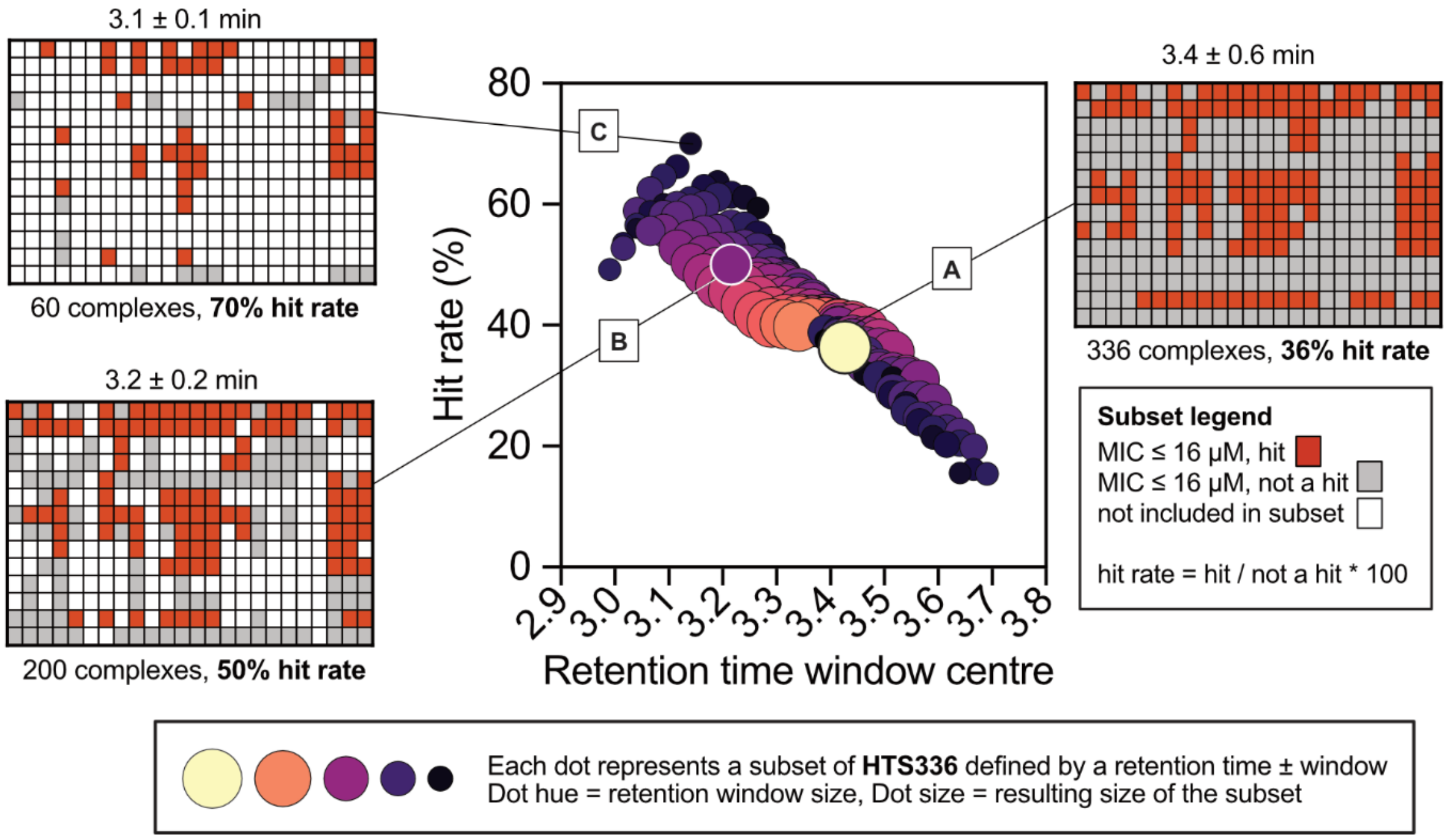

Figure S9: Scanning across different retention times to maximise hit rates in *B. Subtilis*. Each circle represents a sample of **HTS336**, defined by a window size starting at a specific retention time (e.g. 3.2 +/- 0.2). A hit is defined as any compound with an MIC ≤ 16 µM. Minimum sample size in a subset is 50 compounds.

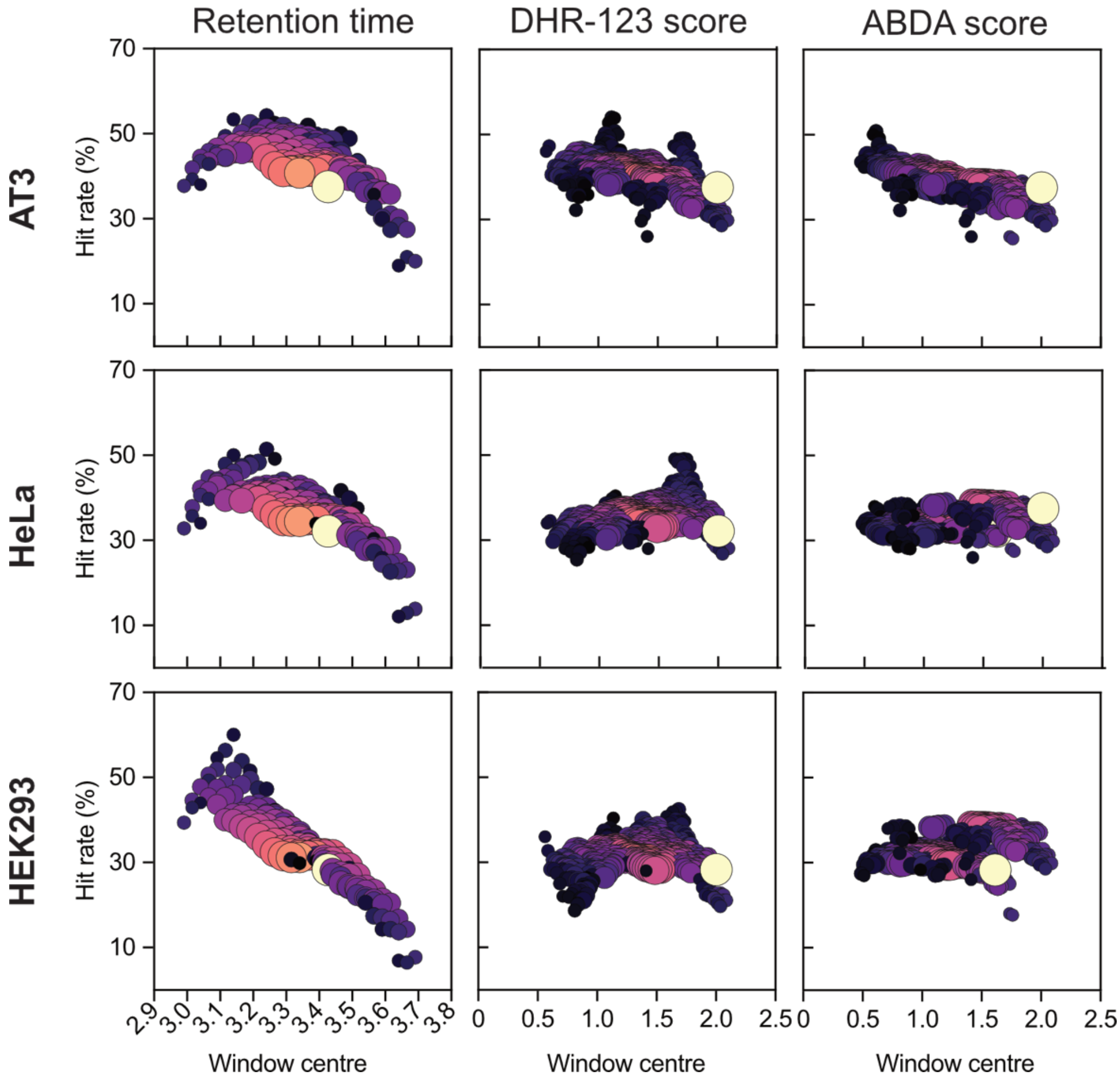

Figure S10: Scanning across different retention times, DHR-123 and ABDA scores in mammalian cells. Minimum sample size of 50 compounds.

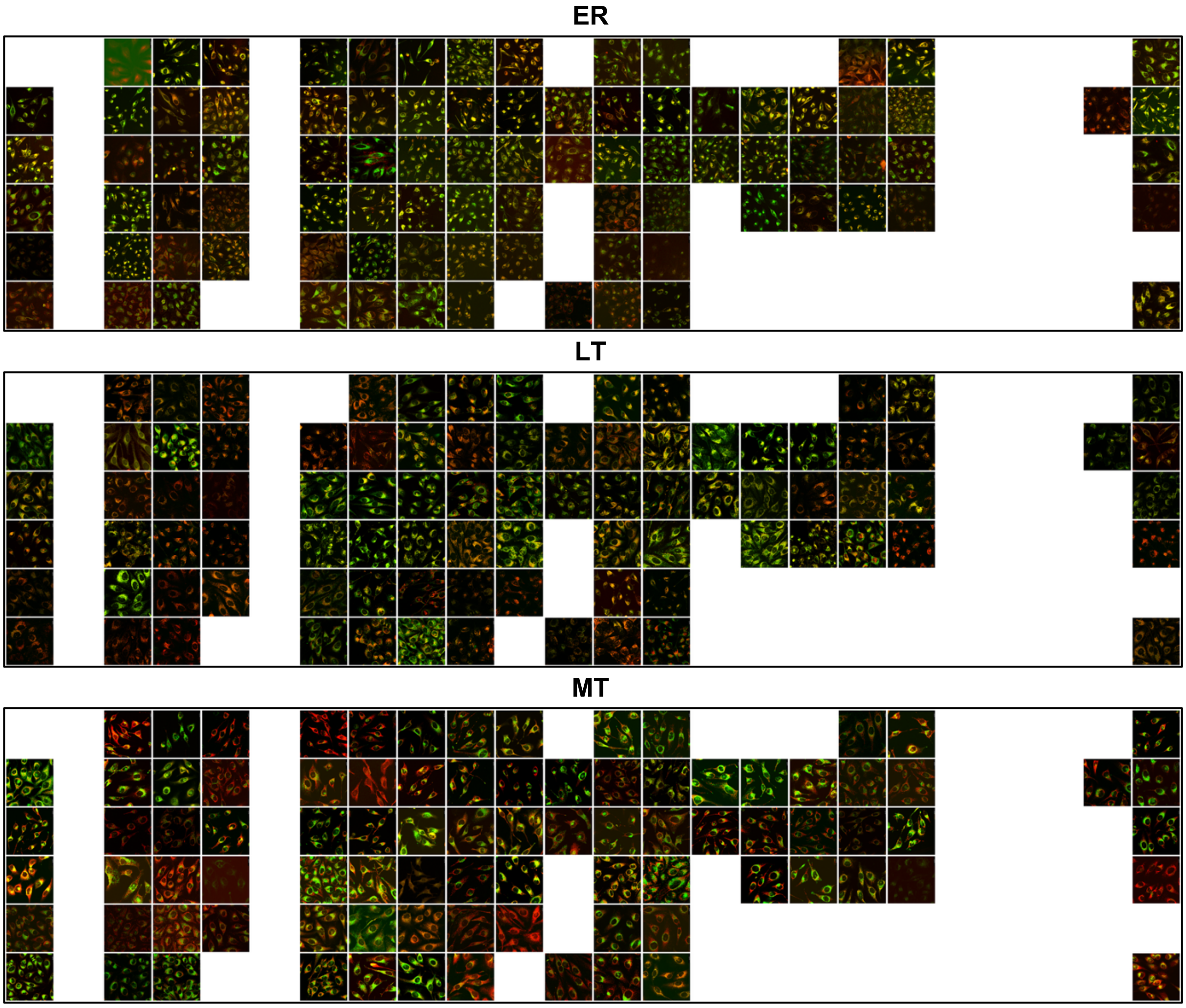

Figure S11: Co-localisation images for ER Tracker red (1 μM), Lyso Tracker red (0.1 μM) and Mitotracker deep red (1 μM). Green channel (λ_{em} = 488 nm, λ_{ex} = 500-620 nm) represents fluorescent signal of Ir-Complexes (4 μM) and Red channel (λ_{em} = 633 nm, λ_{ex} = 650-750 nm) represents fluorescent signal of tracker dyes. Co-localisation of these signals appears in a yellow-orange colour.

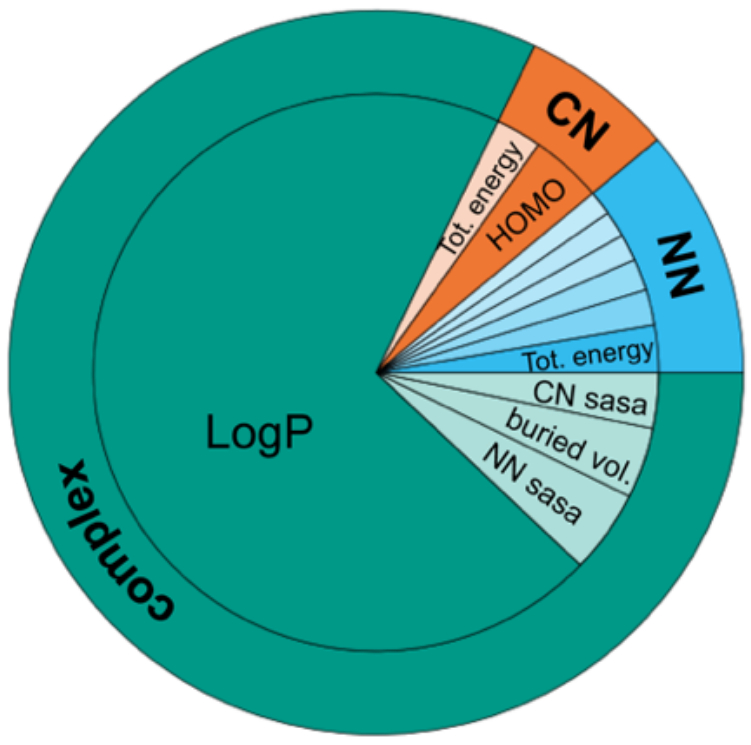

Figure S12: Top 12 ranked features for retention time prediction under the random split with ligand xTB features + complex features.

$$logP = \log\left(\frac{\Delta G_{octanol}}{\Delta G_{water}}\right) \quad (1)$$

$$logP = \frac{-\left(\Delta G^{\circ}_{octanol} - \Delta G^{\circ}_{water}\right)}{2.303\ RT} \quad (2)$$

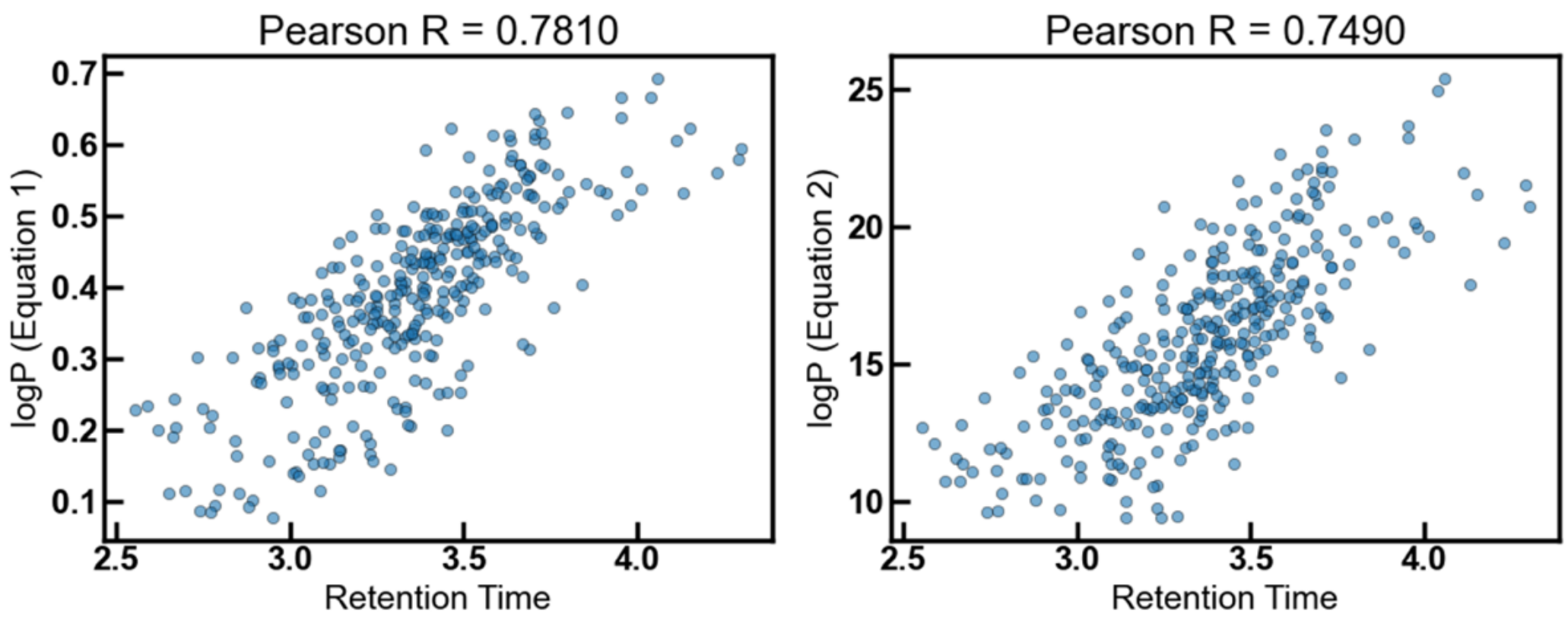

Figure S13: Linear regression for the experimentally determined retention time plotted against xTB-calculated LogP values with two different equations.

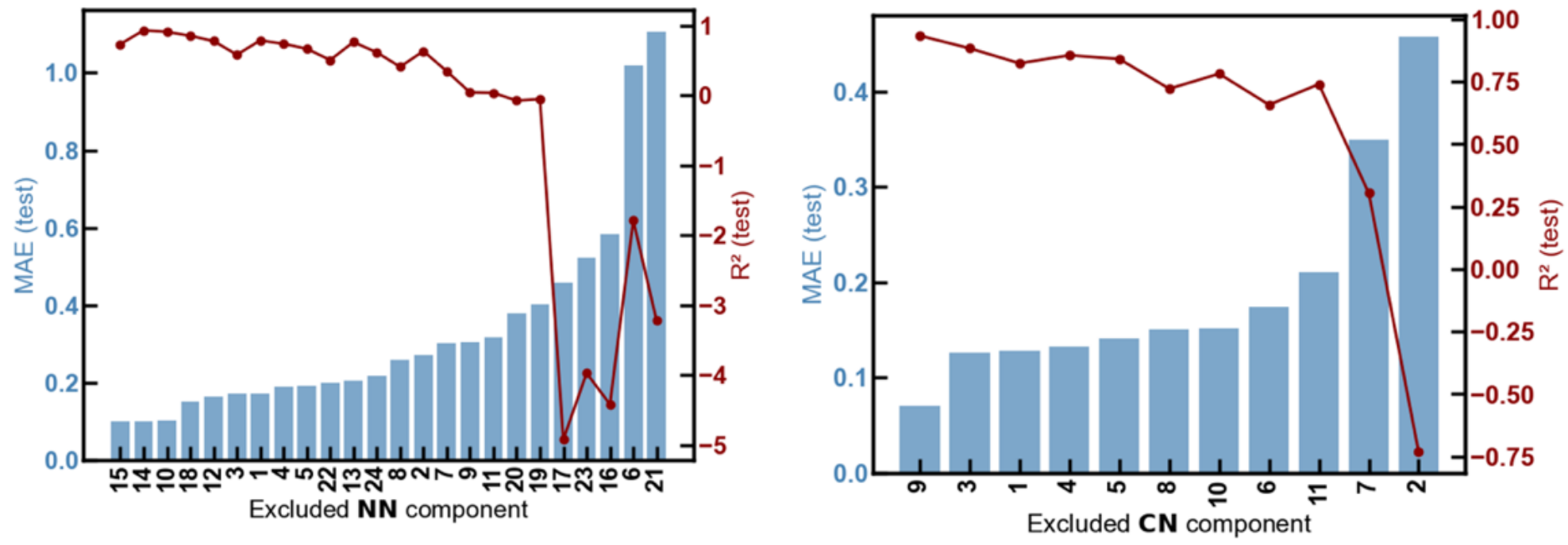

Figure S14: LOGO (leave one group out) analysis for the RF model for ABDA, either leaving out one **NN** or **CN** component.

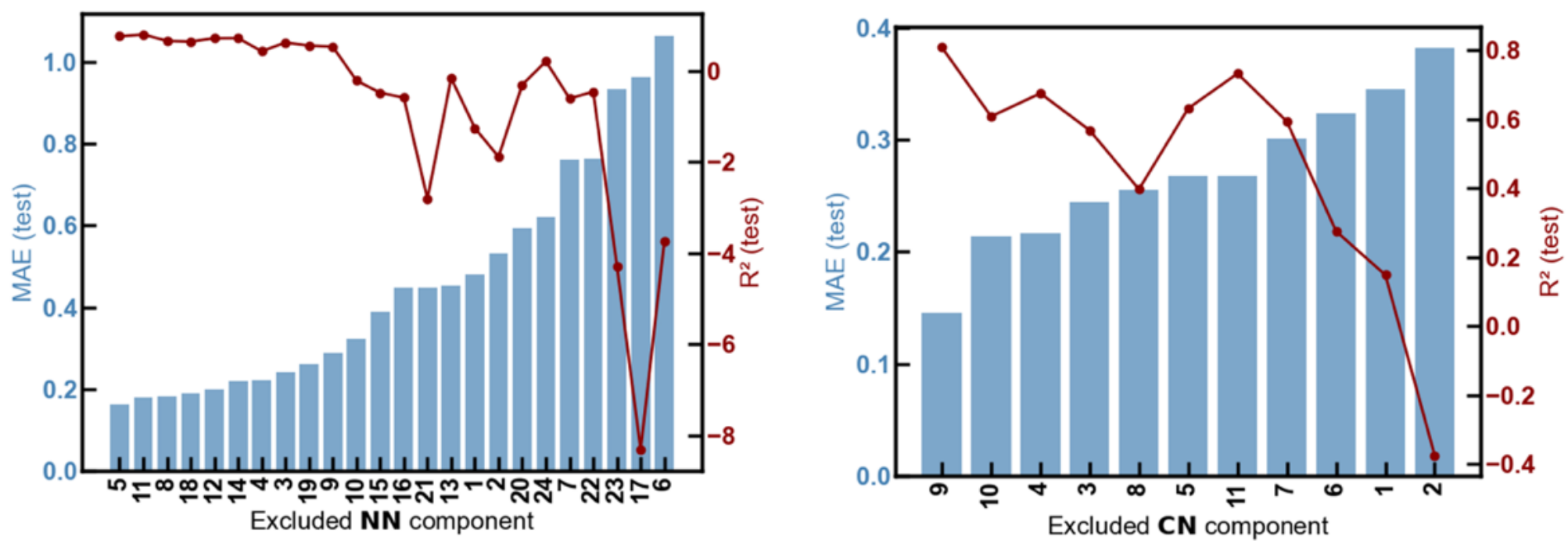

Figure S15: LOGO (leave one group out) analysis for the RF model for DHR-123, either leaving out one **NN** or **CN** component.

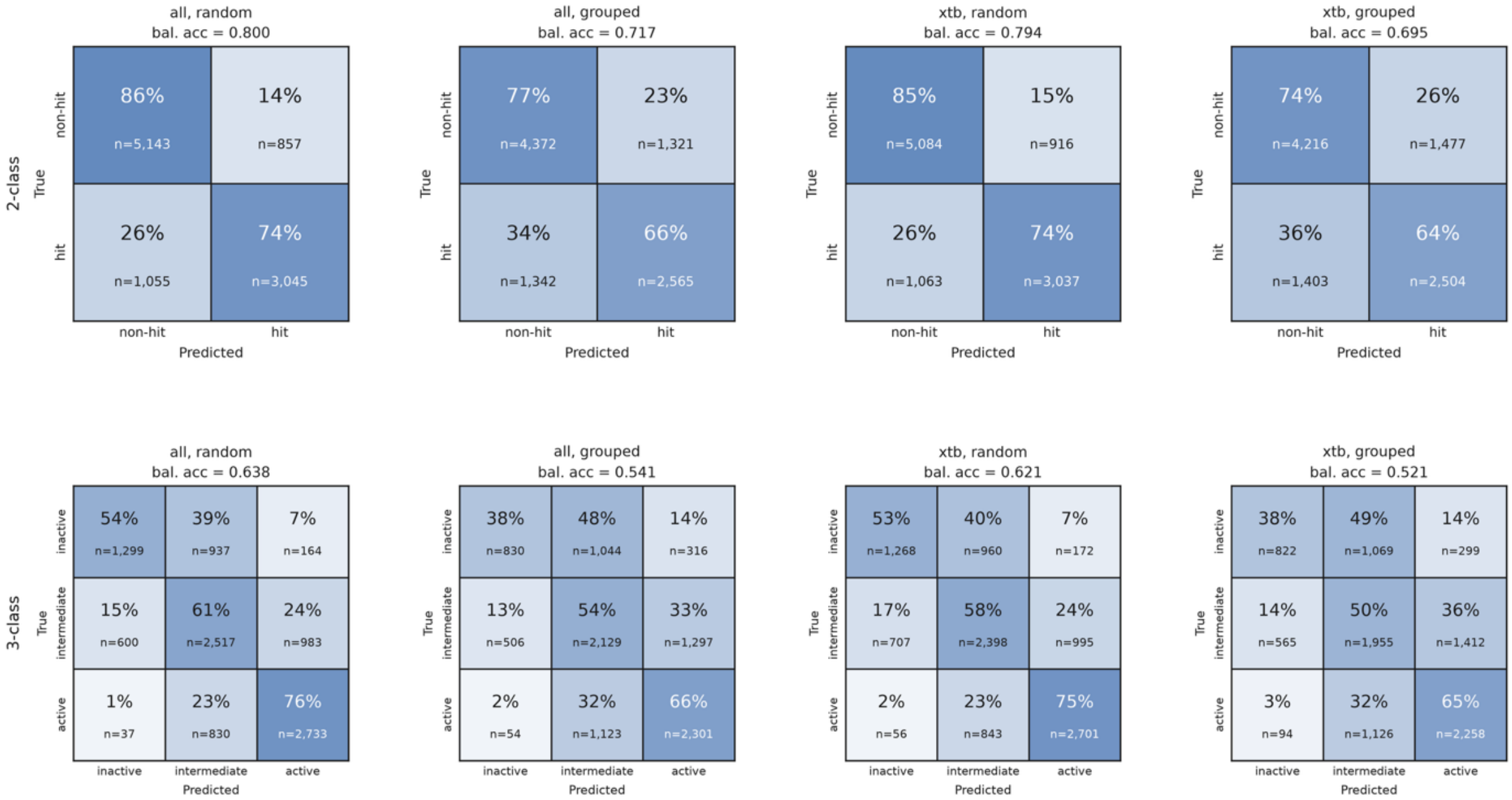

Figure S16: Confusion matrices for ‘AT3 irradiation’ RF models, in which predictions have been aggregated over 100 CV splits.

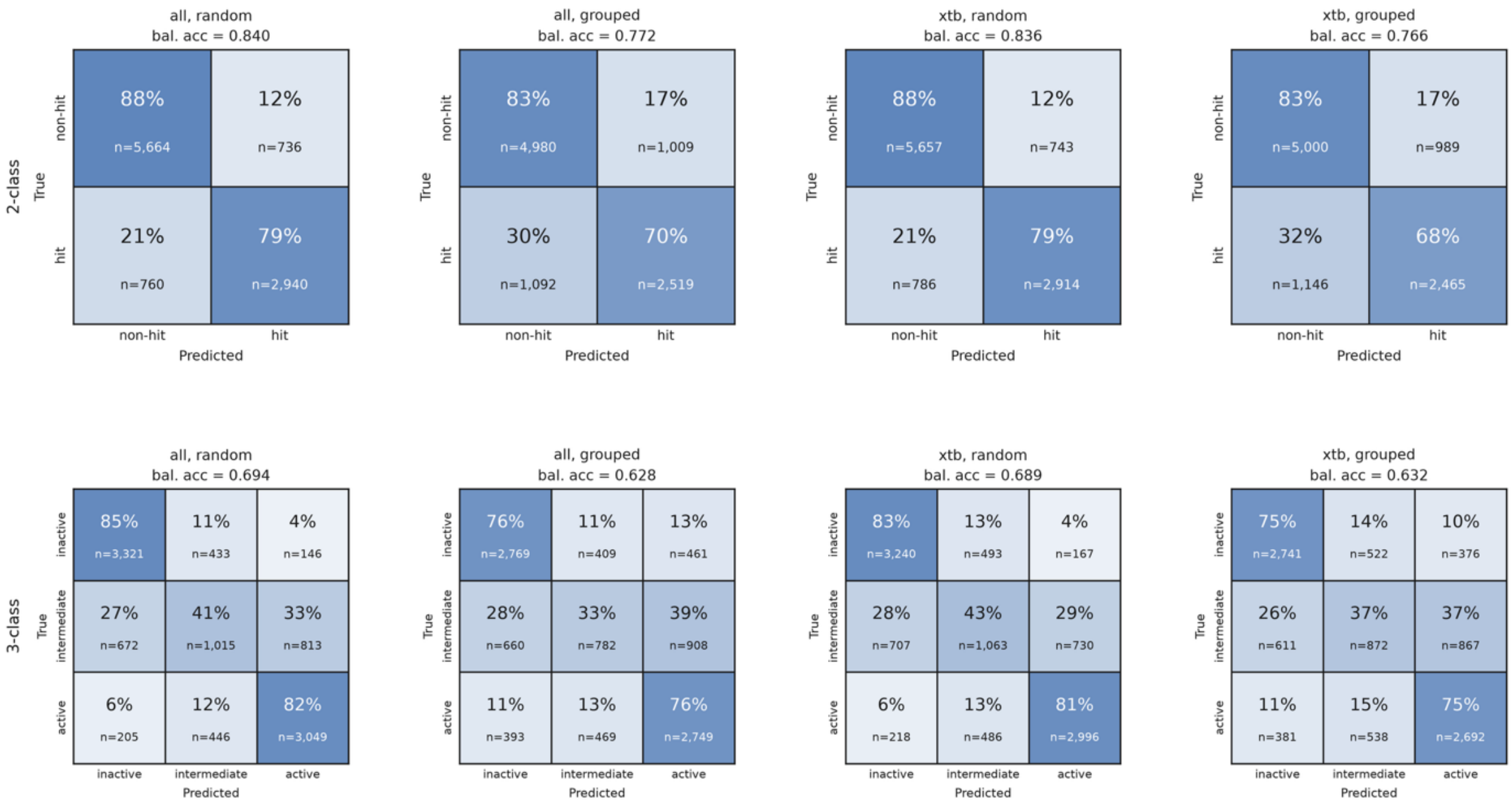

Figure S17: Confusion matrices for ‘B Subtilis dark’ RF models, in which predictions have been aggregated over 100 CV splits.

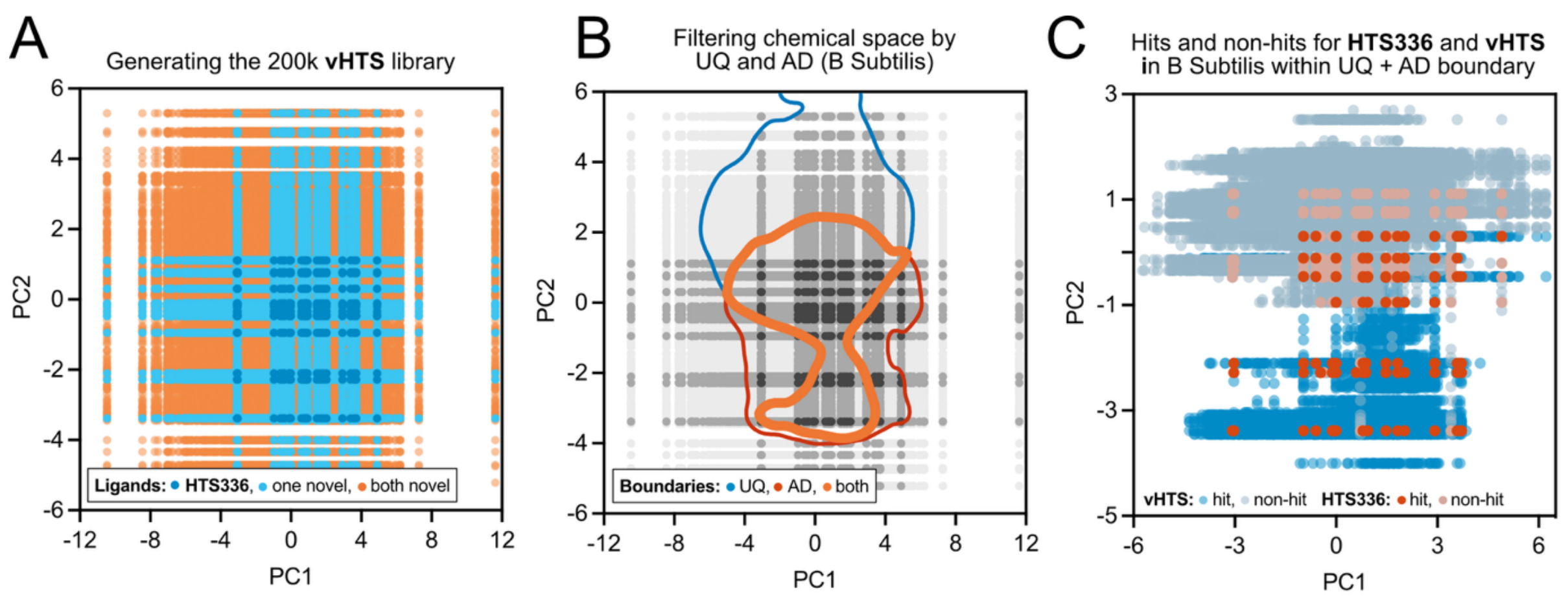

Figure S18: A) PCA analysis of a ~200k virtual library for HTS, showing extension of chemical space from **HTS336**, (B) filtering **vHTS** using uncertainty quantification and applicability domain cutoffs, for B Subtilis, (C) PCA analysis of real (**HTS336**) and predicted (**vHTS**) hits and non-hits against B Subtilis.

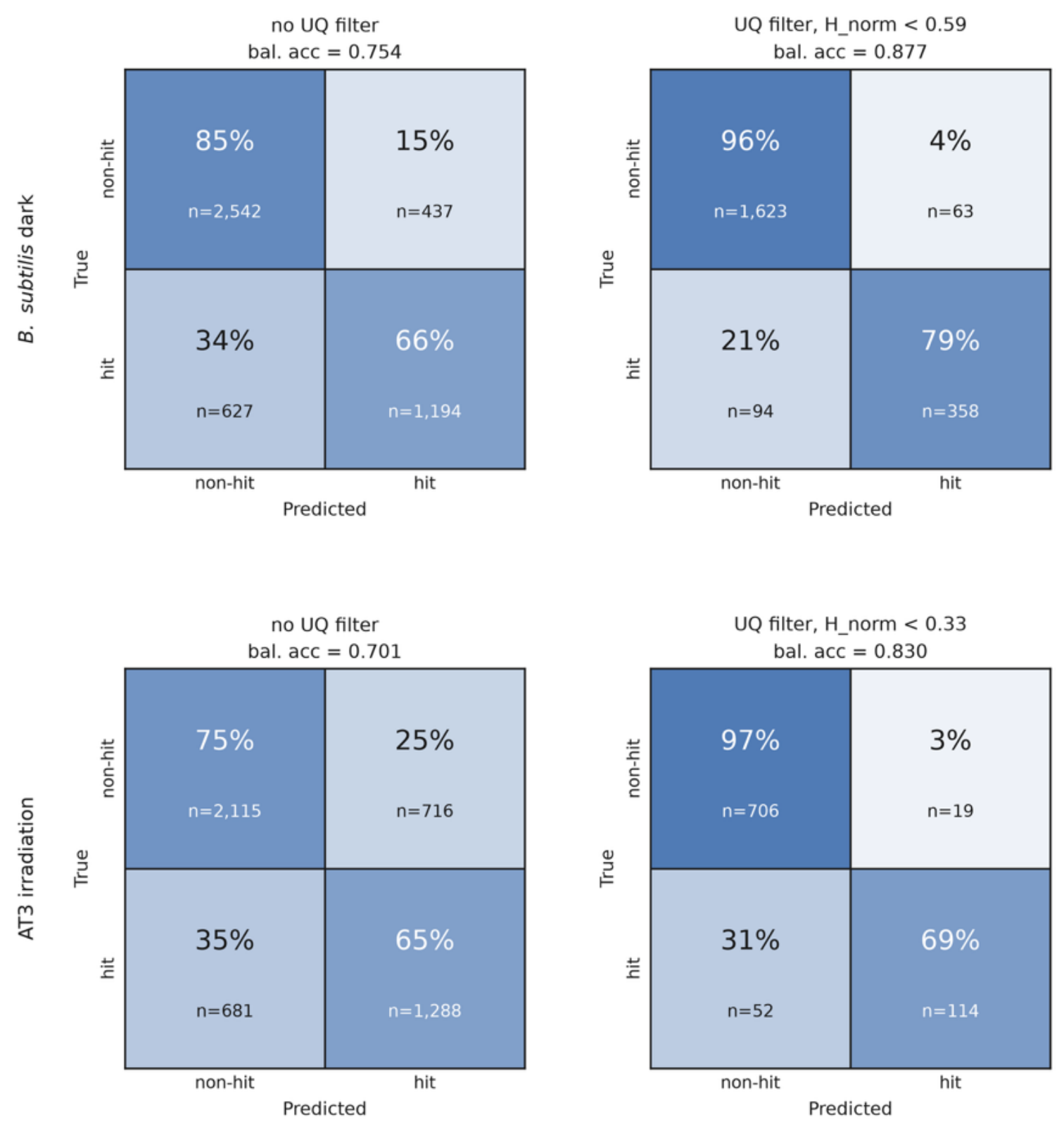

Figure S19: Confusion matrices for the virtual HTS Random Forest classifiers before and after the uncertainty (UQ) filter, aggregated over 50 grouped cross-validation splits (4 **NN** + 2 **CN** ligands withheld per split).

Table S1: Feature descriptors used in the ML analysis.

Alias names	Definitions
	**xTB features**
**n_charge**	Partial charge of nitrogen coordinating atom in the **CN** ligand
**c_charge**	Partial charge of carbon coordinating atom in the **CN** ligand
**n_f+, n_f-, n_f0**	Fukui index of nitrogen coordinating atom in the **CN** ligand. f+, f-, f0 refer to the nucleophilic, electrophilic, and radical Fukui index respectively.
**c_f+, c_f-, c_f0**	Fukui index of carbon coordinating atom in the **CN** ligand
**HOMO_eV**	HOMO (included separately for both **NN** and **CN** ligands)
**LUMO_eV**	LUMO (included separately for both **NN** and **CN** ligands)
**HOMO_LUMO_gap_eV**	HOMO-LUMO gap (included separately for both **NN** and **CN** ligands)
**total_energy_kcal**	Total energy (included separately for both **NN** and **CN** ligands)
**IP_eV**	Ionization potential (included separately for both **NN** and **CN** ligands)
**EA_eV**	Electron affinity. (included separately for both **NN** and **CN** ligands)
**n1_charge**	Partial charge of one nitrogen coordinating atom in the **NN** ligand
**n2_charge**	Partial charge of the other nitrogen coordinating atom in the **NN** ligand
**n1_f+, n1_f-, n1_f0**	Fukui index of one nitrogen coordinating atom in the **NN** ligand.
**n2_f+, n2_f-, n2_f0**	Fukui index of the other nitrogen coordinating atom in the **NN** ligand.
	**Ligand DFT**
**HOMO**	DFT-computed HOMO
**LUMO**	DFT-computed LUMO
**E_plus**	Single point energy at +1 charge
**E**	Single point energy at 0 charge
**E_minus**	Single point energy at -1 charge
**logP**	logP values with Eqn 2 using CPCM implicit solvation model
**G_octanol**	Single point energy + vibrational correction in octanol
**G_water**	Single point energy + vibrational correction in water
	**Complex DFT**
**logP**	xTB-computed logP from Eqn 1
**fraction_buried_volume**	metal buried volume
**CN_sasa**	CN ligand solvent accessible surface area
**NN_sasa**	NN ligand solvent accessible surface area

Table S2: Random forest regression model performance for physicochemical properties, with accuracy metrics. MAE = mean absolute error, MSE = mean squared error.

target	feature_set	n_features	split	Test R2	Test MAE	Test MSE
ABDA	all	48	random	0.7434 ± 0.0667	0.2074 ± 0.0177	0.0780 ± 0.0173
ABDA	all	48	grouped	0.2275 ± 0.4517	0.3392 ± 0.0845	0.1948 ± 0.1009
ABDA	xTB+complex	32	random	0.7379 ± 0.0758	0.2093 ± 0.0187	0.0795 ± 0.0191
ABDA	xTB+complex	32	grouped	0.2259 ± 0.4472	0.3391 ± 0.0830	0.1942 ± 0.0983
ABDA	xTB+DFT	44	random	0.7712 ± 0.0868	0.1900 ± 0.0190	0.0691 ± 0.0226
ABDA	xTB+DFT	44	grouped	0.1880 ± 0.3933	0.3483 ± 0.0823	0.2122 ± 0.1001
ABDA	xTB	28	random	0.7674 ± 0.0921	0.1906 ± 0.0190	0.0702 ± 0.0235
ABDA	xTB	28	grouped	0.2647 ± 0.4203	0.3215 ± 0.0954	0.1956 ± 0.1159
Retention time	all	48	random	0.8422 ± 0.0285	0.0876 ± 0.0083	0.0139 ± 0.0028
Retention time	all	48	grouped	0.5305 ± 0.2787	0.1424 ± 0.0410	0.0360 ± 0.0189
Retention time	xTB+complex	32	random	0.8369 ± 0.0292	0.0885 ± 0.0083	0.0143 ± 0.0028
Retention time	xTB+complex	32	grouped	0.5431 ± 0.2467	0.1417 ± 0.0374	0.0352 ± 0.0168
Retention time	xTB+DFT	44	random	0.8783 ± 0.0253	0.0747 ± 0.0069	0.0107 ± 0.0024
Retention time	xTB+DFT	44	grouped	0.4271 ± 0.3186	0.1651 ± 0.0458	0.0446 ± 0.0230
Retention time	xTB	28	random	0.8688 ± 0.0289	0.0781 ± 0.0075	0.0115 ± 0.0026
Retention time	xTB	28	grouped	0.4347 ± 0.3241	0.1647 ± 0.0463	0.0441 ± 0.0243
DHR-123	all	48	random	0.6599 ± 0.0540	0.2741 ± 0.0203	0.1373 ± 0.0241
DHR-123	all	48	grouped	0.2315 ± 0.2992	0.3956 ± 0.0720	0.2753 ± 0.1022
DHR-123	xTB+complex	32	random	0.6444 ± 0.0591	0.2829 ± 0.0228	0.1435 ± 0.0255
DHR-123	xTB+complex	32	grouped	0.1691 ± 0.2936	0.4144 ± 0.0742	0.3005 ± 0.1084
DHR-123	xTB+DFT	44	random	0.6883 ± 0.0537	0.2610 ± 0.0212	0.1258 ± 0.0232
DHR-123	xTB+DFT	44	grouped	0.1941 ± 0.3335	0.4055 ± 0.0791	0.2876 ± 0.1044
DHR-123	xTB	28	random	0.6825 ± 0.0587	0.2656 ± 0.0229	0.1281 ± 0.0250
DHR-123	xTB	28	grouped	0.1561 ± 0.3299	0.4172 ± 0.0854	0.3034 ± 0.1125

Table S3: Random forest classifier performance (mean ± SD) for five biological targets, two feature sets, and two split strategies, under 2- and 3-classification schemes. acc: accuracy; bacc: balanced accuracy; AUC: area under the ROC curve; F1: macro-averaged F1 score.

target	n_classes	features	split	n_class0	n_class1	n_class2	acc	bacc	auc	f1_macro	f1_class0	f1_class1	f1_class2
AT3_light	2	all	grouped	200	136	0	0.723 ± 0.084	0.717 ± 0.092	0.810 ± 0.082	0.705 ± 0.093	0.765 ± 0.073	0.646 ± 0.130	
AT3_light	2	all	random	200	136	0	0.811 ± 0.035	0.800 ± 0.036	0.887 ± 0.027	0.802 ± 0.036	0.843 ± 0.030	0.760 ± 0.046	
AT3_light	2	xTB	grouped	200	136	0	0.700 ± 0.078	0.695 ± 0.087	0.788 ± 0.087	0.683 ± 0.087	0.744 ± 0.067	0.622 ± 0.125	
AT3_light	2	xTB	random	200	136	0	0.804 ± 0.030	0.794 ± 0.032	0.875 ± 0.027	0.795 ± 0.032	0.837 ± 0.026	0.754 ± 0.040	
BSub_dark	2	all	grouped	214	122	0	0.781 ± 0.071	0.772 ± 0.070	0.872 ± 0.054	0.761 ± 0.072	0.820 ± 0.068	0.701 ± 0.096	
BSub_dark	2	all	random	214	122	0	0.852 ± 0.034	0.840 ± 0.037	0.922 ± 0.027	0.840 ± 0.037	0.883 ± 0.028	0.797 ± 0.047	
BSub_dark	2	xTB	grouped	214	122	0	0.778 ± 0.064	0.766 ± 0.068	0.864 ± 0.049	0.756 ± 0.066	0.819 ± 0.062	0.692 ± 0.091	
BSub_dark	2	xTB	random	214	122	0	0.849 ± 0.032	0.836 ± 0.035	0.919 ± 0.028	0.836 ± 0.034	0.881 ± 0.026	0.792 ± 0.044	
BSub_light	2	all	grouped	205	131	0	0.780 ± 0.071	0.781 ± 0.071	0.879 ± 0.053	0.766 ± 0.073	0.811 ± 0.068	0.721 ± 0.095	
BSub_light	2	all	random	205	131	0	0.843 ± 0.032	0.834 ± 0.036	0.924 ± 0.024	0.834 ± 0.035	0.872 ± 0.026	0.796 ± 0.045	
BSub_light	2	xTB	grouped	205	131	0	0.786 ± 0.061	0.784 ± 0.064	0.874 ± 0.049	0.772 ± 0.063	0.818 ± 0.060	0.726 ± 0.085	
BSub_light	2	xTB	random	205	131	0	0.844 ± 0.033	0.835 ± 0.037	0.922 ± 0.025	0.835 ± 0.035	0.873 ± 0.027	0.797 ± 0.045	
HEK_light	2	all	grouped	237	99	0	0.778 ± 0.075	0.720 ± 0.087	0.839 ± 0.075	0.715 ± 0.092	0.845 ± 0.057	0.584 ± 0.141	
HEK_light	2	all	random	237	99	0	0.838 ± 0.028	0.787 ± 0.041	0.904 ± 0.027	0.797 ± 0.038	0.888 ± 0.020	0.706 ± 0.058	
HEK_light	2	xTB	grouped	237	99	0	0.768 ± 0.073	0.718 ± 0.087	0.816 ± 0.077	0.708 ± 0.089	0.836 ± 0.056	0.580 ± 0.137	
HEK_light	2	xTB	random	237	99	0	0.831 ± 0.028	0.783 ± 0.041	0.892 ± 0.028	0.790 ± 0.038	0.882 ± 0.020	0.698 ± 0.058	
HeLa_light	2	all	grouped	224	112	0	0.728 ± 0.080	0.691 ± 0.088	0.788 ± 0.078	0.678 ± 0.092	0.800 ± 0.065	0.557 ± 0.134	
HeLa_light	2	all	random	224	112	0	0.800 ± 0.035	0.756 ± 0.040	0.874 ± 0.034	0.766 ± 0.040	0.855 ± 0.026	0.676 ± 0.057	
HeLa_light	2	xTB	grouped	224	112	0	0.704 ± 0.076	0.672 ± 0.087	0.760 ± 0.086	0.656 ± 0.087	0.780 ± 0.062	0.531 ± 0.129	
HeLa_light	2	xTB	random	224	112	0	0.792 ± 0.034	0.749 ± 0.039	0.868 ± 0.035	0.757 ± 0.039	0.848 ± 0.026	0.665 ± 0.056	
AT3_light	3	all	grouped	78	137	121	0.548 ± 0.093	0.541 ± 0.086	0.742 ± 0.064	0.531 ± 0.089	0.467 ± 0.117	0.514 ± 0.101	0.613 ± 0.117
AT3_light	3	all	random	78	137	121	0.648 ± 0.044	0.638 ± 0.048	0.812 ± 0.029	0.642 ± 0.047	0.595 ± 0.083	0.599 ± 0.054	0.731 ± 0.043
AT3_light	3	xTB	grouped	78	137	121	0.524 ± 0.087	0.521 ± 0.082	0.717 ± 0.066	0.509 ± 0.083	0.449 ± 0.114	0.480 ± 0.102	0.597 ± 0.108
AT3_light	3	xTB	random	78	137	121	0.630 ± 0.045	0.621 ± 0.047	0.797 ± 0.030	0.623 ± 0.047	0.569 ± 0.076	0.577 ± 0.058	0.723 ± 0.047
BSub_dark	3	all	grouped	131	83	122	0.656 ± 0.071	0.628 ± 0.060	0.830 ± 0.041	0.608 ± 0.069	0.738 ± 0.087	0.380 ± 0.109	0.707 ± 0.089
BSub_dark	3	all	random	131	83	122	0.731 ± 0.038	0.694 ± 0.039	0.877 ± 0.027	0.690 ± 0.041	0.820 ± 0.037	0.459 ± 0.078	0.791 ± 0.043
BSub_dark	3	xTB	grouped	131	83	122	0.657 ± 0.069	0.632 ± 0.062	0.819 ± 0.041	0.615 ± 0.071	0.740 ± 0.082	0.399 ± 0.116	0.707 ± 0.085
BSub_dark	3	xTB	random	131	83	122	0.723 ± 0.041	0.689 ± 0.043	0.871 ± 0.027	0.686 ± 0.045	0.803 ± 0.037	0.466 ± 0.082	0.789 ± 0.047
BSub_light	3	all	grouped	107	98	131	0.625 ± 0.071	0.624 ± 0.063	0.810 ± 0.047	0.600 ± 0.071	0.665 ± 0.113	0.412 ± 0.098	0.724 ± 0.083
BSub_light	3	all	random	107	98	131	0.714 ± 0.040	0.703 ± 0.041	0.875 ± 0.025	0.699 ± 0.042	0.775 ± 0.051	0.532 ± 0.078	0.791 ± 0.041
BSub_light	3	xTB	grouped	107	98	131	0.626 ± 0.078	0.624 ± 0.067	0.801 ± 0.048	0.602 ± 0.075	0.656 ± 0.115	0.426 ± 0.098	0.725 ± 0.086
BSub_light	3	xTB	random	107	98	131	0.712 ± 0.036	0.701 ± 0.037	0.871 ± 0.025	0.699 ± 0.038	0.766 ± 0.051	0.541 ± 0.067	0.789 ± 0.033
HEK_light	3	all	grouped	113	134	89	0.570 ± 0.074	0.579 ± 0.071	0.780 ± 0.048	0.567 ± 0.078	0.669 ± 0.084	0.535 ± 0.094	0.497 ± 0.128
HEK_light	3	all	random	113	134	89	0.670 ± 0.042	0.669 ± 0.043	0.846 ± 0.024	0.668 ± 0.043	0.784 ± 0.047	0.606 ± 0.053	0.615 ± 0.073
HEK_light	3	xTB	grouped	113	134	89	0.562 ± 0.076	0.571 ± 0.072	0.755 ± 0.050	0.559 ± 0.079	0.668 ± 0.084	0.521 ± 0.089	0.489 ± 0.116
HEK_light	3	xTB	random	113	134	89	0.652 ± 0.044	0.650 ± 0.045	0.831 ± 0.026	0.650 ± 0.045	0.769 ± 0.049	0.591 ± 0.053	0.590 ± 0.077

HeLa_light	3	all	grouped	98	141	97	0.552 ± 0.077	0.562 ± 0.075	0.762 ± 0.057	0.550 ± 0.079	0.614 ± 0.102	0.554 ± 0.090	0.484 ± 0.116
HeLa_light	3	all	random	98	141	97	0.660 ± 0.040	0.663 ± 0.041	0.838 ± 0.027	0.664 ± 0.040	0.737 ± 0.055	0.625 ± 0.050	0.629 ± 0.056
HeLa_light	3	xTB	grouped	98	141	97	0.544 ± 0.073	0.556 ± 0.074	0.740 ± 0.060	0.544 ± 0.074	0.623 ± 0.088	0.529 ± 0.095	0.481 ± 0.114
HeLa_light	3	xTB	random	98	141	97	0.648 ± 0.043	0.652 ± 0.044	0.827 ± 0.026	0.653 ± 0.043	0.720 ± 0.058	0.605 ± 0.051	0.634 ± 0.060

Experimental Methods

Synthesis

^{1}H and ^{13}C NMR spectra were recorded on a Bruker Avance 400 MHz (with a Bruker SampleJet attachment for the calibration and final complex syntheses). LCMS analysis was carried out on an Agilent 1260 Infinity with a Raptor C18 column (50 mm x 2.1 mm, 2.7 µm particle size). A two-minute gradient from 5-95% MeCN in water was used, supplemented with 0.1% formic acid. All chemicals were purchased from Sigma–Aldrich, Fluorochem or VWR and used without further purification.

General procedures

General synthetic procedure for NN ligands: A mixture of 1,10-phenanthroline-5,6-dione (0.52 g, 2.4 mmol), one equivalent of the corresponding aldehyde (2.4 mmol), ammonium acetate (3.8 g, 50 mmol) and glacial acetic acid (10 mL) was refluxed for 3 h, then cooled to room temperature and diluted with water (10 mL). Dropwise addition of concentrated aqueous ammonia to neutralize gave a yellow precipitate, which was collected and washed with water and ice-cold ethanol. Characterisation details for each of these ligands are provided below.

*General procedure for the synthesis of iridium dimers $[Ir(\mathbf{CN})(\mu\text{-}Cl)]_2$ (for **CN01-CN04**):* A microwave vial equipped with a magnetic stirrer was charged with 1.0 mL of water, 3.0 mL of 2-methoxyethanol, 0.41 mmol of the corresponding CN ligand, and iridium(III) chloride hydrate (53% iridium content) (68 mg, 0.187 mmol). The reaction vessel was closed and placed in a microwave reactor and then heated for10 minutes at 150 °C. An additional 1.0 mL of water was added, followed by filtration of the resulting precipitate which was then washed with methanol and diethyl ether. Characterisation details for each of these complexes (i.e., $[Ir(\mathbf{CN})(\mu\text{-}Cl)]_2$ with **CN01-14**) are provided below.

General procedure for the preparative synthesis of iridium complexes: For the complexes that were isolated and purified, the following steps were carried out: first, the appropriate iridium dimer (0.017 mmol) and **NN** ligand (0.03 mmol) were mixed in a mixture of chloroform (15 mL) and methanol (5 mL) and stirred at 40 ˚C overnight. Next, the solvent was removed, and the complex purified via column chromatography (0-10%methanol in CH_2Cl_2).

High-throughput synthesis

The high-throughput synthetic step was carried out via the following: first, an appropriate quantity of the **NN** ligands and **CN**-containing iridium(III) dimers were prepared in DMSO-d^6 at a concentration of between 10-15 mM (calculated per iridium centre for the **CN**-dimers). Next, 300 µL of this solution was combined with 100 µL of a 20 mM stock of ethylene carbonate, for which enough stock was prepared so the whole library was calibrated to the same solution. After the actual concentrations had been calculated, reaction plates were prepared in which the appropriate quantity of **NN** ligand and **CN**-containing dimer was added to reach a final concentration of 1 mM (again, per iridium(III) centre for the **CN**-dimers) in 200 µL of DMSO. Next, the reaction plates were heated to 150 °C for 4 hours, after which they were allowed to cool and transferred to a separate well of a 2 mL Porvair polypropylene plate.

LCMS analysis

Purities of the resulting complexes calculated by averaging the automatically integrated peak areas of 5 wavelengths of the UV spectra in the LCMS reports after 1 minute to account for the injection peak.

Assay plate preparation

All assay plate preparation was carried out using the Beckmann Coulter Echo. A 384-well plate was prepared with the entire library. This stock plate was then used to prepare GreinerBio black µClear 384/1536 plates.

Irradiation

Irradiation was carried out as followed: for 96-well plates, a Lumidox 470 nm 96-LED array was set to 2 mA (~2 mW flux), and the plate placed directly on top of the array for 12 minutes. For 384 well-plates, in order for each well of the 384 well plate to receive a more equal dose of light, a clear 96-well plate was placed between the Lumidox array and the plate being irradiated, and the LED array set to 15 mA (~6 mW flux) to account for the drop off in flux with the additional distance.

ABDA ROS generation

A series of plates were prepared in which 800 nL of the appropriate complex was combined with 50 µL of a 200 µM ABDA stock solution in PBS, leading to a final complex concentration of 16 µM. $[Ru(bpy)_3]^{2+}$ at the same concentration was added to the plate. The absorbance of the whole plate was measured using a BMG LabTech Clariostar plate reader, then the plate was irradiated and shaken for 30 seconds and the absorbance remeasured. This process was repeated for the following timepoints: 60, 120 and 180 seconds. The experiment was repeated in triplicate and averaged. The absorbance at 380 nm corresponding to the ABDA maxima was extracted, fitted to a linear regression and normalised to $[Ru(bpy)_3]^{2+}$.

DHR-123 ROS generation

A series of plates were prepared in which 50 nL of the appropriate complex was combined with a 10 µM DHR-123 stock solution in PBS in triplicate, leading to a final complex concentration of 1 µM. $[Ru(bpy)_3]^{2+}$ at the same concentration was added to the plate. The emission in each well was measured using λ_{ex} = 505 nm and λ_{em} = 535 nm using a BMG LabTech Clariostar plate reader, then the plate was irradiated, shaken for 30 seconds and the emission remeasured. This process was repeated for the following timepoints: 60, 120, 180 and 240 seconds. The experiment was repeated in triplicate and averaged. The emission was fitted to a linear regression and normalised to $[Ru(bpy)_3]^{2+}$.

Tissue culture

HeLa, AT3 and HEK cells were grown in high glucose Dulbecco's modified Eagle medium (DMEM) containing 10% foetal bovine serum at 37 humidified air.

Mammalian Cell Screening

Cells were plated into 384 well plates at a volume of 25 µL/well and 1250 cells/well, in which two columns were reserved for negative (no compound) and positive (no cells) controls. After 24 hours, complexes were added either using the following protocols, dark; three-point screen (5, 10 and 15 µM) to distinguish between those with reasonable toxicity (<50% viability at 5 µM) from ones likely to be non-toxic, i.e. >50% viability at 15 µM, and light; a two-point screen at 1.5 and 10 µM, with the aim of identifying highly active complexes (those with complete cell death at 1.5 µM) and those unlikely to be taken up at all by cells (no toxicity at 10 µM under light irradiation). Complexes were taken forward for light IC_{50} determination if they

achieved <40% cell viability at 1.5 μM. For IC_{50} determination, complexes were tested at 0.2, 0.4, 0.6, 0.8, 1 and 2 μM, again in triplicate. Irradiation was carried out four hours after complex addition. Remaining cell viability was calculated 24 hours after initial complex addition using the Promega MTS assay.

Bacterial screening

Complexes were plated into 1536-well plates in which two sets of three columns were reserved for controls (bacteria, DMSO, and chloramphenicol). For bacterial screening a two-point screen was carried out (30 μM and 5 μM) under both light and dark conditions. Complexes were taken forward for MIC analysis if they showed <20% remaining viability under irradiation conditions at the lower concentration of 5 μM. MICs were calculated for this subset in both the light and the dark conditions due to similarity in behaviour profile observed in the initial screen. For MIC calculation, the following concentrations were tested: 0.5, 1, 2, 4, 8, 16 μM. Irradiation was carried out four hours after complex addition. Remaining bacteria viability was calculated 24 hours after initial complex addition using OD measurements.

Co-localisation

HeLa cells were plated at 5000 cells per well in a 96-wellplate (Grenier Bio-One, CELLSTAR® plate) and allowed to adhere in high-glucose DMEM at 37 °C and 5% CO_2. After 24h, Ir-complexes were added at 4 μM to the inner 48 wells (ignoring all wells closest to the edge) and left to incubate for a further 4h. To wells containing Ir-complexes, either Mitotracker deep red (1 μM), Lysotracker red (100 nM) or ER tracker red (1 μM) were added to the wells and left to incubate for 1h further before the media was removed and cells were washed with DMEM.

Confocal Microscopy

Confocal Microscopy images were either collected using Nikon Eclipse Ti-2 or Leica SPII 5 inverted microscope.

For images collected with the Nikon Eclipse Ti-2, samples were excited at 470 nm and the emission was collected between 500-600 nm. Images were collected using an automated stage with auto-focus used before collecting each image using a 20x air objective.

For images collected using Leica SPII 5, samples were excited at either 488 nm (internal Ar+ laser) or 633 nm (internal HeNe laser) and emission was collected at 500-620 nm and 650-750 nm respectively. Images were collected at 512 x 512 pixels resolution with repetition rate of 8 frames using a 20x object and zoom factor of 1.5x. For Mitotracker deep red and Lysotracker red, both channels were collected simultaneously, however for ER tracker red, the Ir-complex images were collected first, then ER tracker red images to prevent any signal appearing in the incorrect channel as there was some overlap between the dye and complex emissions.

IC_{50} Determination using MTT.

MCA205 fibrosarcoma cells were detached, harvested, and seeded at a density of 6,000 cells per well in a 96-well plate. The cells were then incubated for 24 h at 37 °C and 10% CO_2. Subsequently, the cells were treated with different concentrations of the tested substances in full RPMI at a constant DMSO concentration of 0.5% and incubated for 4 h. Afterwards, the cell medium in each well was exchanged by fresh medium and the plate was irradiated with blue light for 10 minutes (450 nm, power: 20%, 10 min, 1.2 J/cm^2). A solution of 0.5% DMSO in cell growth medium served as a negative control and one series of cells was treated with varying concentrations of auranofin as a positive control. After 24 h of incubation, a solution of MTT in DPBS (50 µL, 2.5 mg/mL) was added and incubated for 2 h. The supernatant solution was carefully removed and DMSO (200 µL) was added to each well. The absorbance of formazan crystals was measured at 550 nm, using a reference wavelength of 620 nm, with either a Tecan Infinite Nano M or a BMG Labtech Clariostar plate reader.

Cell Death

MCA205 cells were detached, harvested, and seeded at a density of 500,000 cells per well in 6-well plates. After incubation at 37°C at 5% CO_2 overnight, the cells were treated with varying concentrations of the test compound with a final DMSO concentration of 0.5%. After 4 h of incubation, the medium in each well was exchanged with fresh medium and the plate was irradiated with blue light for 10 minutes (450 nm, power: 20%, 10 min, 1.2 J/cm^2). A solution of 0.5% DMSO in cell growth medium served as a negative control. After 24 h, the media was collected. The cells were washed with DPBS and detached using 300 µL TryplE. TryplE was deactivated by addition of 900 µL of DPBS and the cell suspensions were added to the respective tube. The cells were pelleted by centrifugation (300 x g, 4°C, 5 min), the supernatant was removed, and the cells were resuspended in Annexin-V binding buffer (200 µL, 10 mM Hepes, pH 7.4, 0.14 mM NaCl and 2.5 mM CaCl2). SYTOX blue with a final concentration of 5 µM was added and 5 µL of Annexin-V Alexa Fluor

488 conjugate were added per tube. After 10 minutes incubation on ice, cell death was determined via FACS analysis.

Kinetic Extracellular ATP release.

MCA205 fibrosarcoma cells were detached, harvested, and seeded at a density of 10,000 cells per well in a white, opaque-walled 96 well plate (BrandPlates, 781965) using full RPMI medium. The cells were incubated for 24 h at 37 °C at 10% CO_2. Different complex concentrations were added and incubated for 4 h, after which the medium in each well was exchanged by prewarmed L-15 media (10% FBS, 100 µL) and the plate was irradiated with blue light for 10 minutes (450 nm, power: 20%, 10 min, 1.2 J/cm^2). The assay solution was prepared according to the manual provided (Promega, RealTime-Glo MT Extracellular ATP Assay) using L-15 media. The compound solutions were added to the respective wells (50 µL). Subsequently, assay solution was added (50 µL) and the plate was sealed using a Breathe-Easy membrane (Diversified Biotech). The luminescence intensity was recorded using a BMG Labtech Clariostar Plate reader at a temperature of 37 °C, every 10 min, for 24 h.

HMGB1

MCA205 cells were detached, harvested, and seeded at a density of 10,000 cells per well in a white, opaque-walled 96 well plate (BrandPlates, 781965) using full RPMI medium (40 µL). Row 1 and 2 of the plate were left empty. Cells were incubated at 37 °C, with 10% CO_2 for 24 h. A 1% DMSO solution in RPMI was prepared as a negative control. Dilution series of the different samples in RPMI were prepared, maintaining a DMSO concentration of 1%. The samples were added (40 µL) to the respective wells and incubated at 37 °C, with 10% CO_2 for 4 h, after which the medium in each well was exchanged by fresh medium and the plate was irradiated with blue light for 10 minutes (450 nm, power: 20%, 10 min, 1.2 J/cm^2). As a positive control, HMGB1 was thawed and a dilution range was prepared (0.0, 4.1, 10.2, 25.6, 64.0, 160.0, 400.0, 1000.0 ng/mL). The positive control was added to the wells in row 1 and 2 (80 µL per well). The HMGB1 antibody mixture was prepared according to protocol and added to the 96 well plate (20 µL per well). The plate was shaken (10 s, 300 rpm) and afterwards incubated for 80 min at 37 °C, with 10% CO_2. The Lumit detection mixture was prepared according to the manual, added to the 96 well plate (25 µL) and the plate was shaken (10 s, 300 rpm). After an incubation period of 5 min, the luminescence was recorded using a plate reader.

Translocation of CRT

For the detection of cell surface–exposed CRT, 500,000 MCA205 cells per well were seeded in a 6-well plate. After 24 h of incubation at 37°C and 10% CO_2, cells were treated with the respective compounds in the given concentrations and incubated for 4 h after which the medium was exchanged and the plate irradiated with blue light for 10 minutes (450 nm, power: 20%, 10 min, 1.2 J/cm^2). After irradiation, the cells were incubated for further 20 h. Subsequently, the medium was collected in tubes. The cells were washed with DPBS and detached using 300 µL TryplE. TryplE was neutralized by addition of 900 µL of DPBS and the cell suspensions were added to the respective tube. Cells were pelleted, washed twice with ice-cold FACS buffer, and resuspended in 50 µL of the appropriate staining solution prepared on ice (anti-calreticulin antibody ab210431, 1:100 in FACS buffer). Subsequently, cells were washed with ice-cold FACS buffer and finally resuspended in 350 µL FACS buffer containing SYTOX green (0.5 µM). Flow cytometry was performed on a BD FACSCanto device.

Computational Methods

3D structure generation

Three-dimensional starting geometries were generated for all ligands. Each canonical SMILES was converted to a 3D structure using the ETKDGv3[1] conformer generation algorithm. The embedded conformer was then minimised with the Universal Force Field[2] (UFF, maximum 2000 iterations). The lowest energy conformer was selected as the final ligand geometry. All iridium complex geometries were constructed from ligand 3D structures using molSimplify.[3] These geometries were then optimized using the GFN2-xTB[4] method with analytical linearized Poisson-Boltzmann (ALPB) solvent correction of water (--alpb flag).

xTB semi-empirical calculations

GFN2-xTB calculations were run per ligand. The --vipea flag was used to compute the vertical ionisation potential (IP) and electron affinity (EA) by self-consistent-charge single-point calculations on the neutral, cationic, and anionic species; the atomic partial charges for the neutral ground state were extracted, as well as HOMO and LUMO energies and total energy. The --vfukui flag was used to obtain condensed Fukui functions (f^+, f^-, f^0) at each atom. **CN** ligands were calculated at formal charge −1 (reflecting the anionic cyclometallating carbon) and in the doublet; **NN** ligands were calculated at formal charge 0 and in the singlet state.

The xTB output files were parsed to extract a 14-descriptor feature vector for each ligand. For **CN** ligands the descriptors were: partial charge at the coordinating N (n_charge) and C (c_charge); condensed Fukui indices (f^+, f^-, f^0) at N and C; HOMO energy (eV); LUMO energy (eV); HOMO-LUMO gap (eV); total energy (converted from hartrees to kcal/mol using the factor 627.509 kcal/mol/Eh); vertical IP (eV); and vertical EA (eV). For **NN** ligands the same orbital and energy descriptors were extracted, with partial charges and Fukui functions collected at both coordinating nitrogen atoms (n1 and n2).

DFT calculations

To assess whether the semiempirical GFN2-xTB method provides sufficient accuracy, density functional theory (DFT) calculations were done with Orca 5.0.1.[5] Ligand geometries were first optimized at the B3LYP/def2-SVP level, followed by single-point energy calculations on the optimized neutral geometries at the B3LYP/def2-TZVP level. The calculations were done by using C-PCM[6] implicit solvation model of water and D4[7] dispersion corrections. The single-point

calculations were carried out at three different charges: 0, +1 and -1, which allowed us to calculate vertical IP and EA values analogous to those obtained from GFN2-xTB. The HOMO and LUMO was extracted from the neutral single point calculation. We specified ligands to be in a singlet state when neutral and in a doublet state otherwise.

Additional complex-level descriptors

Complex-level descriptors were computed from the optimized iridium complex geometries. Each complex in HTS336 was geometry optimized in octanol and water using GFN2-xTB with ALPB solvent correction to estimate logP of the entire complex. The theoretical logP values were calculated from two different equations (1 and 2, below) and they demonstrated good correlation with experimentally determined retention time (Figure S13). Three steric descriptors, which are the metal buried volume[8] (fraction_buried_volume) and ligand solvent accessible surface area[9,10] (CN_sasa, NN_sasa), were calculated from xTB-optimized complex geometries in water using the MORFEUS package.[11] A larger buried volume corresponds to increased steric bulk around the metal center.

$$logP = \log\left(\frac{\Delta G_{octanol}}{\Delta G_{water}}\right) \tag{1}$$

$$logP = \frac{-(\Delta G^{\circ}_{octanol} - \Delta G^{\circ}_{water})}{2.303\,RT} \tag{2}$$

Random Forest model validation

Random Forest classifiers were trained and evaluated across five biological targets (BSub dark MIC, BSub light MIC, AT3 light IC50, HeLa light IC50, HEK light IC50), two labelling schemes, and two feature sets. All models comprised 500 trees with max_features='sqrt' and min_samples_leaf=1. Binary classifiers assigned a positive label to compounds meeting an activity threshold (bacteria: dark or light MIC ≤ 16 μM; mammalian cells: IC50 ≤ 1.5 μM). Ternary classifiers divided compounds into three activity classes: highly active (bacteria: MIC ≤ 16 μM; mammalian: IC_{50} ≤ 1.5 μM), moderately active (bacteria: MIC < 100 μM; mammalian: IC_{50} ≤ 10 μM), and inactive. Two feature sets were evaluated: the 28-dimensional xTB descriptor vector described above (xTB), and an extended set additionally incorporating four complex-level descriptors (fraction buried volume, CN and NN solvent-accessible surface area, logP) and three experimental descriptors (ABD, RET, and DHR values; xTB+complex+exp). During training, all features were standardized using a StandardScaler from scikit-learn.[12] Recursive feature elimination with cross-validation (RFECV) was applied to identify the optimal subset of predictive features, using 5-fold cross-validation with mean absolute error as the scoring metric. Feature

importance was assessed using the mean decrease in impurity (MDI) method intrinsic to the Random Forest algorithm.

Model performance was estimated by 100-run cross-validation using two split strategies applied independently in each run. Random splits used a stratified 70/30 train/test partition. Grouped splits held out 4 **NN** ligands and 2 **CN** ligands at random, placing all complexes containing any held-out ligand in the test set and only complexes sharing no held-out ligand with the test set in the training set; this evaluates generalisation to novel ligand combinations not represented during training. Performance metrics reported are accuracy, balanced accuracy, macro-averaged AUC-ROC, and macro F1, each summarised as mean ± standard deviation across 100 runs. Final models were trained on the full 336-compound dataset using the same hyperparameters and saved for downstream vHTS prediction.

CN ligand extraction

CN ligands for the virtual screen were extracted from MetalCytoToxDB. All SMILES strings in the database were fragmented by splitting on '.' delimiters, canonicalised, and deduplicated. Each unique fragment was then assessed against the same **CN** classification criteria used for **HTS336** (one anionic aromatic carbon, aromatic nitrogen donor, four-atom aromatic coordinating path, no second **NN**-type nitrogen, 10–55 heavy atoms). Fragments containing acidic functional groups (carboxylic acids, sulfonic acids, or phosphonic acids, detected by SMARTS matching) were removed, as were any fragments whose canonical SMILES matched those already present in the **HTS336 CN** ligand set. This produced 187 novel **CN** ligands, each stored with its coordinating atom indices.

Virtual NN ligand generation

A set of virtual phenanthroline-imidazole (**NN**) ligands was generated by computationally applying a Schiff condensation reaction to commercially available aromatic aldehydes from the Enamine catalogue (11,507 compounds). The reaction SMARTS [c:1][CH1;!R](=O)>>[c:1]-c1nc2c3cccnc3c3ncccc3c2[nH]1 converts an aryl aldehyde into the corresponding 2-aryl-imidazole-phenanthroline product. Aldehydes were pre-filtered to retain only those with molecular weight ≤ 250 Da, ≤ 4 rotatable bonds, ≤ 3 rings, exactly one aromatic aldehyde group, no boron atoms, no quaternary or pyridinium nitrogen, no sulfonate or nitro groups, and no acidic functional groups. Reaction products were canonicalised, deduplicated, and verified to contain exactly one valid **NN** bidentate. After removal of any products matching **HTS336 NN** SMILES, the surviving set was reduced to 1,000 maximally diverse ligands by MaxMin selection on Morgan fingerprints (radius 2, 2048 bits, Tanimoto distance metric) computed on the aldehyde precursor structures.

Combinatorial library construction

Two combinatorial libraries were constructed by taking the full cross-product of all **NN** and **CN** ligand feature vectors. The **HTS336**-only library (24 **NN** × 14 **CN** = 336 complexes) was used exclusively for model training and validation. The full virtual library combined the **HTS336** pool with the novel MetalCytoDB **CN** ligands (total 201 **CN**) and novel aldehyde **NN** ligands (total 1,024 **NN**), giving 205,824 complexes. Each complex was assigned one of three labels: **HTS336** (both ligands from **HTS336**), **one_HTS336** (exactly one ligand from **HTS336**), or novel (both ligands novel). The feature vector for each complex concatenated the 14 **NN** descriptors (prefixed A_) with the 14 **CN** descriptors (prefixed B_), giving a 28-dimensional input matrix. See ESI supporting files for structure codes and SMILES.

vHTS prediction, uncertainty quantification, and chemical space visualisation

The trained classifiers were applied to all 205,824 complexes in the full library. For each complex, the Random Forest returned a predicted probability of activity, p(active), and a normalised Shannon entropy of the class probability vector as a measure of prediction uncertainty:

$$H_{norm}(X) = \frac{-\sum_{i=1}^{n} p_i \log_b(p_i)}{\log_b(n)}$$

where H_norm = 0 indicates maximal confidence and H_norm = 1 indicates maximal uncertainty.

To establish an empirical confidence threshold, a 50-run grouped cross-validation was performed on the HTS336 training set. In each run, 4 **NN** ligands and 2 **CN** ligands were withheld, and their associated complexes formed the test fold. Out-of-fold H_norm values were averaged per compound, and the highest threshold at which mean out-of-fold accuracy of retained compounds exceeded 95% was identified (evaluated in steps of 0.01 from 1.0 downward). This yielded thresholds of H_norm < 0.59 (BSub) and H_norm < 0.33 (AT3). Novel compounds in the **vHTS** predictions were filtered to retain only those below the relevant threshold.

Principal component analysis was applied to the 28-dimensional feature matrix to produce a 2D embedding for visualisation of chemical space coverage.

Applicability domain filter

An applicability domain (AD) filter was applied to ensure that virtual screen predictions fell within the chemical space represented by the training data. The 28-dimensional xTB feature vectors for all complexes were standardised using a StandardScaler fitted to the **HTS336** training set. k-nearest neighbour distances

(k = 5, Euclidean metric) from each **HTS336** complex to its five nearest neighbours within the training set were computed via leave-one-out, and the 99th percentile of these distances was taken as the AD threshold. Each complex in the full library was then assigned its mean distance to its 5 nearest neighbours in the scaled training set; complexes exceeding the threshold were flagged as outside the AD and excluded from downstream analysis.

Fragment enrichment analysis

Fragment-level SAR was extracted from the AD+UQ-filtered novel complex pools by grouping complexes independently by CN identity and NN identity. For each unique fragment appearing in at least 3 complexes, the following statistics were computed: total number of complexes (n), number of predicted hits (p(active) > 0.5), hit rate (hits/n), mean predicted activity, maximum predicted activity, and mean model confidence (1 - h_norm). Statistics were computed separately for the BSub and AT3 filtered pools and then outer-merged, with fragments absent from one pool assigned zero values for that target.

$$W = \frac{\hat{p} + \frac{z^2}{2n} - z\sqrt{\frac{\hat{p}(1-\hat{p})}{n} + \frac{z^2}{4n^2}}}{1 + \frac{z^2}{n}}$$

Fragments were ranked and shortlisted using the Wilson score lower bound of the 95% confidence interval for the hit rate, which penalises fragments with inflated apparent hit rates from small sample sizes without requiring a tuning parameter. Three shortlists of fragments were produced: BSub-selective (highest BSub Wilson score, mean predicted activity < 0.30 in AT3, at least 3 observed hits in BSub); AT3-selective (highest AT3 Wilson score, mean predicted activity < 0.30 in BSub, at least 3 observed hits in AT3); and broad-spectrum (highest combined BSub + AT3 Wilson score, at least 3 complexes in both pools).

Synthesis and characterisation

[Ir(**CN01**)$_2$(**NN03**)]Cl

The 'General procedure for the preparative synthesis of iridium complexes' was followed, combining **NN03** (0.065 mmol, 20.89 mg, 1 eqv) with **CN01** (0.0325 mmol, 34.84 mg, 0.5 eqv). The product was obtained in 89% yield (50 mg). ^{1}H NMR (400 MHz, DMSO) δ 15.35 (s, 1H), 9.57 (d, *J* = 8.3 Hz, 1H), 9.18 (d, *J* = 8.2 Hz, 1H), 8.64 (d, *J* = 8.2 Hz, 2H), 8.28 (d, *J* = 8.2 Hz, 2H), 8.22 – 8.05 (m, 6H), 7.97 (d, *J* = 7.8 Hz, 2H), 7.89 (t, *J* = 7.8 Hz, 2H), 7.53 (d, *J* = 5.8 Hz, 2H), 7.08 (t, *J* = 7.5 Hz, 2H), 6.99 (dt, *J* = 14.8, 7.6 Hz, 4H), 6.30 (d, *J* = 7.5 Hz, 2H).

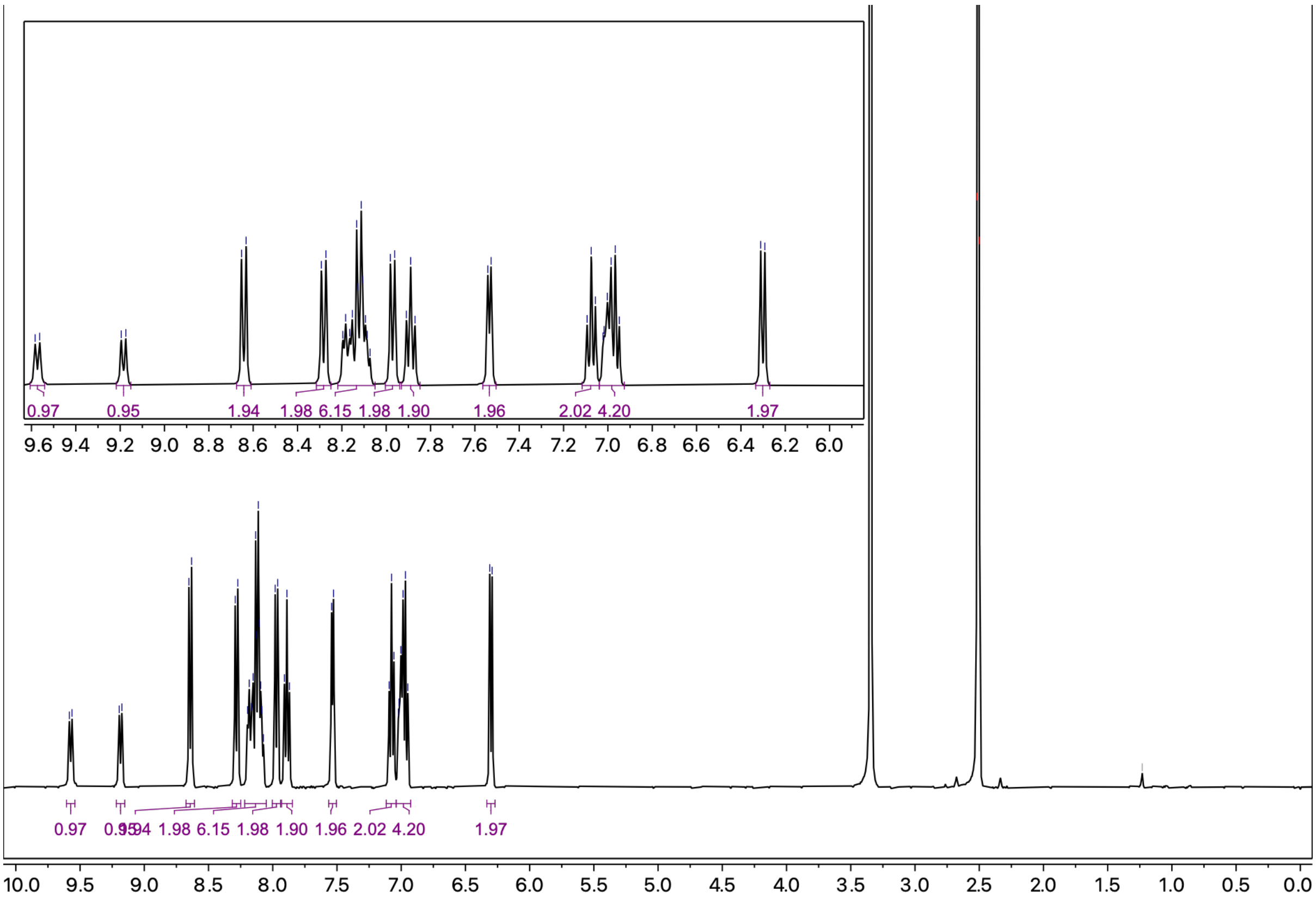

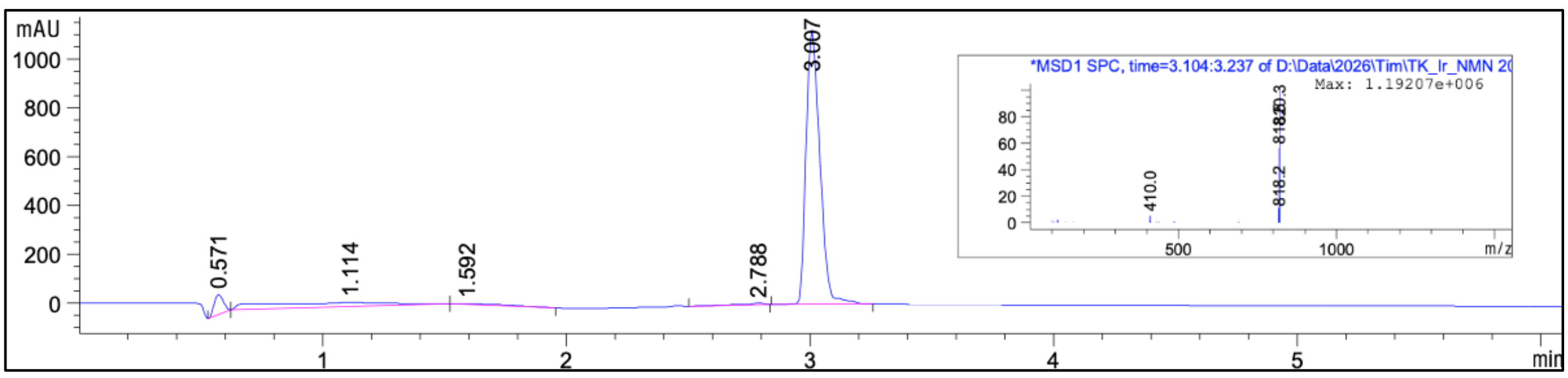

[Ir(**CN01**)$_2$(**NN08**)]Cl

The 'General procedure for the preparative synthesis of iridium complexes' was followed, combining **NN08** (0.065 mmol, 22.52 mg, 1 eqv) with **CN01** (0.0325 mmol, 34.84 mg, 0.5 eqv). The product was obtained in 73% yield (42 mg). ^{1}H NMR (400 MHz, DMSO) δ 9.31 (s, 2H), 9.06 (d, *J* = 8.4 Hz, 1H), 8.29 (d, *J* = 8.2 Hz, 2H), 8.20 (t, *J* = 7.7 Hz, 4H), 8.12 (dq, *J* = 7.5, 4.0 Hz, 3H), 7.98 (d, *J* = 7.8 Hz, 2H), 7.90 (t, *J* = 7.9 Hz, 2H), 7.80 (t, *J* = 7.8 Hz, 2H), 7.70 (dq, *J* = 10.3, 6.2 Hz, 2H), 7.55 (d, *J* = 5.8 Hz, 2H), 7.08 (t, *J* = 7.5 Hz, 2H), 6.99 (dt, *J* = 14.7, 7.0 Hz, 4H), 6.32 (d, *J* = 7.5 Hz, 2H).

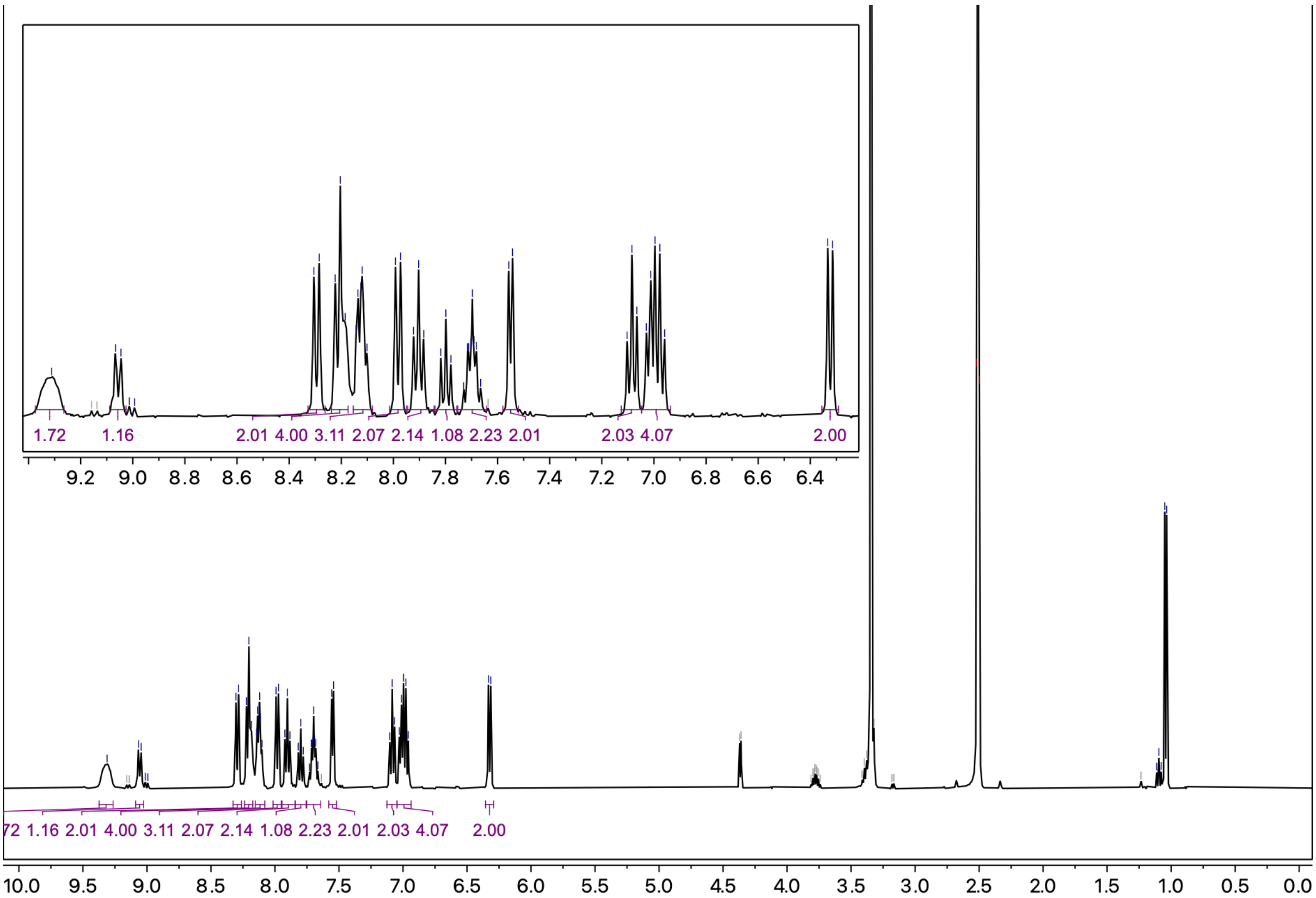

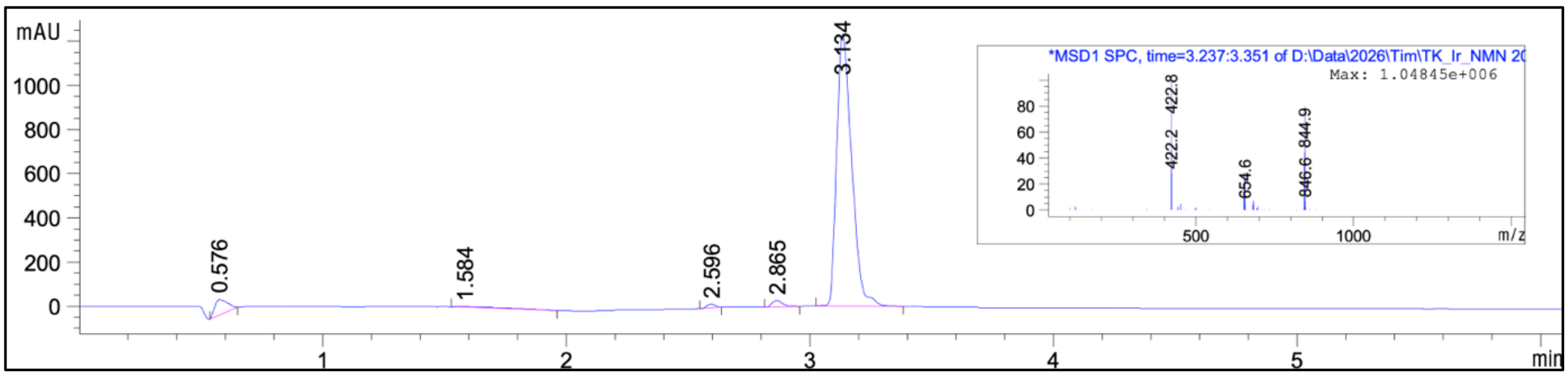

[Ir(**CN06**)$_2$(**NN07**)]Cl

The 'General procedure for the preparative synthesis of iridium complexes' was followed, combining **NN07** (0.065 mmol, 25.58 mg, 1 eqv) with **CN06** (0.0325 mmol, 41.35 mg, 0.5 eqv). The product was obtained in 84% yield (56 mg). ^{1}H NMR (400 MHz, DMSO) δ 9.07 – 9.00 (m, 2H), 8.42 (d, *J* = 8.0 Hz, 2H), 8.36 (d, *J* = 7.8 Hz, 2H), 8.04 (dq, *J* = 7.2, 4.7 Hz, 6H), 7.89 (hept, *J* = 5.0 Hz, 4H), 7.55 (d, *J* = 7.8 Hz, 2H), 7.48 – 7.38 (m, 4H), 7.19 (t, *J* = 7.7 Hz, 2H), 6.98 (t, *J* = 7.4 Hz, 2H), 6.31 (d, *J* = 7.6 Hz, 2H), 3.53 (s, 2H), 2.38 (s, 4H), 1.54 (s, 4H), 1.42 (s, 2H).

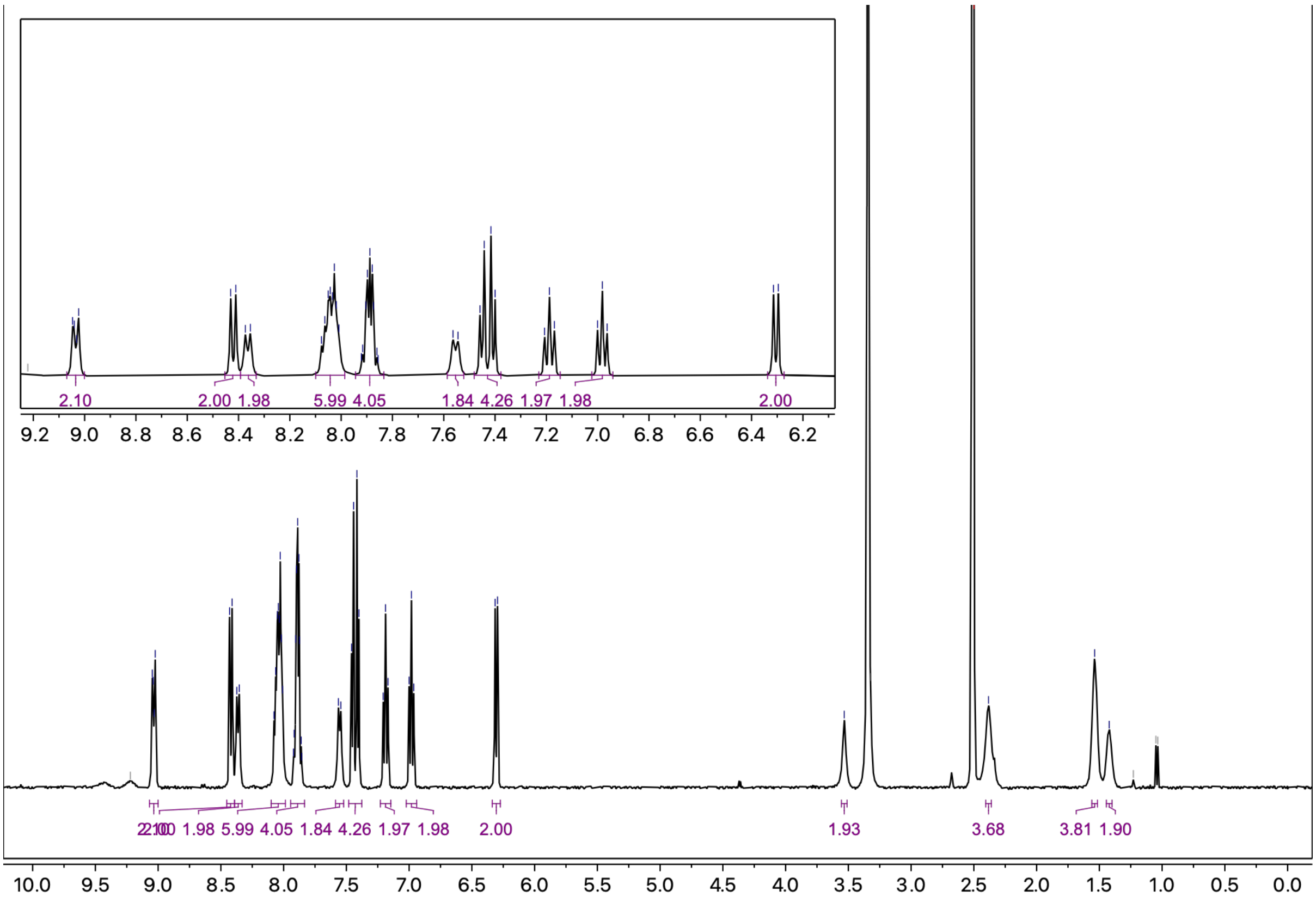

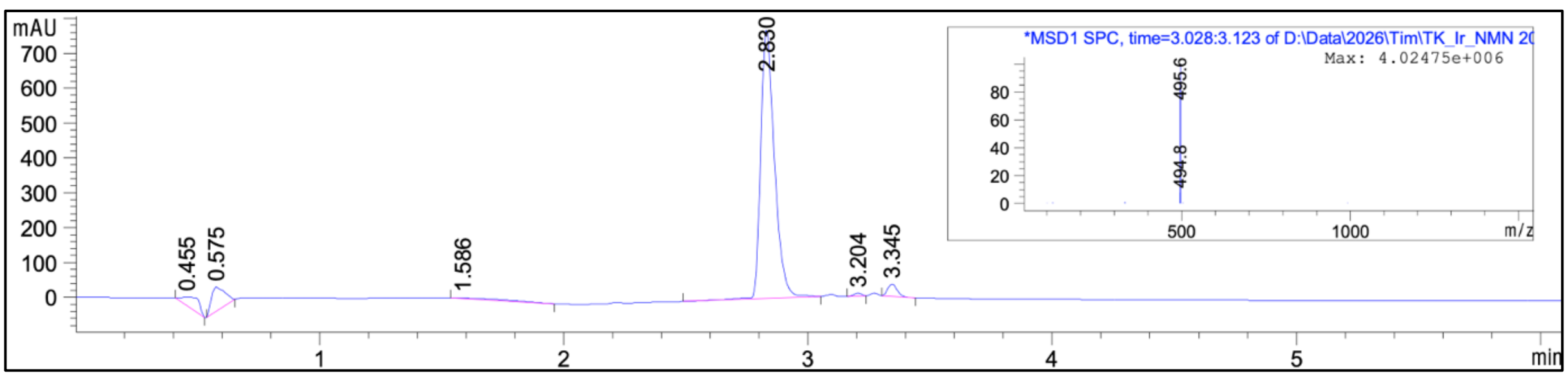

[Ir(**CN07**)$_2$(**NN11**)]Cl

The 'General procedure for the preparative synthesis of iridium complexes' was followed, combining **NN11** (0.065 mmol, 22.64 mg, 1 eqv) with **CN07** (0.0325 mmol, 41.35 mg, 0.5 eqv). The product was obtained in 73% yield (47 mg). ^{1}H NMR (400 MHz, DMSO) δ 14.90 (s, 1H), 9.27 (s, 1H), 9.11 (s, 1H), 9.07 – 8.98 (m, 3H), 8.71 (dd, *J* = 8.9, 1.9 Hz, 1H), 8.64 (d, *J* = 8.9 Hz, 2H), 8.52 (d, *J* = 8.8 Hz, 2H), 8.45 (d, *J* = 4.5 Hz, 2H), 8.38 (d, *J* = 7.9 Hz, 2H), 8.28 (d, *J* = 9.0 Hz, 1H), 8.13 (s, 2H), 7.82 (d, *J* = 8.1 Hz, 2H), 7.26 (d, *J* = 7.2 Hz, 2H), 7.25 – 7.15 (m, 4H), 6.89 (t, *J* = 7.5 Hz, 4H), 6.54 (d, *J* = 7.6 Hz, 2H), .

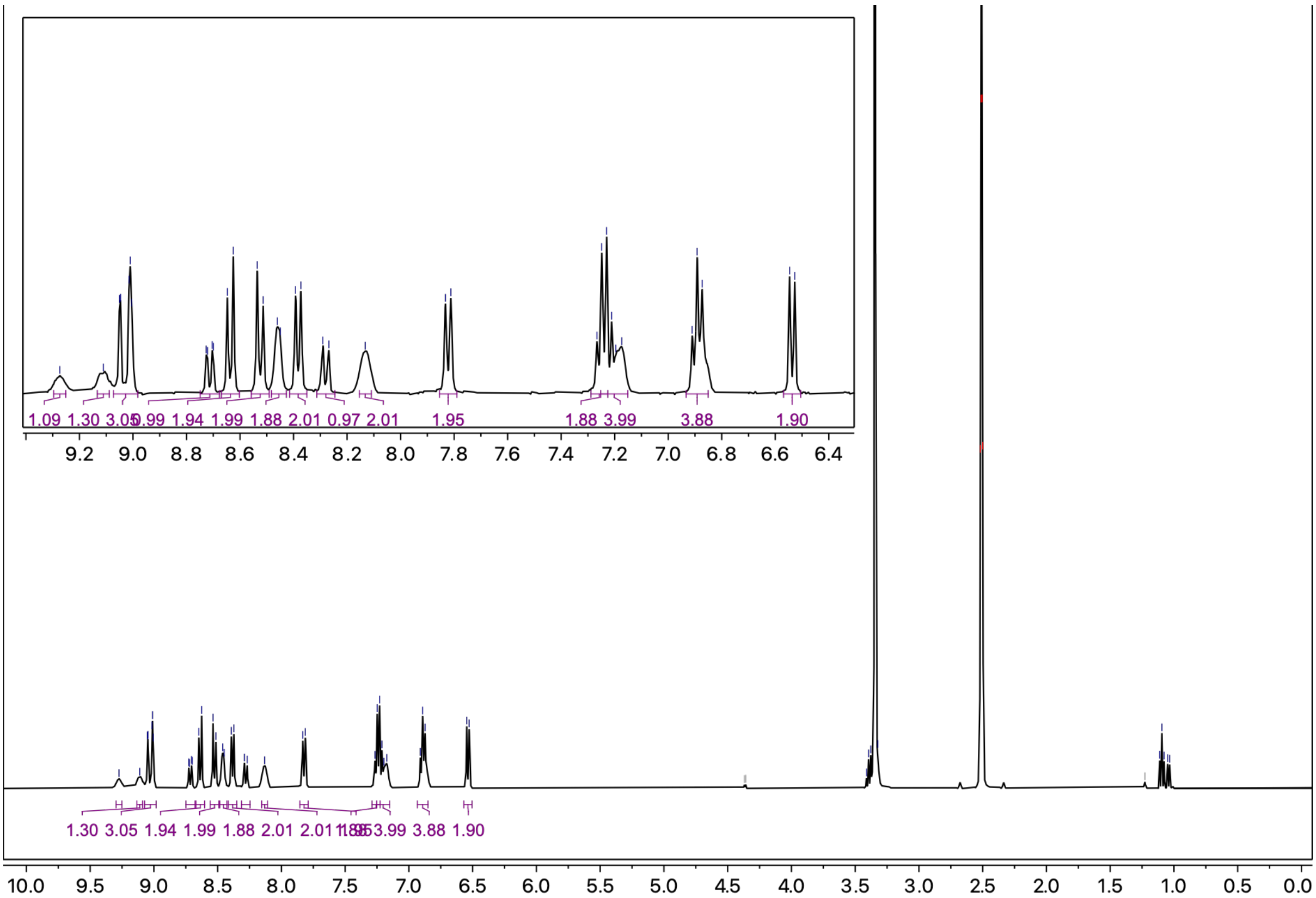

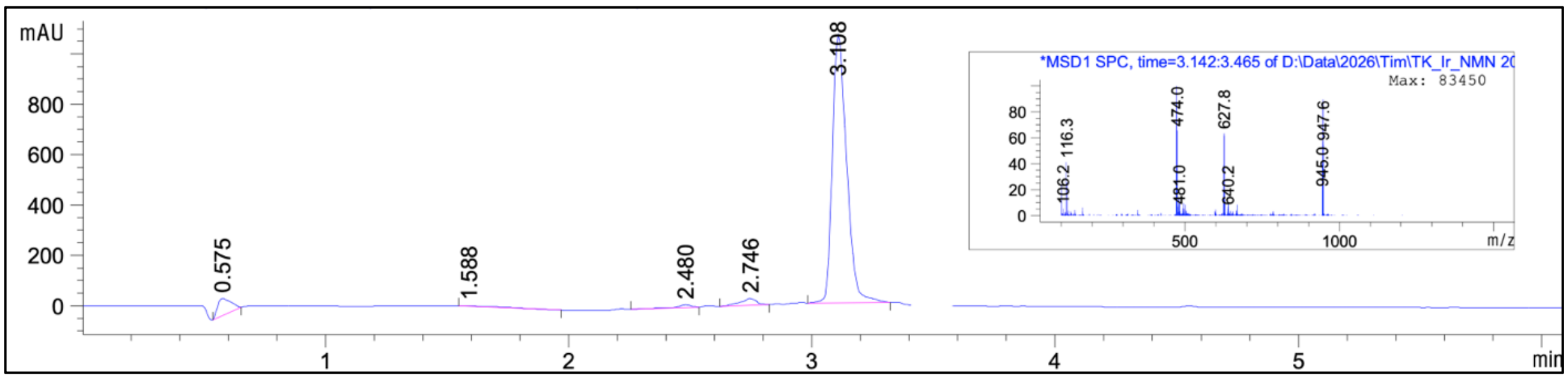

[Ir(**CN08**)$_2$(**NN18**)]Cl

The 'General procedure for the preparative synthesis of iridium complexes' was followed, combining **NN18** (0.065 mmol, 21.80 mg, 1 eqv) with **CN08** (0.0325 mmol, 41.74 mg, 0.5 eqv). The product was obtained in 93% yield (59 mg). ^{1}H NMR (400 MHz, DMSO) δ 9.26 (s, 2H), 8.41 (s, 1H), 8.34 (d, *J* = 5.0 Hz, 2H), 8.11 (d, *J* = 7.7 Hz, 4H), 8.09 – 7.97 (m, 2H), 7.75 (d, *J* = 8.3 Hz, 2H), 7.55 – 7.42 (m, 2H), 7.15 (dt, *J* = 20.8, 7.7 Hz, 4H), 6.91 (t, *J* = 7.5 Hz, 2H), 6.77 (t, *J* = 7.8 Hz, 2H), 6.39 (d, *J* = 7.6 Hz, 2H), 5.38 (d, *J* = 8.3 Hz, 2H), 4.38 (s, 6H).

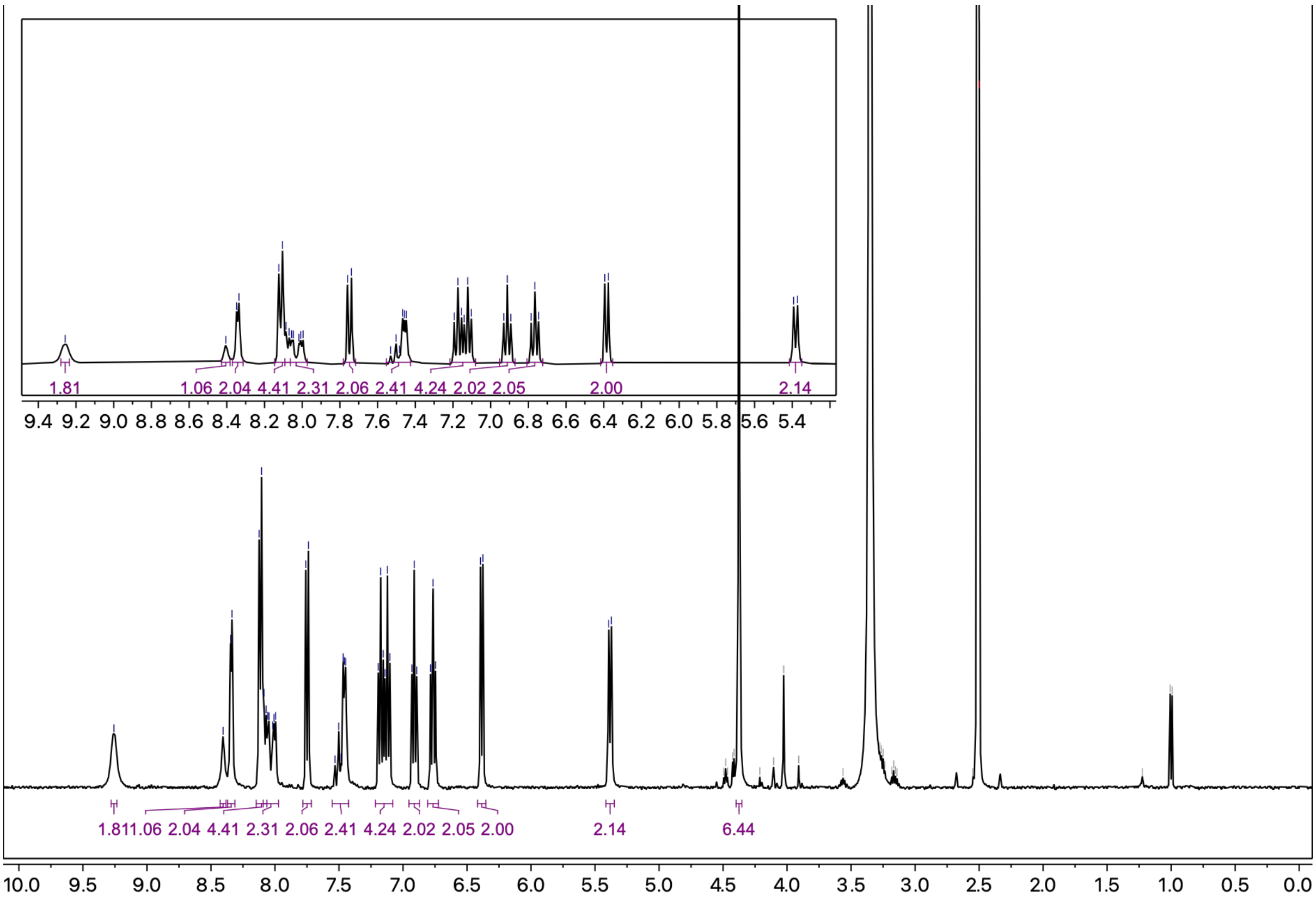

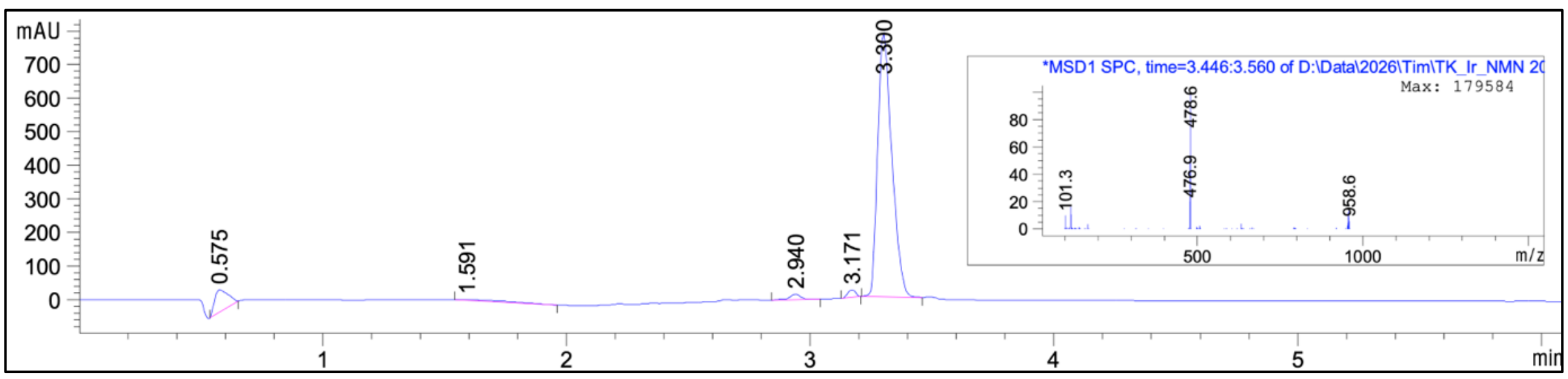

[Ir(**CN10**)$_2$(**NN14**)]Cl

The 'General procedure for the preparative synthesis of iridium complexes' was followed, combining **NN14** (0.065 mmol, 21.86 mg, 1 eqv) with **CN10** (0.0325 mmol, 46.04 mg, 0.5 eqv). The product was obtained in 74% yield (50 mg). ^{1}H NMR (400 MHz, DMSO) δ 15.03 (s, 1H), 9.53 (d, *J* = 8.4 Hz, 1H), 9.29 (d, *J* = 8.3 Hz, 1H), 9.07 (s, 1H), 8.59 (d, *J* = 8.8 Hz, 1H), 8.43 (dd, *J* = 12.9, 5.1 Hz, 2H), 8.22 (dt, *J* = 8.8, 5.2 Hz, 3H), 8.08 (dd, *J* = 20.1, 8.5 Hz, 5H), 7.28 – 7.19 (m, 2H), 6.93 (q, *J* = 8.3 Hz, 2H), 6.82 (dd, *J* = 8.5, 2.4 Hz, 2H), 5.84 – 5.71 (m, 4H), 4.39 (s, 3H), 3.53 (s, 6H).

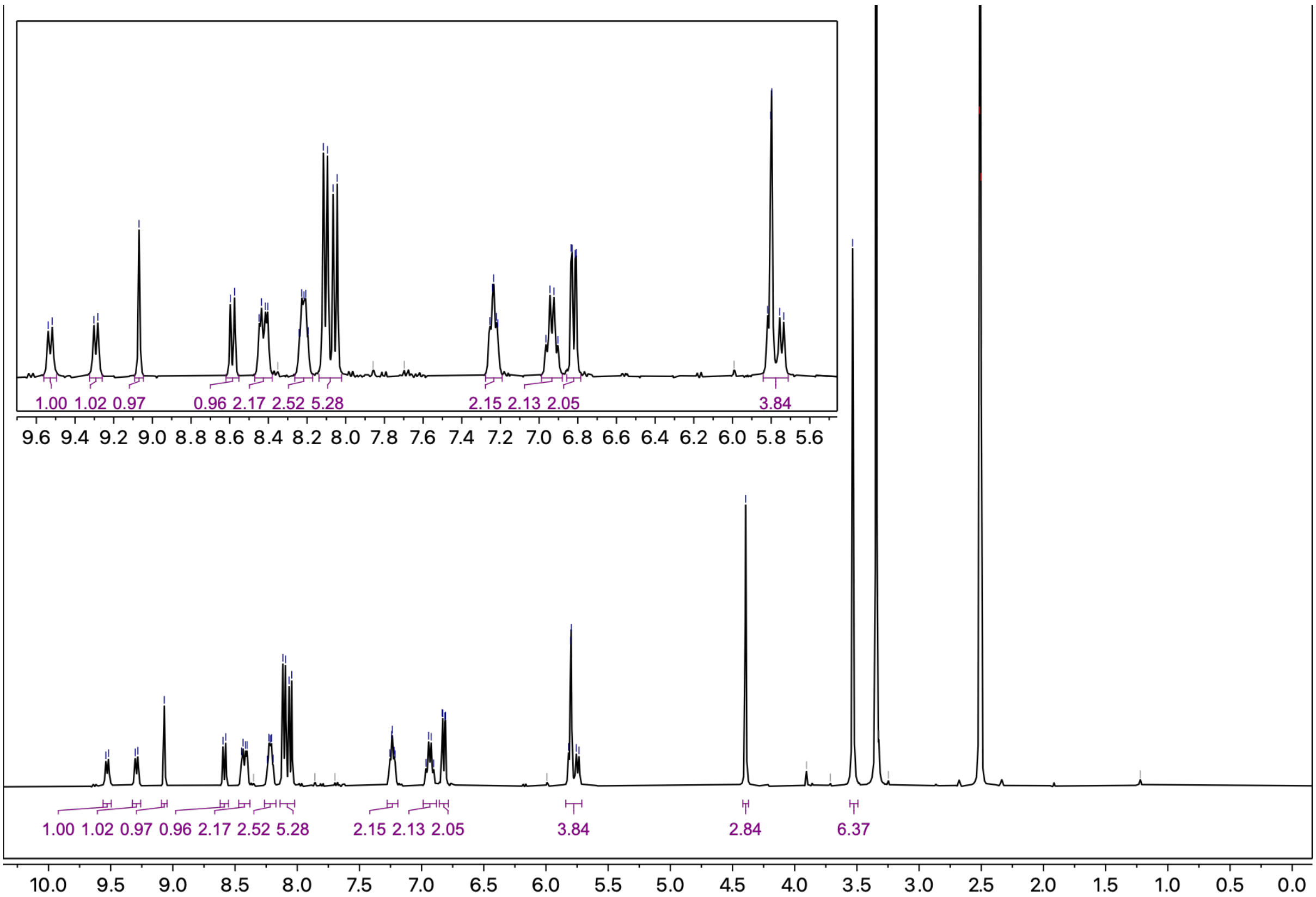

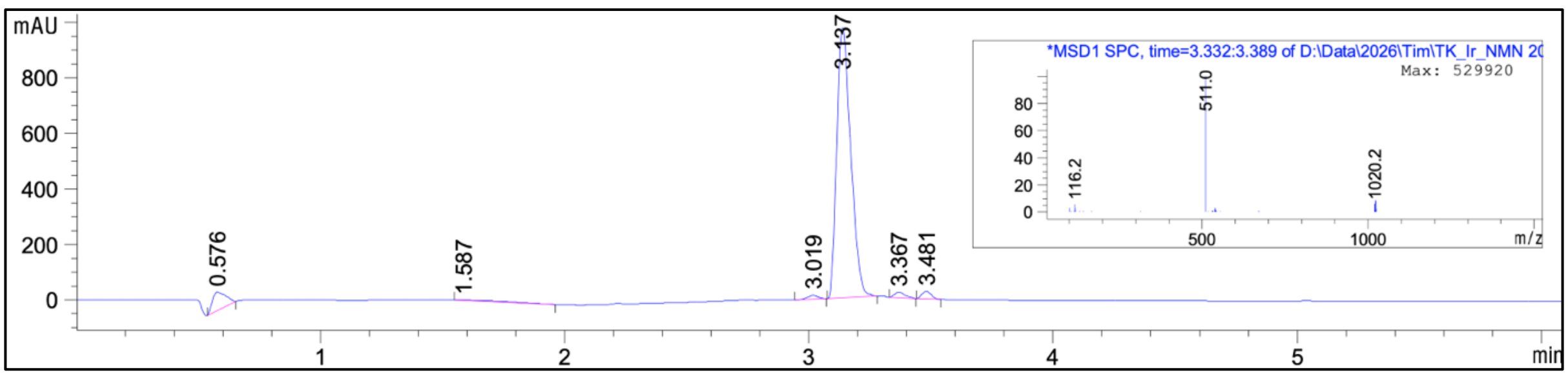

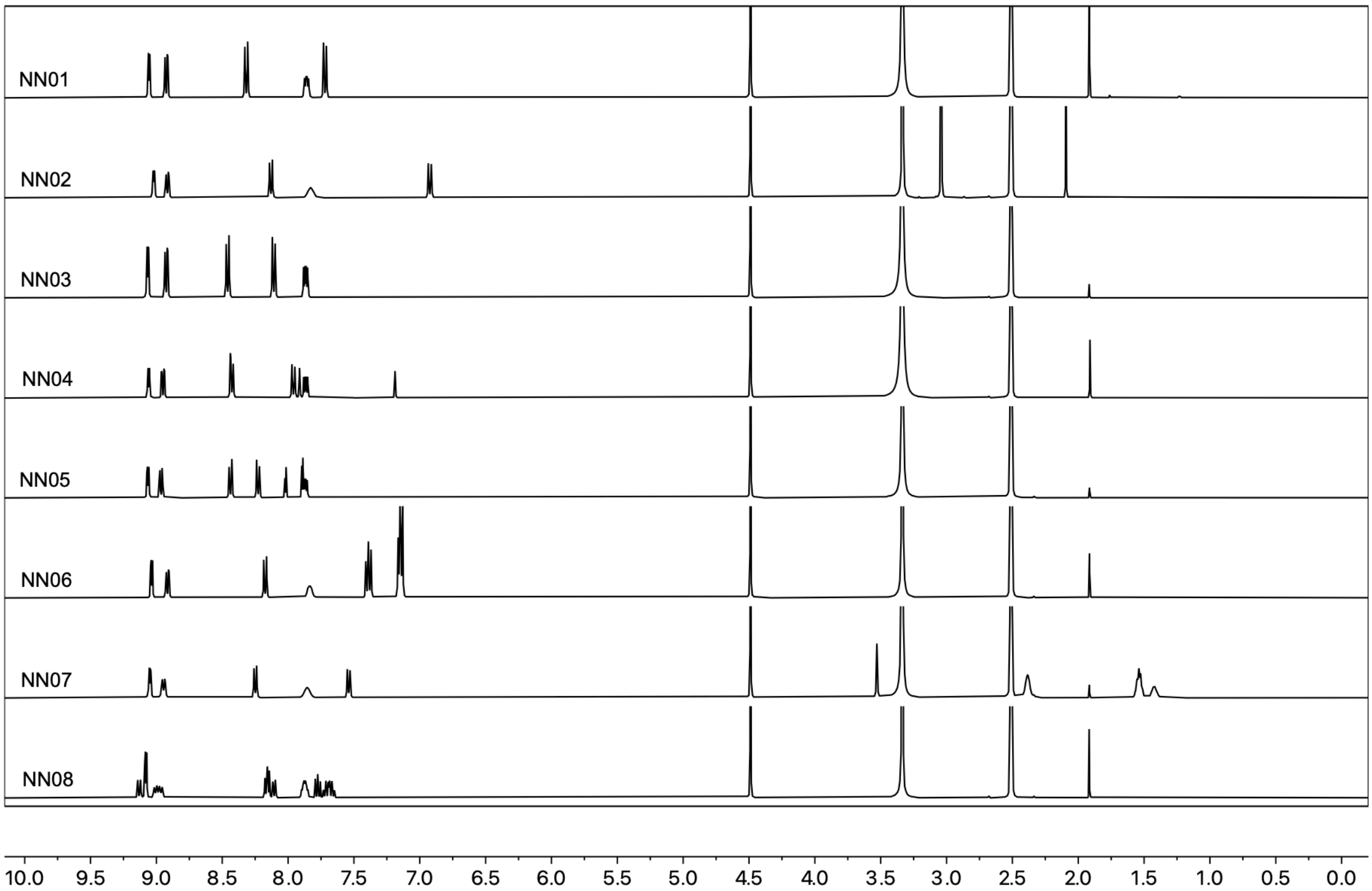

Figure S20: ^{1}H NMR spectra for **NN01-08** fragments in DMSO-d_6.

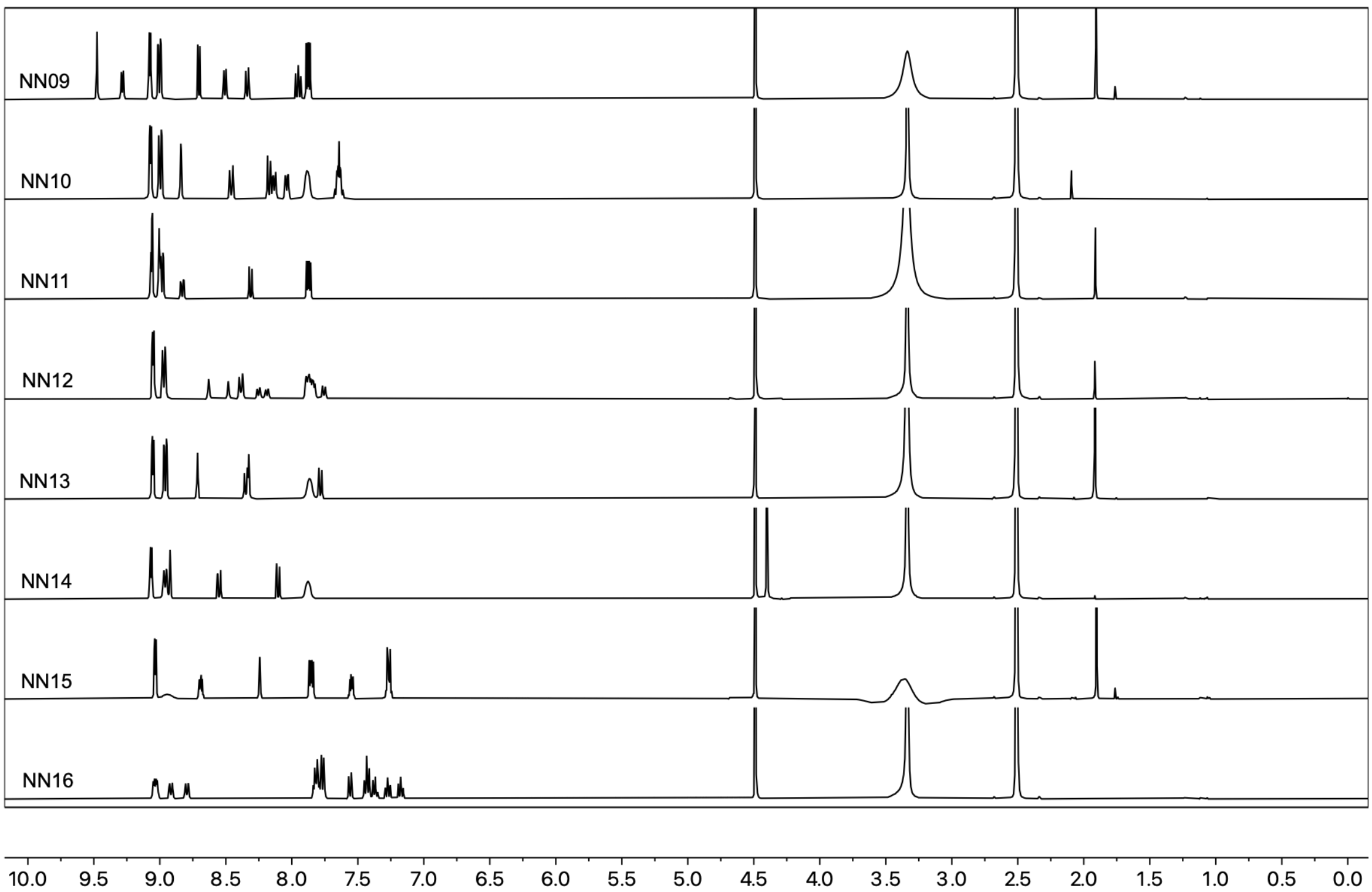

Figure S21: ^{1}H NMR spectra for **NN09-16** fragments in DMSO-d_6.

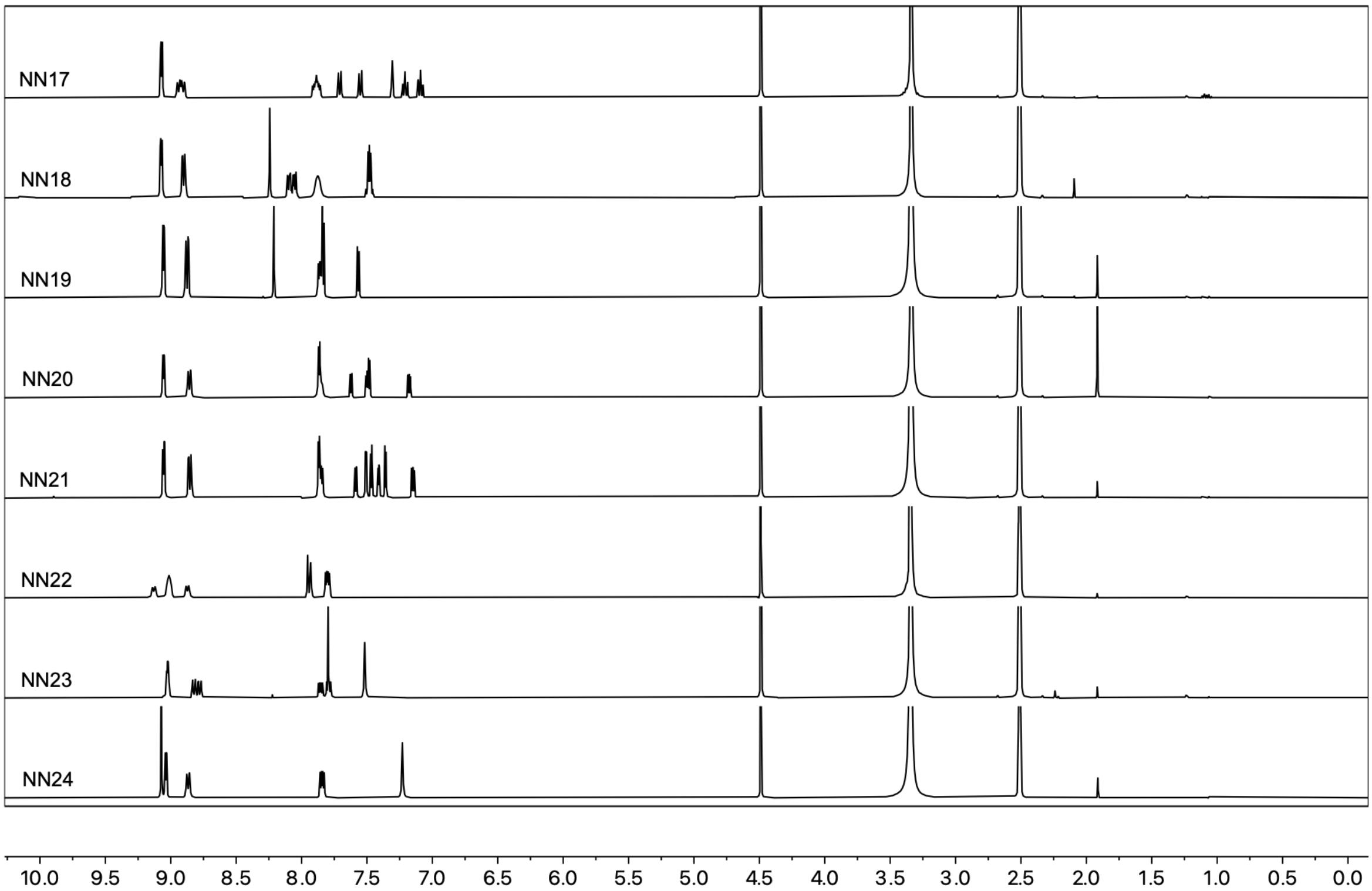

Figure S22: ^{1}H NMR spectra for **NN17-24** fragments in DMSO-d_6.

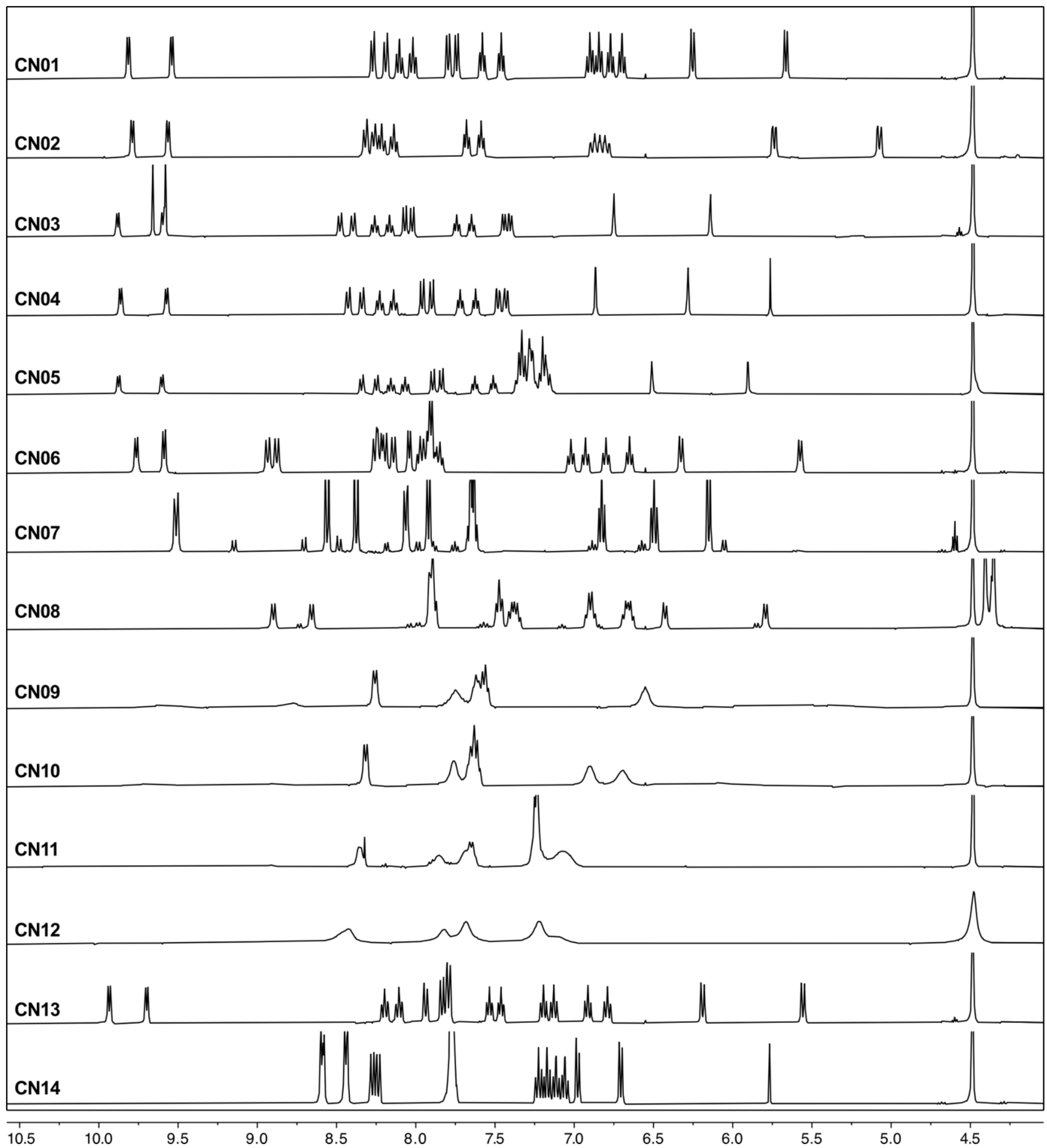

Figure S23: ^{1}H NMR spectra for [Ir(**CN**)(μ_2-Cl]$_2$ with **CN01-14** in DMSO-d_6.

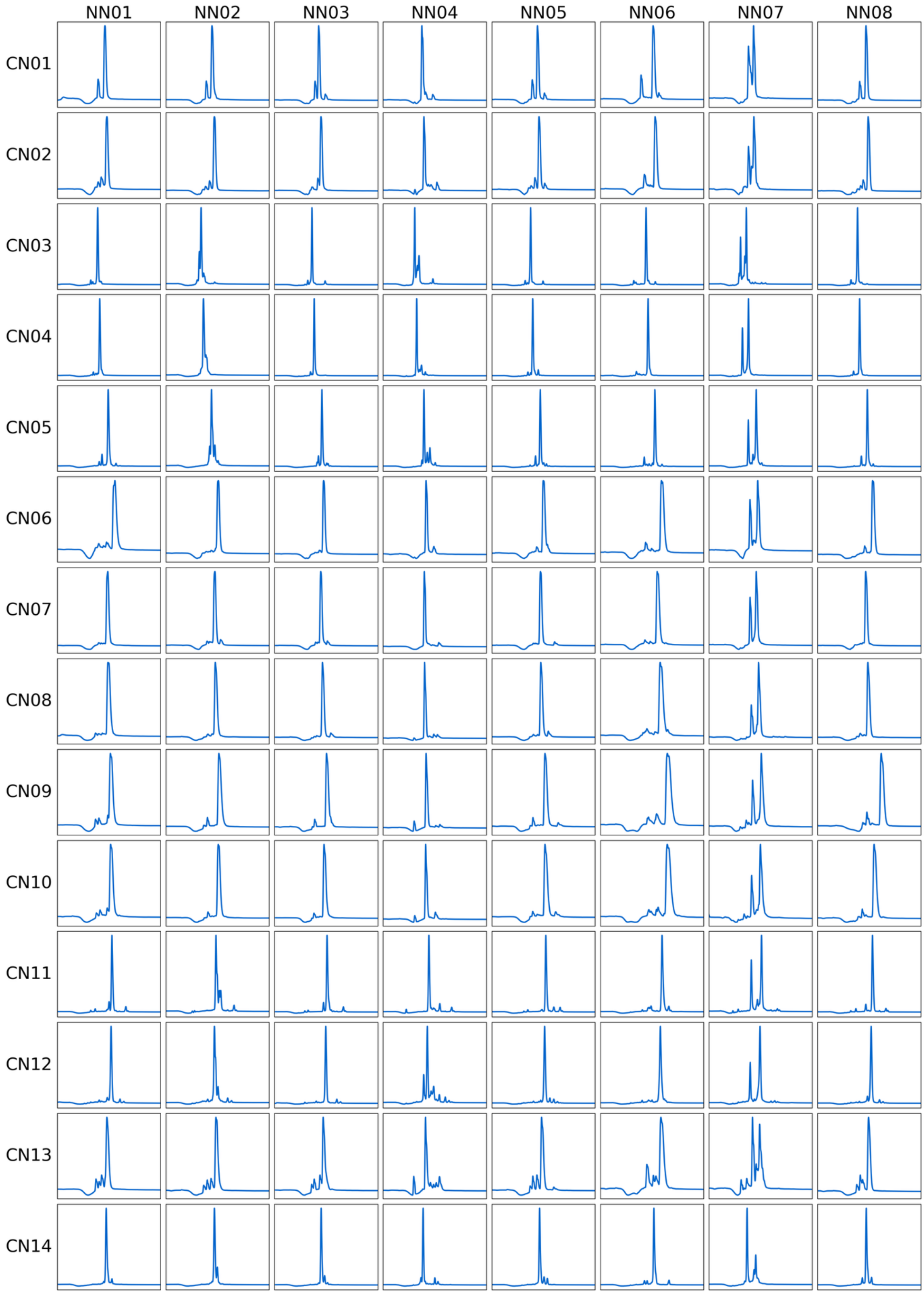

Figure S24: LCMS traces for [Ir(**CN**)$_2$(**NN**)]$^+$ complexes with **NN01-8** and **CN01-14**. UV trace at 254 nm is shown.

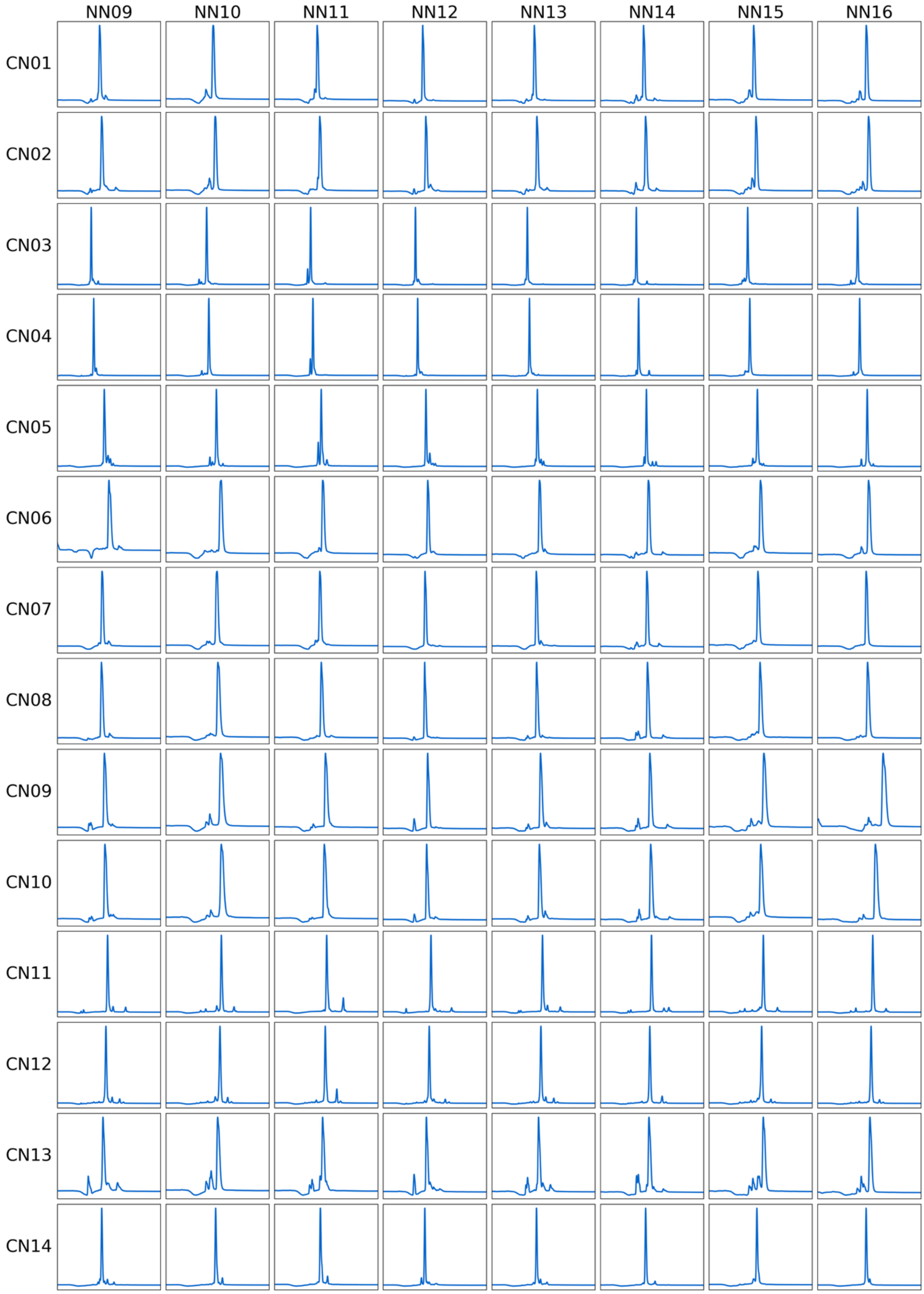

Figure S25: LCMS traces for [Ir(**CN**)$_2$(**NN**)]$^+$ complexes with **NN09-16** and **CN01-14**. UV trace at 254 nm is shown.

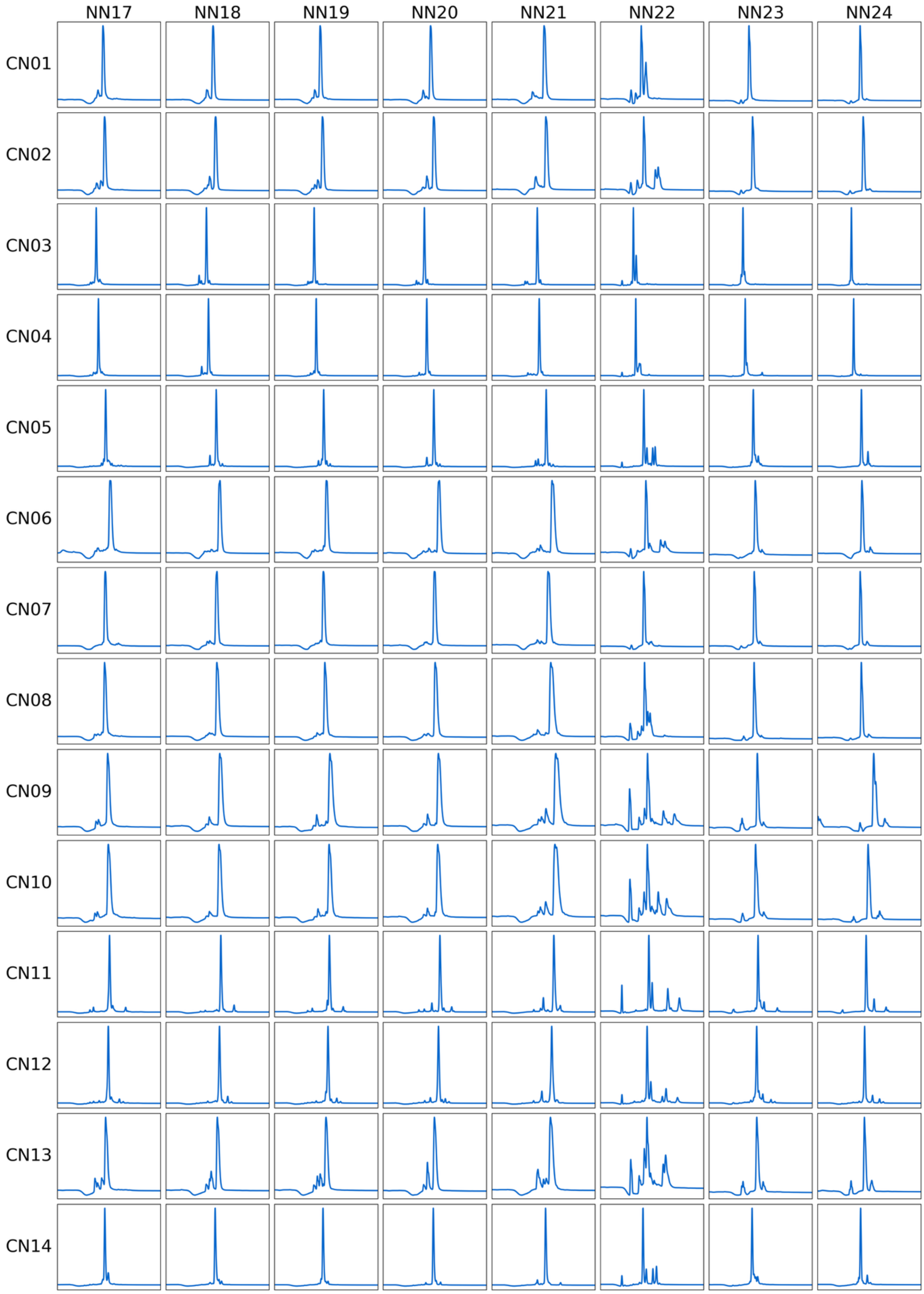

Figure S26: LCMS traces for [Ir(**CN**)$_2$(**NN**)]$^+$ complexes with **NN17-24** and **CN01-14**. UV trace at 254 nm is shown.

Additional Supplementary Figures

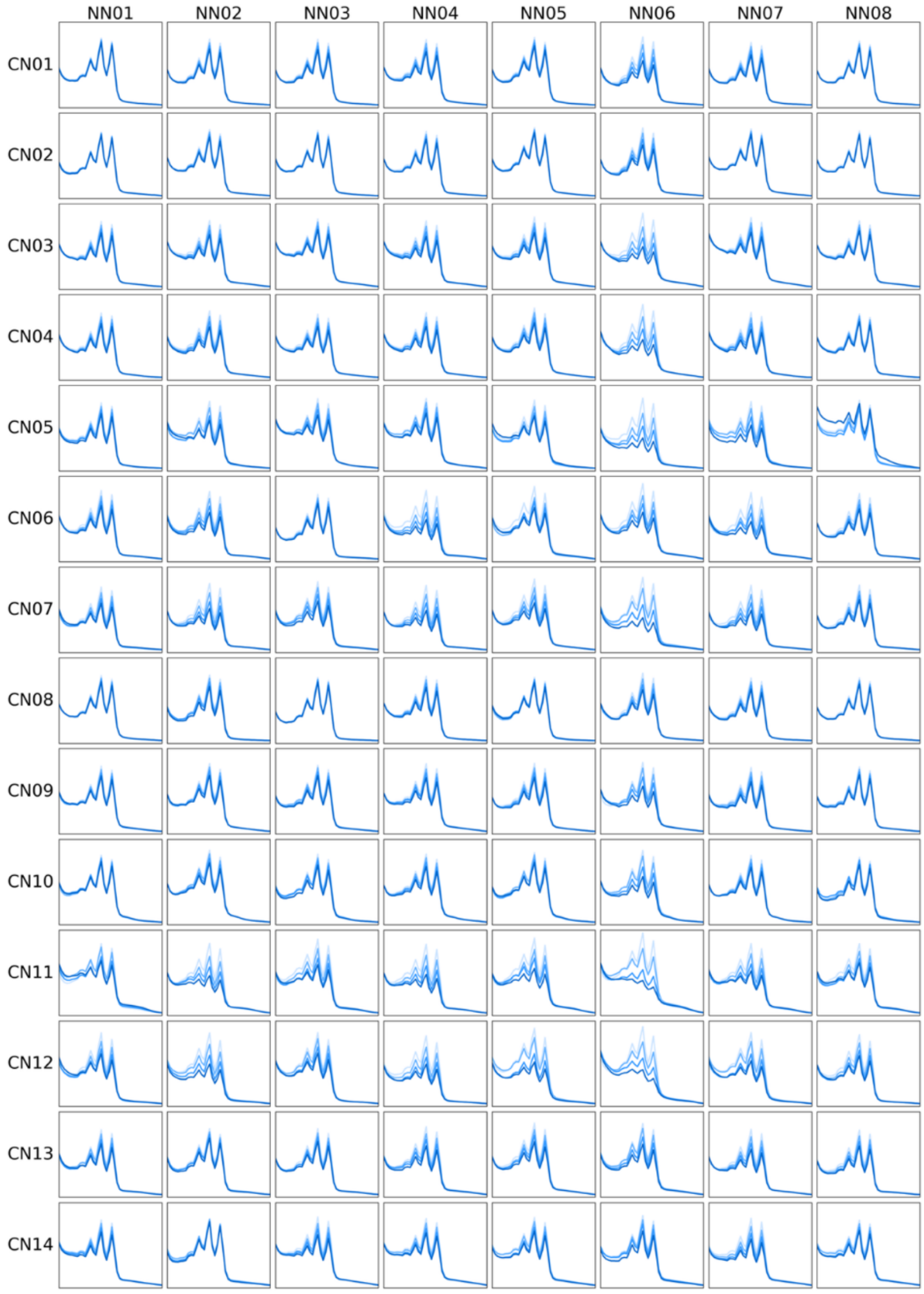

Figure S27: ABDA spectrum curves for complexes containing **CN01**-**14** and **NN01**-**8** for 0-3 minutes of irradiation (blue light, 470 nm, 8 mW). Complex concentration is 16 µM, ABDA concentration 200 µM. All graphs share the same axes (x, 300-500 nm absorbance; y, 0-1.5 absorbance units).

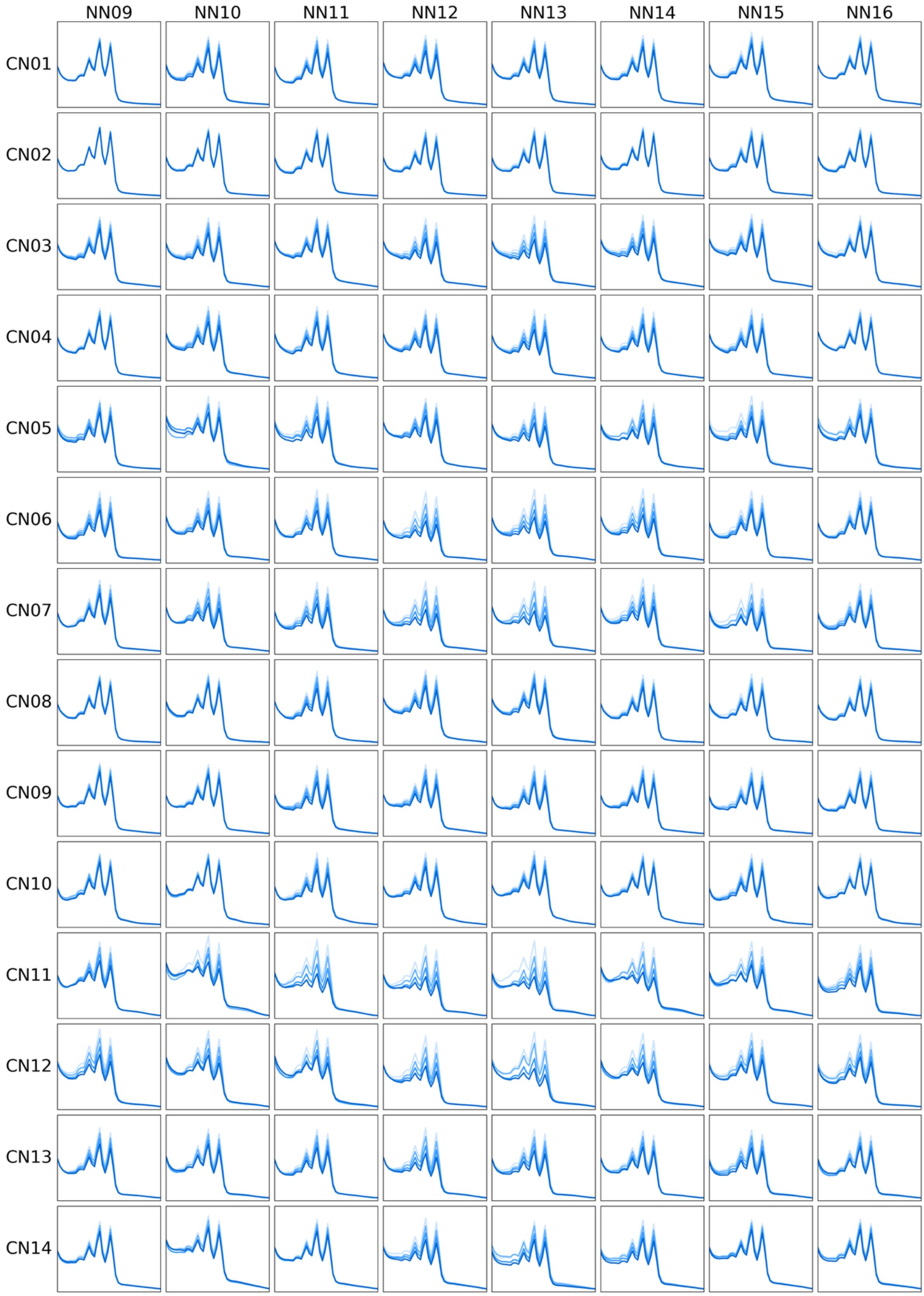

Figure S28: ABDA spectrum curves for complexes containing **CN01-14** and **NN09-16** for 0-3 minutes of irradiation (blue light, 470 nm, 8 mW). Complex concentration is 16 µM, ABDA concentration 200 µM. All graphs share the same axes (x, 300-500 nm absorbance; y, 0-1.5 absorbance units).

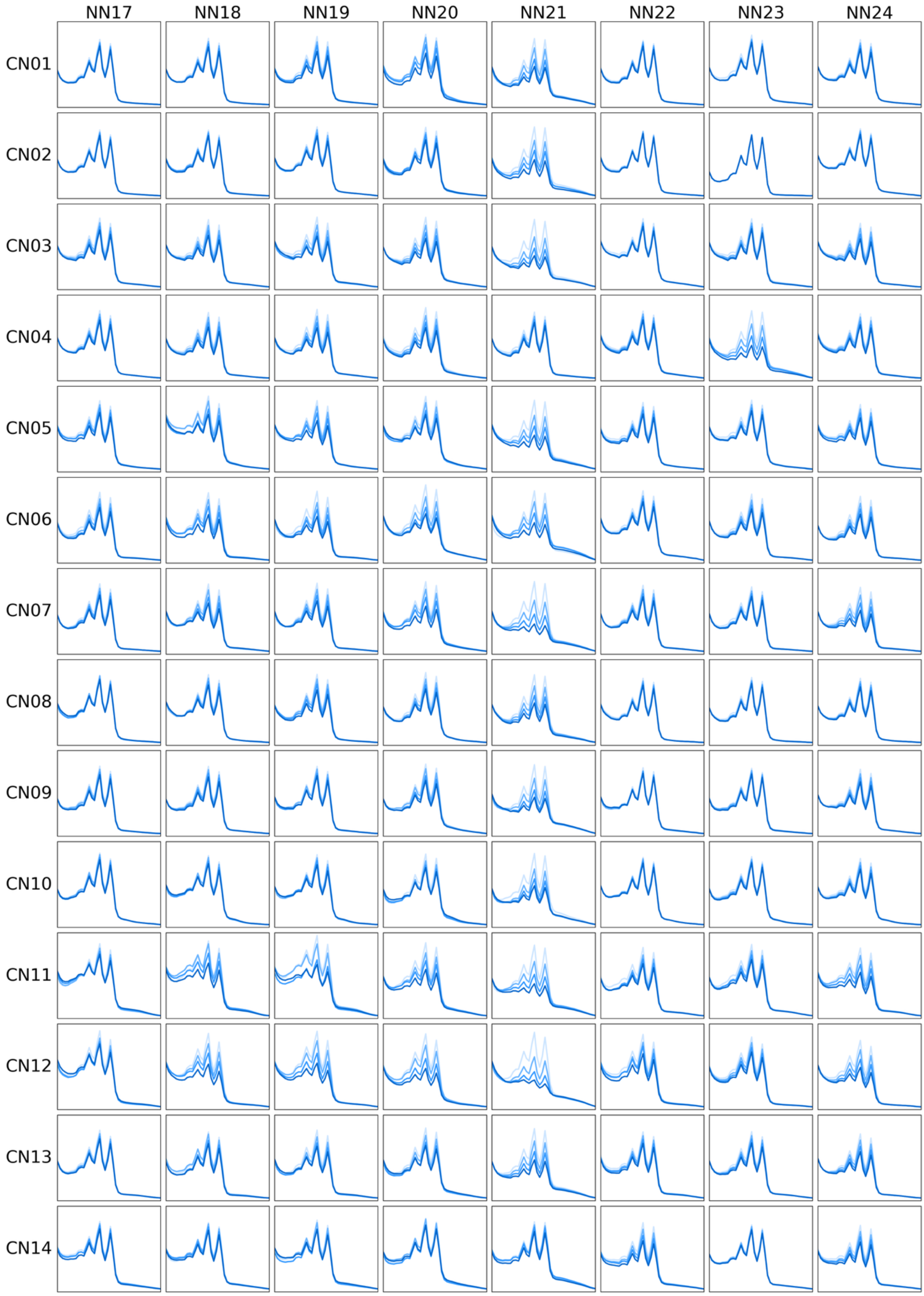

Figure S29: ABDA spectrum curves for complexes containing **CN01-14** and **NN17-24** for 0-3 minutes of irradiation (blue light, 470 nm, 8 mW). Complex concentration is 16 µM, ABDA concentration 200 µM. All graphs share the same axes (x, 300-500 nm absorbance; y, 0-1.5 absorbance units).

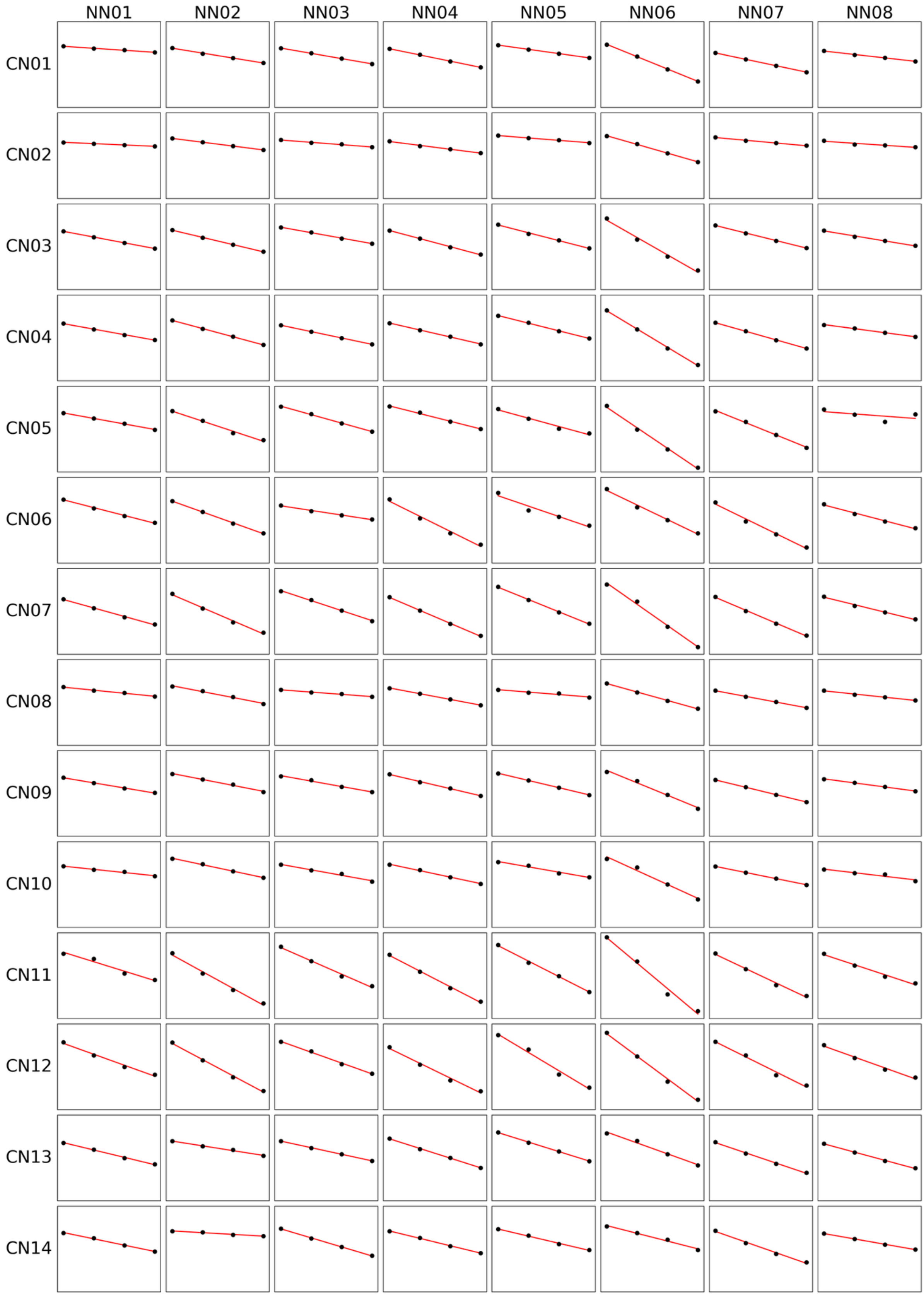

Figure S30: ABDA absorbance at 380 nm for complexes containing **CN01**-**14** and **NN01**-**8** plotted against 0-3 minutes of irradiation (blue light, 470 nm, 8 mW). Complex concentration is 16 µM, ABDA concentration 200 µM. All graphs share the same axes (x, 0-3 minutes; y, 0.5-1.5 absorbance units).

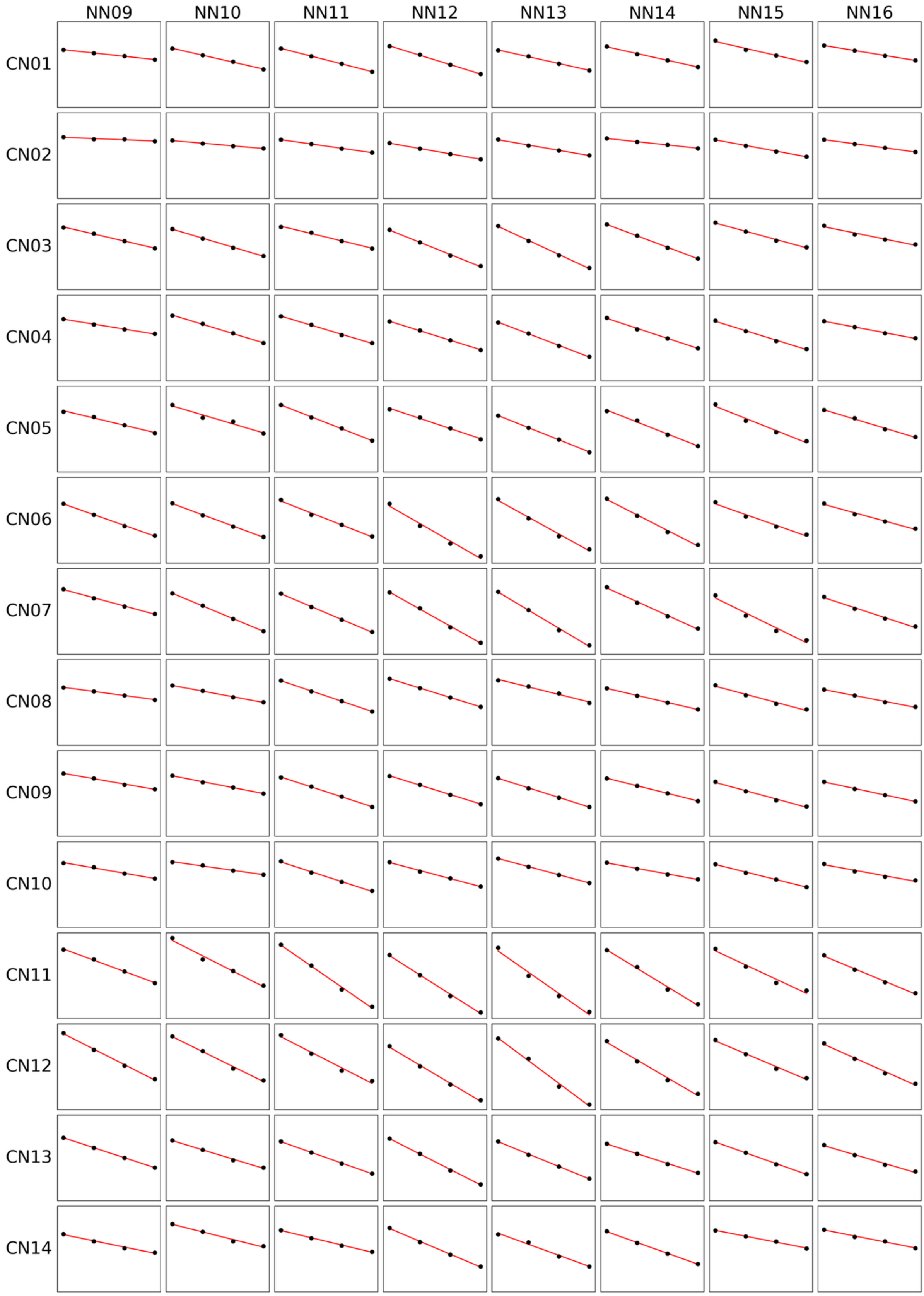

Figure S31: ABDA absorbance at 380 nm for complexes containing **CN01**-**14** and **NN09**-**16** plotted against 0-3 minutes of irradiation (blue light, 470 nm, 8 mW). Complex concentration is 16 µM, ABDA concentration 200 µM. All graphs share the same axes (x, 0-3 minutes; y, 0.5-1.5 absorbance units).

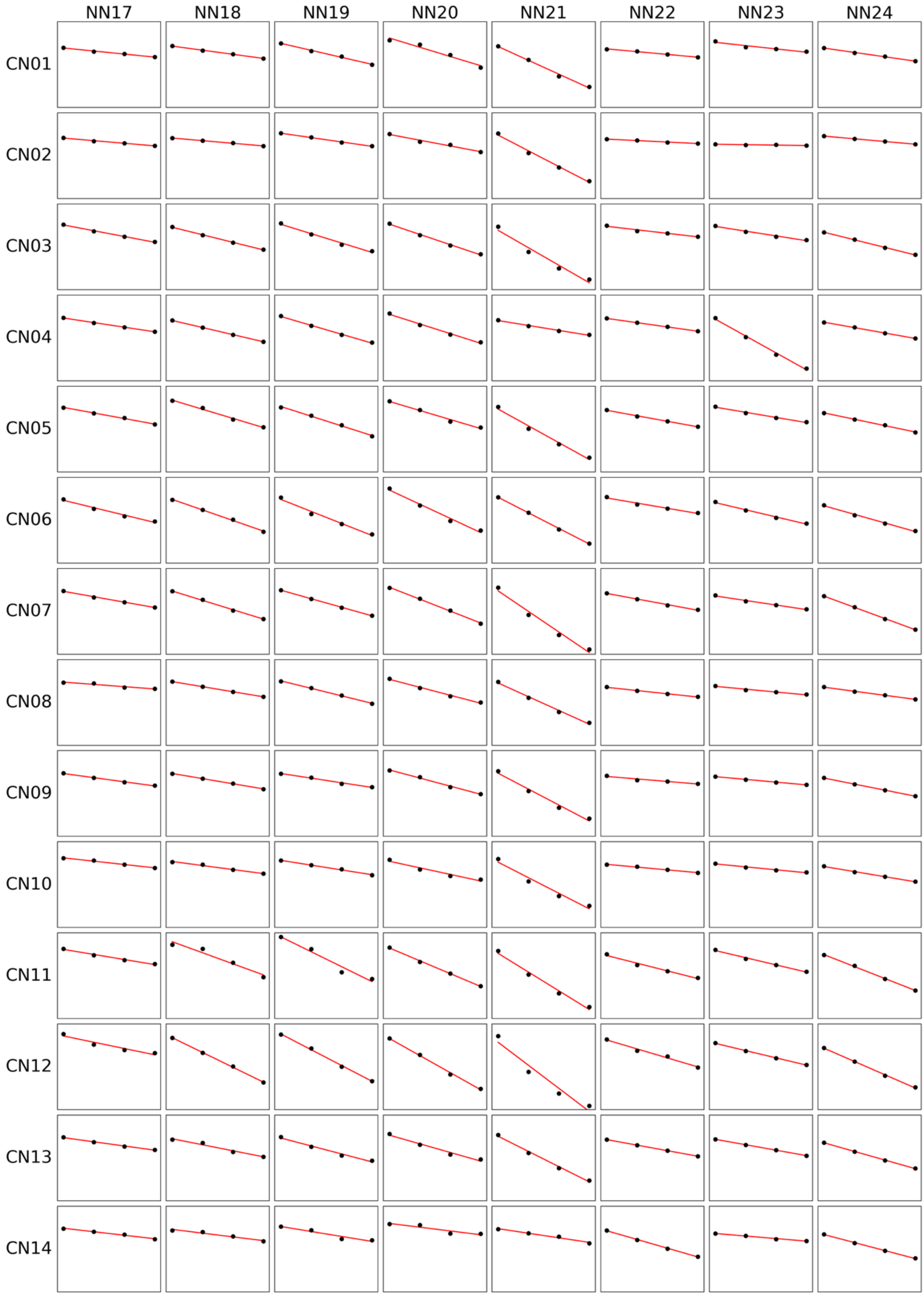

Figure S32: ABDA absorbance at 380 nm for complexes containing **CN01**-**14** and **NN17**-**24** plotted against 0-3 minutes of irradiation (blue light, 470 nm, 8 mW). Complex concentration is 16 μM, ABDA concentration 200 μM. All graphs share the same axes (x, 0-3 minutes; y, 0.5-1.5 absorbance units).

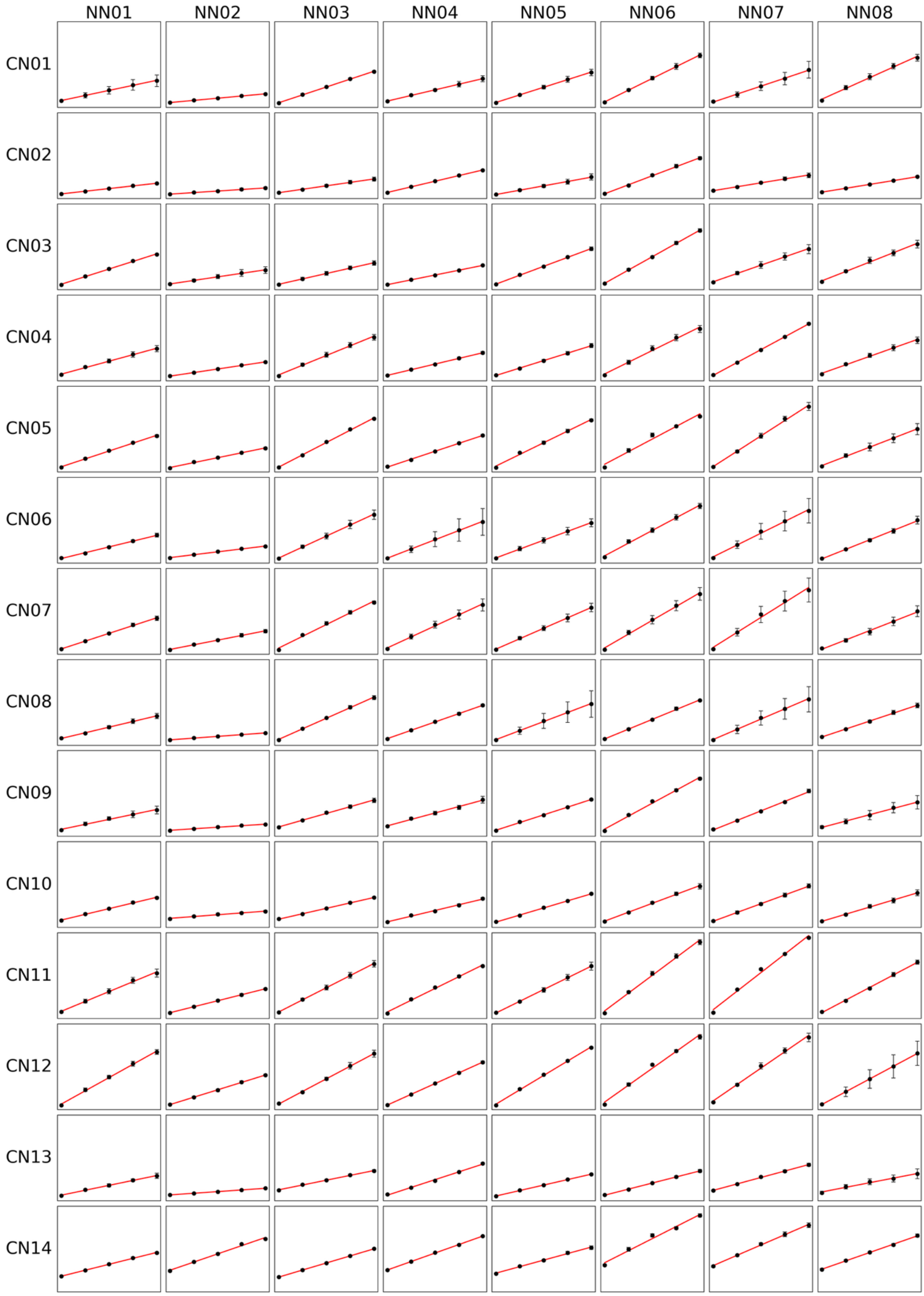

Figure S33: DHR-123 emission at 505 nm for complexes containing **CN01-14** and **NN01-8** plotted against 0-4 minutes of irradiation (blue light, 470 nm, 8 mW). Complex concentration is 2 μM, DHR-123 concentration is 20 μM. All graphs share the same axes (x, 0-3 minutes; y, 0.5-1.5 absorbance units).

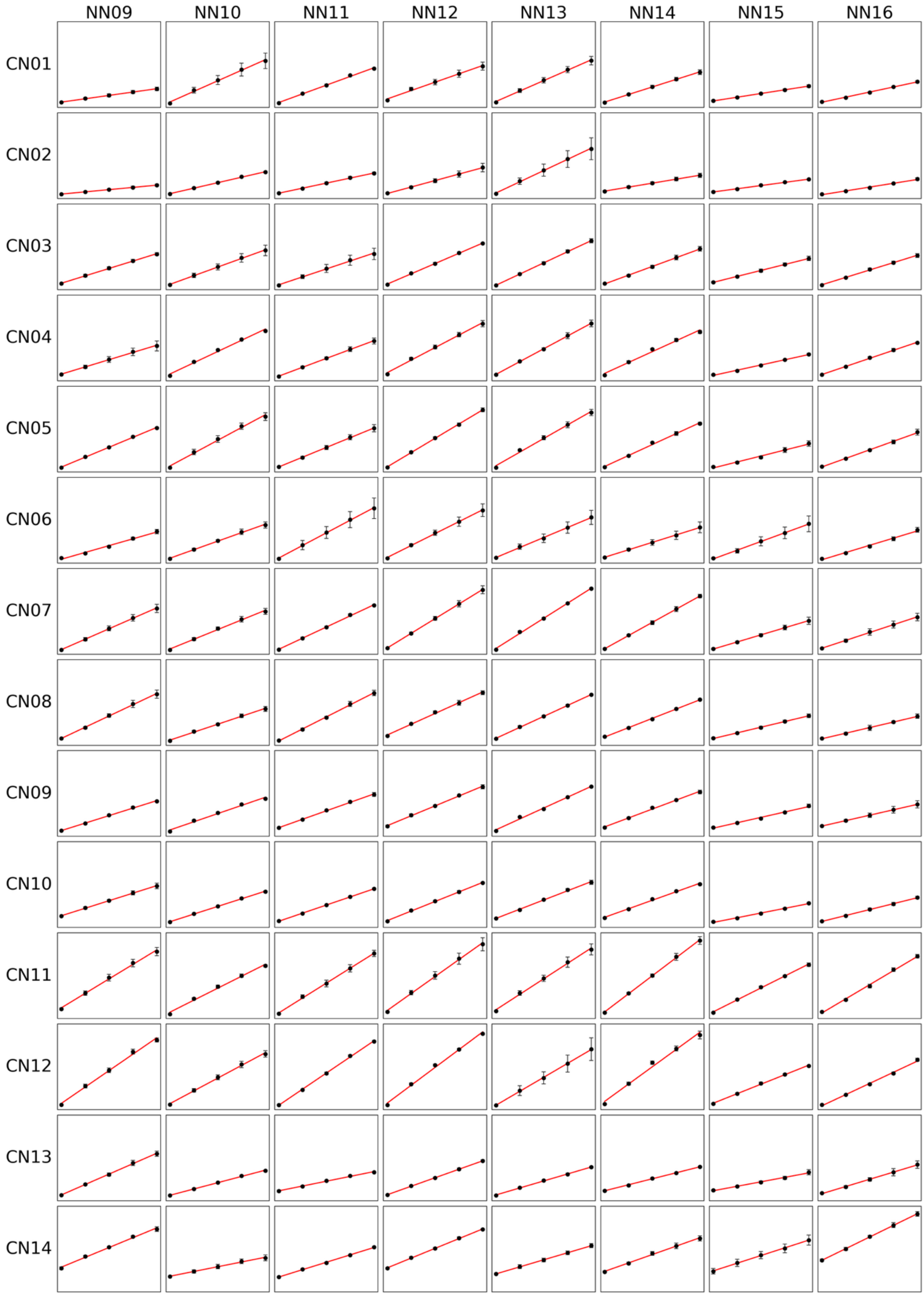

Figure S34: DHR-123 emission at 505 nm for complexes containing **CN01-14** and **NN09-16** plotted against 0-4 minutes of irradiation (blue light, 470 nm, 8 mW). Complex concentration is 2 µM, DHR-123 concentration is 20 µM. All graphs share the same axes (x, 0-3 minutes; y, 0.5-1.5 absorbance units).

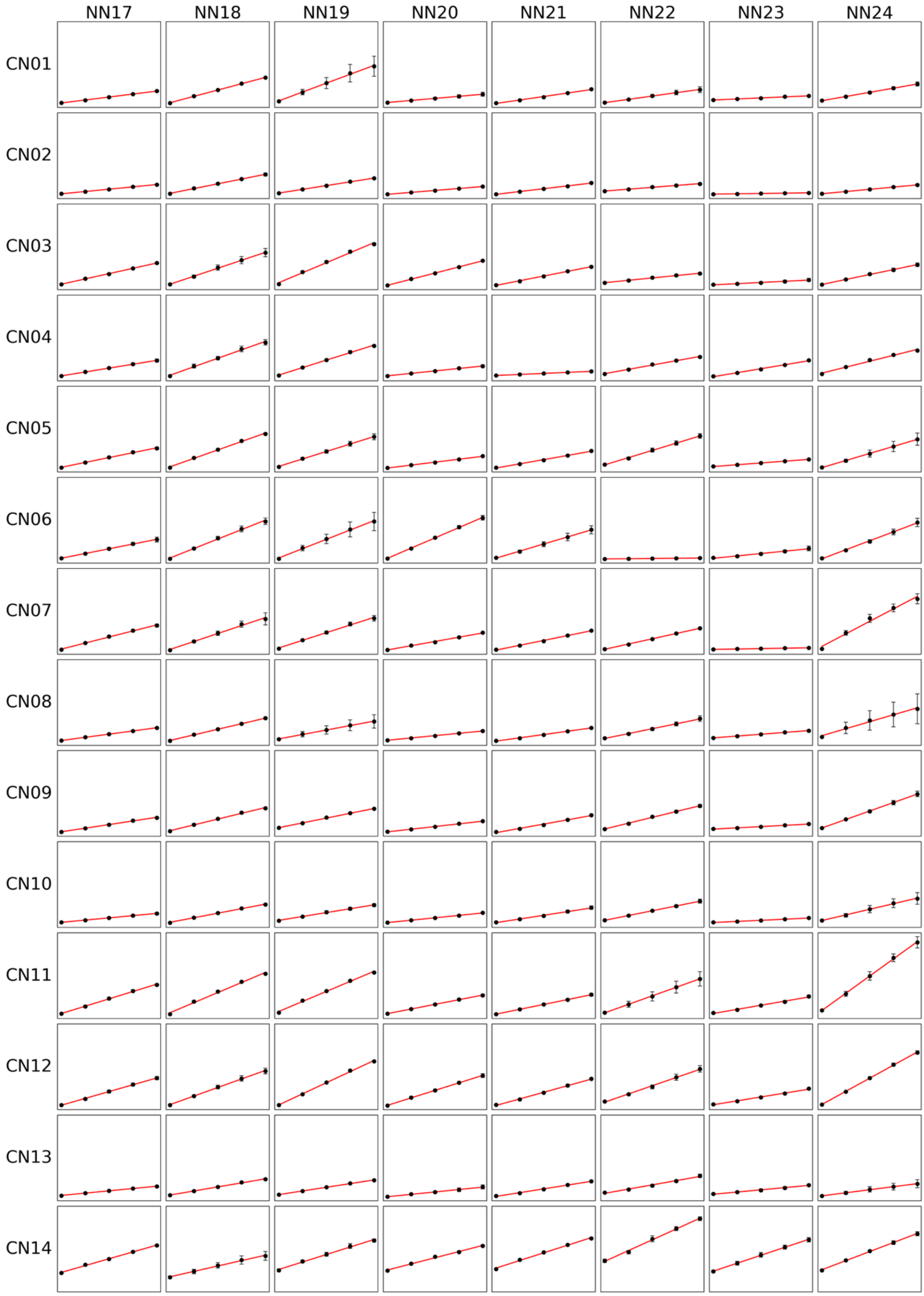

Figure S35: DHR-123 emission at 505 nm for complexes containing **CN01-14** and **NN17-24** plotted against 0-4 minutes of irradiation (blue light, 470 nm, 8 mW). Complex concentration is 2 μM, DHR-123 concentration is 20 μM. All graphs share the same axes (x, 0-3 minutes; y, 0.5-1.5 absorbance units).

Light, 1.5 µM

	NN01	NN02	NN03	NN04	NN05	NN06	NN07	NN08	NN09	NN10	NN11	NN12	NN13	NN14	NN15	NN16	NN17	NN18	NN19	NN20	NN21	NN22	NN23	NN24
CN01	13.1	72.9	3.6	31.7	3.7	86.4	63.8	-1.4	3.0	0.3	3.5	90.7	66.1	1.2	29.4	13.3	39.4	17.5	2.8	1.7	33.3	69.5	77.1	37.9
CN02	79.1	56.8	3.5	9.5	21.3	72.7	16.2	5.3	5.3	3.3	-1.6	74.5	14.6	4.3	34.9	58.6	119.0	76.0	11.6	12.5	99.7	58.9	64.6	13.7
CN03	103.0	63.1	40.3	95.3	80.2	83.1	97.8	85.1	84.5	77.6	66.6	91.5	89.3	69.4	88.2	100.0	103.4	54.6	56.7	76.0	34.5	62.5	78.8	107.5
CN04	2.4	73.6	28.8	50.2	1.6	64.5	-2.6	0.1	-1.6	0.2	5.2	79.5	97.2	61.6	59.5	85.5	73.8	8.9	4.4	21.8	95.3	64.0	9.3	81.6
CN05	91.1	86.7	73.3	77.4	106.7	71.1	11.1	76.5	58.3	84.0	91.0	86.0	94.2	55.2	63.0	72.2	110.3	76.0	92.3	84.0	93.4	75.5	71.0	6.6
CN06	99.9	37.8	2.7	0.9	55.9	55.7	52.4	14.8	0.1	98.6	13.8	7.6	6.7	10.8	-1.8	1.6	83.2	84.6	60.1	70.1	123.2	78.7	-1.7	1.0
CN07	31.1	46.7	21.2	3.1	8.4	85.0	-2.5	7.5	6.5	30.3	2.4	66.4	10.7	10.6	9.0	52.9	109.4	43.6	44.4	58.0	102.8	57.3	81.3	17.0
CN08	8.8	38.2	21.7	21.5	6.6	66.4	5.0	4.5	3.5	38.5	3.4	81.2	50.6	6.9	4.1	32.1	42.9	39.2	10.5	55.9	69.2	68.3	68.8	17.2
CN09	39.9	54.8	26.5	14.0	35.3	84.6	0.1	9.9	1.6	40.5	3.4	80.8	-2.1	13.1	65.8	71.4	82.2	56.1	62.9	85.5	56.6	58.9	81.7	2.2
CN10	50.4	72.1	33.3	17.1	91.5	71.1	-0.4	29.5	4.6	66.4	26.5	74.1	5.5	12.5	48.5	60.6	99.1	63.9	78.7	86.7	77.1	98.9	76.5	3.3
CN11	67.8	82.5	70.3	121.0	72.5	64.2	58.1	82.7	72.3	89.5	72.9	116.5	90.0	63.1	68.6	93.7	84.7	80.9	89.9	105.8	98.7	60.8	69.9	93.8
CN12	87.0	46.0	69.8	109.3	77.2	72.5	35.6	132.3	68.1	76.2	67.0	81.5	44.4	31.5	79.6	126.6	76.4	83.7	80.8	108.3	86.8	4.4	65.4	111.2
CN13	17.4	53.9	7.5	8.7	31.1	58.8	-1.8	5.2	0.2	20.5	2.3	73.1	36.7	-4.0	7.2	88.5	54.2	68.0	45.8	56.7	56.5	50.0	35.7	46.2
CN14	110.6	76.4	88.3	103.5	88.0	98.4	72.4	86.3	111.1	72.3	80.7	109.5	82.9	85.5	84.7	86.8	101.2	70.7	73.5	102.9	95.9	74.4	100.7	86.7

Light, 10 µM

	NN01	NN02	NN03	NN04	NN05	NN06	NN07	NN08	NN09	NN10	NN11	NN12	NN13	NN14	NN15	NN16	NN17	NN18	NN19	NN20	NN21	NN22	NN23	NN24
CN01	8.8	52.8	1.0	2.0	1.7	18.8	-2.6	-2.1	0.9	-1.5	-0.5	59.2	2.4	-5.1	3.4	-1.3	2.0	-2.0	2.0	3.5	4.9	13.1	72.8	-3.5
CN02	57.8	-3.0	2.9	0.9	-1.8	77.0	-4.1	-0.3	1.8	-3.6	-2.9	23.7	-1.9	-4.0	0.2	-3.3	13.5	0.3	1.0	1.7	14.8	-3.0	72.7	-4.0
CN03	43.2	71.2	6.4	97.6	36.4	84.3	57.1	8.3	6.8	10.4	28.2	78.6	91.4	69.2	99.1	81.5	88.7	7.7	27.1	5.0	0.7	61.2	73.3	115.2
CN04	1.4	49.5	0.1	13.1	-3.0	32.6	-3.6	2.1	-1.3	-0.1	1.0	55.8	66.3	8.7	8.9	68.2	21.0	2.6	2.3	1.6	94.4	45.0	2.5	50.7
CN05	50.0	25.8	52.6	8.6	59.8	50.8	-2.2	24.6	5.7	62.9	36.9	46.9	26.9	1.7	21.2	71.6	102.9	71.2	83.6	74.1	80.9	9.4	44.6	-0.5
CN06	94.9	2.1	1.0	1.4	4.7	11.9	4.9	2.7	2.3	14.3	-0.4	2.4	2.1	-1.8	-1.6	1.7	7.8	26.7	4.0	10.3	113.4	71.6	-0.2	0.5
CN07	7.6	6.3	8.8	-0.2	5.8	42.3	-1.6	-0.7	3.3	1.9	3.3	3.6	-0.8	9.9	-0.3	1.7	32.3	3.2	3.7	12.8	82.6	2.2	77.5	1.2
CN08	1.3	-2.1	0.9	2.7	-1.2	58.2	-4.1	0.6	1.4	-1.7	0.1	8.0	-5.2	-2.0	-0.3	1.0	3.9	0.9	-0.2	4.6	5.8	14.8	29.3	-3.1
CN09	7.8	5.6	1.4	6.6	-0.4	36.4	-3.0	4.3	3.3	3.3	0.6	5.9	-2.0	-2.7	3.1	3.2	22.0	9.2	15.2	17.7	0.9	-0.8	3.5	-1.2
CN10	4.5	17.7	2.3	7.4	24.9	32.9	-1.1	0.7	-0.2	8.8	7.7	1.5	-0.5	-2.6	-1.1	2.6	44.6	22.8	32.5	28.0	3.8	62.1	3.0	1.6
CN11	32.7	35.1	50.8	84.3	60.3	41.4	6.7	45.3	36.1	48.5	49.9	56.9	39.6	32.3	46.5	58.8	57.8	62.5	53.6	69.9	57.8	2.0	29.5	84.7
CN12	34.9	0.4	39.1	8.0	47.2	23.6	0.4	108.4	3.1	56.2	5.7	7.0	-2.9	2.8	28.6	98.6	43.2	67.9	66.0	108.3	47.7	-0.5	2.7	1.7
CN13	2.1	-2.0	1.7	3.3	1.4	15.0	-1.5	1.1	-1.9	-1.3	0.7	9.0	-3.8	-2.9	-0.8	4.9	3.0	22.7	6.0	10.8	-1.1	1.5	-1.2	-0.2
CN14	104.4	12.2	80.0	89.7	92.8	98.6	16.9	82.7	79.6	75.9	90.9	91.5	59.1	78.6	63.1	80.2	109.5	72.1	91.2	94.1	84.0	15.7	16.9	7.6

Figure S36: AT3 screening results for HTS336 library.

Dark, 5 µM

	NN01	NN02	NN03	NN04	NN05	NN06	NN07	NN08	NN09	NN10	NN11	NN12	NN13	NN14	NN15	NN16	NN17	NN18	NN19	NN20	NN21	NN22	NN23	NN24
CN01	52.6	70.3	83.4	84.4	89.6	118.1	54.1	4.7	8.7	63.9	76.0	66.1	74.5	66.7	74.6	59.8	93.6	83.2	75.7	77.2	68.5	91.1	74.0	48.9
CN02	93.2	93.7	58.9	94.5	61.9	91.7	75.9	37.6	94.3	61.1	-2.8	85.1	34.0	-2.9	84.3	77.4	104.0	78.9	86.9	72.1	80.9	86.9	80.8	-0.9
CN03	89.7	78.0	86.4	78.8	84.2	103.0	75.7	75.9	88.5	76.7	86.1	82.1	86.9	94.0	91.9	88.8	111.8	79.0	90.2	77.4	87.6	94.1	79.1	85.7
CN04	81.2	74.0	84.7	73.1	83.7	100.8	60.7	66.9	66.2	72.9	85.7	71.5	65.9	90.1	80.1	86.2	72.8	84.3	82.2	71.7	71.9	84.3	78.4	71.9
CN05	73.3	89.6	76.2	105.5	71.5	88.4	78.6	69.9	70.5	74.9	90.7	72.5	68.0	87.4	67.8	79.8	89.1	75.6	107.8	68.7	96.0	76.8	80.1	66.6
CN06	104.4	75.1	80.8	74.2	89.0	85.0	102.1	82.4	91.3	76.3	93.1	75.7	67.9	83.6	84.8	80.3	96.3	75.9	77.0	69.7	80.9	106.5	88.4	39.2
CN07	92.0	76.8	78.7	80.3	73.6	101.0	76.1	93.9	78.3	101.8	82.4	77.9	72.8	94.1	76.7	65.3	91.8	82.1	81.5	93.8	71.7	93.8	81.8	68.1
CN08	37.6	46.1	93.8	81.5	73.0	71.3	78.9	33.6	68.6	53.3	86.0	87.0	71.0	90.9	63.2	47.1	71.3	69.3	71.9	60.9	65.8	99.2	80.8	70.5
CN09	21.9	41.1	58.5	96.3	58.3	71.5	59.4	19.4	70.9	30.4	81.1	78.3	86.5	109.0	63.3	47.7	54.0	37.2	52.0	31.4	78.8	84.8	86.7	75.4
CN10	26.2	55.6	89.3	79.0	72.1	54.1	76.0	19.3	87.6	50.6	102.2	70.1	72.2	82.8	70.4	67.4	72.9	83.8	52.4	52.1	60.9	81.2	80.1	68.4
CN11	46.2	83.8	72.2	66.0	72.7	53.6	62.9	50.2	66.5	64.5	76.7	84.6	72.4	76.1	61.4	61.6	71.6	71.5	59.3	58.3	66.3	86.3	72.6	84.7
CN12	112.2	81.7	92.8	84.4	91.5	87.0	103.4	80.2	92.0	85.2	100.5	84.5	90.1	84.3	89.4	94.6	78.4	90.5	114.1	86.2	89.9	93.3	94.0	139.0
CN13	30.2	33.1	67.4	89.0	58.8	38.3	56.8	26.6	75.9	28.6	86.7	83.4	96.4	93.4	74.8	35.4	60.4	48.4	36.1	31.0	53.6	84.6	82.0	64.3
CN14	98.9	57.0	103.3	91.1	105.6	81.5	23.9	93.6	93.6	88.7	108.1	79.2	79.3	99.5	80.6	89.0	94.4	88.2	95.0	75.8	72.3	79.8	80.5	52.9

Dark, 10 µM

	NN01	NN02	NN03	NN04	NN05	NN06	NN07	NN08	NN09	NN10	NN11	NN12	NN13	NN14	NN15	NN16	NN17	NN18	NN19	NN20	NN21	NN22	NN23	NN24
CN01	3.6	56.8	79.4	66.7	66.6	85.6	0.7	-3.5	-2.6	16.4	-1.6	62.7	69.3	-0.9	53.2	29.0	41.7	70.2	64.6	69.1	70.6	74.5	64.8	15.5
CN02	9.6	114.2	56.1	0.3	14.1	86.8	2.7	-1.5	-2.6	11.5	-2.5	94.0	-0.3	-1.7	24.3	3.1	100.4	66.9	60.1	56.9	67.3	79.8	71.5	-4.7
CN03	89.3	70.2	82.1	76.5	74.0	106.6	58.4	61.7	84.3	66.7	78.7	74.0	90.6	90.9	90.0	76.0	115.7	81.8	81.9	84.7	87.2	89.0	87.2	71.2
CN04	17.5	67.9	74.2	68.5	85.8	69.1	50.2	32.3	29.7	64.4	87.6	60.9	62.8	81.4	78.7	88.7	58.5	72.8	69.4	46.5	70.2	82.4	70.6	67.3
CN05	73.9	82.0	74.7	79.5	62.4	91.5	52.5	70.8	73.0	66.4	93.2	68.0	48.3	75.6	63.6	67.8	83.8	67.6	93.7	71.6	69.6	62.9	72.4	67.5
CN06	101.9	62.4	81.1	43.1	87.2	81.5	75.0	64.4	89.3	66.9	68.9	67.4	39.1	32.5	79.0	58.2	100.8	74.4	74.9	70.1	70.0	113.7	64.9	-5.0
CN07	65.4	79.2	68.9	68.4	73.8	91.1	73.2	87.5	56.7	86.5	71.8	67.8	64.5	63.7	72.2	56.2	79.9	80.7	77.6	94.4	72.6	98.9	76.0	39.7
CN08	30.7	37.1	78.6	58.5	68.6	47.0	33.5	35.4	54.6	19.3	83.6	62.7	54.1	72.4	48.3	34.9	34.5	67.4	23.7	38.3	52.3	101.8	73.1	25.4
CN09	17.3	50.7	40.7	82.3	28.0	33.8	32.7	27.9	53.4	11.7	82.1	75.7	62.6	76.8	37.0	36.9	24.9	23.0	20.2	23.1	44.7	73.0	56.2	24.4
CN10	31.4	47.1	69.8	62.9	54.3	28.9	49.0	27.8	77.9	27.8	108.3	62.6	54.8	70.9	65.9	32.9	50.3	28.4	24.7	37.1	42.6	72.6	74.1	12.6
CN11	44.0	67.6	61.1	64.3	55.0	31.5	51.9	36.4	59.2	41.8	74.6	74.9	73.8	65.8	66.0	41.5	50.7	64.4	32.4	35.4	64.3	92.5	69.0	83.0
CN12	94.1	84.8	89.1	83.3	94.2	77.1	105.4	67.7	100.3	74.5	100.0	85.4	90.2	90.0	77.0	73.8	74.7	88.2	102.3	81.4	84.2	74.5	95.6	113.6
CN13	26.6	45.7	55.8	92.5	33.4	26.7	38.9	9.8	47.4	20.1	68.8	83.7	95.1	83.1	65.1	33.6	37.1	29.0	29.5	21.9	29.4	74.9	60.8	49.0
CN14	95.9	19.8	104.2	86.2	108.8	77.1	11.3	72.8	45.6	73.6	103.1	80.2	39.9	81.5	86.7	86.7	95.4	93.6	105.3	75.2	78.8	35.7	10.1	7.0

Dark, 15 µM

	NN01	NN02	NN03	NN04	NN05	NN06	NN07	NN08	NN09	NN10	NN11	NN12	NN13	NN14	NN15	NN16	NN17	NN18	NN19	NN20	NN21	NN22	NN23	NN24
CN01	4.1	37.1	76.0	19.0	52.3	65.6	-3.9	-2.4	-1.6	2.6	-1.7	57.3	54.8	-1.8	19.3	10.4	30.2	50.5	52.6	71.0	61.9	60.7	59.1	-5.0
CN02	3.8	96.8	23.0	-3.3	-3.9	83.5	-1.7	-2.9	-3.8	-0.5	-3.4	114.8	-3.7	-3.3	-2.5	-2.2	102.2	60.7	21.0	38.1	62.3	59.2	71.5	-4.1
CN03	56.5	65.8	68.1	67.9	76.7	86.1	55.7	57.8	67.0	55.1	74.9	63.7	76.2	89.2	83.3	68.9	86.5	71.2	74.7	78.3	85.2	87.8	105.0	62.2
CN04	31.1	59.2	71.3	73.6	78.1	49.7	42.8	14.6	41.2	38.4	78.8	64.7	61.0	76.2	75.9	57.6	57.0	70.3	61.3	60.5	63.6	67.7	67.0	56.2
CN05	67.5	71.5	78.0	65.2	72.8	77.9	30.6	79.9	61.8	71.5	87.2	72.0	44.9	52.6	63.7	65.0	77.4	66.4	89.3	62.2	63.8	56.1	57.3	10.8
CN06	86.5	61.5	84.3	29.5	102.8	80.5	78.1	61.0	61.6	62.6	55.7	51.0	8.5	16.3	65.9	58.6	109.4	67.4	67.0	64.2	69.0	117.0	59.9	-4.5
CN07	61.4	66.4	61.0	59.9	62.0	93.2	56.1	66.2	45.7	61.5	51.4	65.6	48.4	51.8	68.3	47.3	59.8	81.2	67.2	80.4	63.3	63.9	80.3	23.3
CN08	31.0	18.6	64.9	53.1	80.1	26.2	6.7	58.2	20.6	25.7	76.2	52.3	39.3	55.9	42.0	38.1	26.6	42.4	18.3	19.3	35.0	91.7	59.5	11.4
CN09	21.3	38.5	39.4	48.5	16.6	23.6	15.2	27.5	35.0	12.5	76.4	76.4	40.9	80.9	31.2	39.3	18.2	14.9	15.7	22.7	22.9	59.9	44.0	0.7
CN10	21.9	55.6	43.1	52.9	38.2	25.7	26.3	27.5	57.8	16.6	92.8	59.8	46.4	47.3	54.9	37.8	26.7	30.8	21.2	19.0	26.8	80.9	46.9	-1.8
CN11	30.8	67.3	61.9	58.9	49.5	26.3	47.4	32.3	52.6	30.8	62.8	57.9	63.5	67.7	42.9	32.7	39.4	55.8	28.0	32.0	47.0	80.2	61.9	70.3
CN12	73.3	95.0	80.8	93.0	94.0	70.1	129.0	59.7	105.6	74.6	96.2	78.4	100.1	86.2	83.5	71.9	62.8	77.5	89.0	65.6	87.2	71.0	93.8	127.0
CN13	28.1	37.2	44.6	89.2	27.8	26.3	15.6	9.7	26.9	18.1	51.5	82.3	82.0	40.7	52.6	25.4	32.5	28.8	23.7	21.2	25.0	73.5	71.6	37.4
CN14	77.7	13.7	101.8	55.1	90.6	82.3	-1.2	52.5	30.6	38.2	69.1	81.4	8.4	60.9	70.0	76.6	100.3	92.5	98.3	77.4	77.1	7.7	3.7	-1.5

Figure S37: AT3 screening results for HTS336 library.

Light, 1.5 µM

	NN01	NN02	NN03	NN04	NN05	NN06	NN07	NN08	NN09	NN10	NN11	NN12	NN13	NN14	NN15	NN16	NN17	NN18	NN19	NN20	NN21	NN22	NN23	NN24
CN01	50.4	75.8	13.7	26.0	41.3	103.7	28.3	104.0	11.6	7.6	27.7	86.4	44.5	16.2	26.3	11.3	74.0	37.1	11.5	4.6	64.9	84.6	90.2	43.7
CN02	83.1	104.5	37.2	43.1	51.4	108.6	34.5	4.9	37.6	28.6	27.5	93.8	60.6	10.5	71.5	65.5	102.5	48.9	73.8	11.3	99.1	88.6	100.5	22.5
CN03	93.4	99.1	50.2	102.0	96.9	104.4	88.9	94.1	84.0	95.6	77.8	93.1	109.5	94.6	107.8	98.6	98.4	93.8	80.9	15.6	98.0	101.3	97.3	98.0
CN04	12.2	105.1	38.4	57.6	94.5	110.0	75.4	51.4	1.4	5.8	40.5	96.9	96.8	94.8	97.0	101.6	96.9	33.7	51.1	7.5	107.3	99.0	88.1	98.9
CN05	112.3	84.2	106.8	97.3	111.8	99.4	75.0	102.9	79.9	110.7	97.1	102.0	82.9	89.4	102.0	111.6	103.2	112.9	100.6	106.9	108.3	88.7	98.3	46.8
CN06	41.3	10.7	11.1	6.5	75.5	64.6	15.4	13.2	0.4	46.7	1.6	25.9	4.1	37.8	24.0	33.3	65.9	73.5	59.6	94.9	98.2	51.1	40.6	6.3
CN07	20.1	38.5	4.5	4.0	35.2	98.8	60.7	6.0	1.1	9.2	0.8	51.0	43.6	16.5	75.6	50.3	75.1	49.0	24.4	42.3	100.5	68.6	93.6	11.3
CN08	37.8	57.0	66.1	42.7	43.1	106.0	21.1	108.0	6.8	53.3	11.8	49.6	12.0	14.1	54.3	60.9	53.4	82.8	57.9	53.9	96.6	64.2	89.4	47.2
CN09	61.4	77.3	18.5	10.5	92.9	105.1	58.3	75.0	2.6	82.7	0.2	30.9	65.7	1.0	81.5	97.4	105.3	102.6	100.3	100.6	107.6	93.1	99.4	94.7
CN10	61.7	70.8	52.7	33.9	94.0	97.5	53.3	67.4	9.5	90.1	23.1	48.3	9.1	0.9	87.4	88.8	100.1	101.4	100.6	102.8	104.8	90.1	69.2	48.5
CN11	93.3	104.1	112.2	83.6	105.0	94.2	107.5	86.1	104.0	100.8	105.8	91.2	103.9	98.8	105.4	89.5	100.7	107.3	101.0	96.9	98.7	100.1	102.3	94.1
CN12	105.1	67.6	100.9	72.1	110.7	100.5	74.0	98.0	57.8	105.1	95.1	57.8	102.0	98.1	99.0	101.0	103.7	110.4	104.4	101.7	99.6	46.3	97.0	73.1
CN13	49.9	90.3	57.2	29.0	68.1	94.5	7.3	66.5	5.2	45.5	7.5	91.6	21.5	2.9	52.5	75.8	71.9	102.6	88.7	106.6	97.6	78.9	66.8	14.9
CN14	120.3	121.7	105.9	109.2	114.1	104.7	109.1	102.3	116.1	107.4	110.9	105.0	102.4	116.6	106.0	101.7	111.8	108.3	106.5	106.3	102.5	112.2	112.2	115.2

Light, 10 µM

	NN01	NN02	NN03	NN04	NN05	NN06	NN07	NN08	NN09	NN10	NN11	NN12	NN13	NN14	NN15	NN16	NN17	NN18	NN19	NN20	NN21	NN22	NN23	NN24
CN01	1.9	34.6	1.4	1.1	12.7	57.7	-0.7	108.6	3.0	1.6	1.0	25.9	-0.3	-3.0	3.0	2.8	36.3	5.4	3.9	7.2	6.6	15.1	44.1	1.0
CN02	27.5	42.3	3.0	0.2	1.4	77.5	-0.8	1.8	1.2	-2.5	3.8	31.5	7.5	-3.7	8.6	3.7	47.5	6.8	9.7	5.7	27.1	7.9	93.7	-2.1
CN03	45.7	87.8	29.6	90.1	52.4	82.2	29.3	36.8	15.1	59.7	34.8	88.7	97.9	94.7	90.9	97.2	77.1	44.6	45.8	5.8	36.0	98.1	90.0	99.2
CN04	4.4	69.9	40.3	18.3	3.7	87.1	9.9	2.0	1.5	2.3	21.7	69.2	86.0	50.9	77.4	52.1	44.2	13.8	25.1	8.6	90.6	86.9	7.9	88.5
CN05	57.1	41.1	59.5	38.3	76.2	79.3	4.7	74.7	9.8	67.7	59.4	47.5	30.2	10.4	82.1	89.8	114.1	90.4	75.2	97.8	96.5	28.0	76.9	29.6
CN06	2.4	6.7	4.8	11.4	4.9	29.7	1.5	6.8	6.4	7.1	2.9	3.0	3.0	3.1	8.1	9.8	7.6	4.8	7.8	12.3	68.3	1.2	3.9	3.5
CN07	3.3	4.3	6.7	6.4	7.6	55.7	4.6	3.5	5.4	9.5	1.4	6.8	1.9	3.1	4.1	7.8	15.0	9.0	7.7	9.9	98.1	6.5	30.7	0.8
CN08	0.1	41.7	16.2	1.5	0.8	109.7	2.8	103.0	3.2	1.7	4.3	0.3	3.4	3.6	2.1	5.8	4.1	9.8	14.1	4.5	18.1	29.0	40.5	4.2
CN09	13.8	40.1	1.6	3.7	28.1	61.3	37.6	3.4	5.6	5.7	2.4	0.5	3.1	4.1	6.6	36.0	47.7	42.8	30.9	25.9	44.1	38.4	71.8	77.9
CN10	15.9	40.0	6.7	0.1	25.0	48.1	42.3	3.2	1.2	16.6	1.4	4.2	4.6	3.3	11.1	63.2	59.3	41.6	38.3	37.5	69.8	27.0	40.2	2.1
CN11	57.7	53.6	105.3	81.2	95.3	93.3	54.7	78.2	57.9	64.8	73.8	45.3	92.4	107.6	102.8	78.6	63.1	106.0	77.6	96.6	78.3	17.6	106.5	24.7
CN12	53.6	8.4	47.8	8.1	90.9	97.3	6.8	83.8	6.4	70.2	34.2	5.5	36.6	21.8	103.0	100.3	101.1	63.8	97.8	102.0	74.7	3.2	52.2	5.0
CN13	2.9	52.5	4.3	4.3	6.1	52.0	2.5	40.7	1.5	4.2	4.5	44.4	3.9	5.7	4.7	11.1	8.9	9.2	12.7	28.2	32.0	18.0	7.7	6.1
CN14	110.9	59.6	118.0	96.3	127.6	108.9	74.5	113.2	76.8	128.8	105.8	104.8	77.9	104.4	108.5	122.4	124.7	121.2	130.5	112.4	107.9	51.5	75.1	43.6

Figure S38: HEK screening results for HTS336 library.

Dark, 5 µM

	NN01	NN02	NN03	NN04	NN05	NN06	NN07	NN08	NN09	NN10	NN11	NN12	NN13	NN14	NN15	NN16	NN17	NN18	NN19	NN20	NN21	NN22	NN23	NN24
CN01	94.2	55.6	90.7	73.7	96.2	95.5	78.9	98.7	70.3	88.2	92.8	93.0	52.0	43.7	64.3	59.6	92.9	95.3	92.0	91.8	99.0	71.9	92.4	91.5
CN02	95.9	78.7	86.5	41.6	94.9	99.5	73.2	76.1	92.7	90.0	32.5	92.9	53.4	3.1	88.0	69.8	90.8	97.9	92.8	94.5	100.7	73.9	106.3	73.3
CN03	94.5	97.9	84.3	98.2	83.2	95.3	71.0	62.6	81.8	81.1	81.4	95.6	96.9	90.1	93.1	92.7	99.0	93.1	89.5	98.4	103.5	84.2	96.5	87.2
CN04	91.1	85.8	99.9	92.0	91.7	82.8	55.6	44.3	94.9	64.7	88.9	97.3	93.8	75.7	87.7	52.4	83.9	93.0	97.3	96.0	97.6	81.8	106.0	83.7
CN05	110.8	80.5	97.1	100.4	103.0	95.0	87.4	100.3	100.3	100.4	101.4	92.8	82.0	84.5	104.5	101.8	102.5	99.1	105.9	101.2	99.4	62.1	89.5	80.4
CN06	91.0	69.4	99.9	74.6	96.9	93.9	81.2	95.6	86.8	96.4	89.0	77.0	65.1	73.1	93.0	85.6	100.5	100.7	97.8	102.5	100.1	46.0	73.4	53.6
CN07	99.5	46.5	108.1	67.8	93.2	94.9	72.6	81.0	89.9	95.9	80.1	85.4	53.2	54.9	84.5	50.2	104.4	105.2	100.1	111.7	101.9	52.5	79.4	52.9
CN08	85.2	45.5	98.3	67.0	105.3	107.4	62.7	94.7	79.6	101.2	78.0	65.0	50.5	55.4	72.8	72.9	101.0	102.8	92.3	104.8	101.3	60.1	63.2	65.1
CN09	67.9	51.9	85.4	85.5	90.0	101.0	83.4	51.4	90.1	37.4	98.8	61.2	57.5	93.3	51.5	73.9	95.4	98.3	98.7	92.2	97.3	80.8	77.3	87.1
CN10	74.7	47.8	94.3	90.3	96.3	104.2	80.8	34.7	93.0	61.3	90.8	63.6	52.2	82.6	88.2	97.1	81.6	98.8	90.0	101.2	101.9	50.0	50.2	80.4
CN11	80.0	97.7	105.2	93.2	94.8	103.8	96.4	96.8	103.7	103.8	113.8	90.0	106.2	105.5	95.4	78.9	109.1	97.5	112.6	101.2	104.0	96.1	92.7	87.8
CN12	99.9	92.1	98.9	97.4	104.0	101.3	91.4	87.0	96.5	108.9	103.3	91.7	103.9	96.8	99.7	100.0	105.1	101.6	107.8	105.1	109.7	68.7	84.0	79.6
CN13	68.1	56.0	50.9	100.8	46.6	73.2	70.8	56.4	94.8	44.4	93.0	90.3	85.5	103.2	50.9	90.0	67.7	43.5	44.9	66.0	93.4	90.6	84.5	74.8
CN14	115.5	88.3	113.1	114.1	118.3	114.3	94.3	128.5	120.8	112.9	117.1	114.9	102.7	119.0	100.0	106.2	124.4	104.6	113.5	111.0	108.0	84.2	78.1	89.2

Dark, 10 µM

	NN01	NN02	NN03	NN04	NN05	NN06	NN07	NN08	NN09	NN10	NN11	NN12	NN13	NN14	NN15	NN16	NN17	NN18	NN19	NN20	NN21	NN22	NN23	NN24
CN01	24.9	49.9	96.9	43.9	79.5	79.1	52.3	91.5	1.6	52.3	39.9	88.8	46.8	28.2	52.8	38.0	58.9	90.6	77.6	86.7	105.4	45.5	62.5	50.4
CN02	56.5	57.8	16.9	3.2	30.2	94.1	9.4	26.2	-1.8	26.4	-0.7	82.3	48.0	-1.4	55.7	44.0	71.2	83.9	71.5	79.6	96.1	56.9	92.6	-1.2
CN03	84.3	90.1	79.5	99.2	66.6	86.7	62.2	39.9	72.0	71.9	63.6	92.5	89.0	95.3	97.6	69.9	90.1	92.0	87.0	95.2	103.8	77.7	97.5	86.9
CN04	61.0	78.1	94.3	88.2	90.2	72.4	43.7	29.2	62.2	49.8	91.6	91.7	85.6	65.1	80.7	46.8	71.1	89.4	93.5	83.1	93.5	73.1	101.4	81.1
CN05	106.9	55.2	105.6	101.1	103.5	95.2	82.6	100.1	98.6	111.1	103.1	93.5	61.6	57.3	100.4	104.6	112.5	105.0	105.5	106.2	111.8	43.7	71.8	53.1
CN06	95.8	52.7	90.3	62.1	90.2	109.0	68.9	73.1	65.1	91.0	79.9	57.8	55.0	37.1	69.2	68.0	93.5	102.7	102.5	102.0	113.0	36.9	48.1	-1.8
CN07	79.2	45.3	98.4	53.7	79.6	102.2	52.3	60.1	68.7	77.6	64.8	69.1	41.0	45.2	63.5	45.7	89.2	104.2	99.2	104.0	100.3	37.6	58.5	39.4
CN08	64.7	40.9	105.3	65.7	76.3	110.3	51.4	93.1	57.7	32.9	71.0	51.7	45.1	48.5	63.6	64.9	72.4	100.6	71.1	112.8	108.5	39.1	47.5	72.2
CN09	26.3	49.3	41.9	67.3	33.6	40.9	77.0	27.7	91.4	29.7	90.0	49.7	51.7	76.9	50.1	63.4	59.6	37.1	32.8	61.3	80.3	59.5	59.8	93.2
CN10	27.3	39.8	47.6	62.8	47.5	56.2	60.2	26.6	90.4	27.5	85.3	43.4	49.5	64.5	33.0	79.8	58.1	36.0	30.6	48.8	91.6	41.3	36.6	59.2
CN11	71.0	98.3	111.5	97.1	98.5	105.7	93.3	57.9	106.5	70.8	105.3	94.7	104.6	102.9	55.2	90.1	61.3	108.0	56.7	104.3	104.0	61.4	94.0	86.0
CN12	85.5	72.2	106.1	92.1	101.2	112.3	85.7	101.3	102.5	103.9	97.9	78.3	88.3	101.5	106.3	84.2	109.1	110.1	108.9	110.8	110.6	64.6	70.1	58.3
CN13	31.2	65.4	40.3	97.3	42.1	43.7	43.3	29.8	45.0	30.8	97.7	87.2	90.6	79.7	41.8	72.8	52.3	35.7	40.2	39.7	56.4	88.7	73.9	65.7
CN14	125.3	67.5	122.4	104.4	127.0	117.0	59.0	103.7	107.1	119.2	116.3	112.5	68.3	98.0	93.8	90.7	124.3	107.7	117.4	116.0	116.7	53.9	55.3	49.8

Dark, 15 µM

	NN01	NN02	NN03	NN04	NN05	NN06	NN07	NN08	NN09	NN10	NN11	NN12	NN13	NN14	NN15	NN16	NN17	NN18	NN19	NN20	NN21	NN22	NN23	NN24
CN01	3.9	51.7	93.7	27.8	74.8	70.1	37.9	94.9	-0.9	37.4	13.6	71.7	44.9	22.0	45.7	25.2	59.5	72.8	60.2	67.7	96.6	40.8	48.9	29.7
CN02	9.3	40.3	8.0	-0.5	12.2	84.3	-3.1	-0.4	-1.5	4.9	-3.5	69.1	38.7	1.0	35.8	27.2	22.1	36.5	46.3	68.9	92.6	52.1	113.1	-4.2
CN03	68.9	87.0	73.5	90.1	63.5	79.0	48.8	38.0	53.6	61.0	58.1	83.0	86.1	88.6	90.7	59.2	84.5	81.7	81.4	80.4	99.8	70.4	89.7	74.7
CN04	45.7	74.0	92.6	82.9	82.7	60.3	40.5	23.8	47.9	49.9	89.1	80.3	82.1	63.0	86.2	40.2	62.9	89.1	82.6	82.9	87.1	68.5	101.0	72.4
CN05	105.6	51.9	105.3	89.3	104.1	79.3	74.3	96.1	89.4	102.9	101.1	82.5	55.3	49.5	96.8	92.2	106.8	95.8	103.8	112.5	105.5	38.9	55.8	49.0
CN06	84.2	43.9	93.3	46.7	77.4	100.6	36.9	55.2	47.4	85.9	74.1	48.2	44.3	21.0	50.5	54.3	87.1	92.9	99.6	103.3	107.3	26.5	42.6	-3.0
CN07	65.0	47.1	95.0	46.8	73.3	90.3	42.4	48.6	59.6	73.0	55.1	56.2	42.1	46.8	51.7	40.9	79.7	107.5	103.1	106.7	102.5	29.3	41.2	36.7
CN08	53.1	33.5	105.2	47.8	59.2	99.2	53.8	97.3	46.8	33.9	63.6	42.5	41.7	36.3	57.9	53.5	54.2	86.1	40.4	66.9	102.0	36.5	40.3	56.0
CN09	27.0	37.7	38.1	64.1	30.9	37.8	39.3	37.3	76.1	23.5	81.4	45.1	37.4	64.9	53.7	45.8	50.9	33.8	29.1	35.6	69.8	45.4	61.0	85.4
CN10	21.1	34.2	28.7	54.8	34.9	40.9	49.3	37.9	77.2	22.6	87.6	41.2	51.5	52.6	45.8	72.5	45.3	27.4	32.5	36.8	62.3	34.2	34.6	31.4
CN11	60.9	90.1	101.6	98.8	91.8	75.0	84.8	41.4	104.1	46.2	104.8	83.6	93.6	96.0	49.9	83.6	58.2	114.2	45.1	70.4	110.5	46.5	88.3	72.0
CN12	71.2	62.8	107.3	85.3	104.7	107.6	82.1	99.2	86.9	102.9	96.2	64.1	73.3	93.1	91.6	89.4	107.4	106.9	106.8	100.9	110.7	67.0	60.4	49.1
CN13	29.4	57.8	35.3	102.7	40.6	39.8	34.5	30.5	39.9	29.4	79.2	91.0	80.4	75.4	38.1	52.4	48.9	31.4	33.6	33.3	44.9	78.6	68.8	64.4
CN14	119.7	49.7	124.1	97.3	121.2	112.0	37.2	92.3	85.5	116.3	105.6	96.2	51.3	82.2	83.9	64.3	120.0	118.3	114.8	115.1	112.5	36.0	43.7	19.8

Figure S39: HEK screening results for HTS336 library.

Light, 1.5 µM

	NN01	NN02	NN03	NN04	NN05	NN06	NN07	NN08	NN09	NN10	NN11	NN12	NN13	NN14	NN15	NN16	NN17	NN18	NN19	NN20	NN21	NN22	NN23	NN24
CN01	36.0	93.8	10.7	18.1	6.4	110.9	24.5	15.0	3.0	5.2	13.0	116.9	10.4	12.8	62.6	24.7	79.4	15.9	9.8	2.5	38.4	85.2	117.2	77.6
CN02	78.5	92.4	29.4	12.3	11.2	111.2	7.1	6.4	12.4	16.1	20.5	100.9	4.9	8.0	84.1	81.7	102.3	50.6	51.4	116.0	73.4	99.0	109.9	6.5
CN03	105.0	103.8	76.4	111.2	94.2	110.9	102.4	104.2	87.9	107.5	90.3	112.2	99.2	104.6	111.2	108.3	105.4	98.0	101.7	32.2	31.3	110.8	113.0	105.4
CN04	7.4	108.1	12.1	53.1	6.2	105.7	6.4	3.0	1.3	4.6	35.4	111.1	105.2	57.5	102.6	108.6	100.3	40.4	37.2	5.3	98.3	108.1	6.3	110.6
CN05	108.1	104.5	110.4	109.4	107.0	108.6	16.8	106.9	80.3	112.7	110.3	107.6	105.6	79.5	108.7	109.6	105.6	107.9	112.0	108.7	105.8	88.8	110.0	10.1
CN06	18.4	2.6	12.2	2.3	5.2	94.4	3.1	6.0	-0.4	58.0	3.5	25.7	5.5	1.1	3.8	16.4	54.2	89.7	65.7	85.2	103.1	110.9	3.9	1.6
CN07	18.4	49.8	10.3	4.8	6.6	104.1	4.0	4.7	14.0	17.9	5.4	104.1	13.0	5.0	20.0	60.8	63.3	37.0	94.9	76.4	98.9	81.4	104.0	22.4
CN08	24.7	68.9	58.6	31.4	12.0	109.6	15.8	107.9	6.9	31.7	24.8	101.9	26.6	10.1	33.3	40.2	45.6	67.1	40.8	18.3	71.8	114.1	110.3	35.6
CN09	66.5	103.3	52.9	10.4	64.3	108.4	2.5	17.2	3.3	75.1	11.0	86.9	0.6	5.4	88.3	105.5	111.1	109.3	109.0	98.2	65.3	96.6	102.6	2.7
CN10	78.6	108.4	74.8	50.8	96.3	114.4	8.4	74.8	11.7	107.8	47.9	111.4	61.0	6.9	104.2	98.4	109.0	113.3	116.8	105.6	76.9	100.6	109.7	3.0
CN11	113.6	111.9	110.9	90.5	105.2	115.3	106.1	99.4	113.3	112.7	111.1	96.1	110.9	109.0	112.3	94.2	110.2	118.1	116.6	99.6	92.5	76.3	112.7	81.9
CN12	111.5	65.7	111.7	82.6	111.4	112.4	22.9	94.2	82.2	113.7	97.5	82.2	64.0	55.9	112.7	100.6	108.8	112.2	118.9	100.3	97.1	3.6	90.9	3.4
CN13	55.4	100.4	56.1	70.2	73.0	107.2	2.2	12.9	17.3	101.1	51.9	93.3	18.5	12.5	35.7	81.0	81.3	99.7	92.9	99.0	44.2	91.0	79.5	100.2
CN14	108.0	108.7	117.9	112.6	106.2	110.1	109.9	112.5	108.9	108.3	110.3	111.9	101.0	111.0	110.5	108.8	111.1	117.4	110.7	118.8	102.7	108.9	108.5	107.6

Light, 10 µM

	NN01	NN02	NN03	NN04	NN05	NN06	NN07	NN08	NN09	NN10	NN11	NN12	NN13	NN14	NN15	NN16	NN17	NN18	NN19	NN20	NN21	NN22	NN23	NN24
CN01	7.4	12.2	2.4	0.8	2.5	28.5	-1.2	0.2	0.7	-1.1	4.4	59.7	2.1	-1.0	2.7	0.8	17.8	-0.2	4.3	1.4	4.9	1.2	78.5	-0.9
CN02	6.6	7.3	-1.4	-0.8	-0.1	96.0	-1.0	0.1	0.8	-0.2	-1.9	22.0	-0.1	-2.3	-0.0	0.6	7.7	3.4	8.8	2.0	9.0	-1.6	109.2	-1.1
CN03	46.9	98.0	17.6	105.2	36.8	83.1	54.7	15.3	18.9	33.6	28.8	91.5	91.5	86.5	98.8	92.1	81.1	20.4	35.4	3.7	1.5	93.4	92.4	81.4
CN04	4.9	80.7	7.1	13.0	1.1	68.1	0.5	2.0	-0.2	0.0	6.0	76.6	85.3	2.5	31.0	52.1	19.2	6.1	10.1	1.0	106.2	72.3	4.9	75.8
CN05	56.0	32.8	66.6	10.4	60.7	76.8	0.4	12.4	10.9	79.2	56.5	63.8	18.3	3.9	36.0	92.7	109.4	96.3	79.4	82.1	68.1	2.5	74.7	0.1
CN06	1.3	0.3	5.0	0.9	3.3	12.0	0.7	3.0	1.1	6.6	1.5	2.0	3.9	-0.1	1.6	2.7	5.8	15.0	4.5	8.2	65.3	104.0	2.7	0.9
CN07	3.9	1.7	7.7	0.8	4.3	83.2	0.2	0.9	4.4	8.7	3.6	29.6	2.0	2.4	14.6	2.1	8.0	2.4	5.8	7.1	33.3	5.8	104.4	1.7
CN08	1.6	11.1	9.2	2.8	2.0	98.5	-0.3	0.8	-0.2	0.7	0.2	10.5	-0.7	0.9	2.0	2.7	3.9	4.0	1.5	5.2	7.8	15.0	45.6	-0.0
CN09	8.9	9.5	5.1	1.6	2.9	90.1	-0.9	5.8	2.3	4.1	1.6	5.4	-1.0	0.0	4.6	17.0	33.9	9.9	19.8	11.7	5.8	2.5	13.6	-0.2
CN10	10.5	24.8	-1.5	11.3	16.1	91.8	-0.1	2.2	1.7	8.1	7.8	3.3	1.4	0.2	6.3	13.3	50.3	34.2	54.7	23.1	5.7	16.9	17.3	0.3
CN11	74.7	29.7	103.3	53.3	106.9	68.1	12.9	78.9	63.1	87.0	83.6	56.5	51.3	58.0	59.3	81.8	96.0	100.8	96.8	84.0	24.5	3.9	37.3	3.5
CN12	63.1	3.5	30.1	3.5	62.6	68.8	3.4	48.7	2.7	82.1	4.9	5.5	0.8	5.2	16.2	94.8	79.8	94.0	80.5	93.0	24.4	1.6	4.5	1.7
CN13	3.2	40.4	4.8	2.5	10.2	28.1	0.6	3.3	-0.8	1.3	-5.1	34.1	0.7	-0.3	1.6	8.8	6.6	18.0	8.4	11.1	2.1	7.2	2.8	1.7
CN14	100.9	90.0	100.8	93.9	102.5	91.1	55.0	101.8	75.8	102.7	106.4	75.4	60.3	66.9	94.7	97.9	101.1	106.9	114.8	102.9	106.3	54.2	58.5	7.4

Figure S40: HeLa screening results for HTS336 library.

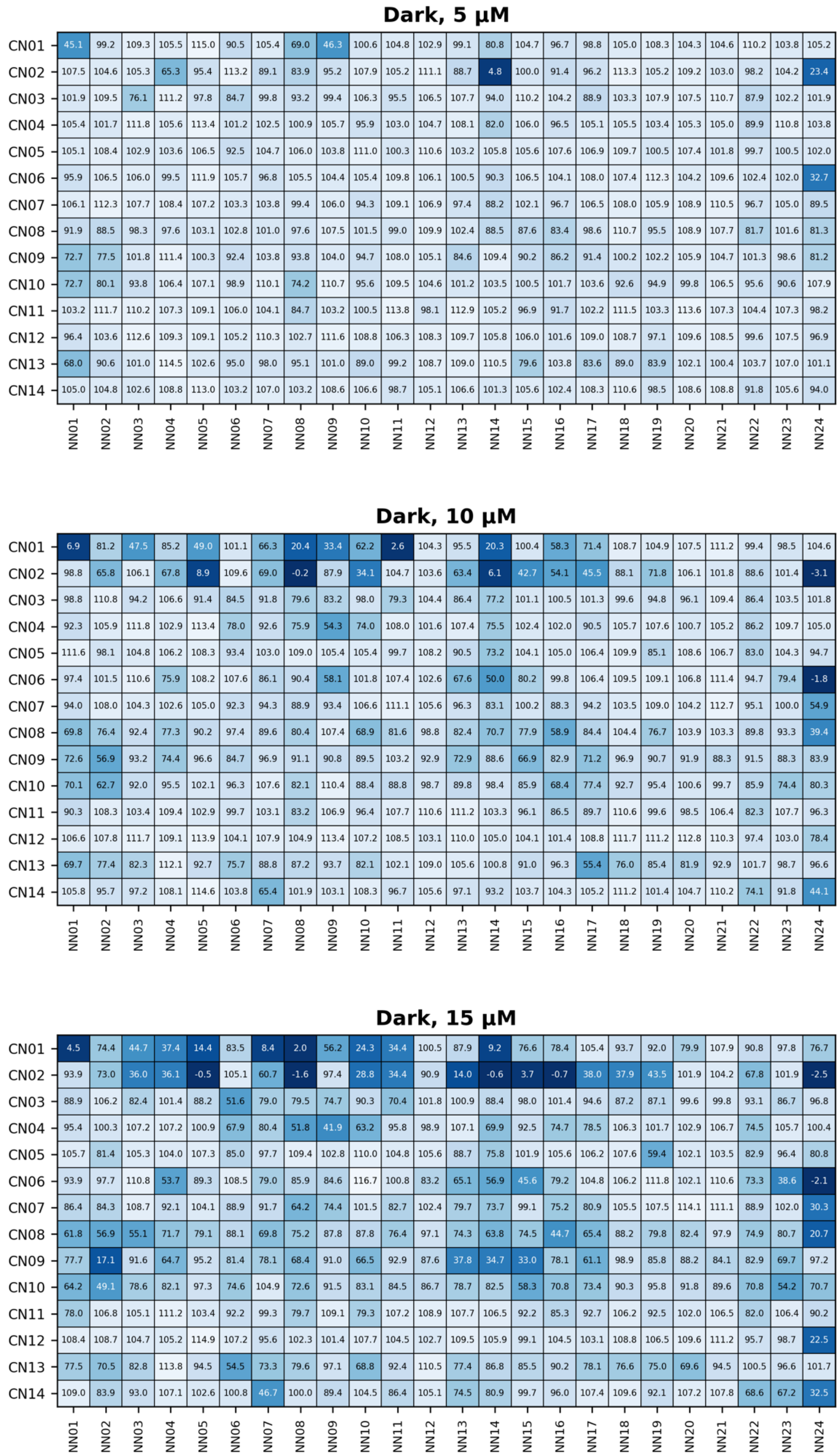

Figure S41: HeLa screening results for HTS336 library.

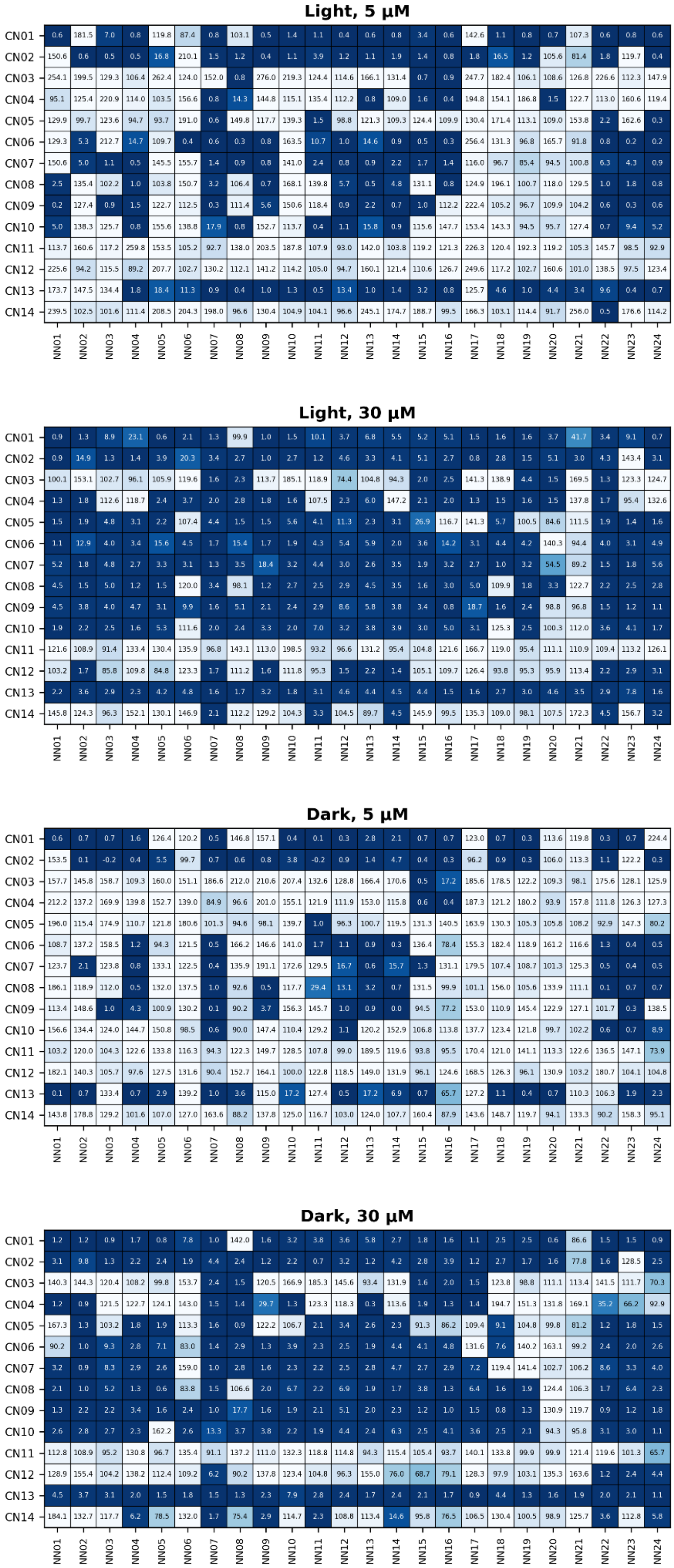

Figure S42: B Sub screening results for HTS336 library.

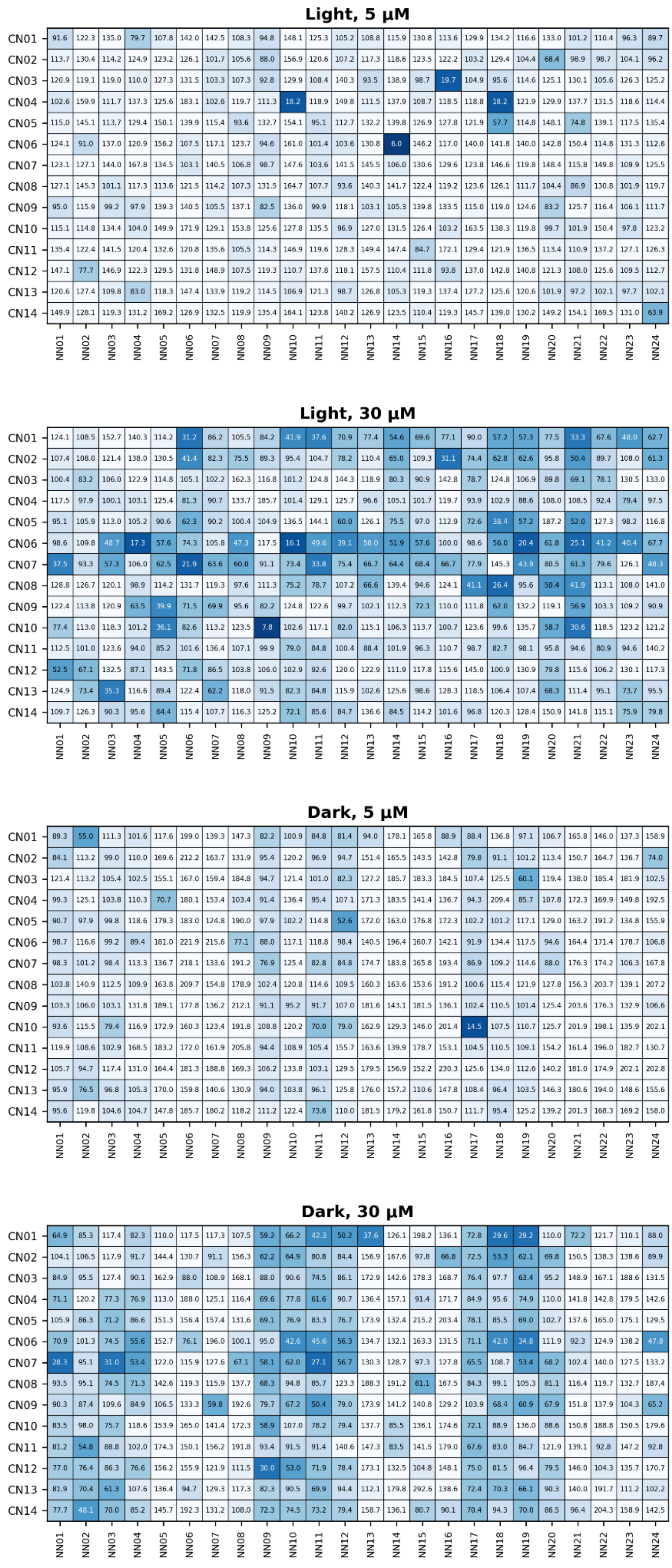

Figure S43: E Coli screening results for HTS336 library.

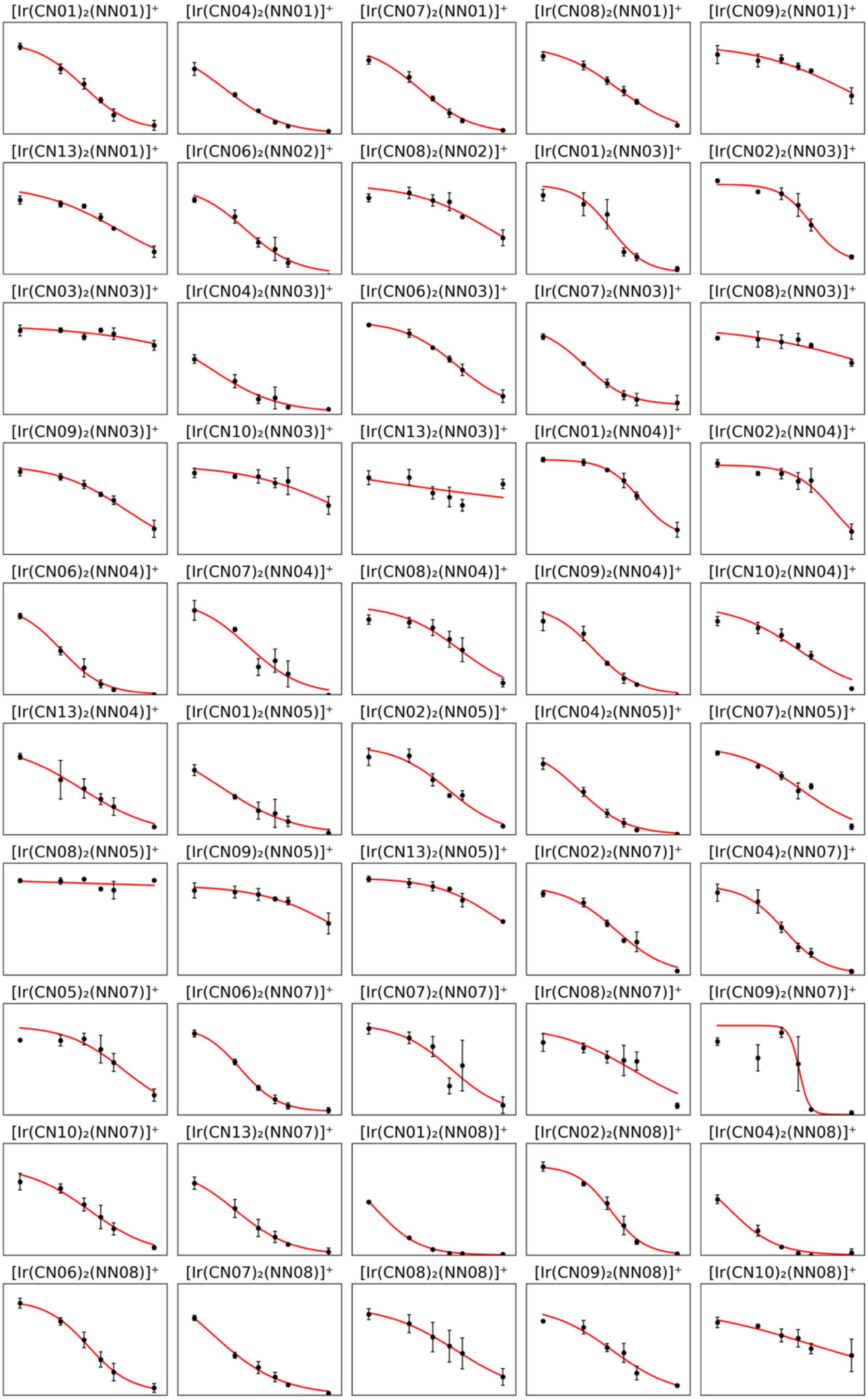

Figure S44: IC_{50} fits for selected complexes from **HTS336** in AT3 cells. Complexes were tested at 0.2, 0.4, 0.6, 0.8, 1.0 and 2.0 μM in triplicate, and irradiated for 12. Minutes using 15 mW/cm^2 of blue light (470 nm).

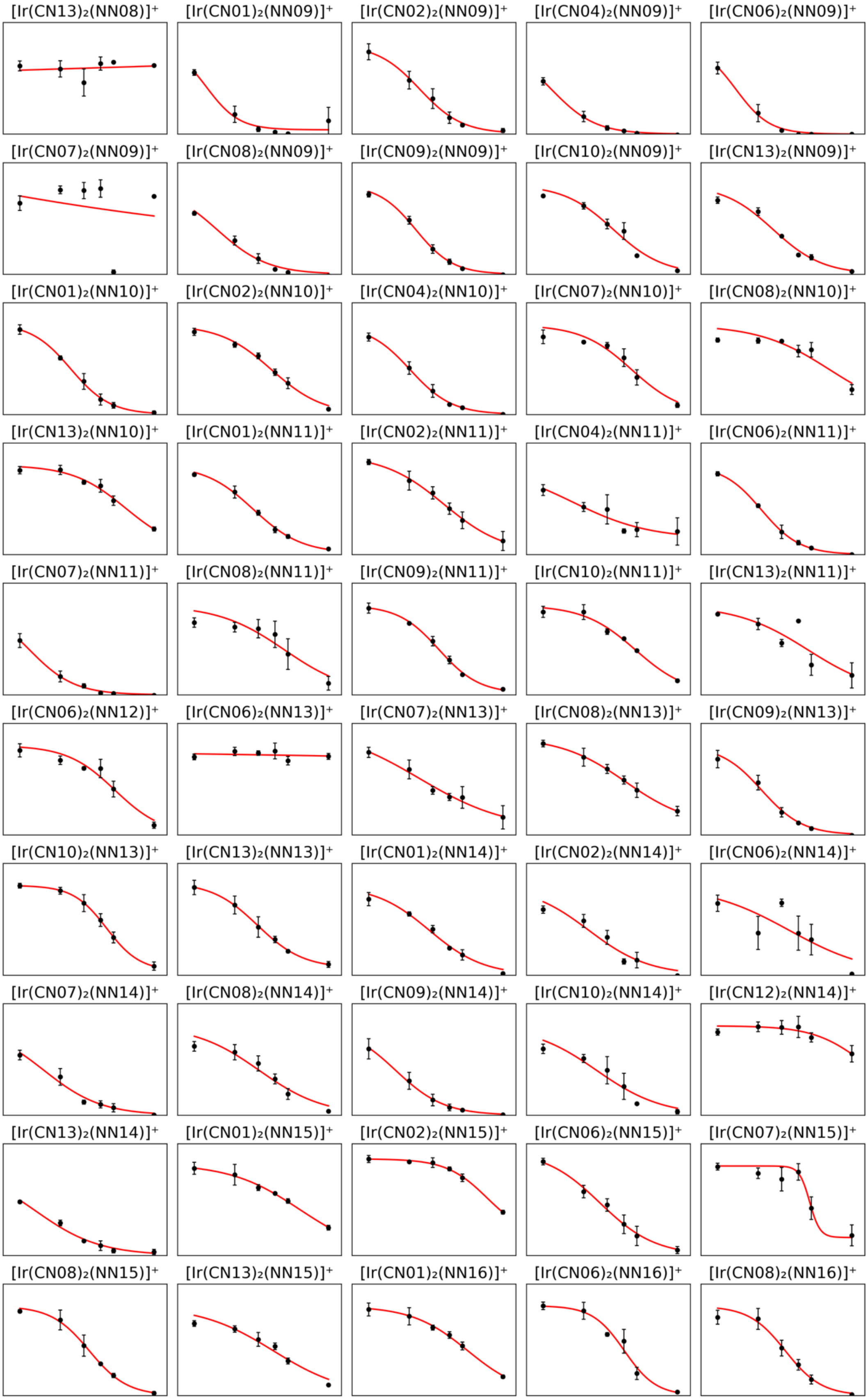

Figure S45: IC_{50} fits for selected complexes from **HTS336** in AT3 cells. Complexes were tested at 0.2, 0.4, 0.6, 0.8, 1.0 and 2.0 μM in triplicate, and irradiated for 12. Minutes using 15 mW/cm^2 of blue light (470 nm).

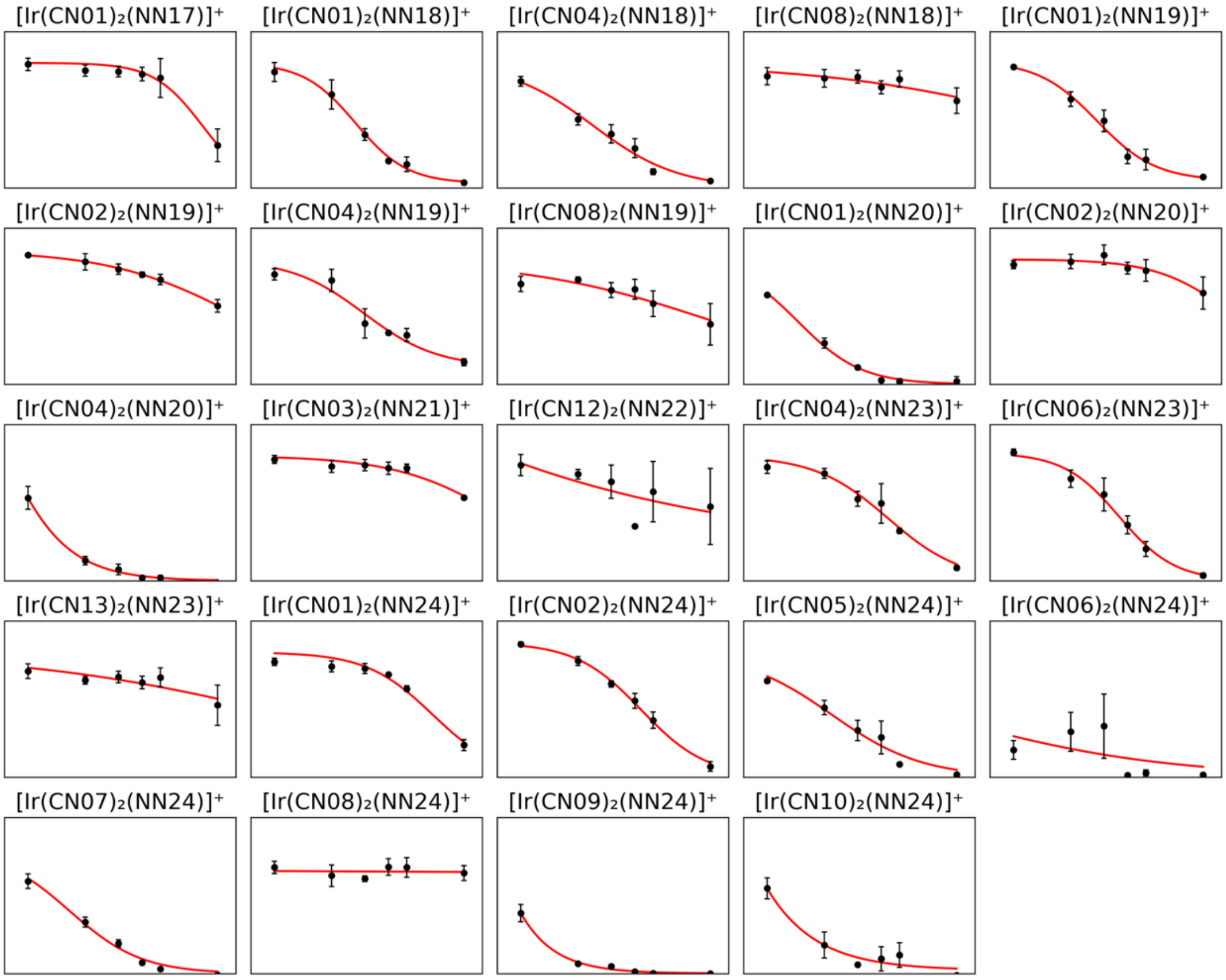

Figure S46: IC_{50} fits for selected complexes from **HTS336** in AT3 cells. Complexes were tested at 0.2, 0.4, 0.6, 0.8, 1.0 and 2.0 μM in triplicate, and irradiated for 12. Minutes using 15 mW/cm^2 of blue light (470 nm).

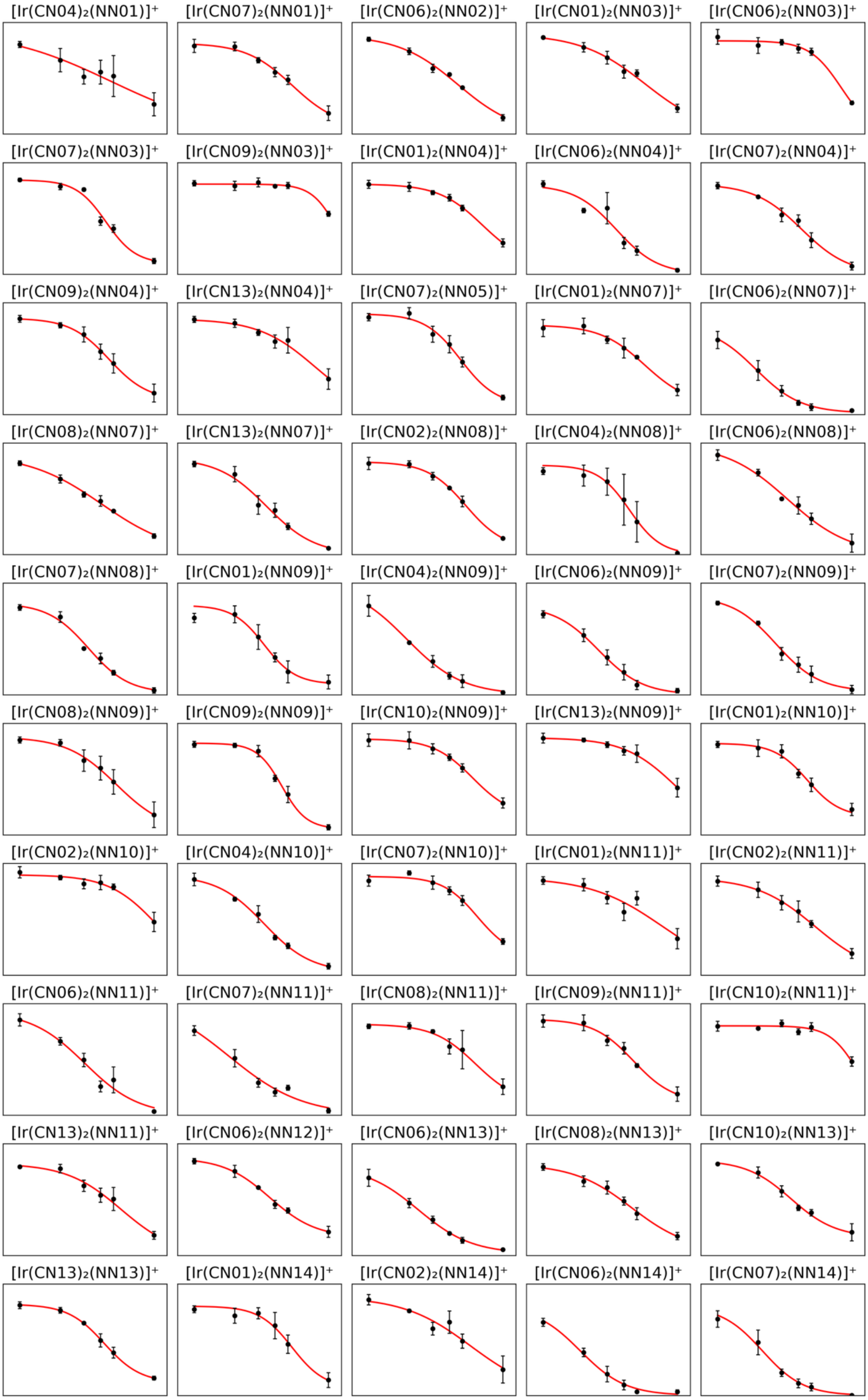

Figure S47: IC_{50} fits for selected complexes from **HTS336** in HEK cells. Complexes were tested at 0.2, 0.4, 0.6, 0.8, 1.0 and 2.0 μM in triplicate, and irradiated for 12. Minutes using 15 mW/cm^2 of blue light (470 nm).

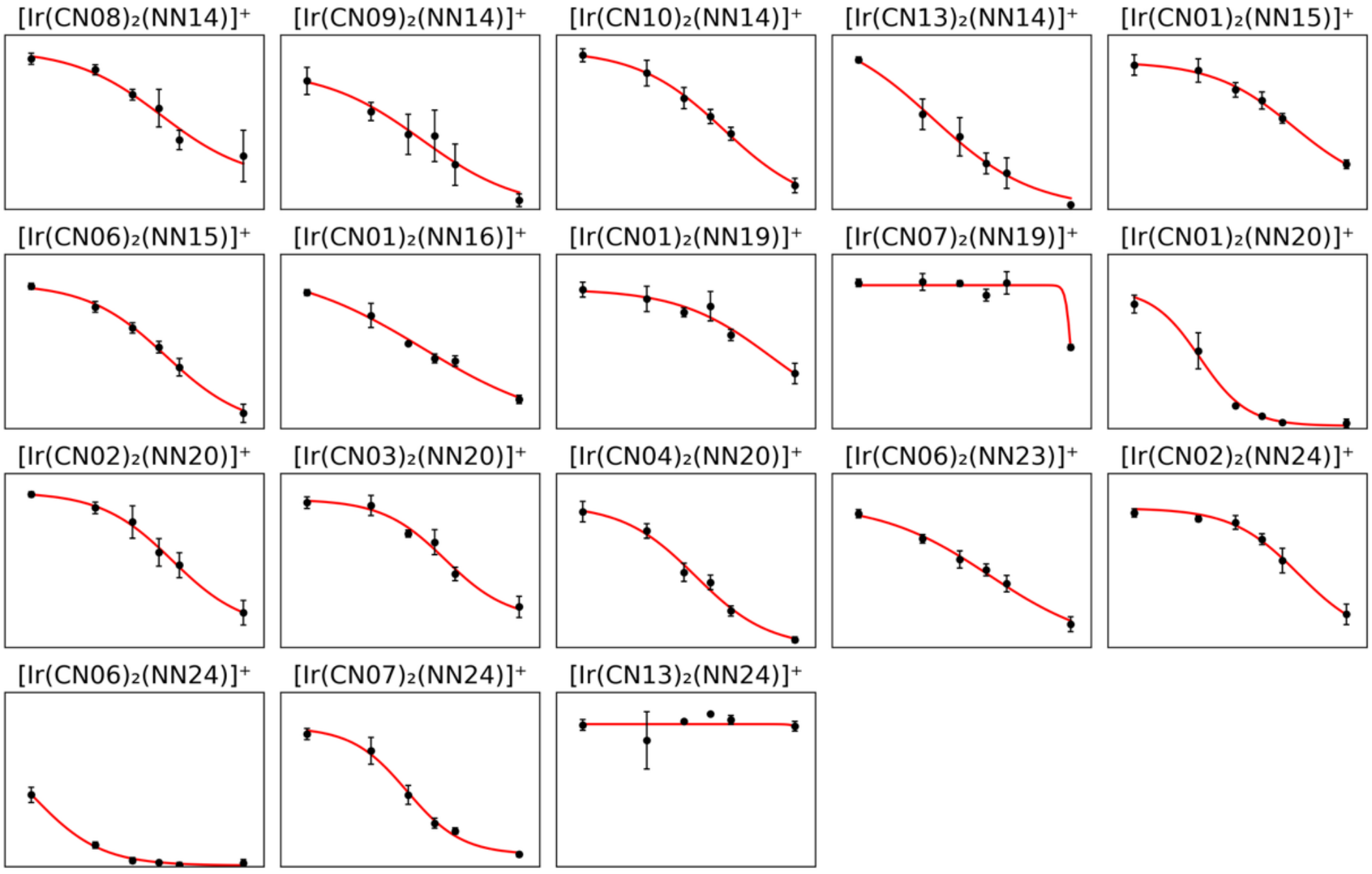

Figure S48: IC_{50} fits for selected complexes from **HTS336** in HEK cells. Complexes were tested at 0.2, 0.4, 0.6, 0.8, 1.0 and 2.0 μM in triplicate, and irradiated for 12. Minutes using 15 mW/cm^2 of blue light (470 nm).

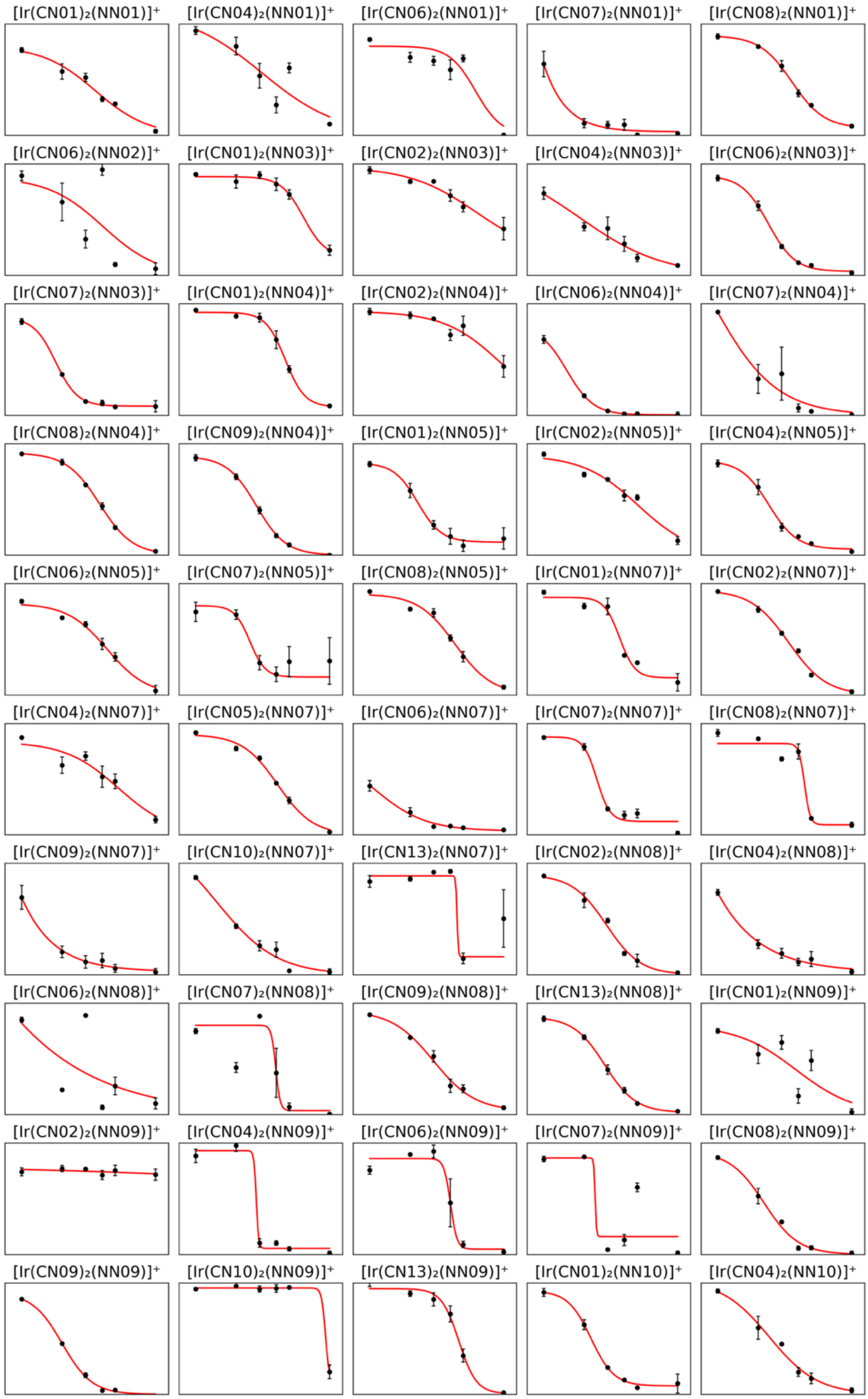

Figure S49: IC_{50} fits for selected complexes from **HTS336** in HeLa cells. Complexes were tested at 0.2, 0.4, 0.6, 0.8, 1.0 and 2.0 μM in triplicate, and irradiated for 12. Minutes using 15 mW/cm^2 of blue light (470 nm).

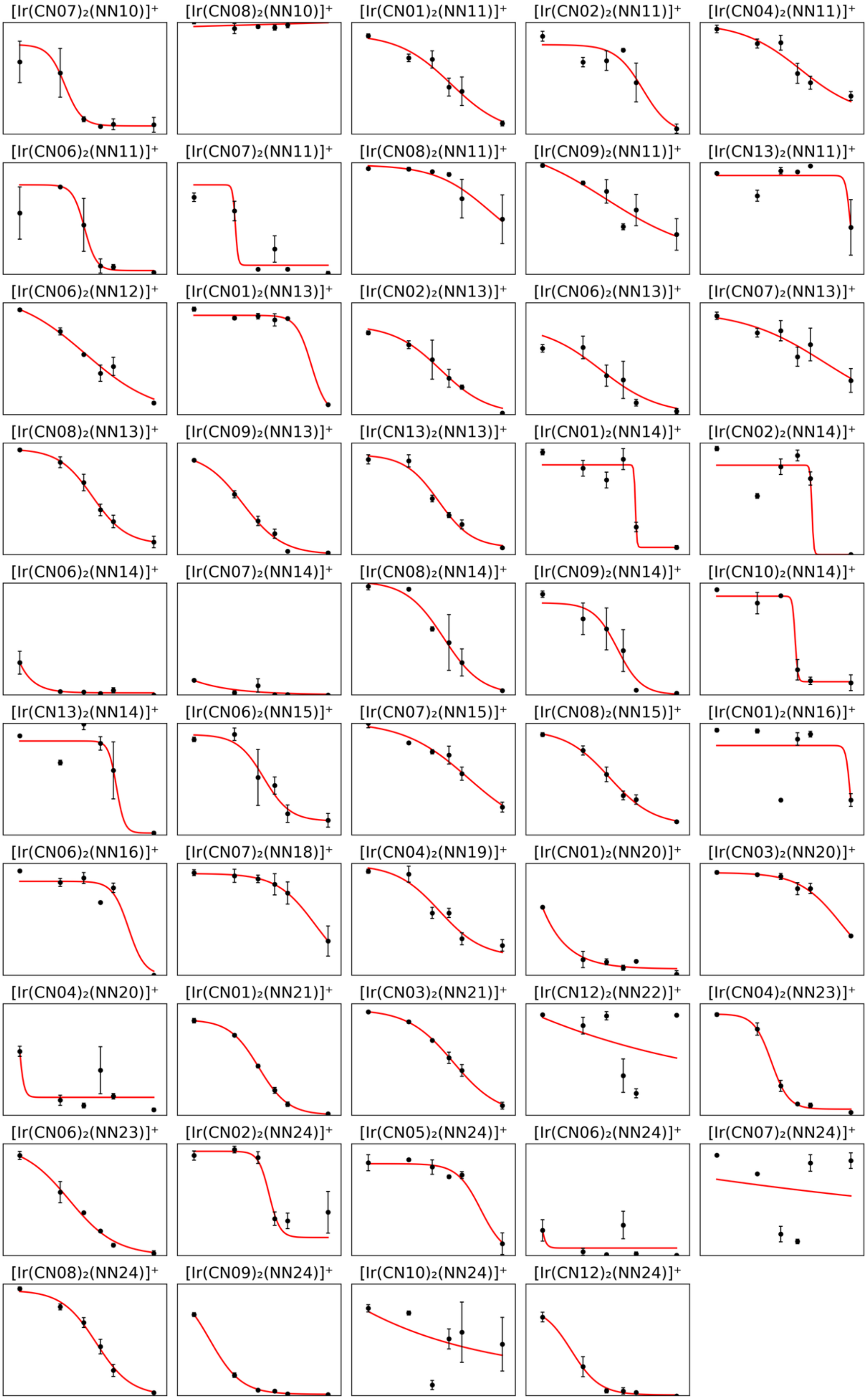

Figure S50: IC_{50} fits for selected complexes from **HTS336** in HeLa cells. Complexes were tested at 0.2, 0.4, 0.6, 0.8, 1.0 and 2.0 μM in triplicate, and irradiated for 12. Minutes using 15 mW/cm^2 of blue light (470 nm).

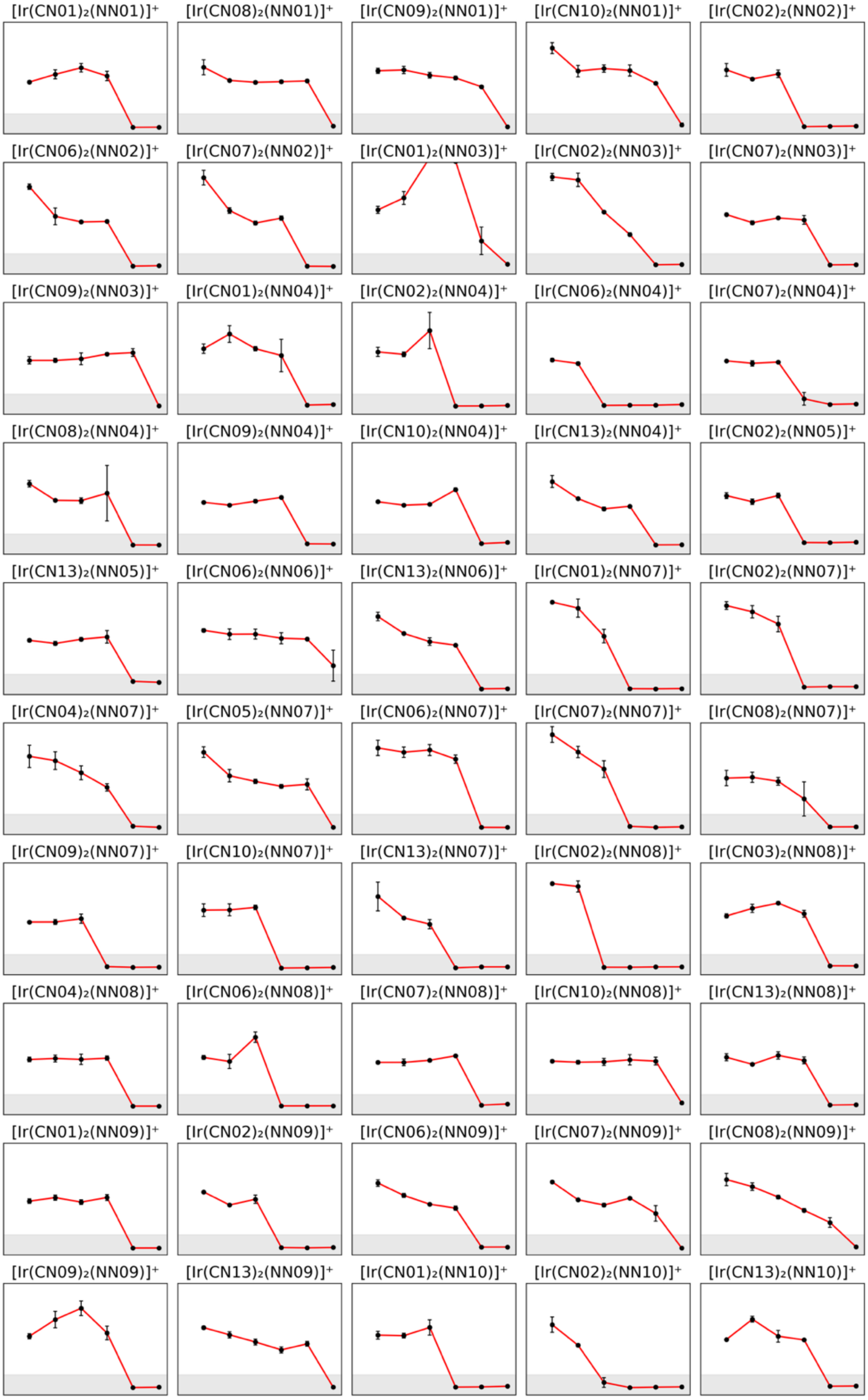

Figure S51: MIC fits for selected complexes from **HTS336** in B. Subtilis bacteria. Complexes were tested at 0.5, 1, 2, 4, 8 and 16 μM in triplicate, and kept in the dark. MICs were defined as the lowest concentration in which remaining viability was <20% (indicated by the grey area on the graph).

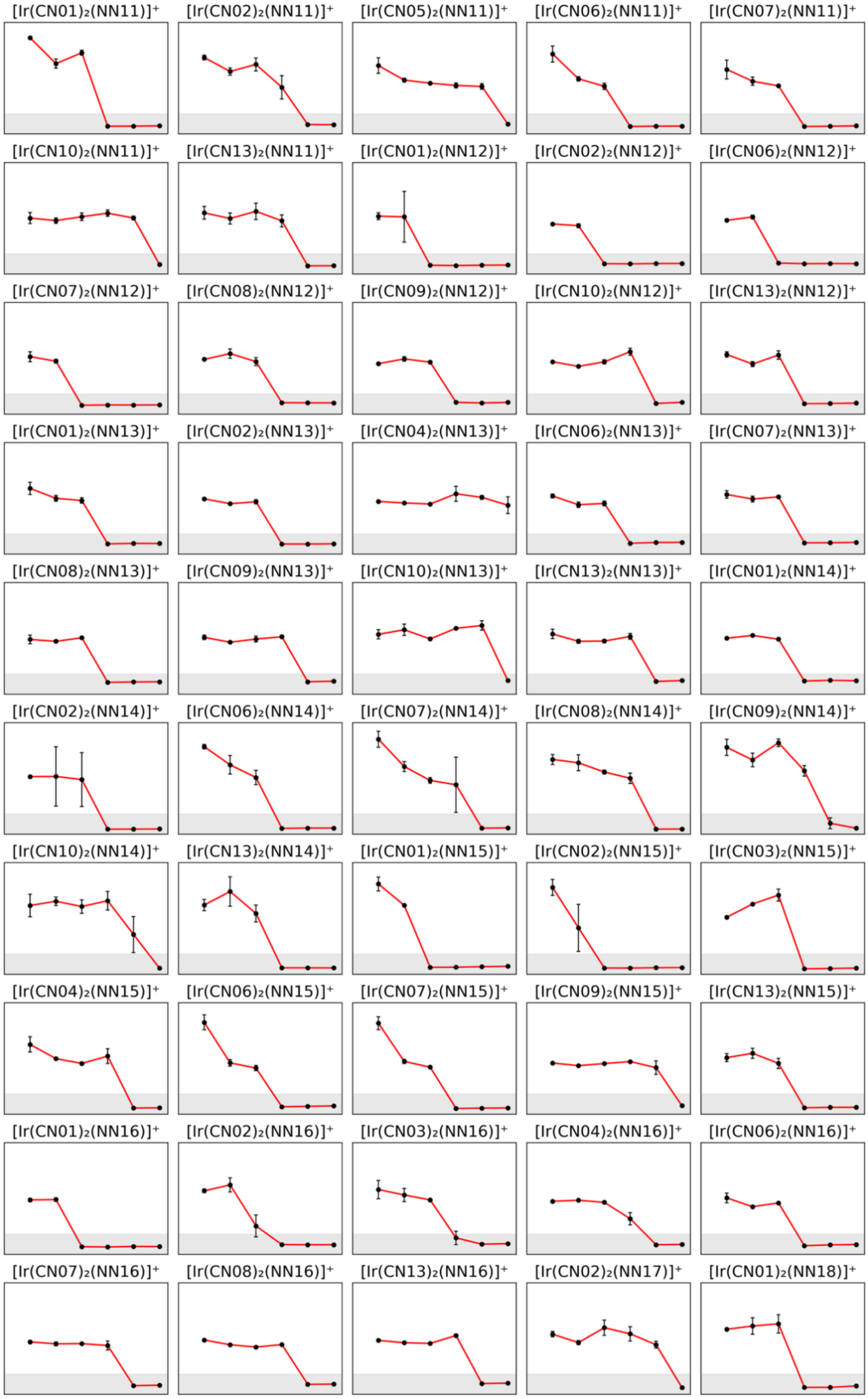

Figure S52: MIC fits for selected complexes from **HTS336** in B. Subtilis bacteria. Complexes were tested at 0.5, 1, 2, 4, 8 and 16 μM in triplicate, and kept in the dark. MICs were defined as the lowest concentration in which remaining viability was <20% (indicated by the grey area on the graph).

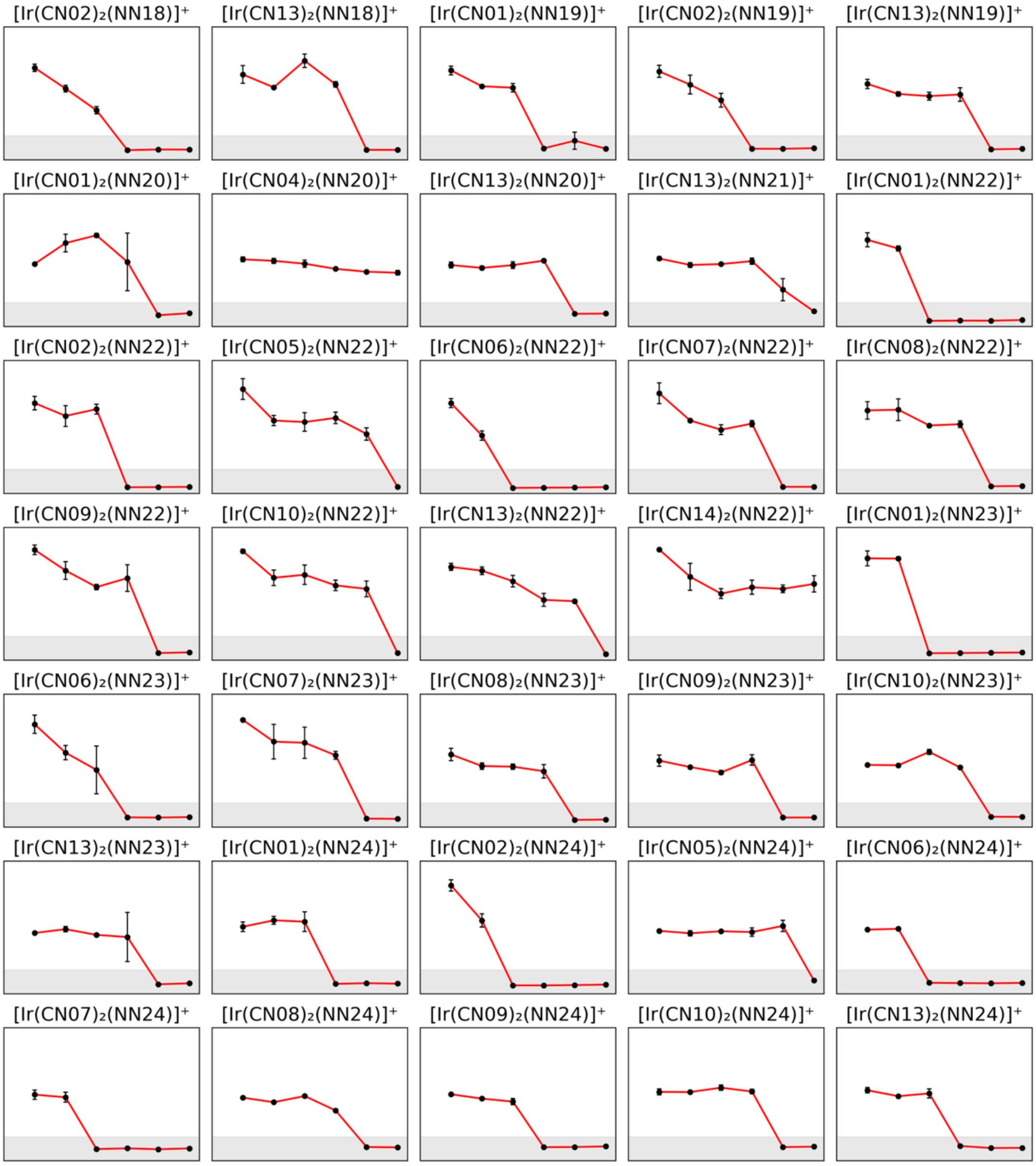

Figure S53: MIC fits for selected complexes from **HTS336** in B. Subtilis bacteria. Complexes were tested at 0.5, 1, 2, 4, 8 and 16 μM in triplicate, and kept in the dark. MICs were defined as the lowest concentration in which remaining viability was <20% (indicated by the grey area on the graph).

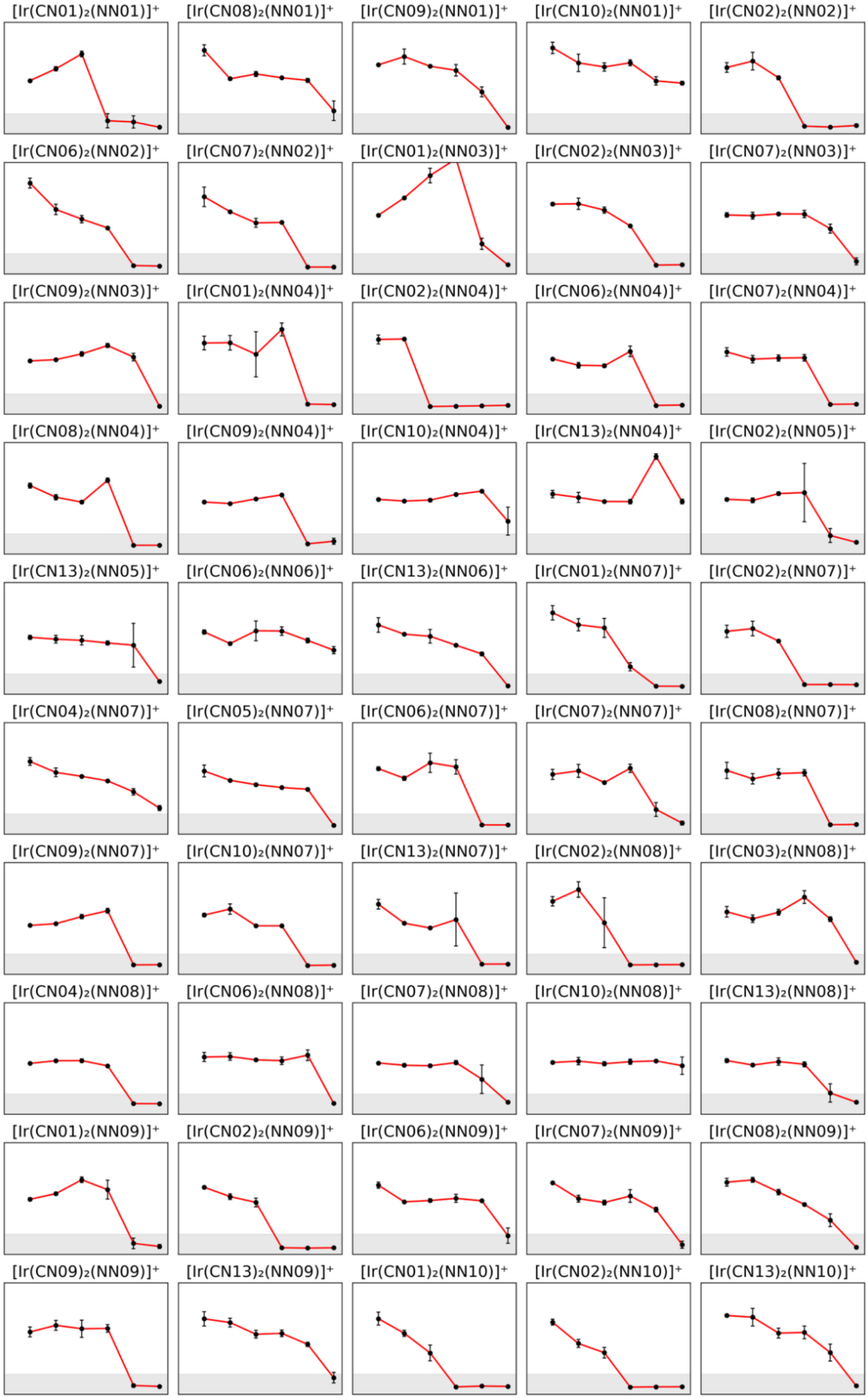

Figure S54: MIC fits for selected complexes from **HTS336** in B. Subtilis bacteria. Complexes were tested at 0.5, 1, 2, 4, 8 and 16 μM in triplicate, and irradiated for 12. Minutes using 15 mW/cm^2 of blue light (470 nm). MICs were defined as the lowest concentration in which remaining viability was <20% (indicated by the grey area on the graph).

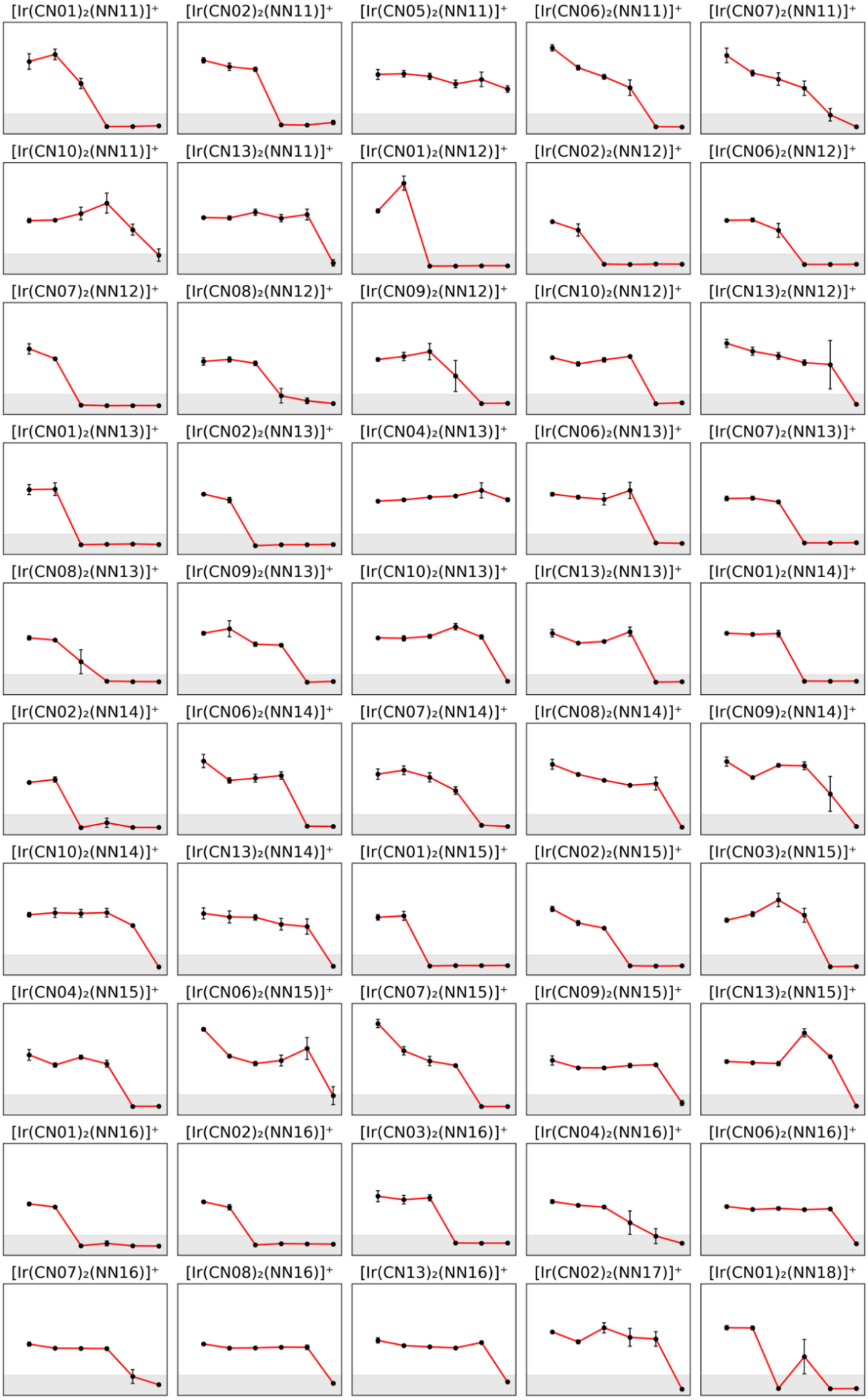

Figure S55: MIC fits for selected complexes from **HTS336** in B. Subtilis bacteria. Complexes were tested at 0.5, 1, 2, 4, 8 and 16 μM in triplicate, and irradiated for 12. Minutes using 15 mW/cm^2 of blue light (470 nm). MICs were defined as the lowest concentration in which remaining viability was <20% (indicated by the grey area on the graph).

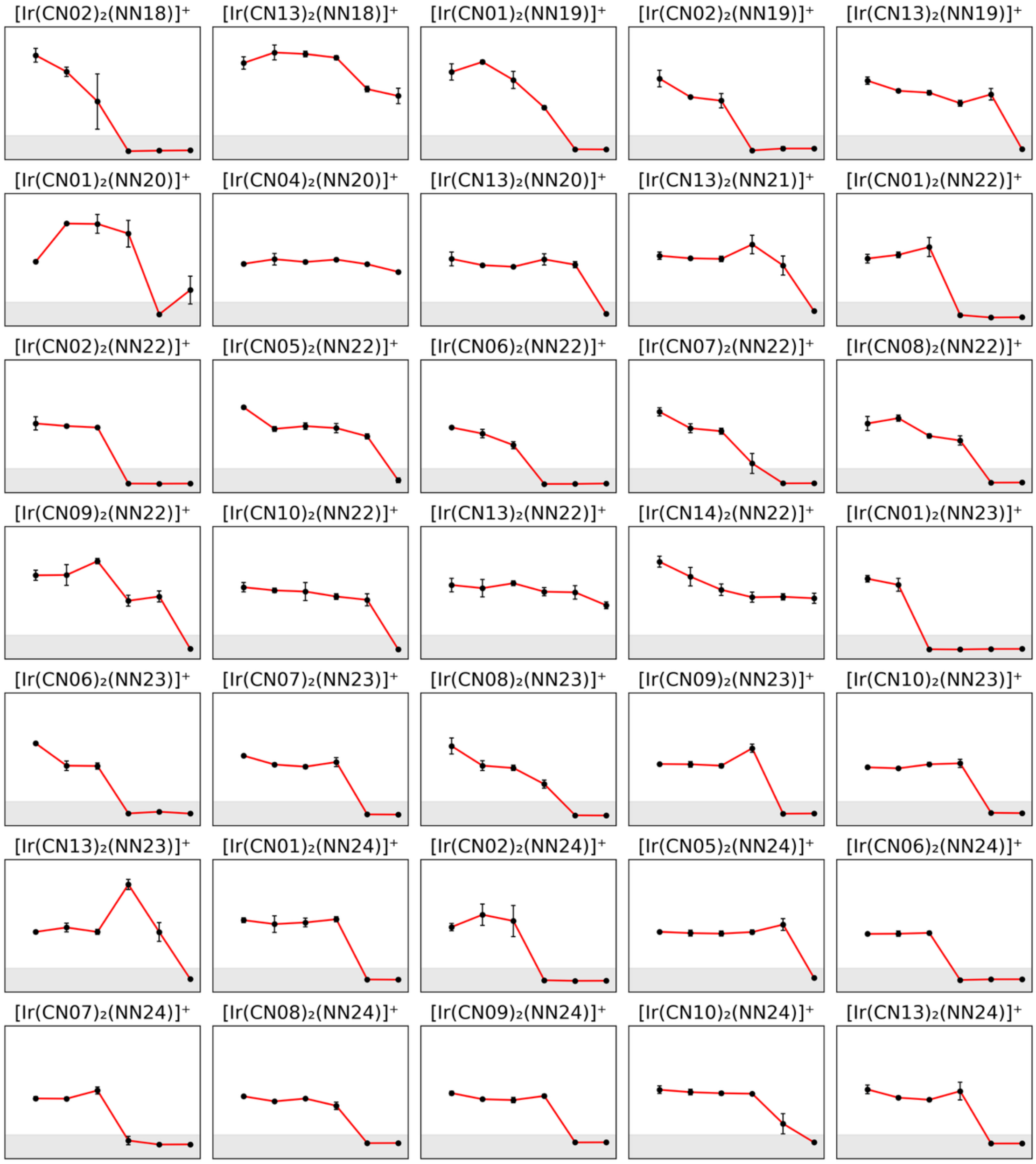

Figure S56: MIC fits for selected complexes from **HTS336** in B. Subtilis bacteria. Complexes were tested at 0.5, 1, 2, 4, 8 and 16 μM in triplicate, and irradiated for 12. Minutes using 15 mW/cm^2 of blue light (470 nm). MICs were defined as the lowest concentration in which remaining viability was <20% (indicated by the grey area on the graph).

	NN01	NN02	NN03	NN04	NN05	NN06	NN07	NN08	NN09	NN10	NN11	NN12	NN13	NN14	NN15	NN16	NN17	NN18	NN19	NN20	NN21	NN22	NN23	NN24
CN01	0.63		0.68	1.30	0.36			0.35	0.30	0.48	0.58			0.58	1.23	1.09	2.43	0.55	0.65	0.38				1.36
CN02			1.10	2.14	0.79		0.69	0.64	0.48	0.77	0.82			0.54	1.93									0.91
CN03																					2.80			
CN04	0.38		0.35		0.40		0.63	0.23	0.35	0.42	0.57							0.49	0.60	0.31			0.88	
CN05							1.16																	0.46
CN06		0.51	1.00	0.41			0.45	0.68	0.37		0.43	1.03		1.16	0.61	1.03							0.74	
CN07	0.51		0.46	0.57	0.88		1.10	0.38	1.66	0.93	0.35		0.69	0.38	1.10									0.36
CN08	0.76	1.62	2.44	0.98			1.09	0.45	0.36	1.43	0.98			0.75	0.66	0.64			1.93					
CN09	1.74		1.24	0.51	2.39		0.80	0.74	0.47		0.68		0.43	0.37										0.20
CN10			2.32	0.88			0.70	1.39	0.70		0.98		0.90	0.60										0.25
CN11																								
CN12														2.76										
CN13	1.08		2.62	0.60	2.49		0.50	2.11	0.55	1.28	0.85		1.14	0.35	0.84								2.38	
CN14																								

Figure S57: IC_{50} values for selected complexes from **HTS336** in AT3 cells.

	NN01	NN02	NN03	NN04	NN05	NN06	NN07	NN08	NN09	NN10	NN11	NN12	NN13	NN14	NN15	NN16	NN17	NN18	NN19	NN20	NN21	NN22	NN23	NN24
CN01			1.37	1.53			1.25		0.72	0.96	1.74			1.06	1.34	0.86			1.59	0.42				
CN02								1.13		2.21	1.16			1.37						1.13				1.14
CN03																				1.13				
CN04	1.32							0.89	0.46	0.74										0.69				
CN05																								
CN06		1.06	1.80	0.70			0.40	0.83	0.53		0.70	0.98	0.48	0.39	0.91								0.90	0.21
CN07	1.16		0.97	0.86	1.11			0.67	0.60	1.61	0.49			0.45					2.07					0.63
CN08							0.98		1.16		1.41		0.98	1.21										
CN09			2.22	1.09					0.94		1.11			0.72										
CN10									1.39		2.11		0.92	1.07										
CN11																								
CN12																								
CN13				1.75			0.74		2.07		1.21		0.96	0.58										
CN14																								

Figure S58: IC_{50} values for selected complexes from **HTS336** in HEK cells.

	NN01	NN02	NN03	NN04	NN05	NN06	NN07	NN08	NN09	NN10	NN11	NN12	NN13	NN14	NN15	NN16	NN17	NN18	NN19	NN20	NN21	NN22	NN23	NN24
CN01	0.73		1.58	1.04	0.51		0.82		0.86	0.51	1.23		1.75	0.99		1.97				0.26	0.63			
CN02			2.04	2.12	1.21		0.75	0.62			1.24		0.71	1.04										1.28
CN03																				1.90	0.99			
CN04	0.89		0.51		0.52		1.18	0.32	0.57	0.58	1.44								1.05	0.27			0.53	
CN05							0.88																	1.60
CN06	1.41	0.79	0.50	0.30	0.91		0.22	0.58	0.82		0.61	0.93	0.60	0.22	0.70	1.52							0.54	0.19
CN07	0.25		0.36	0.39	0.63		0.57	0.79	0.59	0.47	0.47		1.60	0.08	1.50			1.77						0.51
CN08	0.76			0.82	0.94		0.91		0.48	0.00	2.17		1.07	0.84	0.80									0.82
CN09				0.60			0.28	0.66	0.43		1.55		0.50	0.73										0.28
CN10							0.43		1.96					0.76										1.43
CN11																								
CN12																								0.34
CN13							2.09	0.60	0.97		2.01		0.72	1.04										
CN14																								

Figure S59: IC_{50} values for selected complexes from **HTS336** in HeLa cells

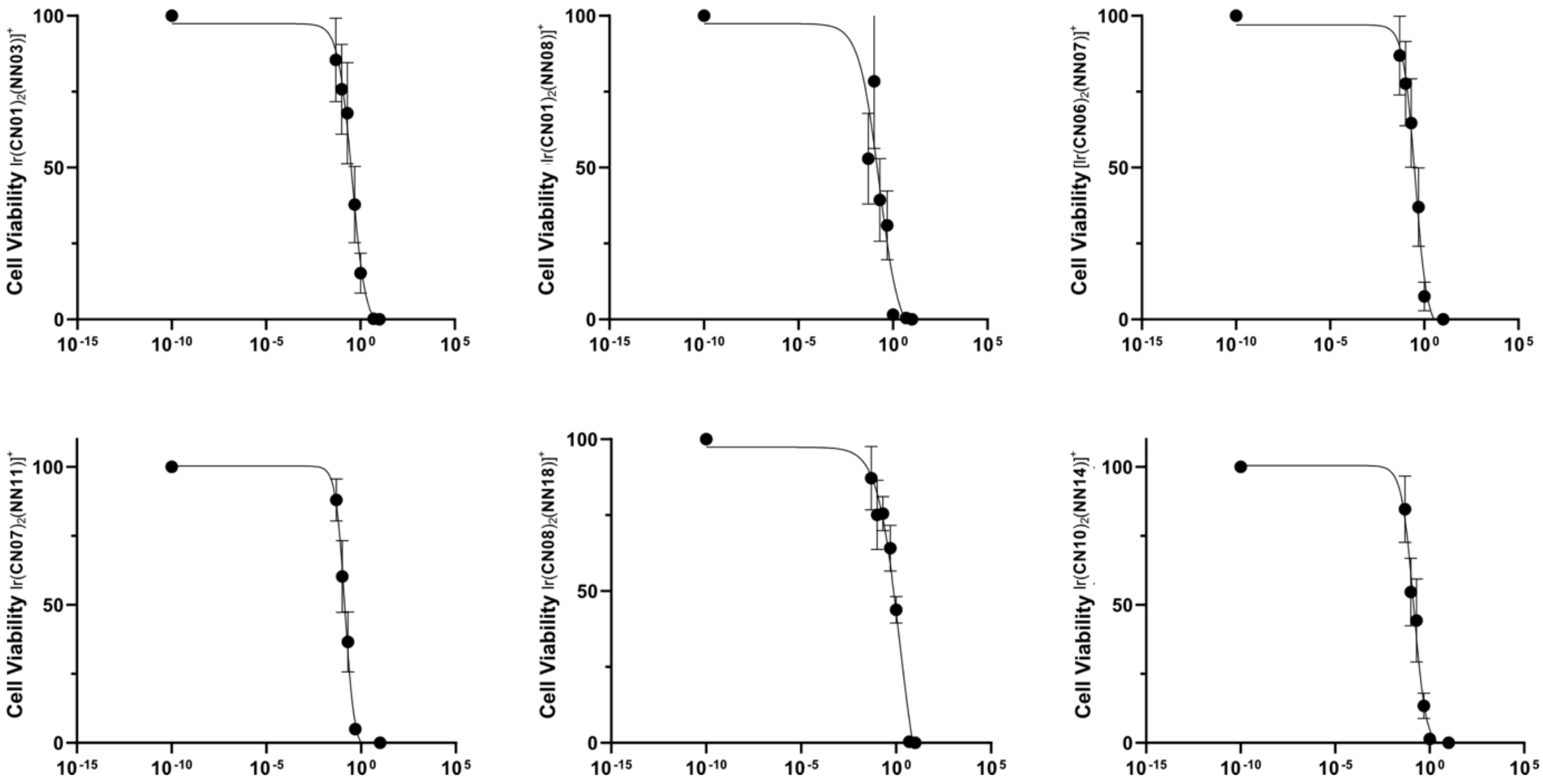

Figure S60: IC50 curves for MCA205 fibrosarcoma cells, with a 4 hour complex incubation, followed by medium exchange and irradiation (450 nm, 1.2 J/cm², 10 min). After a further 24 h, cell viability was assessed by MTT assay (absorbance at 550 nm, ref. 620 nm).

Table S4: Results of MTT assays, given as IC_{50} values in µM, with an incubation period of 24 h. IC_{50} values ± SED were determined from three biological replicates. Cisplatin was used as a positive control and to check cell conformity.

complex	*[Ir(**CN01**)$_2$(**NN03**)]$^+$*	*[Ir(**CN01**)$_2$(**NN08**)]$^+$*	*[Ir(**CN06**)$_2$(**NN07**)]$^+$*	*[Ir(**CN07**)$_2$(**NN11**)]$^+$*	*[Ir(**CN08**)$_2$(**NN18**)]$^+$*	*[Ir(**CN10**)$_2$(**NN14**)]$^+$*
IC_{50}	**0.352 ± 0.208**	**0.369 ± 0.167**	**0.329 ± 0.101**	**0.140 ± 0.021**	**2.243 ± 1.027**	**0.145 ± 0.035**

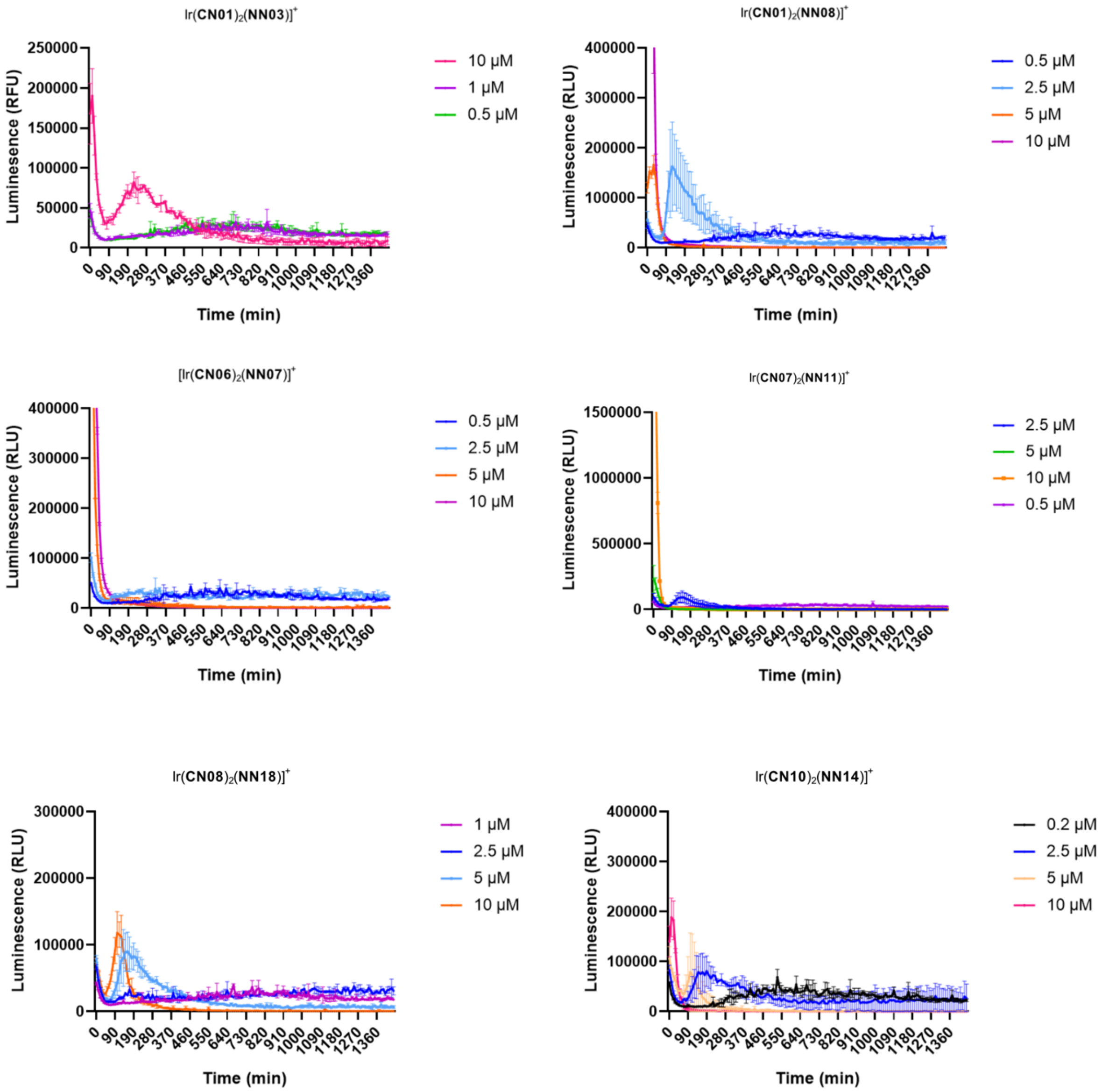

Figure S61: Extracellular ATP results. Untreated MCA205 cells served as controls. The mean luminescence values and SD from three replicates are shown.

Table S5: HMGB-1 levels were quantified in the cell culture medium after 24 h incubation with the indicated compound concentrations, following pre-incubation and irradiation.

compound]/μM	*[Ir(**CN01**)$_2$(**NN03**)]$^+$*	*[Ir(**CN01**)$_2$(**NN08**)]$^+$*	*[Ir(**CN06**)$_2$(**NN07**)]$^+$*	*[Ir(**CN07**)$_2$(**NN11**)]$^+$*		*[Ir(**CN08**)$_2$(**NN18**)]$^+$*	*[Ir(**CN10**)$_2$(**NN14**)]$^+$*
0.05	**-**	**-**	**-**	**-**		**-**	**-**
0.2	-	-	-	-		-	-
0.5	-	-	-	234.28 ng/mL		-	-
1	543.52 ng/mL	350.25 ng/mL	400.04 ng/mL	219.87 ng/mL		185.80 ng/mL	395.46 ng/mL
2.5		283.42 ng/mL	110.22 ng/mL	-	257.87 ng/mL	244.11 ng/mL	157.62 ng/mL
5		179.90 ng/mL	128.15 ng/mL	-	113.01 ng/mL	222.49 ng/mL	193.66 ng/mL

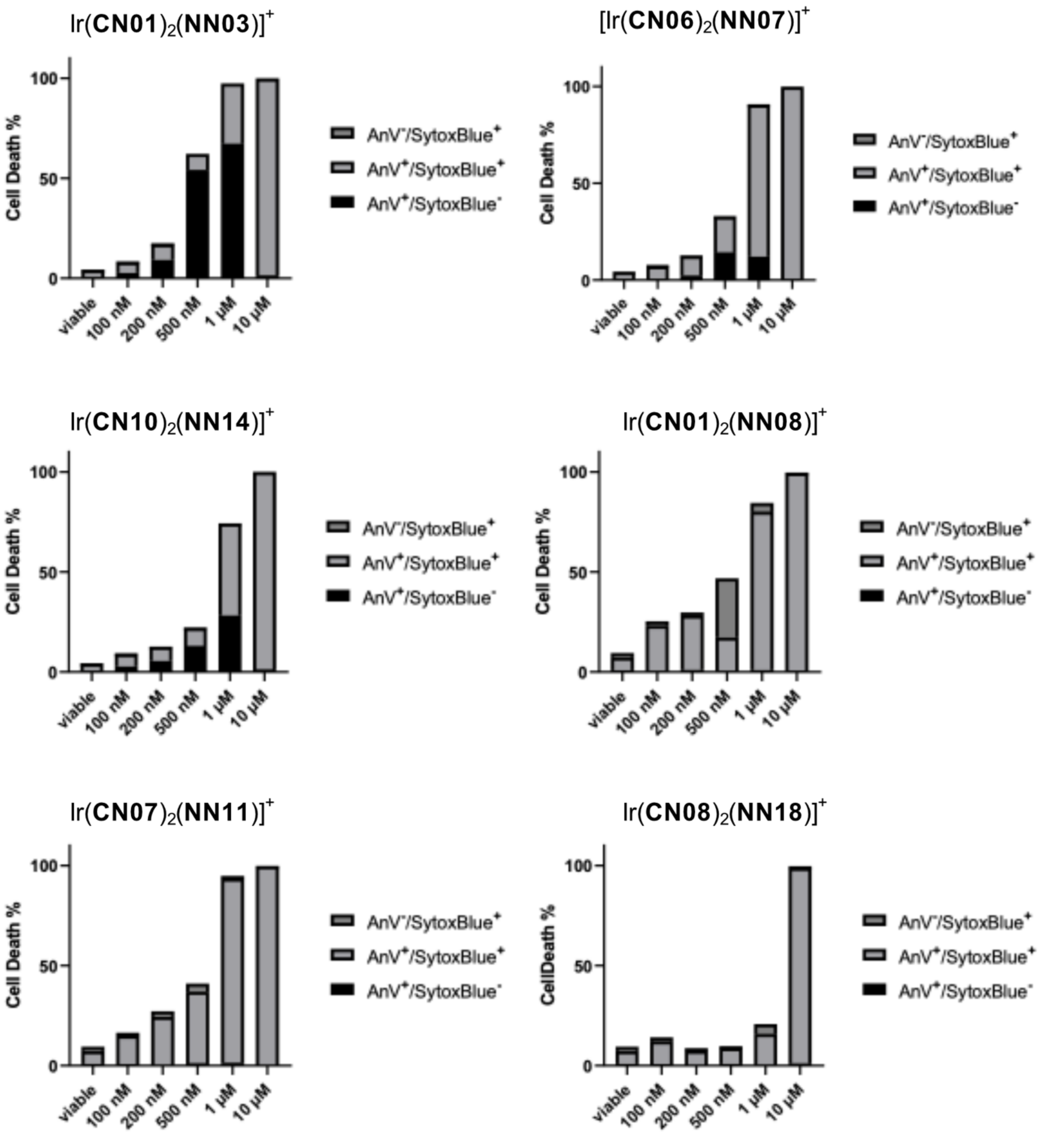

Figure S62: Cell Death assay. After 4 h of incubation and 20 % irradiation, followed by 24 h incubation. Annexin V-FITC and Sytox Blue double staining were performed to identify the lowest effective concentration for subsequent CRT experiments.

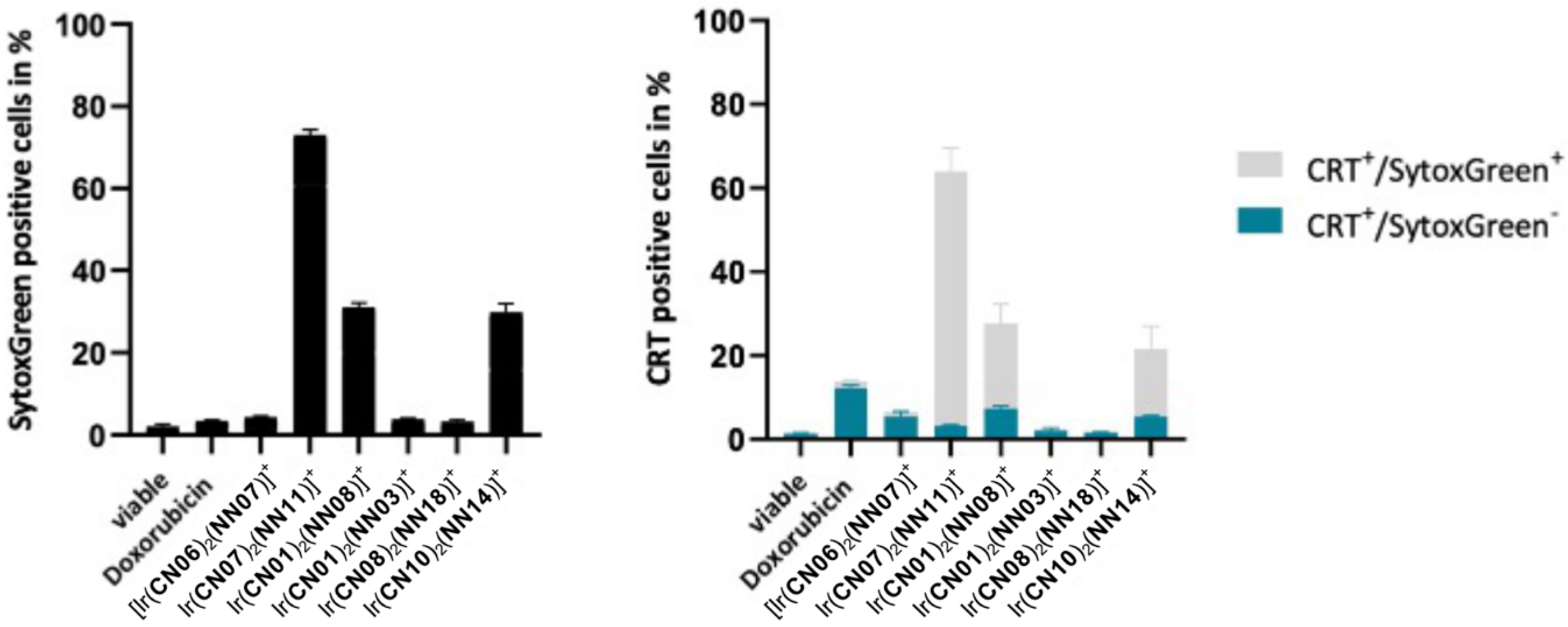

Figure S63: Left: Percentage of dead MCA205 cells after treatment with doxorubicin (4 µM) or the indicated complexes (1 µM). Right: CRT-positive MCA205 cells after the same treatments. After incubation, cells were stained with a fluorescently labeled anti-CRT antibody and a live/dead dye. Data are presented as mean ± SD from three independent experiments.

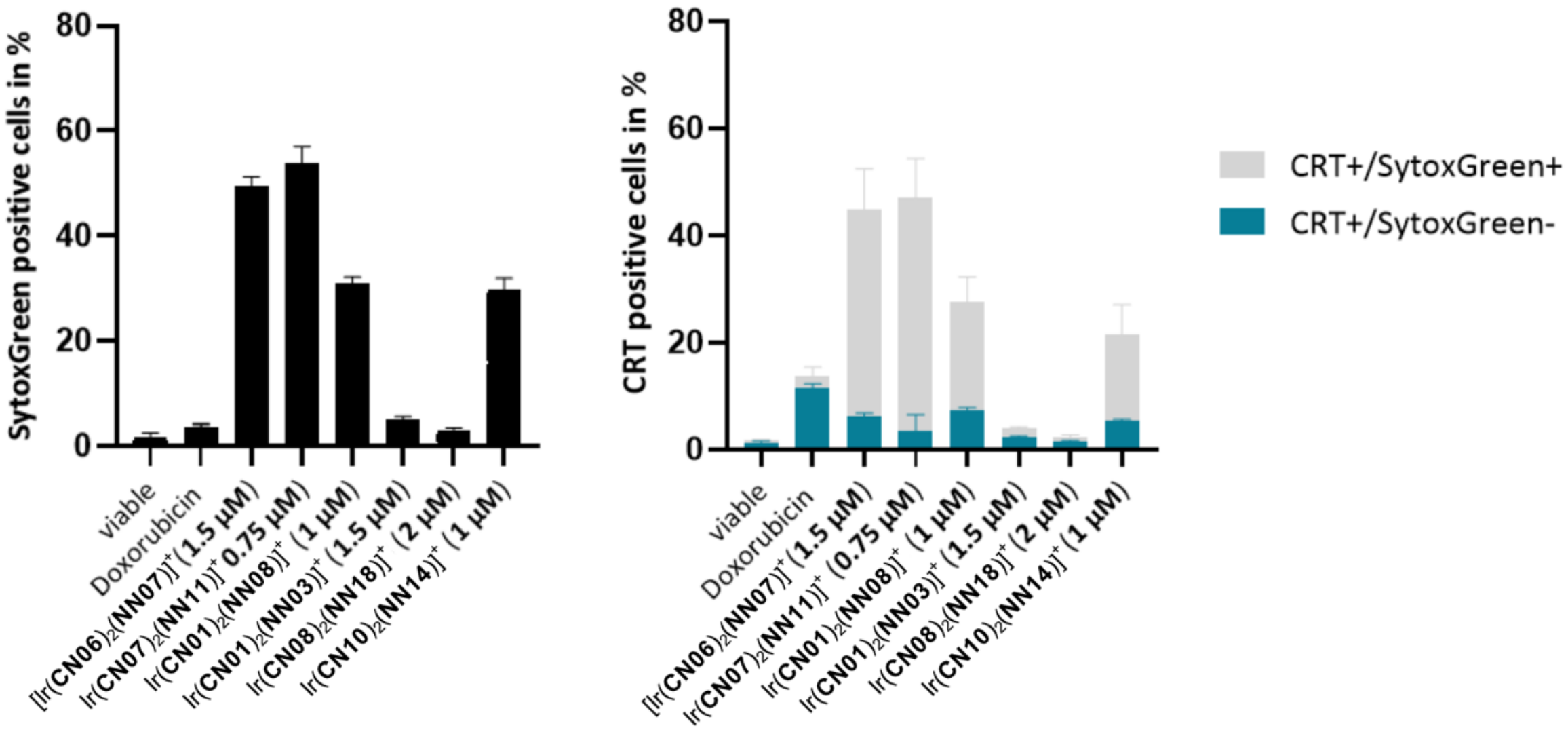

Figure S64: Left: Percentage of dead MCA205 cells after treatment with doxorubicin (4 µM) or the indicated complexes at their indicated concentration. Right: CRT-positive MCA205 cells after the same treatments. After incubation, cells were stained with a fluorescently labeled anti-CRT antibody and a live/dead dye. Data are presented as mean ± SD from three independent experiments.

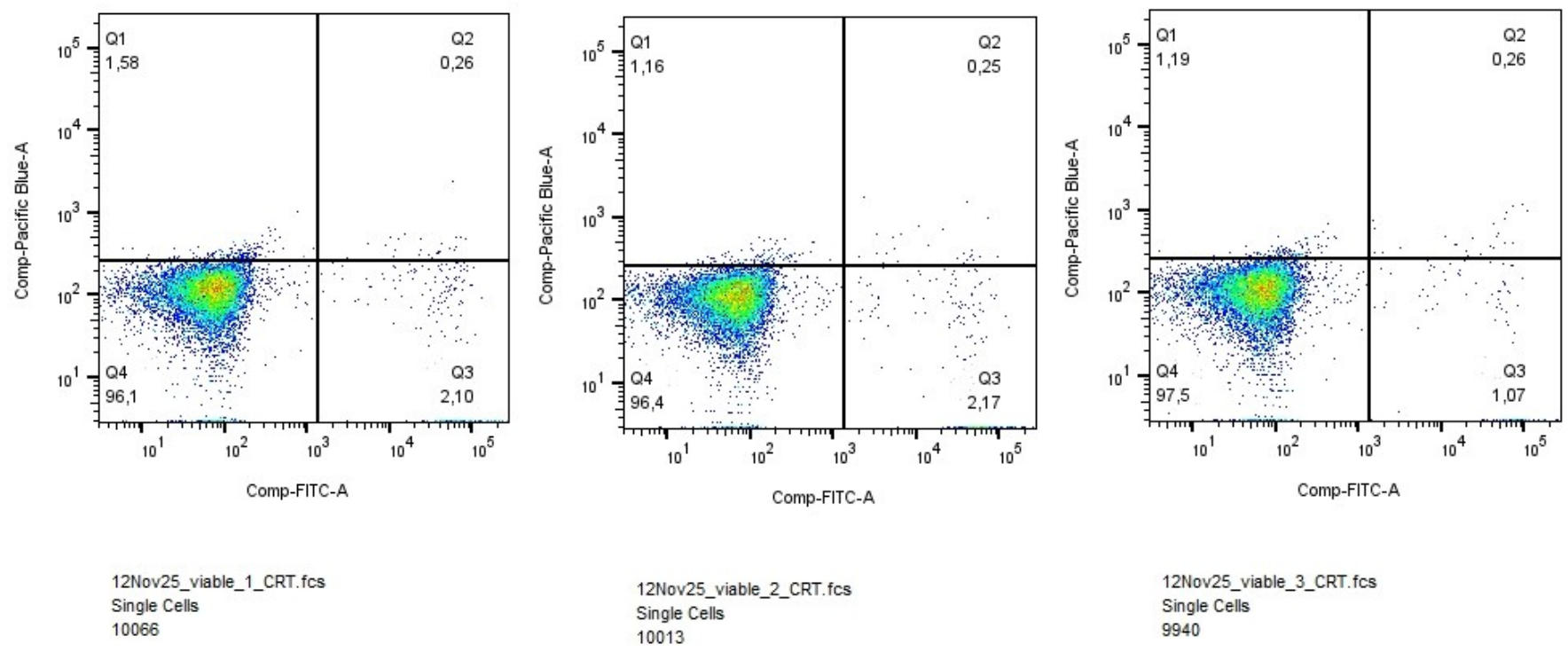

Figure S65: Flow cytometry dot plots showing CRT surface expression versus cell viability, viable results only.

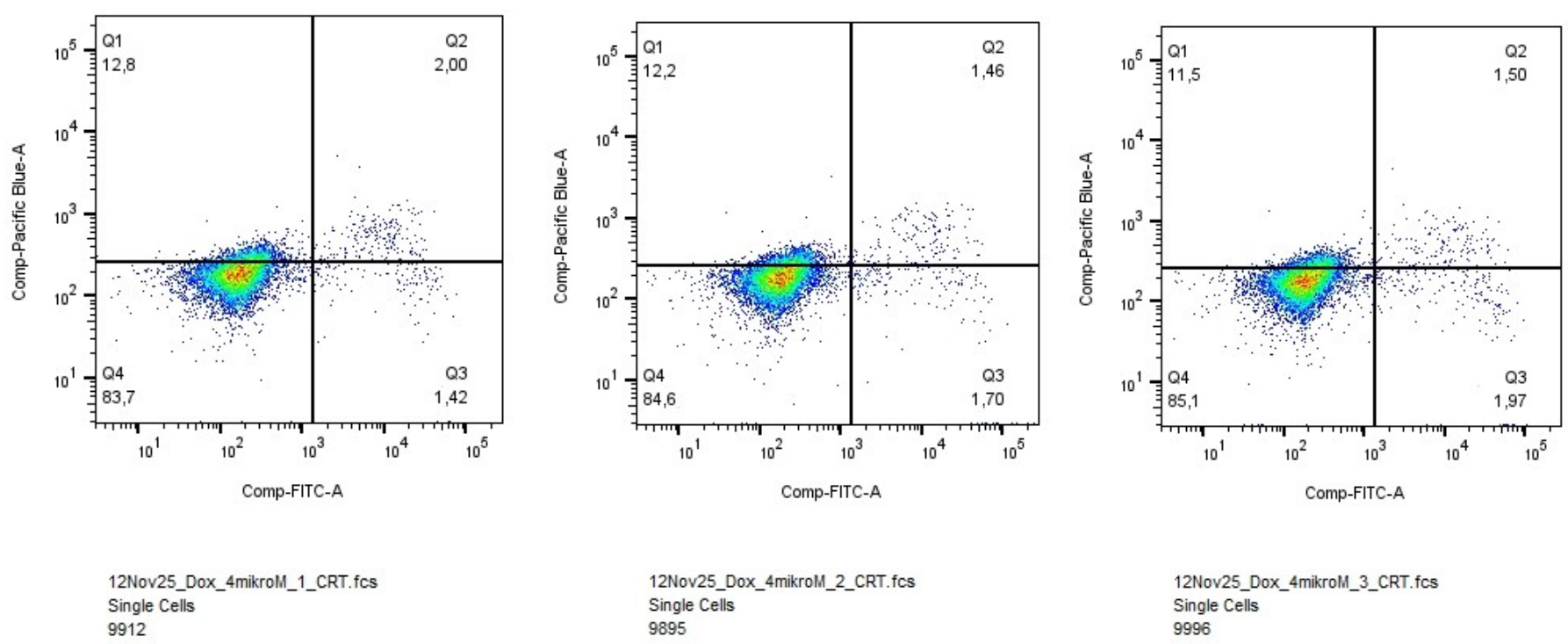

Figure S66: Flow cytometry dot plots showing CRT surface expression versus cell viability, after treatment with Doxorubicin (4 μM).

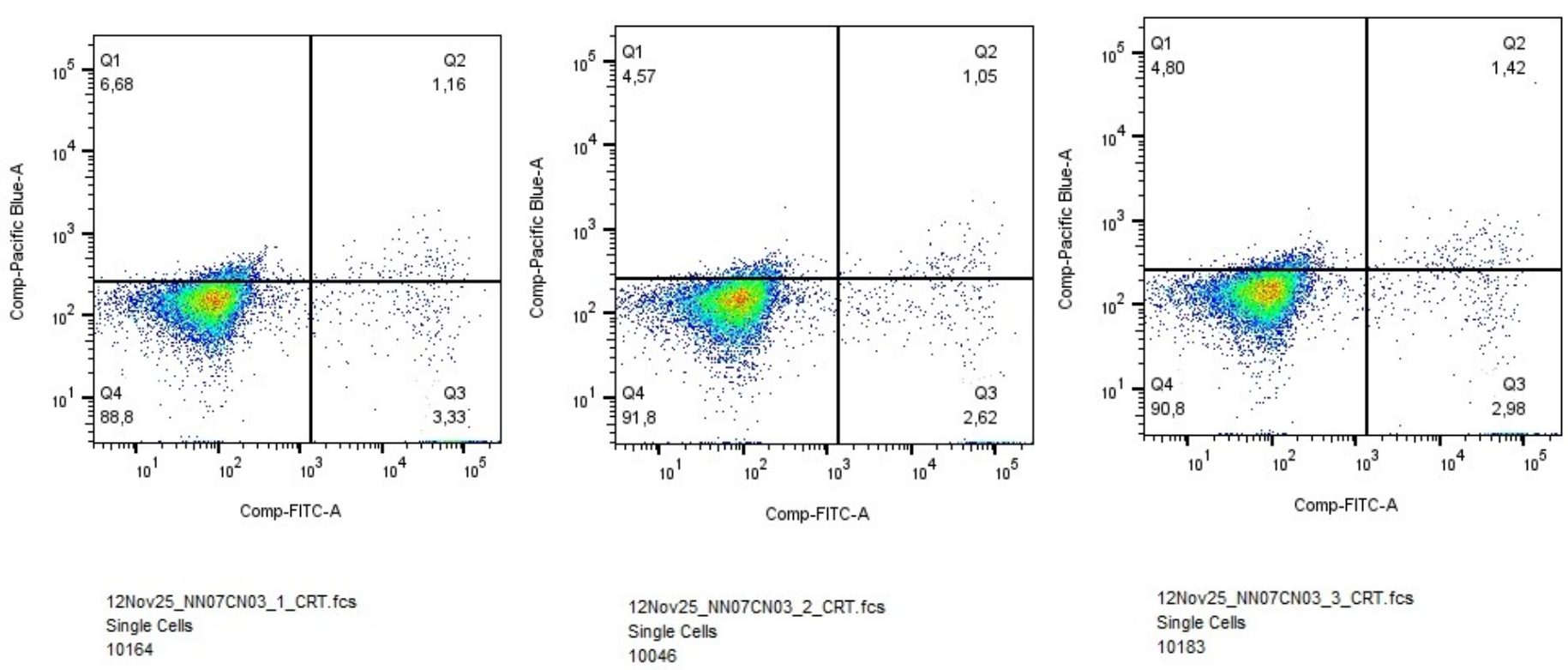

Figure S67: Flow cytometry dot plots showing CRT surface expression versus cell viability, after treatment with $[Ir(CN06)_2(NN07)]^+$ (1 μM).

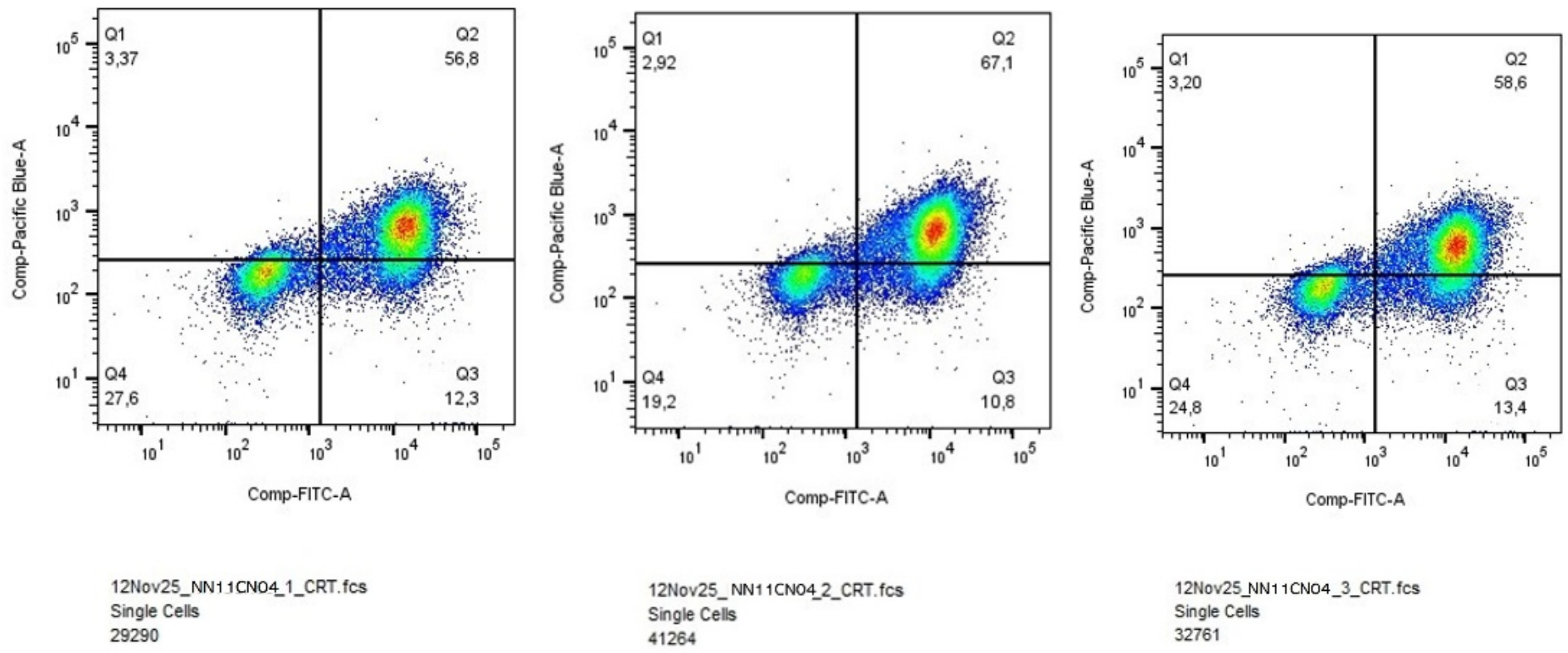

Figure S68: Flow cytometry dot plots showing CRT surface expression versus cell viability, after treatment with $[Ir(CN07)_2(NN11)]^+$ (1 µM).

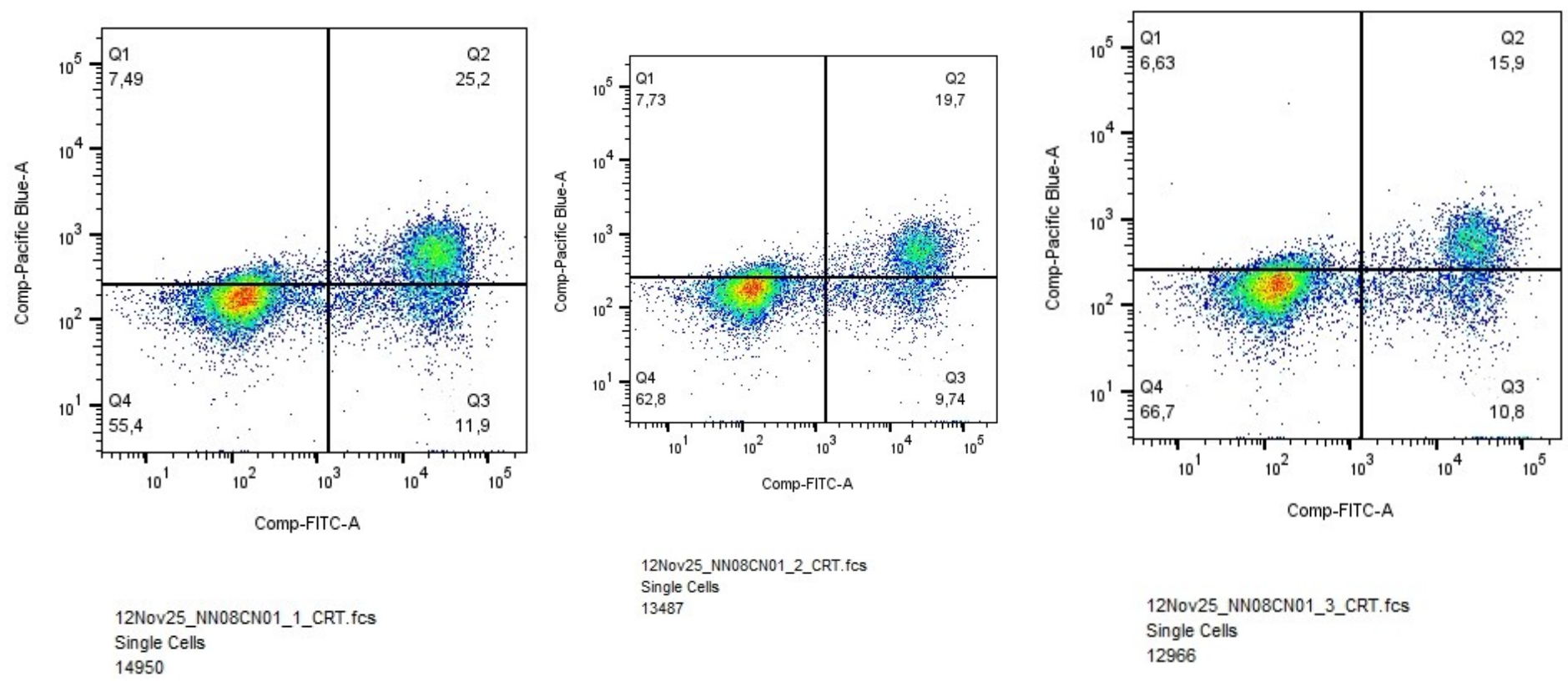

Figure S69: Flow cytometry dot plots showing CRT surface expression versus cell viability, after treatment with $[Ir(CN01)_2(NN08)]^+$ (1 µM).

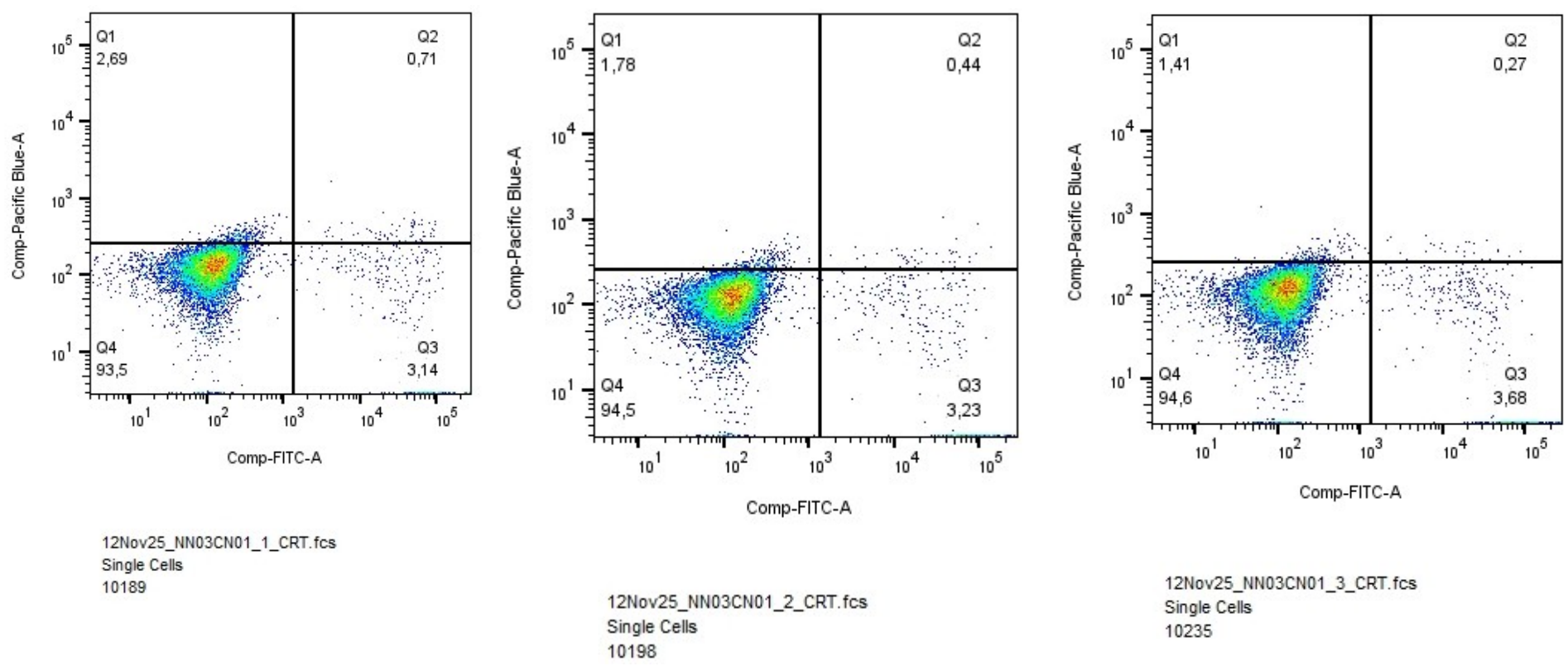

Figure S70: Flow cytometry dot plots showing CRT surface expression versus cell viability, after treatment with $[Ir(CN01)_2(NN03)]^+$ (1 µM).

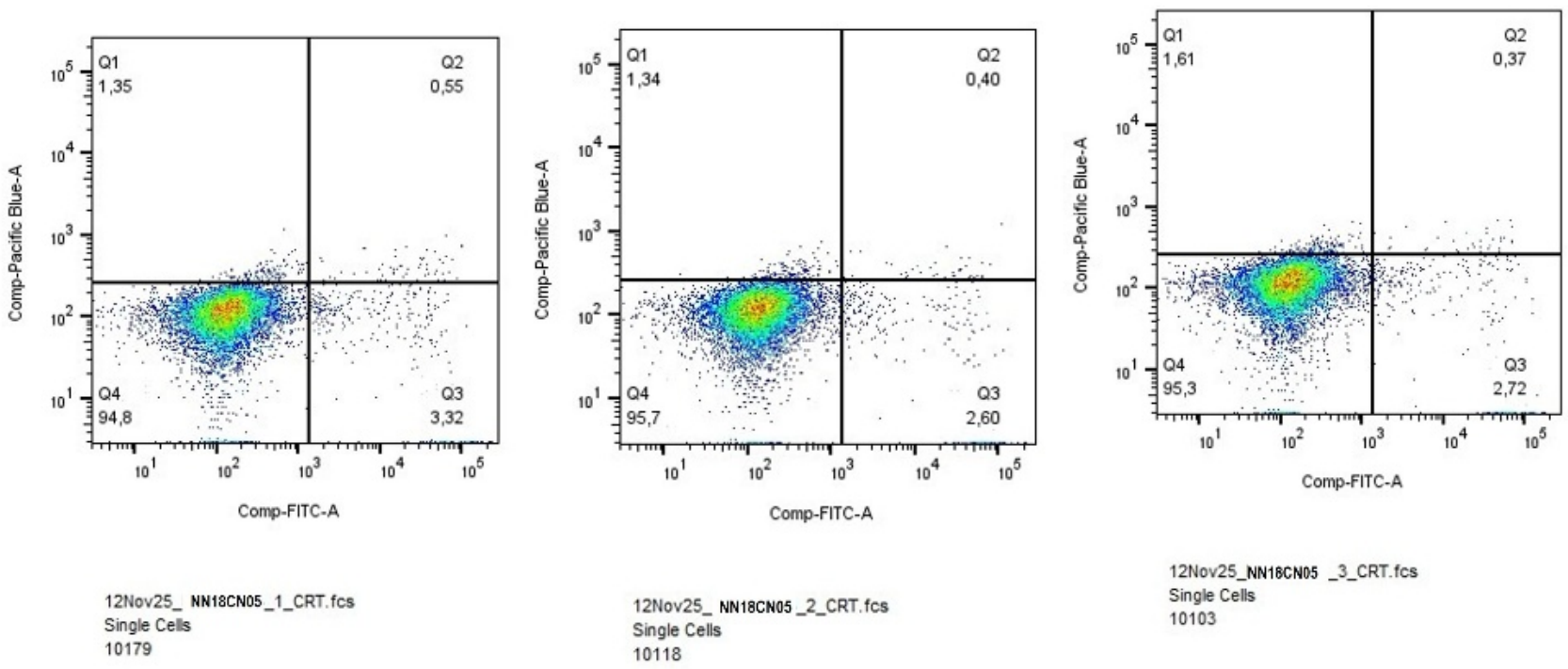

Figure S71: Flow cytometry dot plots showing CRT surface expression versus cell viability, after treatment with $[Ir(CN08)_2(NN18)]^+$ (1 μM).

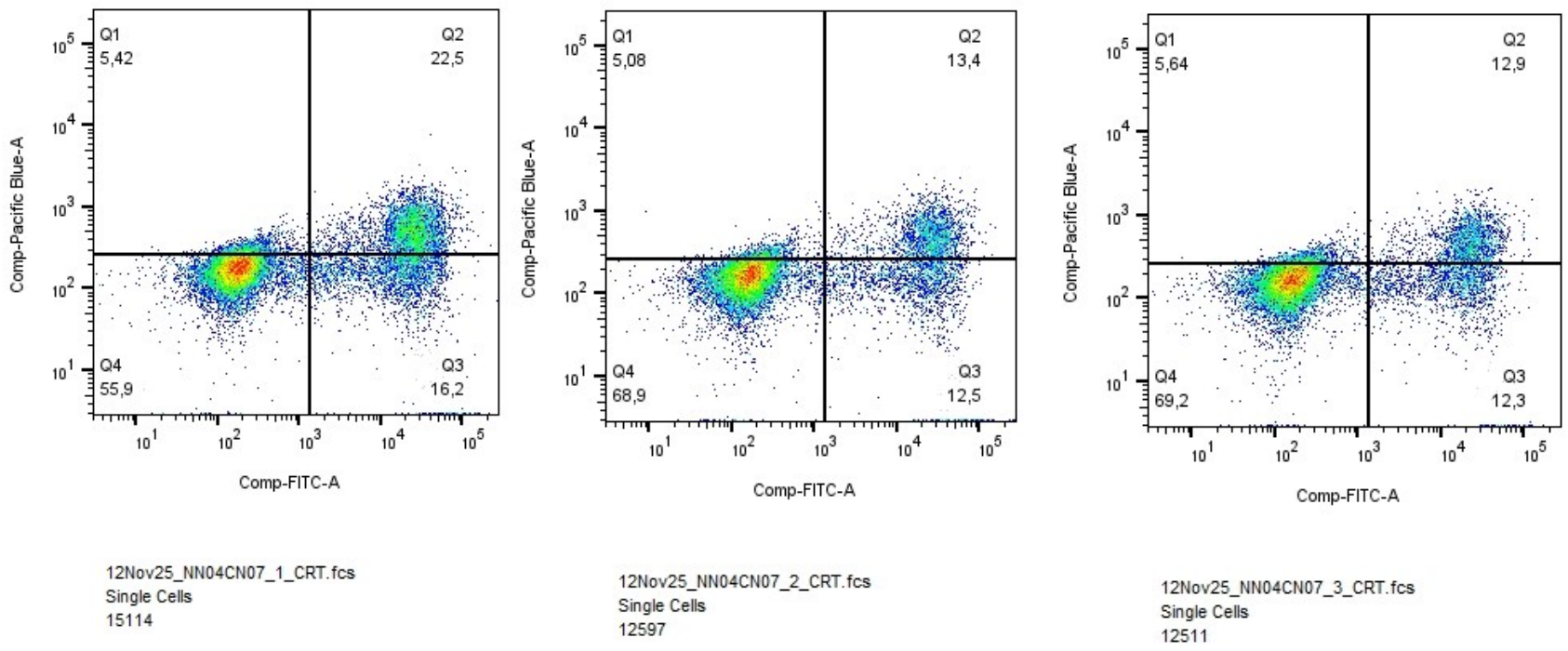

Figure S72: Flow cytometry dot plots showing CRT surface expression versus cell viability, after treatment with $[Ir(CN10)_2(NN14)]^+$ (1 μM).

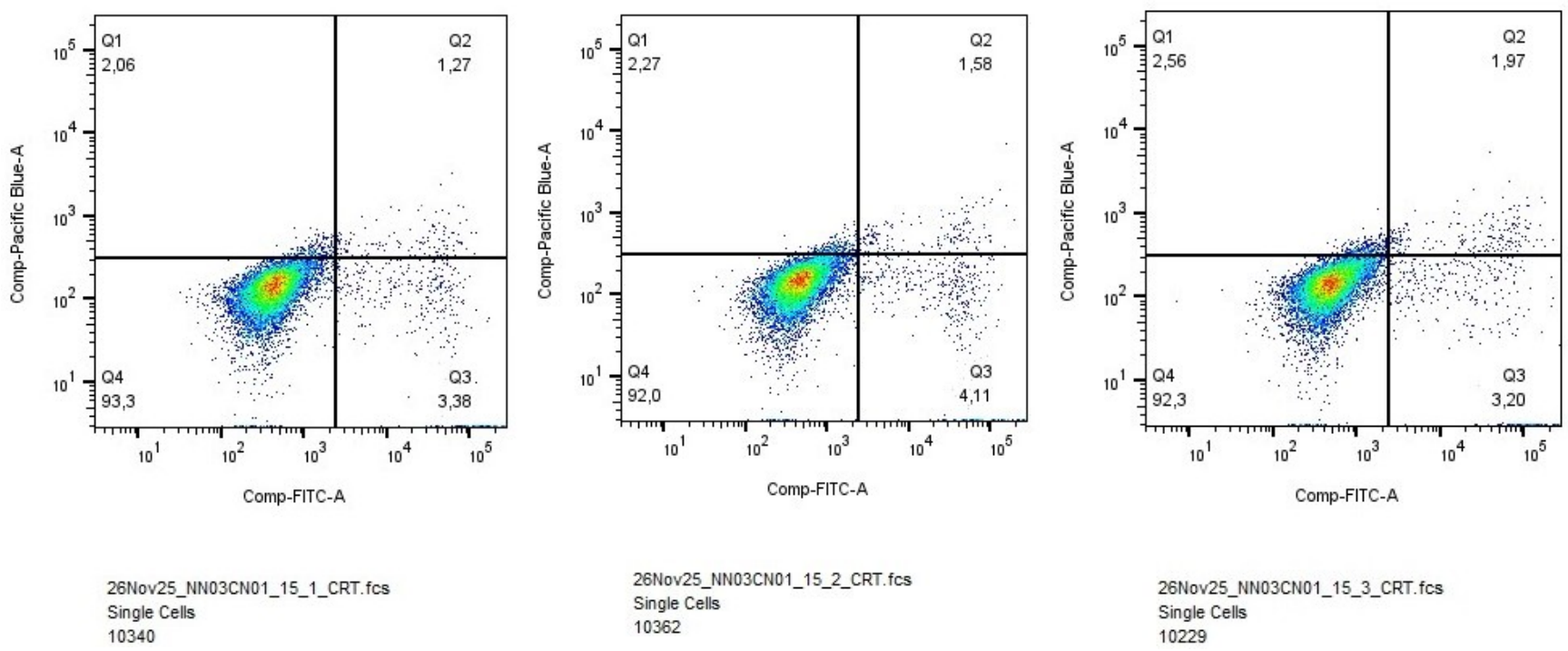

Figure S73: Flow cytometry dot plots showing CRT surface expression versus cell viability, after treatment with $[Ir(CN01)_2(NN03)]^+$ (1.5 μM).

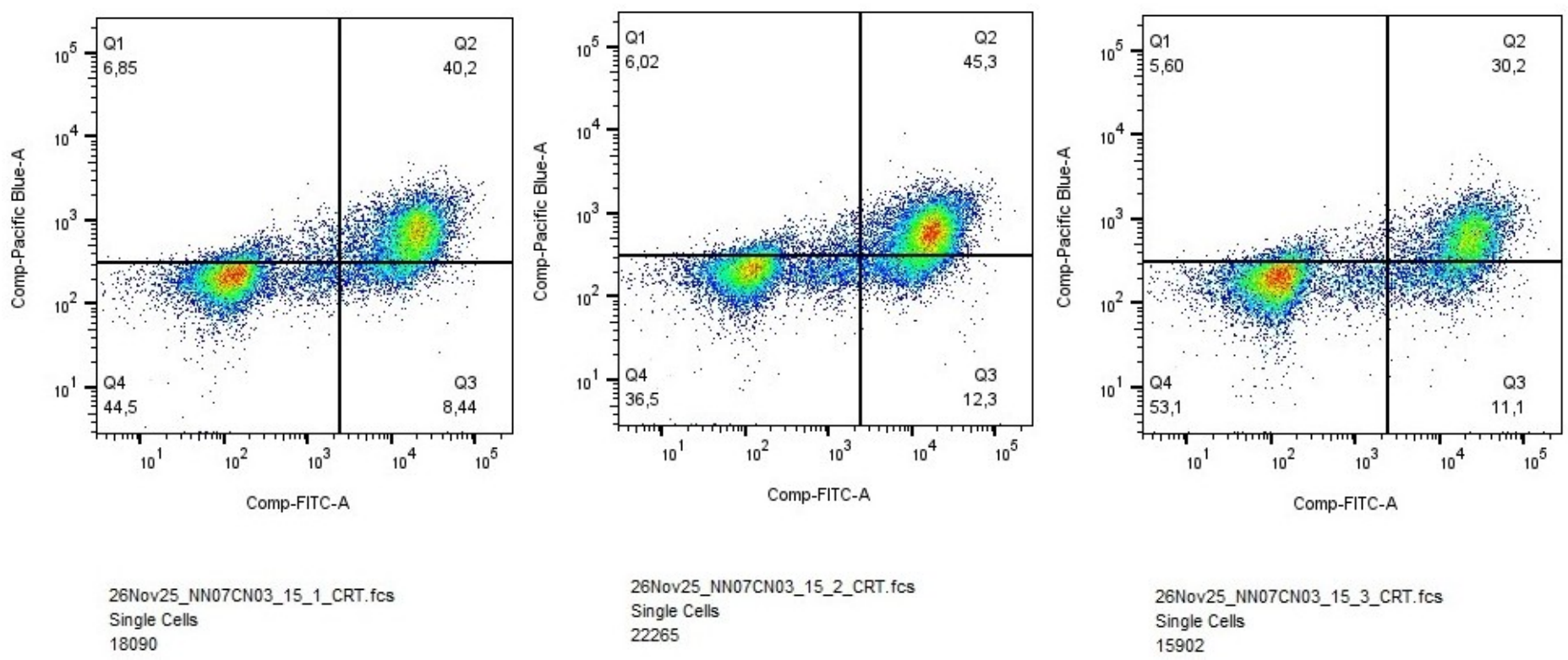

Figure S74: Flow cytometry dot plots showing CRT surface expression versus cell viability, after treatment with $[Ir(CN06)_2(NN07)]^+$ (1.5 μM).

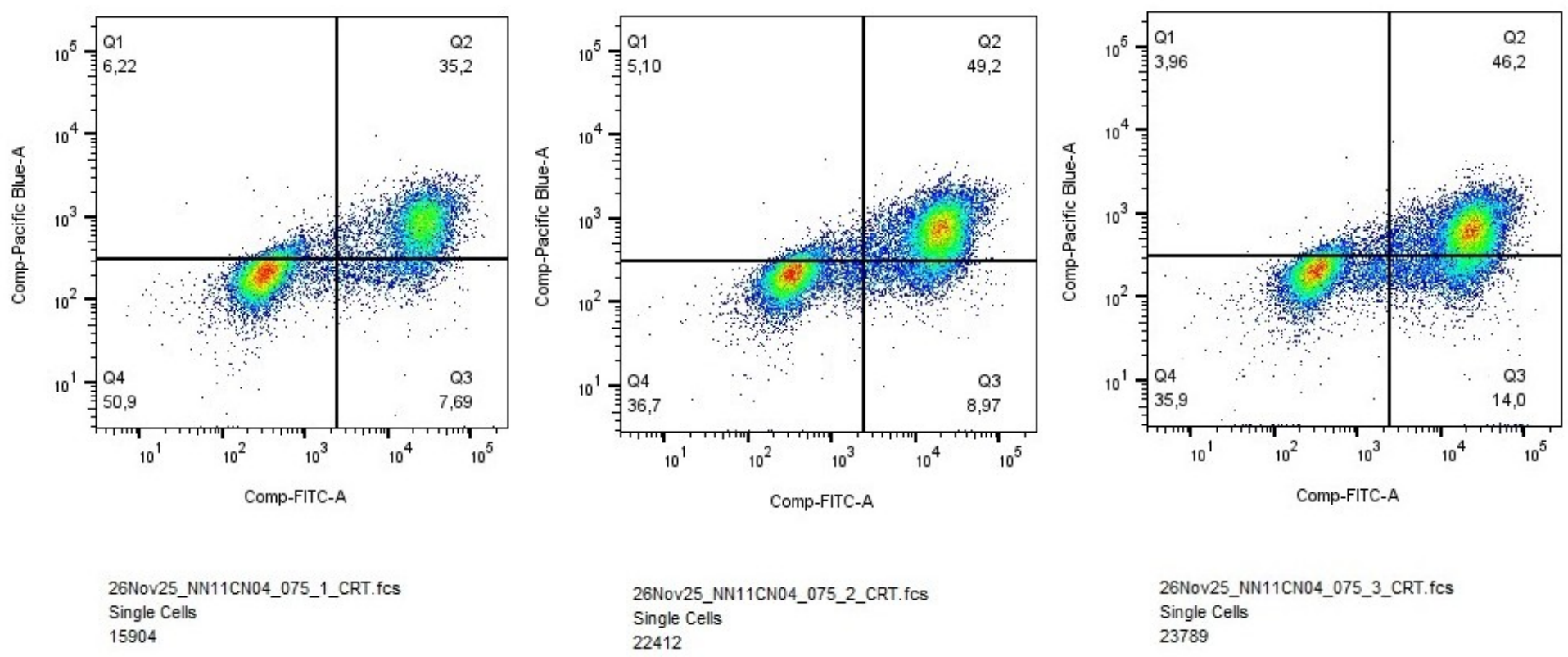

Figure S75: Flow cytometry dot plots showing CRT surface expression versus cell viability, after treatment with $[Ir(CN07)_2(NN11)]^+$ (0.7 μM).

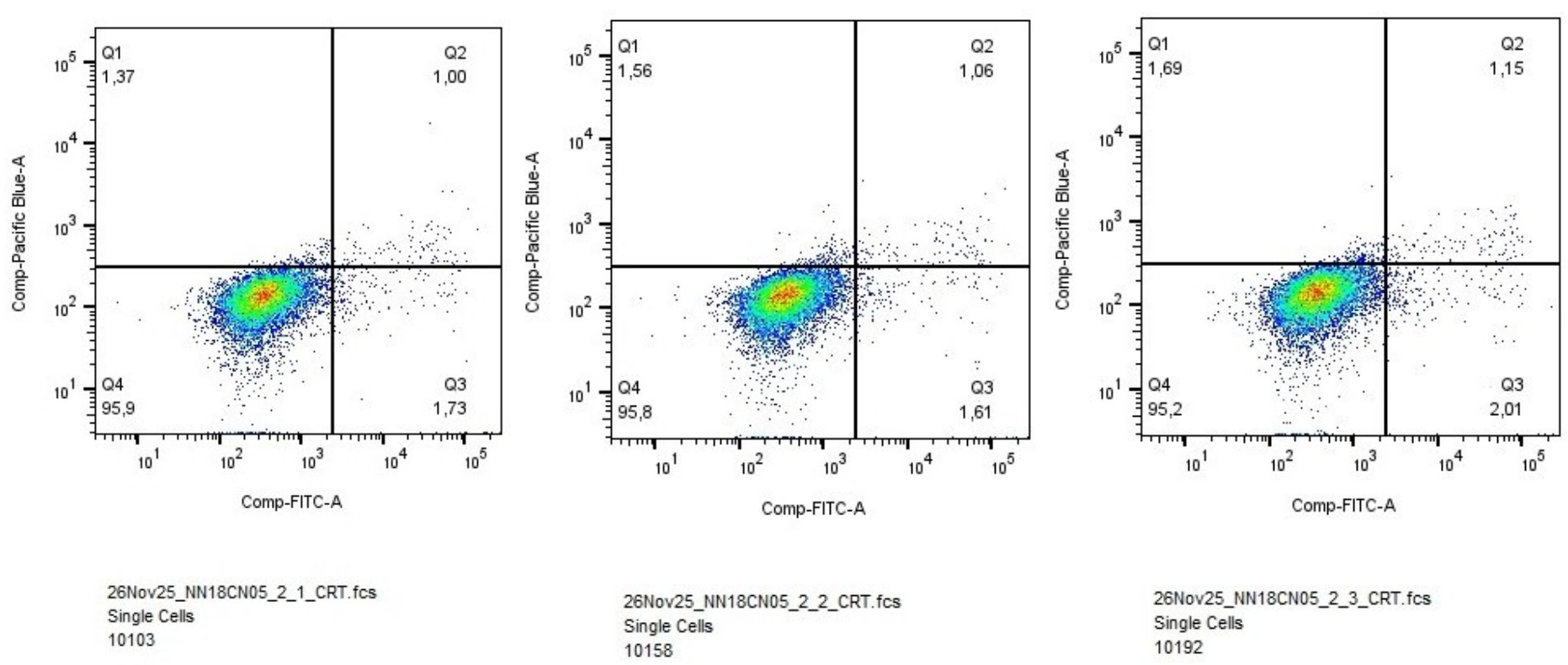

Figure S76: Flow cytometry dot plots showing CRT surface expression versus cell viability, after treatment with $[Ir(CN08)_2(NN18)]^+$ (2 μM).
